# Intramolecular C(sp^3^)–H Bond Oxygenation by Transition‐Metal Acylnitrenoids

**DOI:** 10.1002/anie.202009335

**Published:** 2020-09-24

**Authors:** Yuqi Tan, Shuming Chen, Zijun Zhou, Yubiao Hong, Sergei Ivlev, K. N. Houk, Eric Meggers

**Affiliations:** ^1^ Fachbereich Chemie Philipps-Universität Marburg Hans-Meerwein-Straße 4 35043 Marburg Germany; ^2^ Department of Chemistry and Biochemistry University of California Los Angeles CA 90095-1569 USA

**Keywords:** C−H functionalization, enantioselectivity, nitrenoids, oxygenation, ruthenium

## Abstract

This study demonstrates for the first time that easily accessible transition‐metal acylnitrenoids can be used for controlled direct C(sp^3^)‐H oxygenations. Specifically, a ruthenium catalyst activates N‐benzoyloxycarbamates as nitrene precursors towards regioselective intramolecular C−H oxygenations to provide cyclic carbonates, hydroxylated carbamates, or 1,2‐diols. The method can be applied to the chemoselective C−H oxygenation of benzylic, allylic, and propargylic C(sp^3^)−H bonds. The reaction can be performed in an enantioselective fashion and switched in a catalyst‐controlled fashion between C−H oxygenation and C−H amination. This work provides a new reaction mode for the regiocontrolled and stereocontrolled conversion of C(sp^3^)‐H into C(sp^3^)−O bonds.

## Graphical abstract

Transition‐metal nitrenoids are well‐established for their C−H amination chemistry but this work demonstrates that N‐acylnitrenoids can surprisingly undergo C(sp^3^)–H oxygenations instead, even in an enantioselective fashion.

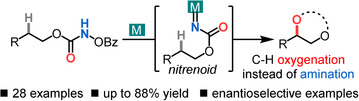

## Introduction

**Figure 1:**
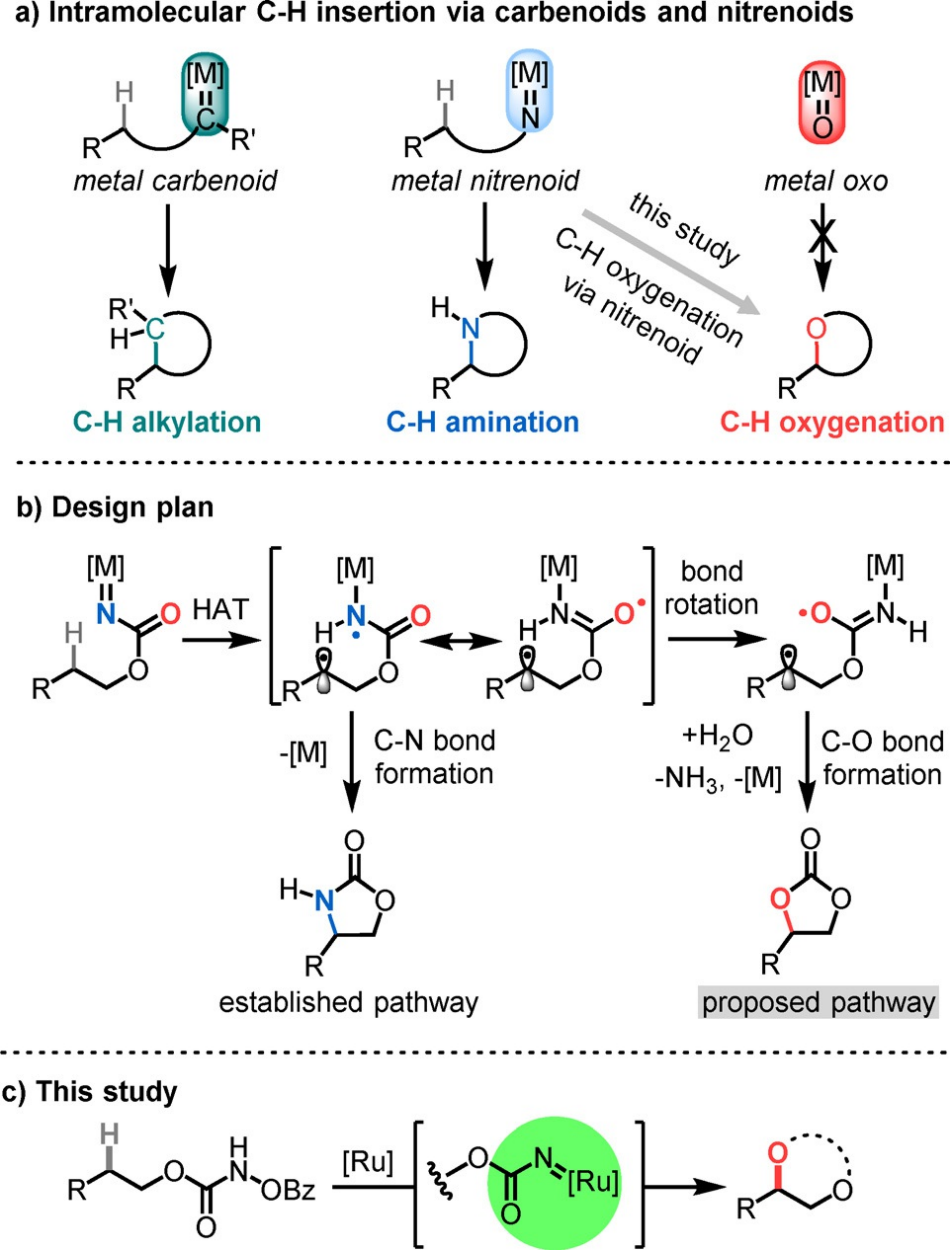
Intramolecular C(sp^3^)–H insertion through intermediate transition metal carbenoids and nitrenoids and design plan for this study.

The direct functionalization of non‐activated C(sp^3^)−H bonds represents an atom‐ and step‐economic strategy for chemical synthesis, opening new opportunities for the streamlined synthesis of complex organic molecules.^[1]^ One attractive mechanistic scenario proceeds through the direct or stepwise insertion of transition metal carbenoids (M=CR_2_), nitrenoids (M=NR), or transition‐metal oxo species (M=O) into C−H bonds (Figure 1 a).^[2]^ Intramolecular, ring‐closing versions of transition metal carbenoid^[3]^ and nitrenoid^[4]^ C−H insertions are of particular current interest because they provide a synthetic tool for the regio‐ and stereocontrolled alkylation and amination of C(sp^3^)−H bonds under typically very mild reaction conditions and without the requirement for directing groups. Analogous intramolecular C(sp^3^)‐H oxygenations^[5]^ through metal oxo species would be highly desirable but are unfortunately not feasible owing to the lower valence of oxygen relative to nitrogen and carbon.

We hypothesized that intramolecular C−H oxygenations might be feasible from well‐known metal N‐acylnitrenoid intermediates as shown in Figure 1 b. The design plan assumes a triplet metal nitrenoid which abstracts intramolecularly a hydrogen from a C(sp^3^)−H bond to form a diradical intermediate. The established pathway continues with a C−N bond formation under release of the metal catalyst. However, we envisioned that under certain circumstances, the lifetime of this intermediate might be long enough for a conformational reorganization of the radical intermediate. Considering that the spin at the nitrogen should be delocalized over the adjacent carbonyl group, we expected that a C−O bond formation could occur instead of the standard C−N bond formation. Such a C(sp^3^)–H oxygenation pathway through an intermediate transition metal nitrenoid has been elusive.

Herein we introduce a ring‐closing C(sp^3^)–H oxygenation that proceeds through a transition‐metal nitrenoid and permits a regioselective intramolecular C−H oxygenation to generate cyclic carbonates, hydroxylated carbamates, or 1,2‐diols (Figure 1 c).

## Results and Discussion

We commenced our study with the substrate **1 aa**, which bears a tosylate leaving group at a carbamate nitrogen (Table 1). Such N‐tosyloxycarbamates were previously introduced by Lebel^[6]^ as a source of metal nitrenes for C−H insertions and aziridinations and Davies^[7]^ subsequently reported an enantioselective intramolecular C−H amination with a chiral dirhodium tetracarboxylate catalyst to provide non‐racemic cyclic carbamates. We instead started with a recently developed class of ruthenium catalysts in which two bidentate N‐(2‐pyridyl)‐substituted N‐heterocyclic carbenes and two acetonitrile ligands are coordinated to the ruthenium center in a *C*_2_‐symmetric fashion.^[8]^ A C(sp^3^)–H amination should afford cyclic carbamate **2 a** whereas the desirable C(sp^3^)‐oxygenation should furnish instead the cyclic carbonate **3 a**. When we reacted N‐tosyloxycarbamate **1 aa** with catalytic amounts of a catalyst (2.0 mol %) containing CF_3_ substituents at the coordinated pyridine ligands (**RuCF_3_**)^[9]^ in the presence of the base K_2_CO_3_, carbamate **2 a** was isolated in 30 % yield but the desired carbonate **3 a** could not be detected (entry 1). Switching to a benzoate leaving group (**1 ab**) afforded the carbamate **2 a** in 87 % yield, thus revealing a very efficient intramolecular C(sp^3^)–H amination (entry 2).

**Table 1:** Initial experiments and optimization of reaction conditions.^[a]^ 
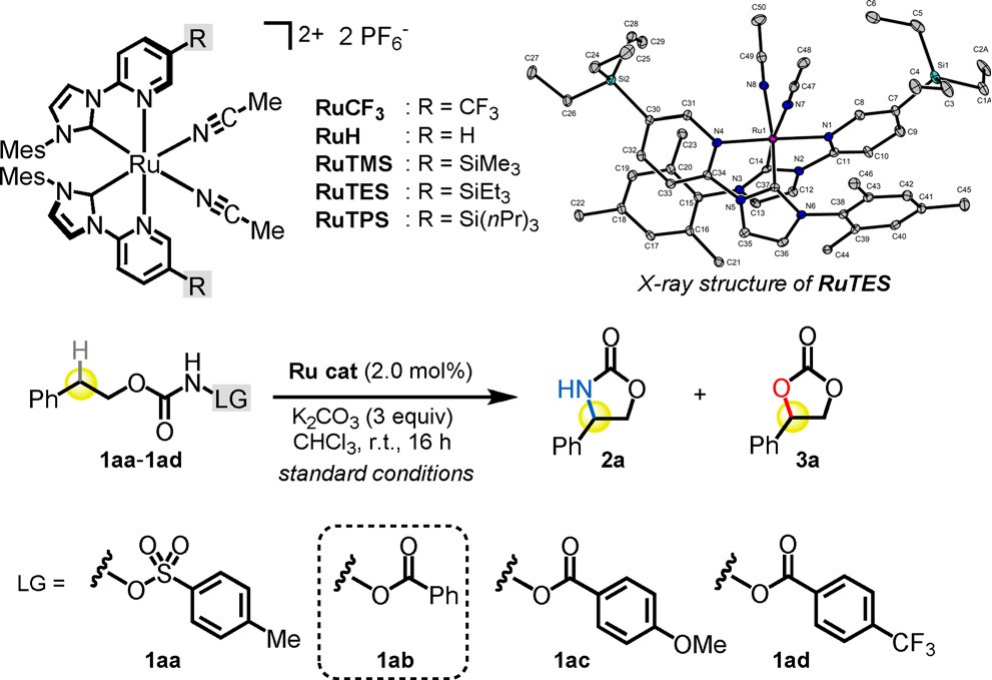

Entry	Catalyst	Substrate	Conditions^[b]^	Conv. [%]^[c]^	Yield [%]^[d]^	
					**2 a**	**3 a**
1	**RuCF_3_**	**1 aa**	standard	100	30	0
2	**RuCF_3_**	**1 ab**	standard	100	87	4
3	**RuH**	**1 ab**	standard	100	68	18
4	**RuTMS**	**1 ab**	standard	100	17	79
5	**RuTES**	**1 ab**	standard	100	11	85
6	**RuTPS**	**1 ab**	standard	88	12	73
7	**RuTES**	**1 ac**	standard	100	10	76
8	**RuTES**	**1 ad**	standard	84	14	35
9	**RuTES**	**1 ab**	1.0 mol % cat.	79	12	62
10	**RuTES**	**1 ab**	1 % H_2_O added	100	12	81
11	**RuTES**	**1 ab**	4 Å MS added	100	14	76
12	**RuTES**	**1 ab**	under air	58	10	47
13	**RuTES**	**1 ab**	no base	12	5	2

[a] Standard reaction conditions: Substrates **1 aa**–**1 ad** (0.2 mmol), K_2_CO_3_ (0.6 mmol), Ru catalyst (0.004 mmol) in CHCl_3_ (4.0 mL) stirred at room temperature for 16 h under an atmosphere of N_2_ unless noted otherwise, then washed with water, and stirred with 1 N HCl (0.2 mL, 0.2 mmol) for 15 min. [b] Deviations from standard conditions shown. [c] Determined by ^1^H NMR of the crude products using 1,3,5‐trimethoxybenzene as internal standard. [d] Isolated yields.

Encouragingly, small amounts (4 %) of the desired carbonate **3 a** could also be identified. We next attempted to increase the yield of the desired carbonate by modifying the catalyst. Accordingly, using a ruthenium catalyst with plain N‐(2‐pyridyl)‐substituted N‐heterocyclic carbene ligands (**RuH**) improved the yield of the carbonate **3 a** to 18 % (entry 3). Increasing the steric bulk by introducing a trimethylsilyl (TMS) group into the pyridine ligand (**RuTMS**) further raised the yield of the carbonate **3 a** to 79 % (entry 4). Finally, using an even more bulky triethylsilyl (TES) group (**RuTES**, see X‐ray structure) provided the optimal result with the formation of carbonate **3 a** in 85 % isolated yield (entry 5). Increasing the bulkiness of the catalyst further by replacing the TES group with a tri‐*n*‐propylsilyl group (**RuTPS**) did not provide improved results but a lower catalytic activity (entry 6). Some control experiments were performed. Introducing an electron‐donating methoxy (**1 ac**) or electron‐withdrawing CF_3_ (**1 ad**) group into the benzoyl moiety resulted in reduced yields (entries 7 and 8). Reducing the catalyst loading, adding water or molecular sieves, or performing the reaction under air resulted in a reduction of the carbonate yield (entries 9–12). Finally, without base the reaction proceeded very sluggish (entry 13).

**Figure 2:**
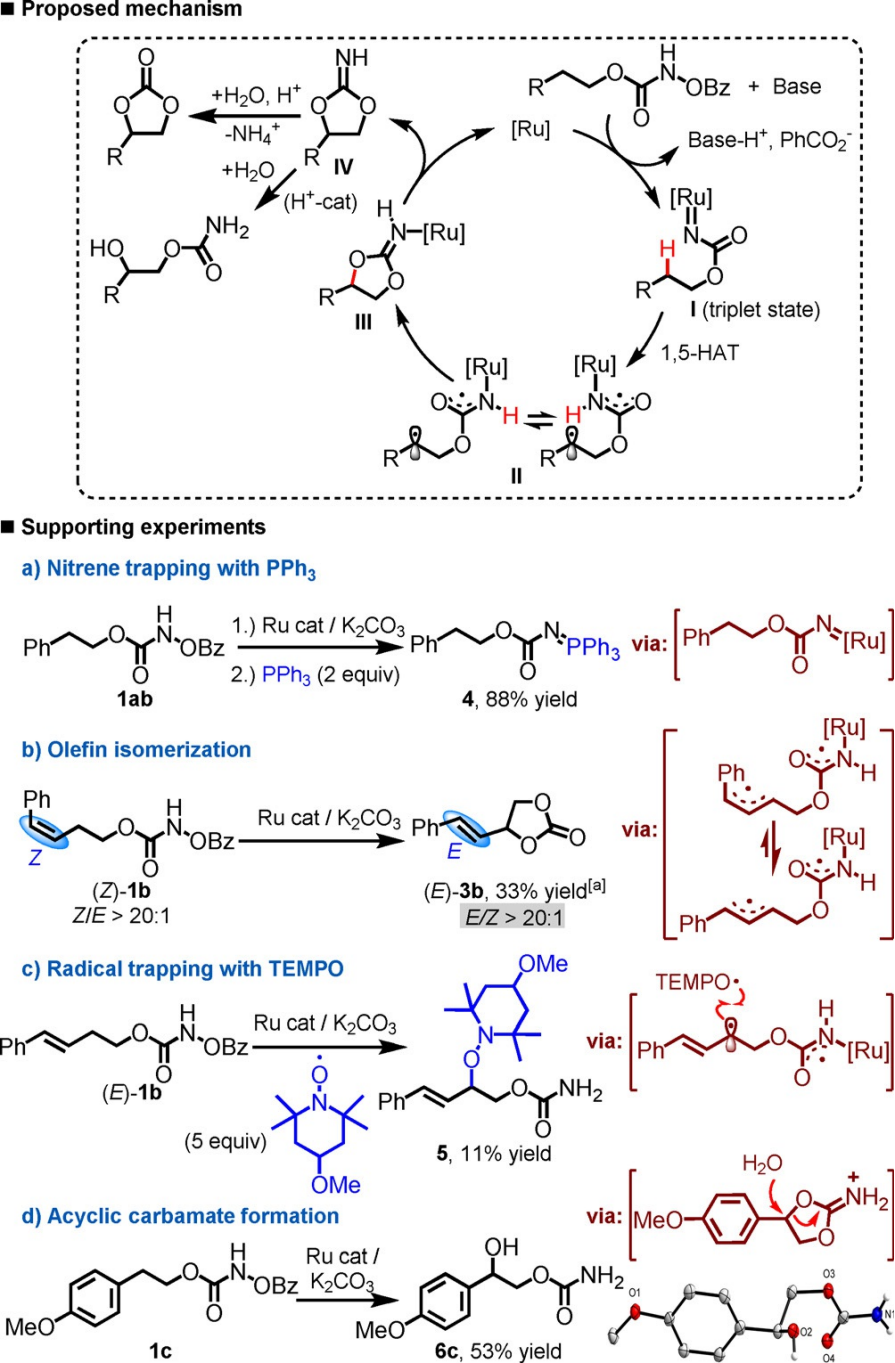
Proposed mechanism and supporting experiments. Reactions performed under standard conditions shown in Table 1. [a] Formed together with the corresponding acyclic carbamate. See the Supporting Information for more details.

The proposed mechanism starts with the reaction of the ruthenium catalyst with the N‐benzoyloxycarbamate substrate **1** under base‐promoted release of benzoic acid to provide a ruthenium nitrenoid intermediate (**I**, Figure 2).^[10]^ This nitrenoid species subsequently performs a 1,5‐hydrogen atom transfer (HAT) from the benzylic C−H bond to the nitrenoid nitrogen to generate the diradical **II**. In this diradical intermediate **II**, the nitrogen radical is conjugated to the adjacent carbonyl group so that a delocalization of the spin can be expected. If the lifetime of this diradical is long enough, a conformational change will now allow a radical‐radical recombination with the oxygen moiety of the ruthenium N‐coordinated amide, thereby forming a new C−O bond (**III**). This is consistent with the observed trend that a more sterically crowded catalyst active site shifts the otherwise preferred C‐N to the previously elusive C−O bond formation. Presumably the competing C−N bond formation is suppressed by the bulky ruthenium catalyst which is directly coordinated to the nitrogen while the oxygen is more remote and thus less sensitive to steric effects. The formed iminocarbonate **III** then dissociates from the ruthenium and provides the cyclic carbonate **3** upon hydrolysis of the exocyclic imine.

Experimental and computational results validate the proposed mechanism. Ruthenium nitrenoid species have been reported to efficiently transfer the nitrene unit to phosphines and sulfides.^[11]^ Indeed, when we performed the ruthenium‐catalyzed reaction with substrate **1 ab** under standard conditions in the presence of 2 equivalents of PPh_3_, the iminophosphorane **4** was formed in 88 % yield so that this experiment supports the intermediate formation of the ruthenium nitrenoid species **I** (Figure 2 a). Experimental support for a radical pathway comes from a reaction with the alkene substrate (*Z*)‐**1 b** which was converted to the cyclic carbonate (*E*)‐**3 b** under complete *Z*→*E* isomerization of the configuration (Figure 2 b).[^12^, ^13^] This can be explained with a conversion of the *Z*‐isomer to the thermodynamically more stable *E*‐isomer at the stage of the diradical intermediate **II**, which for the alkene substrate **1 b** involves a configurationally fluctional allylic radical. A trapping experiment of substrate (*E*)‐**1 b** with 4‐methoxy‐TEMPO which afforded the TEMPO adduct **5** (11 %) provides a further indication for a radical pathway (Figure 2 c). Finally, density functional theory (DFT) calculations were performed and provide additional support for the proposed radical mechanism with a 1,5‐HAT from a triplet state of the ruthenium intermediate **I** being the favored pathway (**I**→**II**) (see Supporting Information for details on the calculations).[^15^, ^16^, ^17^]

Evidence for the intermediate formation for the iminocarbonate **IV**, which we were not able to isolate, stems from a reaction shown in Figure 2 d. When we used substrate **1 c**, in which the phenyl group is functionalized with a *para*‐methoxy group, we obtained under ruthenium catalysis the carbamate **6 c** instead of the cyclic carbonate. This product can be rationalized by a ring opening hydrolysis of the protonated intermediate iminocarbonate **IV**.^[14]^ In summary, the control experiments provide strong support for the proposed radical pathway, which can explain the competing C−O and C−N bond formations.

**Figure 3:**
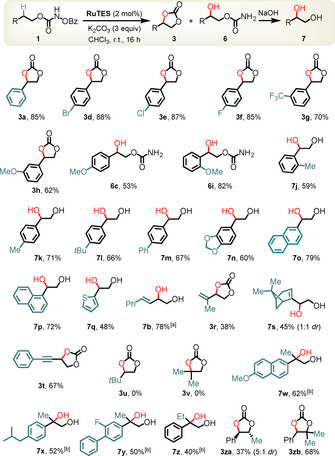
Substrate scope for C(sp^3^)–H oxygenations. Conditions for the optional basic hydrolysis: Dioxane/ NaOH (1 N) 1:1 at 80 °C for 2 hours. [a] From (*E*)‐**1 b**. Starting from (*Z*)‐**1 b**, the yield is 72 %. [b] Performed at 60 °C.

With the optimal conditions in hand, we investigated the scope of this reaction. N‐Benzoyloxycarbamates derived from 2‐phenylethanol with substituents in the phenyl moiety were investigated first. As shown in Figure 3, cyclic carbonates with electron‐withdrawing bromine (**3 d**, 88 %), chlorine (**3 e**, 87 %), or fluorine (**3 f**, 85 %) substituents in *para*‐position of the phenyl moiety were obtained in high yields. Substituents are also accommodated in *meta*‐position as demonstrated for an electron‐withdrawing CF_3_ (**3 g**, 70 %) and an electron‐donating methoxy group (**3 h**, 62 %). As already discussed in the mechanistic section (Figure 2), for a substrate with a methoxy substituent in the *para*‐position of the phenyl moiety, the intermediate iminocarbonate hydrolyzes to a ring‐opened C−H hydroxylated carbamate instead of forming a cyclic carbonate (**1 c** → **6 c**, 53 % yield). A methoxy group in *ortho*‐position of the phenyl ring increases the yield of the C−H hydroxylated carbamate to 82 % (**6 i**). For some substrates, mixtures of cyclic carbonate and C−H hydroxylated carbamate were observed. For those cases, a basic hydrolysis was applied before silica gel chromatography to isolate the corresponding diols. For example, 1‐aryl‐1,2‐ethanediols were obtained with the aryl group being *ortho*‐tolyl (**7 j**, 59 % yield), *para*‐tolyl (**7 k**, 71 % yield), *para*‐*tert*‐butylphenyl **7 l**, 66 %), 4‐biphenyl (**7 m**, 67 %), 1,3‐benzodioxol‐5‐yl (**7 n**, 60 %), 2‐naphthyl (**7 o**, 79 %), or 1‐naphthyl (**7 p**, 72 %). A 2‐thiophene moiety leads to lower 48 % yield (**7 q**). The oxygenated C(sp^3^)−H bond can also be flanked by an alkenyl group. Both *E*‐ and *Z*‐configured alkenes **1 b** afford the diol (*E*)‐**7 b** in a diastereoconvergent reaction in 78 % and 72 % yield, respectively. Additional examples for an allylic C−H hydroxylation are the obtained isopropenyl substituted cyclic carbonate **3 r** (38 %) and the hydroxylated terpene nopol **7 s** (45 % yield). C−H oxygenation in propargylic position is also feasible as shown for the cyclic carbonate **3 t** (67 %), whereas unactivated C−H groups do not provide any C−H oxygenation product (such as **3 u** and **3 v**, 0 %). However, the C−H oxygenation is applicable to tertiary C−H groups as shown for compounds derived from the drugs naproxen (**7 w**, 62 %), ibuprofen (**7 x**, 52 %), flurbiprofen (**7 y**, 50 %), and the sterically congested tertiary alcohol **7 z** (40 %). N‐Benzoyloxycarbamates of 2‐phenylethanol with one (**3 za**, 37 %) or two methyl substituents (**3 zb**, 68 %) in the 1‐position also provide the cyclic carbonate. Overall, the developed C−H oxygenation can be applied to benzylic, allylic, and propargylic secondary and tertiary C(sp^3^)−H bonds with yields of up to 88 %. A few substrates provide only modest C−H oxygenation yields which can be explained with a certain degree of competing C−H amination. Depending on the nature of the substrate, the intermediate iminocarbonate either hydrolyzes to the cyclic carbonate, the hydroxylated carbamate, or a mixture of both. Both can be converted to the corresponding vicinal diols by a brief basic hydrolysis, resulting in an overall vicinal C(sp^3^)–H hydroxylation of the initial alcohol starting materials.

**Figure 4:**
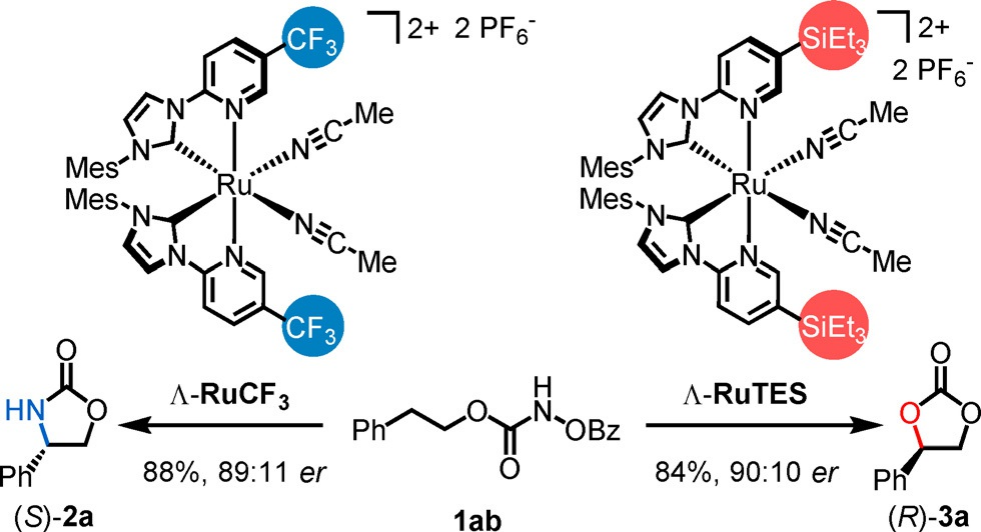
Enantioselective intramolecular C(sp^3^)–H oxygenation and amination. Standard reaction conditions: Catalyst (2 mol %), K_2_CO_3_ (3 equivalents), in CHCl_3_ at room temperature for 16 hours.

In preliminary experiments, we also started to investigate the enantioselective version of this C−H functionalization. The class of ruthenium catalysts used in this study, although containing only achiral ligands, feature a stereogenic metal center, which results in overall chirality with a Λ (left‐handed helical twist of the bidentate ligands) and Δ (right‐handed helical twist) enantiomer.^[18]^ Whereas for all preceding experiments a racemic mixture of **RuTES** was employed, we next synthesized non‐racemic **RuTES** according to a recently developed chiral‐auxiliary‐mediated method.^[8]^ Indeed, when enantiomerically pure Λ‐**RuTES** (2.0 mol %) was reacted with the N‐benzoyloxycarbamate **1 ab** under standard conditions, the cyclic carbonate (*R*)‐**3 a** was obtained with an enantiomeric excess of 90:10 *er* (Figure 4). Interestingly, when the same nitrene precursor **1 ab** was instead reacted with the enantiomerically pure catalyst Λ‐**RuCF_3_** (2.0 mol %) under identical reaction conditions, the cyclic carbamate (*S*)‐**2 a** was obtained in 88 % yield and with 89:11 *er*. Thus, depending on a single functional group at the catalyst, either an enantioselective C−H oxygenation or enantioselective C−H amination[^19^, ^20^, ^21^] can be obtained starting from an identical nitrene precursor. A catalyst‐dependent switch between C−H amination and oxygenation was recently also reported by Lu and co‐workers in a photoredox dual catalysis reaction from N‐benzoyloxycarbamates.^[22]^ White and co‐workers reported a catalyst‐controlled diastereoselective allylic C−H amination versus oxygenation using a combination of palladium(II) catalyst and Lewis acid.^[23]^ However, experiments in both reports were not performed in an enantioselective fashion and occurred by different mechanisms.

## Conclusion

We here introduced a novel reactivity of transition metal nitrenoids complexes, leading to C−H oxygenation instead of the expected and established C−H nitrogenation. Metal‐catalyzed intramolecular carbene and nitrene insertion reactions previously only allowed for the formation of C−C and C−N bonds, respectively, whereas the here reported work expands on this limitation. Furthermore, we demonstrated that the reactivity of the transition metal nitrenoid towards C−H oxygenation or nitrogenation can be tuned simply by changing one substituent on the ruthenium catalyst scaffold and also provides a handle for creating new stereogenic centers in an enantioselective fashion. We believe that this new reaction mode of transition metal nitrenoids will provide untapped opportunities for the streamlined synthesis of alcohols by regiocontrolled and stereocontrolled C(sp^3^)–H oxygenation. Furthermore, cyclic carbonates are useful building blocks for a variety of transformations.^[24]^

## Conflict of interest

The authors declare no conflict of interest.

## Supporting information

As a service to our authors and readers, this journal provides supporting information supplied by the authors. Such materials are peer reviewed and may be re‐organized for online delivery, but are not copy‐edited or typeset. Technical support issues arising from supporting information (other than missing files) should be addressed to the authors.

Supplementary
