# Supplementary material for: Intramolecular C(sp3)–H Bond Oxygenation by Transition‐Metal Acylnitrenoids
Source: Angew Chem Int Ed Engl. 2020 Sep 24;59(48):21706–10. doi: 10.1002/anie.202009335 (PMC7756817; doi:10.1002/anie.202009335)
Supplement: Supplementary file 1 — Supplementary [file ANIE-59-21706-s001.pdf]

Angewandte Chemie
Eine Zeitschrift der Gesellschaft Deutscher Chemiker

Supporting Information

Intramolecular C(sp^3)–H Bond Oxygenation by Transition-Metal Acylnitrenoids

Yuqi Tan, Shuming Chen, Zijun Zhou, Yubiao Hong, Sergei Ivlev, K. N. Houk, and Eric Meggers**

anie_202009335_sm_miscellaneous_information.pdf

Table of Contents

1. General Information S2

2. Synthesis of the Ruthenium Catalysts RuTES and RuTPS S3

3. Synthesis of Substrates S7

4. Experimental and Characterization Data of Products S21

5. Stereochemical Aspects of Intramolecular C(sp^3)-H Oxygenation *versus* Nitrogenation S34

5.1 Enantioselective Cyclic Carbonate Formation by C(sp^3)-H Oxygenation S34
5.2 Racemic Formation of Acyclic Carbamate by C(sp^3)-H Oxygenation S37
5.3 C-H Oxygenation and C-H Amination at a Stereocenter with Racemic Catalyst S39

6. Mechanistic Experiments S41

6.1 Trapping of Ru-Nitrenoid Intermediate S41
6.2 Olefin Isomerization S41
6.3 Trapping of Radical Intermediate with 4-MeO-TEMPO S42

7. Single Crystal X-Ray Diffraction S43

7.1 Single Crystal X-Ray Analysis of *rac*-**RuTES** S43
7.2 Single Crystal X-Ray Analysis of **6c** S45

8. Enantioselectivities as Determined by Chiral HPLC S47

9. NMR Spectra S60

10. Computational Study S134

10.1 Computational Results S134
10.2 Computational Methods S135
10.3 Calculated Energies with Different DFT Methods S136
10.4 Cartesian Coordinates of Computed Structures S137

11. References S147

1. General Information

All reactions were carried out under an atmosphere of nitrogen with magnetic stirring unless otherwise indicated. Catalytic reactions were performed in a Schlenk tube (10 mL). Solvents were distilled under nitrogen from calcium hydride (CH_3CN and CH_2Cl_2) or sodium/benzophenone (THF and toluene). HPLC grade of diethyl ether, $CHCl_3$ were used without further purification. Reagents that were purchased from commercial suppliers were used without further purification. Flash column chromatography was performed with silica gel 60 M from Macherey-Nagel (irregular shaped, 230-400 mesh, pH 6.8, pore volume: 0.81 mL x g^{-1}, mean pore size: 66 Å, specific surface: 492 $m^2 \times g^{-1}$, particle size distribution: 0.5% < 25 μm and 1.7% > 71 μm, water content: 1.6%). ^{1}H NMR, proton decoupled ^{13}C NMR spectra and proton-coupled ^{19}F NMR spectra were recorded on Bruker Avance 250 (250 MHz), Bruker Avance 300 (300 MHz) or Bruker Avance 500 (500 MHz) spectrometers at ambient temperature. NMR standards were used as follows: ^{1}H NMR spectroscopy: δ = 7.26 ppm ($CDCl_3$), δ = 5.35 ppm (CD_2Cl_2), δ = 1.94 ppm (CD_3CN). ^{13}C NMR spectroscopy: δ = 77.2 ppm ($CDCl_3$), δ = 53.8 ppm (CD_2Cl_2), δ = 1.32 ppm (CD_3CN); ^{19}F NMR spectroscopy: δ = 0 ppm ($CFCl_3$). IR spectra were recorded on a Bruker Alpha FT-IR spectrophotometer. High-resolution mass spectra were recorded on a Bruker En Apex Ultra 7.0 TFT-MS instrument. Enantiometric purities of the reaction products were determined with Daicel HPLC columns (Chiralpak OD-H, AD-H, IG or IA with particle size of 5 μm and column size of 250 × 4.6 mm) on an Agilent 1260 Series HPLC System using *n*-hexane/isopropanol as the mobile phase. The column temperature was 25 °C and UV absorption was measured at 210 nm, 216 nm or 254 nm. Optical rotations were measured on a Krüss P8000-T polarimeter with $[\alpha]_D^{22}$ values reported in degrees with concentrations reported in g/100 mL.

2. Synthesis of the Ruthenium Catalysts RuTES and RuTPS

The ruthenium catalysts were synthesized according to our published procedures with slightly modifications.[1,2] The catalysts **RuH**,[1] **$RuCF_3$**,[2] **RuTMS**[2] were reported by us previously. The synthesis of the catalysts **RuTES** and **RuTPS** is shown below.

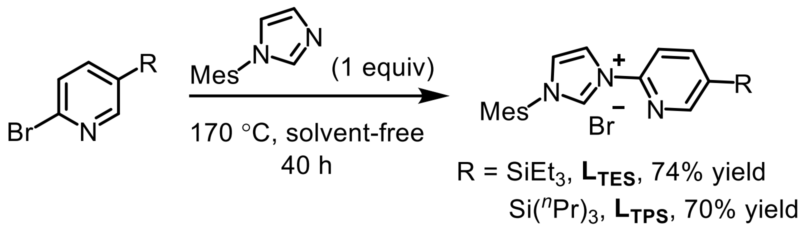

Synthesis of the imidazolium ligand $\mathbf{L_{TES}}$: *N*-Mesitylimidazole (391 mg, 2.1 mmol) and 2-bromo-5-(triethylsilyl)pyridine[3] (572 mg, 2.1 mmol) were stirred in a sealed tube at 170 °C for 40 hours. After cooling to room temperature, the resulting brown solid was washed with diethyl ether for several times until no starting materials were detectable by TLC, and then the solvent was removed to give pure ligand as a pale brown solid (713 mg, 74% yield).

^{1}H NMR (300 MHz, $CDCl_3$) δ 11.38 (s, 1H), 9.21 (d, J = 8.1 Hz, 1H), 8.94-8.92 (m, 1H), 8.52 (m, 1H), 8.18 (dd, J = 8.1 Hz, 1.8 Hz, 1H), 7.34 (m, 1H), 7.03 (s, 2H), 2.34 (s, 3H), 2.17 (s, 6H), 0.99-0.93 (m, 9H), 0.87-0.78 (m, 6H).

^{13}C NMR (75 MHz, $CDCl_3$) δ 153.4, 147.1, 146.4, 141.8, 136.3, 135.8, 134.2, 130.7, 130.2, 124.0, 120.2, 115.7, 21.3, 18.0, 7.3, 3.2.

IR (film): ν (cm^{-1}) 2909, 2873, 1581, 1534, 1481, 1459, 1368, 1331, 1307, 1269, 1243, 1127, 1007, 963, 875, 762, 754, 733, 698, 671, 636, 585, 525.

HRMS (ESI, *m/z*) calcd for $C_{23}H_{32}N_3Si$ [M−Br]$^+$: 378.2360, found: 378.2369.

Synthesis of the imidazolium ligand $\mathbf{L_{TPS}}$: *N*-Mesitylimidazole (391 mg, 2.1 mmol) and 2-bromo-5-(tripropylsilyl)pyridine (660 mg, 2.1 mmol) were stirred in a sealed tube at 170 °C for 40 hours. After cooling to room temperature, the resulting brown solid was washed with diethyl ether for several times until no starting materials were detectable by TLC, and then the solvent was removed to give pure ligand as a pale brown solid (618 mg, 70% yield).

^{1}H NMR (300 MHz, $CDCl_3$) δ 11.43 (s, 1H), 9.23 (d, J = 8.2 Hz, 1H), 8.93 (t, J = 1.8 Hz, 1H), 8.52 (m, 1H), 8.19 (dd, J = 8.2 Hz, 1.7 Hz, 1H), 7.30 (m, 1H), 7.05 (s, 2H), 2.35 (s, 3H), 2.18 (s, 6H), 1.41-1.25 (m, 6H), 0.97 (t, J = 7.2 Hz, 9H), 0.86-0.80 (m, 6H).

^{13}C NMR (75 MHz, $CDCl_3$) δ 153.3, 147.1, 146.4, 141.8, 136.7, 136.4, 134.2, 130.8, 130.3, 123.9, 120.1, 115.6, 21.3, 18.5, 18.0, 17.4, 15.0.

IR (film): ν (cm^{-1}) 2952, 2922, 2865, 1582, 1532, 1482, 1459, 1366, 1330, 1305, 1264, 1243, 1128, 1068, 963, 874, 754, 671, 585, 521.

HRMS (ESI, *m/z*) calcd for $C_{26}H_{38}N_3Si$ [M−Br]$^+$: 420.2830, found: 420.2828.

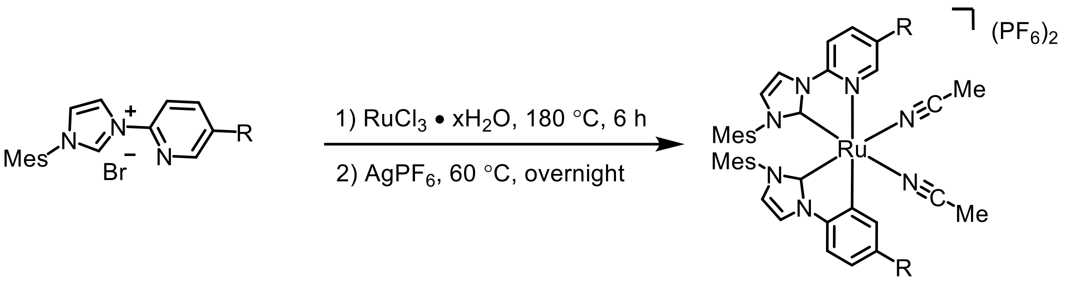

R = SiEt$_3$, *rac*-**RuTES**, 56% yield
Si(^{n}Pr)$_3$, *rac*-**RuTPS**, 55% yield

Synthesis of *rac*-RuTES: A solution of $RuCl_3$•xH_2O (52 mg, 0.25 mmol) and ligand **L$_{TES}$** (229 mg, 0.5 mmol) in ethylene glycol (5.0 mL) was heated at 180 °C for 6 hours. The reaction mixture was treated with saturated aqueous NH_4PF_6 after cooling down to room temperature. A yellow precipitate was formed, which was dissolved and extracted with CH_2Cl_2 for 3 times. The combined organic layers were washed with water and concentrated under reduced pressure to obtain an orange solid, which was dissolved in CH_3CN (5.0 mL) followed by adding $AgPF_6$ (0.3 mmol, 76 mg,). The mixture was stirred at 60 °C overnight. After cooling to room temperature, the mixture was filtered, and the filtrate was collected, evaporated to dryness and purified by column chromatography on silica gel (CH_2Cl_2/CH_3CN = 300:1 to 50:1) to give pure racemic ruthenium catalyst as a yellow solid (172 mg, 56% yield).

^{1}H NMR (300 MHz, CD_2Cl_2) δ 8.43 (m, 2H), 7.98 (d, J = 2.3 Hz, 2H), 7.87 (dd, J = 8.1 Hz, 1.4 Hz, 2H), 7.48 (d, J = 8.1 Hz, 2H), 6.89 (d, J = 2.3 Hz, 2H), 6.67 (s, 2H), 6.60 (s, 2H), 2.26 (s, 6H), 2.16 (s, 6H), 1.95 (s, 6H), 1.53 (s, 6H), 1.10-1.05 (m, 18H), 0.94-0.86 (m, 12H).

^{13}C NMR (75 MHz, CD_2Cl_2) δ 189.9, 155.2, 153.6, 144.4, 140.1, 135.0, 134.1, 133.9, 132.7, 129.74, 129.66, 125.6, 124.3, 117.7, 111.1, 21.1, 17.64, 17.55, 7.5, 3.6, 3.3.

IR (film): ν (cm^{-1}) 2955, 2879, 1596, 1495, 1421, 1331, 1260, 1155, 1134, 1012, 932, 833, 769, 723, 666, 556.

Synthesis of *rac*-RuTPS: A solution of $RuCl_3$•xH_2O (52 mg, 0.25 mmol) and ligand **L$_{TPS}$** (250 mg, 0.5 mmol) in ethylene glycol (5.0 mL) was heated at 180 °C for 6 hours. The reaction mixture was

treated with saturated aqueous NH_4PF_6 after cooling down to room temperature. A yellow precipitate was formed, which was dissolved and extracted with CH_2Cl_2 for 3 times. The combined organic layers were washed with water and concentrated under reduced pressure to obtain an orange solid, which was dissolved in CH_3CN (5.0 mL) followed by adding $AgPF_6$ (0.3 mmol, 76 mg,). The mixture was stirred at 60 °C overnight. After cooling to room temperature, the mixture was filtered, and the filtrate was collected, evaporated to dryness and purified by column chromatography on silica gel (CH_2Cl_2/CH_3CN = 300:1 to 50:1) to give pure racemic ruthenium catalyst as a yellow solid (180 mg, 55% yield).

^{1}H NMR (300 MHz, CD_2Cl_2) δ 8.41 (m, 2H), 7.99 (d, J = 2.3 Hz, 2H), 7.86 (dd, J = 8.2 Hz, 1.5 Hz, 2H), 7.47 (d, J = 8.2 Hz, 2H), 6.88 (d, J = 2.3 Hz, 2H), 6.67 (s, 2H), 6.59 (s, 2H), 2.26 (s, 6H), 2.17 (s, 6H), 1.95 (s, 6H), 1.53 (s, 6H), 1.49-1.38 (m, 12H), 1.05 (t, J = 7.2 Hz, 18H), 0.91-0.86 (m, 12H).

^{13}C NMR (75 MHz, CD_2Cl_2) δ 189.8, 155.1, 153.5, 144.3, 140.1, 135.0, 134.1, 133.9, 133.4, 129.8, 129.6, 125.6, 124.2, 117.8, 111.1, 21.1, 18.7, 17.8, 17.6, 17.5, 15.3, 3.3.

IR (film): ν (cm^{-1}) 2954, 2925, 2868, 1595, 1494, 1420, 1330, 1258, 1154, 1134, 1066, 931, 836, 767, 739, 689, 556.

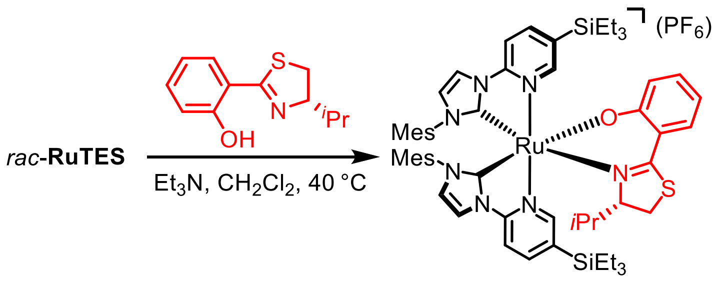

Λ-(*S*)-**RuAux** 43% yield

Synthesis of Λ-(*S*)-RuAux: A mixture of racemic ruthenium complex *rac*-**RuTES** (135.0 mg, 0.11 mmol), chiral auxiliary (*S*)-2-(4-isopropyl-4,5-dihydrothiazol-2-yl)phenol (48.6 mg, 0.22 mmol) and triethylamine (33.4 mg, 0.33 mmol) in CH_2Cl_2 (3.6 mL) was heated at 40 °C for 16 hours. The reaction mixture was cooled to room temperature and concentrated to dryness. The residue was subjected to a flash silica gel chromatography (CH_2Cl_2/CH_3CN = 50:1 to 20:1) to separate the first colorful eluent which was assigned as Λ-(*S*)-**RuAux** (red solid, 57.8 mg, 43% yield).

^{1}H NMR (300 MHz, CD_3CN) δ 8.64 (s, 1H), 8.05 (d, J = 2.3 Hz, 1H), 7.99 (s, 1H), 7.98 (d, J = 2.3 Hz, 1H), 7.77 (dd, J = 8.2 Hz, 1.4 Hz, 1H), 7.67 (dd, J = 8.1 Hz, 1.4 Hz, 1H), 7.47 (d, J = 8.2 Hz, 1H), 7.39 (d, J = 8.1 Hz, 1H), 7.25 (dd, J = 8.1 Hz, 1.6 Hz, 1H), 7.01 (d, J = 2.3 Hz, 1H), 6.93-6.88 (m, 2H), 6.62 (s, 3H), 6.52 (s, 1H), 6.35 (d, J = 8.5 Hz, 1H), 6.18-6.12 (m, 1H), 4.25-4.22 (m, 1H),

3.22-3.08 (m, 2H), 2.20 (s, 3H), 2.16 (s, 3H), 2.10 (s, 3H), 2.07 (s, 3H), 1.60 (s, 3H), 1.36 (s, 3H), 0.96-0.86 (m, 18H), 0.80-0.67 (m, 12H), 0.41 (d, J = 7.1 Hz, 3H), 0.21 (d, J = 6.9 Hz, 3H), −0.27-−0.36 (m, 1H).

^{13}C NMR (75 MHz, CD_3CN) δ 198.3, 196.3, 171.2, 169.2, 155.8, 155.6, 154.8, 154.4, 143.2, 143.0, 139.69, 139.67, 137.8, 135.8, 135.5, 135.1, 134.7, 134.1, 134.0, 130.8, 130.4, 130.3, 130.1, 130.0, 129.7, 126.4, 126.1, 124.5, 117.9, 117.6, 117.0, 113.3, 110.9, 110.5, 87.5, 32.2, 28.8, 21.3, 21.1, 20.0, 19.1, 18.4, 18.1, 17.8, 16.0, 7.82, 7.79, 4.1, 3.8.

IR (film): ν (cm^{-1}) 2953, 2875, 1591, 1549, 1519, 1491, 1461, 1441, 1416, 1322, 1281, 1241, 1196, 1149, 1133, 1007, 929, 839, 767, 719, 682, 590, 556.

CD (MeOH): λ, nm (Δε, $M^{-1}cm^{-1}$) 498 (–5), 411 (+26), 364 (–3), 340 (+21), 306 (–21), 286 (+14), 269 (–16), 238 (+36).

HRMS (ESI, m/z) calcd for $C_{58}H_{76}N_7ORuSSi_2$ $[M-PF_6]^+$: 1076.4423, found: 1076.4475.

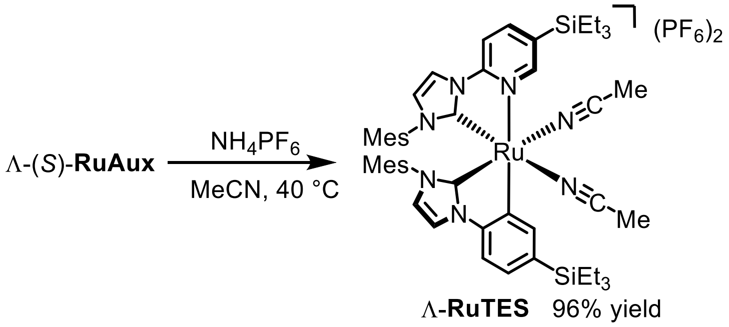

Synthesis of Λ-RuTES: The auxiliary complex Λ-(*S*)-**RuAux** (67.2 mg, 0.055 mmol) together with NH_4PF_6 (134 mg, 0.83 mmol) dissolved in 2 mL CH_3CN was heated at 40 °C for 16 hours. After that, the reaction mixture was cooled to room temperature and concentrated to dryness. The residue was dissolved in CH_2Cl_2 and filtered to remove the remaining NH_4PF_6. The filtrate was subjected to a flash silica gel chromatography (CH_2Cl_2/CH_3CN = 50:1 to 10:1) to provide analytical pure Λ-**RuTES** (yellow solid, 64.8 mg, 96% yield). All other spectroscopic data of enantiopure ruthenium catalysts were in agreement with the racemic catalysts.

CD (MeOH): λ, nm (Δε, $M^{-1}cm^{-1}$) 452 (+7), 387 (+0.4), 344 (+5), 313 (–2), 286 (+44), 270 (–28), 247 (+34), 221 (–15).

3. Synthesis of Substrates

The substrates **1aa-zb** were synthesized according to published procedures with some modifications. The analytical data of **1aa**[4] are in accordance with the literature. The data of **1ab-1ad**, **1b-zb** are provided below.

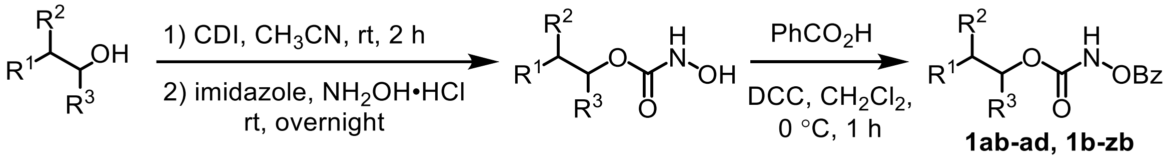

General Procedure: The substrates were prepared according to published procedures with some modifications.[5-6] 1,1'-Carbonyldiimidazole (CDI, 1.5 equiv) was added to a solution of alcohol (1.0 equiv) in acetonitrile (0.2 M) and stirred at room temperature for 2 hours. After that, imidazole (4.0 equiv) and hydroxylamine hydrochloride (5.0 equiv) were added, and stirred at room temperature overnight. The reaction was quenched using a 1 N aqueous solution of HCl (3.0 equiv). The layers were separated and the aqueous layer was extracted using EtOAc (3 x 20 mL). The organic phase was washed with brine, dried over Na_2SO_4, filtered, and concentrated in *vacuo* to afford the crude *N*-hydroxycarbamate, which was purified by flash column chromatography (*n*-hexane/EtOAc = 3:1 to 1:1). To a solution of the *N*-hydroxycarbamate (1.0 equiv) and benzoic acid (1.0 equiv) in CH_2Cl_2 (0.10 M), DCC (1.0 equiv) in CH_2Cl_2 (0.33 M) was added dropwise at 0 °C. After being stirred for 1 hours, the reaction mixture was diluted by Et_2O (10 mL/mmol). The byproduct precipitated out and was removed by filtration. The solvent was removed under cacuum and the residue was purified by column chromatography on silica gel (*n*-hexane/CH_2Cl_2 = 1:1).

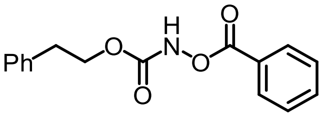

Phenethyl (*N*-benzoyloxy)carbamate (1ab)

A white solid.

^{1}H NMR (300 MHz, $CDCl_3$) δ 8.34 (s, 1H), 8.10-8.07 (m, 2H), 7.67-7.62 (m, 1H), 7.52-7.47 (m, 2H), 7.30-7.20 (m, 5H), 4.44 (t, J = 7.0 Hz, 2H), 2.99 (t, J = 7.0 Hz, 2H).

^{13}C NMR (75 MHz, $CDCl_3$) δ 166.0, 156.7, 137.4, 134.4, 130.1, 129.1, 128.9, 128.7, 126.88, 126.85,

67.4, 35.3.

IR (film): *ν* (cm^{-1}) 3192, 2957, 1771, 1725, 1601, 1492, 1450, 1283, 1259, 1236, 1163, 1109, 1080, 1008, 933, 857, 828, 776, 700, 629, 595, 475.

HRMS (ESI, *m*/*z*) calcd for $C_{16}H_{15}NO_4Na$ [M+Na]$^+$: 308.0893, found: 308.0891.

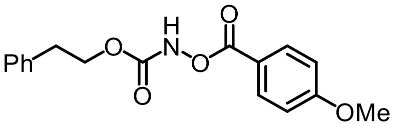

Phenethyl (*N*-(4-methoxybenzoyl)oxy)carbamate (1ac)

A white solid.

^{1}H NMR (300 MHz, $CDCl_3$) *δ* 8.25 (s, 1H), 8.07-8.02 (m, 2H), 7.31-7.20 (m, 5H), 7.00-6.94 (m, 2H), 4.43 (t, *J* = 7.0 Hz, 2H), 3.89 (s, 3H), 2.99 (t, *J* = 7.0 Hz, 2H).

^{13}C NMR (75 MHz, $CDCl_3$) *δ* 165.7, 164.6, 156.8, 137.4, 132.4, 129.1, 128.7, 126.9, 119.0, 114.2, 67.3, 55.7, 35.4.

IR (film): *ν* (cm^{-1}) 3203, 2957, 1760, 1725, 1605, 1492, 1445, 1240, 1164, 1108, 1076, 1024, 998, 966, 856, 747, 701, 633, 606, 588, 536, 496.

HRMS (ESI, *m*/*z*) calcd for $C_{17}H_{17}NO_5Na$ [M+Na]$^+$: 338.0999, found: 338.1009.

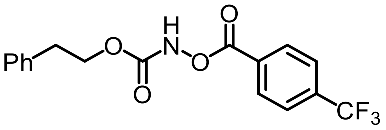

Phenethyl (*N*-(4-(trifluoromethyl)benzoyl)oxy)carbamate (1ad)

A white solid.

^{1}H NMR (300 MHz, $CDCl_3$) *δ* 8.29 (s, 1H), 8.20 (d, *J* = 8.2 Hz, 2H), 7.76 (d, *J* = 8.3 Hz, 2H), 7.31-7.19 (m, 5H), 4.45 (t, *J* = 7.0 Hz, 2H), 3.00 (t, *J* = 7.0 Hz, 2H).

^{13}C NMR (75 MHz, $CDCl_3$) *δ* 164.8, 156.4, 137.2, 135.9 (d, *J* = 32.7 Hz), 130.6, 130.2, 129.1, 128.8, 126.9, 126.0 (q, *J* = 3.7 Hz), 123.6 (d, *J* = 271.1 Hz), 67.5, 35.3.

^{19}F NMR (235 MHz, $CDCl_3$) *δ* -63.3.

IR (film): *ν* (cm^{-1}) 3216, 3119, 1768, 1702, 1514, 1413, 1325, 11688, 1154, 1108, 1051, 957, 857, 829, 769, 750, 696, 569, 493.

HRMS (ESI, *m*/*z*) calcd for $C_{17}H_{14}F_3NO_4Na$ [M+Na]$^+$: 376.0767, found: 376.0778.

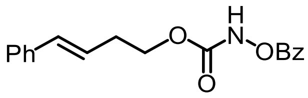

(*E*)-4-Phenylbut-3-en-1-yl (*N*-benzoyloxy)carbamate (1b)

A white solid.

^{1}H NMR (300 MHz, $CDCl_3$) δ 8.31 (s 1H), 8.09-8.06 (m, 2H), 7.65-7.60 (m, 1H), 7.49-7.44 (m, 2H), 7.34-7.18 (m, 5H), 6.47 (d, J = 15.9 Hz, 1H), 6.15 (dt, J = 15.9 Hz, 7.0 Hz, 1H), 4.35 (t, J = 6.7 Hz, 2H), 2.64-2.56 (m, 2H).

^{13}C NMR (75 MHz, $CDCl_3$) δ 166.1, 156.8, 137.3, 134.4, 133.1, 130.1, 128.9, 128.7, 127.5, 126.9, 126.3, 125.0, 84.9, 66.2, 32.6.

IR (film): ν (cm^{-1}) 3250, 1769, 1720, 1599, 1485, 1464, 1450, 1275, 1229, 1179, 1109, 1038, 995, 959, 852, 744, 685, 558, 404.

HRMS (ESI, *m*/*z*) calcd for $C_{18}H_{17}NO_4Na$ [M+Na]$^+$: 334.1050, found: 334.1059.

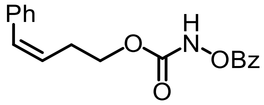

(*Z*)-4-Phenylbut-3-en-1-yl (benzoyloxy)carbamate (1b)

A white solid.

^{1}H NMR (300 MHz, $CDCl_3$) δ 8.32 (s 1H), 8.10-8.07 (m, 2H), 7.67-7.61 (m, 1H), 7.51-7.46 (m, 2H), 7.36-7.24 (m, 5H), 6.56 (d, J = 11.6 Hz, 1H), 5.65 (dt, J = 11.6 Hz, 7.2 Hz, 1H), 4.32 (t, J = 6.7 Hz, 2H), 2.76-2.69 (m, 2H).

^{13}C NMR (75 MHz, $CDCl_3$) δ 166.0, 156.7, 137.2, 134.4, 132.0, 130.2, 128.9, 128.8, 128.5, 127.1, 126.9, 126.8, 66.4, 28.3.

IR (film): ν (cm^{-1}) 3270, 1723, 1599, 1451, 1319, 1226, 1179, 1107, 1045, 1003, 962, 858, 795, 762, 696, 596, 520.

HRMS (ESI, *m*/*z*) calcd for $C_{18}H_{17}NO_4Na$ [M+Na]$^+$: 334.1050, found: 334.1057.

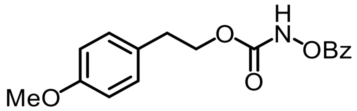

4-Methoxyphenethyl (*N*-benzoyloxy)carbamate (1c)

A white solid.

^{1}H NMR (300 MHz, $CDCl_3$) δ 8.36 (s, 1H), 8.10-8.07 (m, 2H), 7.67-7.62 (m, 1H), 7.52-7.47 (m, 2H), 7.13-7.09 (m, 2H), 6.82-6.78 (m, 2H), 4.39 (t, J = 7.0 Hz, 2H), 3.77 (s, 3H), 2.92 (t, J = 7.0 Hz, 2H).

^{13}C NMR (75 MHz, $CDCl_3$) δ 166.0, 158.6, 156.7, 134.4, 130.14, 130.06, 129.4, 128.9, 126.9, 114.2, 67.6, 55.4, 34.4.

IR (film): ν (cm^{-1}) 3223, 2960, 1766, 1718, 1513, 1467, 1451, 1248, 1233, 1177, 1112, 1027, 1007, 965, 833, 819, 765, 739, 703, 686, 519, 401.

HRMS (ESI, *m*/*z*) calcd for $C_{17}H_{17}NO_5Na$ $[M+Na]^+$: 338.0999, found: 338.1007.

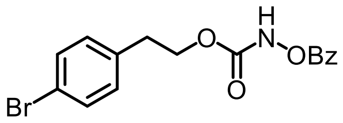

4-Bromophenethyl (*N*-benzoyloxy)carbamate (1d)

A white solid.

^{1}H NMR (300 MHz, $CDCl_3$) δ 8.24 (s, 1H), 8.08-8.05 (m, 2H), 7.69-7.63 (m, 1H), 7.53-7.48 (m, 2H), 7.36 (d, J = 8.3 Hz, 2H), 7.07 (d, J = 8.3 Hz, 2H), 4.41 (t, J = 6.8 Hz, 2H), 2.94 (t, J = 6.8 Hz, 2H).

^{13}C NMR (75 MHz, $CDCl_3$) δ 166.0, 156.6, 136.4, 134.5, 131.8, 130.8, 130.2, 128.9, 126.8, 120.8, 66.9, 34.8.

IR (film): ν (cm^{-1}) 3309, 1746, 1708, 1598, 1486, 1450, 1414, 1320, 1244, 1093, 1067, 1049, 1010, 836, 813, 702, 608, 526, 435.

HRMS (ESI, *m*/*z*) calcd for $C_{16}H_{14}BrNO_4Na$ $[M+Na]^+$: 385.9998, found: 386.0008.

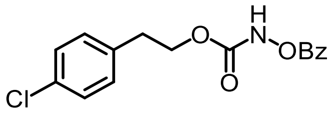

4-Chlorophenethyl (*N*-benzoyloxy)carbamate (1e)

A white solid.

^{1}H NMR (300 MHz, $CDCl_3$) δ 8.33 (s, 1H), 8.08-8.05 (m, 2H), 7.68-7.62 (m, 1H), 7.52-7.47 (m, 2H), 7.21 (d, J = 8.4 Hz, 2H), 7.12 (d, J = 8.4 Hz, 2H), 4.40 (t, J = 6.8 Hz, 2H), 2.95 (t, J = 6.8 Hz, 2H).

^{13}C NMR (75 MHz, $CDCl_3$) δ 166.0, 156.6, 135.9, 134.5, 132.7, 130.4, 130.1, 128.9, 128.8, 126.8, 66.9, 34.6.

IR (film): ν (cm^{-1}) 3307, 1746, 1710, 1491, 1451, 1408, 1320, 1243, 1092, 1067, 1012, 815, 702, 539, 423, 386.

HRMS (ESI, *m*/*z*) calcd for $C_{16}H_{14}ClNO_4Na$ $[M+Na]^+$: 342.0504, found: 342.0513.

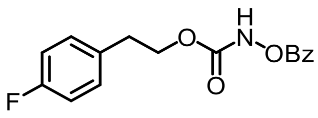

4-Fluorophenethyl (*N*-benzoyloxy)carbamate (1f)

A white solid.

^{1}H NMR (300 MHz, $CDCl_3$) δ 8.32 (s, 1H), 8.08-8.06 (m, 2H), 7.68-7.62 (m, 1H), 7.52-7.47 (m, 2H), 7.17-7.12 (m, 2H), 6.96-6.89 (m, 2H), 4.40 (t, J = 6.9 Hz, 2H), 2.95 (t, J = 6.9 Hz, 2H).

^{13}C NMR (75 MHz, $CDCl_3$) δ 166.0, 161.9 (d, J = 244.4 Hz), 156.7, 134.5, 133.1 (d, J = 3.3 Hz), 130.5 (d, J = 8.0 Hz), 130.1, 128.9, 126.8, 115.5 (d, J = 21.2 Hz), 67.2, 34.5.

^{19}F NMR (235 MHz, $CDCl_3$) δ -116.4.

IR (film): ν (cm^{-1}) 3308, 1746, 1710, 1599, 1507, 1451, 1414, 1321, 1244, 1220, 1093, 1068, 1031, 819, 701, 553, 478, 419.

HRMS (ESI, *m*/*z*) calcd for $C_{16}H_{14}FNO_4Na$ [M+Na]$^+$: 326.0799, found: 326.0807.

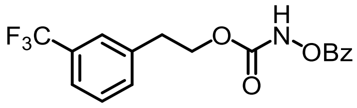

3-(Trifluoromethyl)phenethyl (*N*-benzoyloxy)carbamate (1g)

A white solid.

^{1}H NMR (300 MHz, $CDCl_3$) δ 8.28 (s, 1H), 8.09-8.06 (m, 2H), 7.67-7.62 (m, 1H), 7.52-7.47 (m, 4H), 7.41-7.35 (m, 2H), 4.46 (t, J = 6.8 Hz, 2H), 3.05 (t, J = 6.8 Hz, 2H).

^{13}C NMR (75 MHz, $CDCl_3$) δ 166.0, 156.5, 138.4, 134.5, 132.5, 131.1 (d, J = 32.2 Hz), 130.1, 129.2, 128.1, 126.8, 125.8 (q, J = 3.8 Hz), 123.8 (q, J = 3.8 Hz), 124.3 (d, J = 272.1 Hz), 66.7, 35.1.

^{19}F NMR (235 MHz, $CDCl_3$) δ -62.6.

IR (film): ν (cm^{-1}) 3230, 1764, 1722, 1601, 1475, 1450, 1322, 1281, 1234, 1165, 1113, 1070, 1044, 976, 850, 796, 751, 701, 686, 608.

HRMS (ESI, *m*/*z*) calcd for $C_{17}H_{14}F_3NO_4Na$ [M+Na]$^+$: 376.0767, found: 376.0777.

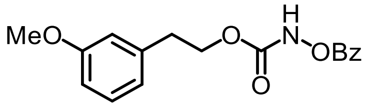

3-Methoxyphenethyl (*N*-benzoyloxy)carbamate (1h)

A white solid.

^{1}H NMR (300 MHz, $CDCl_3$) δ 8.33 (s, 1H), 8.10-8.07 (m, 2H), 7.67-7.62 (m, 1H), 7.52-7.46 (m, 2H), 7.21-7.16 (m, 1H), 6.81-6.76 (m, 3H), 4.43 (t, J = 7.0 Hz, 2H), 3.79 (s, 3H), 2.97 (t, J = 7.0 Hz, 2H).

^{13}C NMR (75 MHz, $CDCl_3$) δ 166.0, 159.9, 156.7, 138.9, 134.4, 130.2, 129.7, 128.9, 126.9, 121.4, 114.8, 112.3, 67.3, 55.3, 35.4.

IR (film): ν (cm^{-1}) 3307, 1745, 1707, 1413, 1402, 1318, 1244, 1066, 1010, 983, 812, 744, 718, 526, 487, 466, 437.

HRMS (ESI, m/z) calcd for $C_{17}H_{17}NO_5Na$ [M+Na]$^+$: 338.0999, found: 338.1007.

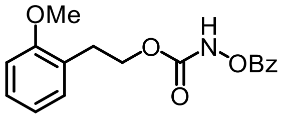

2-Methoxyphenethyl (*N*-benzoyloxy)carbamate (1i)

A white solid.

^{1}H NMR (300 MHz, $CDCl_3$) δ 8.29 (s, 1H), 8.10-8.07 (m, 2H), 7.67-7.62 (m, 1H), 7.52-7.46 (m, 2H), 7.24-7.12 (m, 2H), 6.86-6.82 (m, 2H), 4.42 (t, J = 7.0 Hz, 2H), 3.82 (s, 3H), 3.01 (t, J = 7.0 Hz, 2H).

^{13}C NMR (75 MHz, $CDCl_3$) δ 166.0, 157.8, 156.8, 134.4, 131.0, 130.2, 128.9, 128.2, 127.0, 125.6, 120.6, 110.5, 66.4, 55.4, 30.3.

IR (film): ν (cm^{-1}) 3207, 1772, 1718, 1601, 1493, 1460, 1438, 1288, 1236, 1104, 1080, 1034, 963, 854, 768, 751, 703, 684, 613, 573, 525, 473.

HRMS (ESI, m/z) calcd for $C_{17}H_{17}NO_5Na$ [M+Na]$^+$: 338.0999, found: 338.1008.

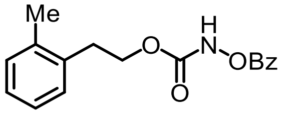

2-Methylphenethyl (*N*-benzoyloxy)carbamate (1j)

A white solid.

^{1}H NMR (300 MHz, $CDCl_3$) δ 8.33 (s, 1H), 8.12-8.08 (m, 2H), 7.68-7.62 (m, 1H), 7.52-7.47 (m, 2H), 7.16-7.08 (m, 4H), 4.40 (t, J = 7.4 Hz, 2H), 3.01 (t, J = 7.4 Hz, 2H), 2.34 (s, 3H).

^{13}C NMR (75 MHz, $CDCl_3$) δ 166.0, 156.7, 136.6, 135.3, 134.4, 130.6, 130.2, 129.8, 128.9, 127.1, 126.9, 126.3, 66.5, 32.6, 19.5.

IR (film): ν (cm^{-1}) 3223, 1759, 1688, 1415, 1346, 1235, 1178, 1095, 1037, 999, 856, 747, 707, 617, 584, 449, 423.

HRMS (ESI, m/z) calcd for $C_{17}H_{17}NO_4Na$ [M+Na]$^+$: 322.1050, found: 322.1058.

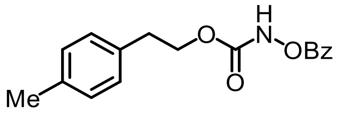

4-Methylphenethyl (*N*-benzoyloxy)carbamate (1k)

A white solid.

^{1}H NMR (300 MHz, $CDCl_3$) δ 8.30 (s, 1H), 8.10-8.07 (m, 2H), 7.68-7.62 (m, 1H), 7.52-7.47 (m, 2H), 7.12-7.06 (m, 4H), 4.41 (t, J = 7.0 Hz, 2H), 2.95 (t, J = 7.0 Hz, 2H), 2.31 (s, 3H).

^{13}C NMR (75 MHz, $CDCl_3$) δ 166.0, 156.7, 136.4, 134.4, 134.2, 130.2, 129.0, 128.9, 126.9, 67.5, 34.9, 21.2.

IR (film): ν (cm^{-1}) 3197, 1738, 1463, 1230, 1119, 1013, 966, 816, 749, 707, 688, 528, 490.

HRMS (ESI, *m*/*z*) calcd for $C_{17}H_{17}NO_4Na$ [M+Na]$^+$: 322.1050, found: 322.1058.

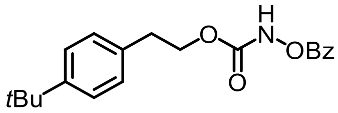

4-(*tert*-Butyl)phenethyl (*N*-benzoyloxy)carbamate (1l)

A white solid.

^{1}H NMR (300 MHz, $CDCl_3$) δ 8.32 (s, 1H), 8.11-8.09 (m, 2H), 7.68-7.63 (m, 1H), 7.52-7.47 (m, 2H), 7.30-7.28 (m, 2H), 7.16-7.13 (m, 2H), 4.43 (t, J = 7.1 Hz, 2H), 2.96 (t, J = 7.1 Hz, 2H), 1.30 (s, 9H).

^{13}C NMR (75 MHz, $CDCl_3$) δ 166.0, 156.7, 149.7, 134.4, 134.3, 130.2, 128.9, 128.8, 126.9, 125.6, 67.5, 34.8, 34.6, 31.5.

IR (film): ν (cm^{-1}) 3347, 2959, 1749, 1600, 1508, 1474, 1450, 1415, 1327, 1240, 1180, 1093, 1062, 1050, 1010, 836, 816, 757, 697, 573, 497.

HRMS (ESI, *m*/*z*) calcd for $C_{20}H_{23}NO_4Na$ [M+Na]$^+$: 364.1519, found: 364.1528.

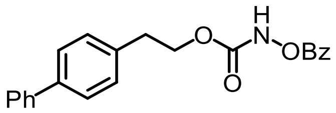

2-([1,1'-Biphenyl]-4-yl)ethyl (*N*-benzoyloxy)carbamate (1m)

A white solid.

^{1}H NMR (300 MHz, $CDCl_3$) δ 8.30 (s, 1H), 8.10-8.07 (m, 2H), 7.66-7.61 (m, 1H), 7.58-7.55 (m, 2H), 7.51-7.41 (m, 6H), 7.36-7.27 (m, 3H), 4.48 (t, J = 6.9 Hz, 2H), 3.04 (t, J = 6.9 Hz, 2H).

^{13}C NMR (75 MHz, $CDCl_3$) δ 166.0, 156.7, 141.0, 139.9, 136.4, 134.4, 130.2, 129.5, 128.9, 127.5,

127.4, 127.2, 126.9, 67.3, 35.0.

IR (film): *v* (cm^{-1}) 3416, 3398, 3380, 3366, 3271, 3226, 2921, 2894, 2850, 1990, 1608, 1589, 1460, 1431, 1375, 1118, 1140, 1025, 654, 618, 584, 541, 503, 402.

HRMS (ESI, *m*/*z*) calcd for $C_{22}H_{19}NO_4Na$ $[M+Na]^+$: 384.1206, found: 384.1216.

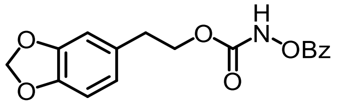

2-(Benzo[d][1,3]dioxol-5-yl)ethyl (*N*-benzoyloxy)carbamate (1n)

A white solid.

^{1}H NMR (300 MHz, $CDCl_3$) δ 8.30 (s, 1H), 8.10-8.07 (m, 2H), 7.67-7.62 (m, 1H), 7.52-7.47 (m, 2H), 6.71-6.63 (m, 3H), 5.92 (s, 2H), 4.38 (t, *J* = 6.9 Hz, 2H), 2.90 (t, *J* = 6.9 Hz, 2H).

^{13}C NMR (75 MHz, $CDCl_3$) δ 166.0, 156.7, 147.9, 146.5, 134.4, 131.1, 130.2, 128.9, 126.9, 122.1, 109.5, 108.5, 101.1, 67.5, 35.1.

IR (film): *v* (cm^{-1}) 3214, 2904, 1768, 1721, 1502, 1486, 1450, 1438, 1285, 1237, 1194, 1108, 1079, 1030, 1006, 961, 919, 857, 815, 768, 751, 700, 684, 604, 427.

HRMS (ESI, *m*/*z*) calcd for $C_{17}H_{15}NO_6Na$ $[M+Na]^+$: 352.0792, found: 352.0801.

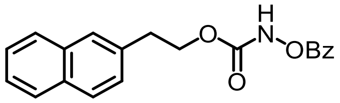

2-(Naphthalen-2-yl)ethyl (*N*-benzoyloxy)carbamate (1o)

A white solid.

^{1}H NMR (300 MHz, $CDCl_3$) δ 8.30 (s, 1H), 8.07-8.04 (m, 2H), 7.81-7.74 (m, 3H), 7.66-7.61 (m, 2H), 7.49-7.42 (m, 4H), 7.34 (dd, *J* = 8.4 Hz, 1.4 Hz, 1H), 4.53 (t, *J* = 7.0 Hz, 2H), 3.16 (t, *J* = 6.9 Hz, 2H).

^{13}C NMR (75 MHz, $CDCl_3$) δ 166.0, 156.7, 134.8, 134.4, 133.7, 132.5, 130.1, 128.9, 128.4, 127.8, 127.7, 127.6, 127.4, 126.8, 126.3, 125.7, 67.3, 35.5.

IR (film): *v* (cm^{-1}) 3217, 2184, 1768, 1711, 1696, 1420, 1334, 1233, 1104, 1066, 1045, 1006, 996, 816, 737, 696, 552, 479, 464, 396.

HRMS (ESI, *m*/*z*) calcd for $C_{20}H_{17}NO_4Na$ $[M+Na]^+$: 358.1050, found: 358.1059.

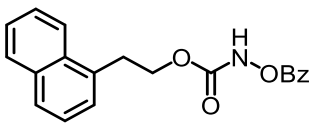

2-(Naphthalen-1-yl)ethyl (*N*-benzoyloxy)carbamate (1p)

A white solid.

^{1}H NMR (300 MHz, $CDCl_3$) δ 8.36 (s, 1H), 8.11-8.07 (m, 3H), 7.88-7.85 (m, 1H), 7.78-7.73 (m, 1H), 7.68-7.62 (m, 1H), 7.57-7.46 (m, 4H), 7.40-7.35 (m, 2H), 4.57 (t, *J* = 7.4 Hz, 2H), 3.48 (t, *J* = 7.4 Hz, 2H).

^{13}C NMR (75 MHz, $CDCl_3$) δ 166.0, 156.7, 134.4, 134.1, 133.1, 132.2, 130.2, 129.0, 128.9, 127.8, 127.4, 126.9, 126.5, 125.9, 125.6, 123.6, 66.8, 32.5.

IR (film): ν (cm^{-1}) 3204, 1769, 1725, 1600, 1488, 1467, 1452, 1238, 1111, 1040, 966, 816, 734, 712, 689, 652, 603, 587, 427.

HRMS (ESI, *m*/*z*) calcd for $C_{20}H_{17}NO_4Na$ [M+Na]$^+$: 358.1050, found: 358.1059.

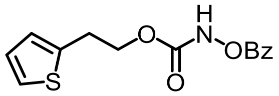

2-(Thiophen-2-yl)ethyl (*N*-benzoyloxy)carbamate (1q)

A white solid.

^{1}H NMR (300 MHz, $CDCl_3$) δ 8.40 (s, 1H), 8.11-8.08 (m, 2H), 7.67-7.62 (m, 1H), 7.52-7.46 (m, 2H), 7.14 (dd, *J* = 5.1 Hz, 1.1 Hz, 1H), 6.93-6.90 (m, 1H), 6.87-6.85 (m, 1H), 4.44 (t, *J* = 6.7 Hz, 2H), 3.21 (t, *J* = 6.7 Hz, 2H).

^{13}C NMR (75 MHz, $CDCl_3$) δ 166.0, 156.5, 139.3, 134.4, 130.2, 128.9, 127.1, 126.9, 125.9, 124.3, 67.0, 29.5.

IR (film): ν (cm^{-1}) 3227, 1773, 1716, 1599, 1492, 1466, 1451, 1237, 1116, 1040, 1002, 964, 852, 765, 701, 494.

HRMS (ESI, *m*/*z*) calcd for $C_{14}H_{13}NO_4SNa$ [M+Na]$^+$: 314.0457, found: 314.0465.

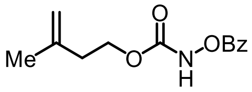

3-Methylbut-3-en-1-yl (*N*-benzoyloxy)carbamate (1r)

A colorless oil.

^{1}H NMR (300 MHz, $CDCl_3$) δ 8.33 (s, 1H), 8.10-8.08 (m, 2H), 7.66-7.61 (m, 1H), 7.51-7.46 (m, 2H), 4.77 (d, J = 17.0 Hz, 2H), 4.34 (t, J = 6.8 Hz, 2H), 2.39 (t, J = 6.8 Hz, 2H), 1.75 (s, 3H).

^{13}C NMR (75 MHz, $CDCl_3$) δ 166.1, 156.8, 141.2, 134.4, 130.1, 128.9, 126.9, 112.9, 65.2, 36.9, 22.6.

IR (film): ν (cm^{-1}) 3267, 2968, 1734, 1601, 1452, 1332, 1227, 1179, 1108, 1046, 1004, 893, 704, 685, 550.

HRMS (ESI, *m/z*) calcd for $C_{13}H_{15}NO_4Na$ $[M+Na]^+$: 272.0893, found: 272.0901.

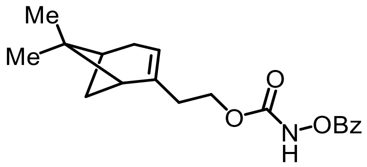

2-(6,6-Dimethylbicyclo[3.1.1]hept-2-en-2-yl)ethyl (*N*-benzoyloxy)carbamate (1s)

A colorless oil.

^{1}H NMR (300 MHz, $CDCl_3$) δ 8.30 (s 1H), 8.11-8.08 (m, 2H), 7.66-7.61 (m, 1H), 7.51-7.46 (m, 2H), 5.29-5.28 (m, 1H), 4.30-4.16 (m, 2H), 2.38-2.01 (m, 7H), 1.25 (s, 3H), 1.13 (d, J = 8.6 Hz, 1H), 0.81 (s, 3H).

^{13}C NMR (75 MHz, $CDCl_3$) δ 166.1, 156.8, 143.7, 134.4, 130.1, 128.9, 126.9, 119.4, 65.2, 45.8, 40.9, 38.2, 36.2, 31.8, 31.5, 26.4, 21.3.

IR (film): ν (cm^{-1}) 3278, 2913, 1737, 1601, 1452, 1329, 1228, 1179, 1106, 1004, 856, 704, 686, 572.

HRMS (ESI, *m/z*) calcd for $C_{19}H_{23}NO_4Na$ $[M+Na]^+$: 352.1519, found: 352.1528.

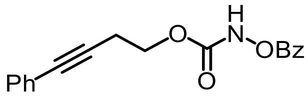

4-Phenylbut-3-yn-1-yl (*N*-benzoyloxy)carbamate (1t)

A white solid.

^{1}H NMR (300 MHz, $CDCl_3$) δ 8.34 (s 1H), 8.11-8.08 (m, 2H), 7.66-7.60 (m, 1H), 7.49-7.44 (m, 2H), 7.41-7.35 (m, 2H), 7.31-7.25 (m, 3H), 4.41 (t, J = 6.9 Hz, 2H), 2.81 (t, J = 6.9 Hz, 2H).

^{13}C NMR (75 MHz, $CDCl_3$) δ 166.0, 156.4, 134.4, 131.9, 130.2, 128.9, 128.4, 128.2, 126.8, 123.4, 84.9, 82.5, 64.7, 20.3.

IR (film): ν (cm^{-1}) 3226, 1773, 1720, 1599, 1488, 1451, 1234, 1180, 1116, 1048, 998, 952, 852, 752, 700, 688, 592, 527, 451.

HRMS (ESI, *m*/*z*) calcd for $C_{18}H_{15}NO_4Na$ $[M+Na]^+$: 332.0893, found: 332.0901.

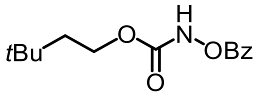

3,3-Dimethylbutyl (*N*-benzoyloxy)carbamate (1u)

A white solid.

^{1}H NMR (300 MHz, $CDCl_3$) δ 8.32 (s 1H), 8.11-8.08 (m, 2H), 7.66-7.61 (m, 1H), 7.51-7.46 (m, 2H), 4.29 (t, *J* = 7.4 Hz, 2H), 1.61 (t, *J* = 7.4 Hz, 2H), 0.94 (s, 9H).

^{13}C NMR (75 MHz, $CDCl_3$) δ 166.1, 156.9, 134.4, 130.1, 128.9, 127.0, 65.0, 41.9, 29.9, 29.7.

IR (film): ν (cm^{-1}) 3275, 2957, 1736, 1601, 1452, 1366, 1228, 1179, 1107, 1045, 1005, 856, 705, 686, 574.

HRMS (ESI, *m*/*z*) calcd for $C_{14}H_{19}NO_4Na$ $[M+Na]^+$: 288.1206, found: 288.1214.

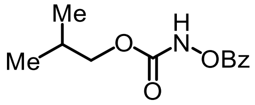

Isobutyl (*N*-benzoyloxy)carbamate (1v)

A white solid.

^{1}H NMR (300 MHz, $CDCl_3$) δ 8.36 (s 1H), 8.11-8.08 (m, 2H), 7.66-7.61 (m, 1H), 7.51-7.46 (m, 2H), 4.01 (d, *J* = 6.6 Hz, 2H), 2.04-1.91 (m, 1H), 0.93 (d, *J* = 6.7 Hz, 6H).

^{13}C NMR (75 MHz, $CDCl_3$) δ 166.1, 157.0, 134.3, 130.1, 128.9, 127.0, 72.9, 28.0, 19.0.

IR (film): ν (cm^{-1}) 3244, 2962, 1761, 1723, 1601, 1469, 1452, 1231, 1177, 1108, 1040, 1003, 969, 853, 750, 703, 685, 588.

HRMS (ESI, *m*/*z*) calcd for $C_{12}H_{15}NO_4Na$ $[M+Na]^+$: 260.0893, found: 260.0901.

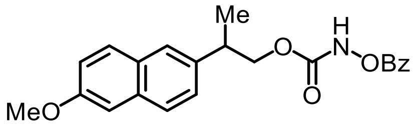

2-(6-Methoxynaphthalen-2-yl)propyl (*N*-benzoyloxy)carbamate (1w)

A white solid.

^{1}H NMR (300 MHz, $CDCl_3$) δ 8.25 (s 1H), 8.04-8.01 (m, 2H), 7.67-7.57 (m, 4H), 7.47-7.42 (m, 2H), 7.31 (dd, *J* = 8.5 Hz, 1.7 Hz, 1H), 7.14-7.09 (m, 2H), 4.44-4.34 (m, 2H), 3.92 (s, 3H), 3.34-3.22 (m,

1H), 1.39 (d, J = 7.0 Hz, 3H).

^{13}C NMR (75 MHz, $CDCl_3$) δ 166.0, 157.7, 156.8, 137.9, 134.3, 133.7, 130.1, 129.3, 129.2, 128.9, 127.2, 126.8, 126.3, 125.8, 119.0, 105.8, 71.6, 55.5, 39.1, 18.1.

IR (film): ν (cm^{-1}) 3263, 2964, 1765, 1728, 1608, 1504, 1478, 1450, 1395, 1266, 1230, 1216, 1137, 1091, 1033, 953, 851, 809, 749, 710, 686, 580, 474.

HRMS (ESI, m/z) calcd for $C_{22}H_{21}NO_5Na$ $[M+Na]^+$: 402.1312, found: 402.1322.

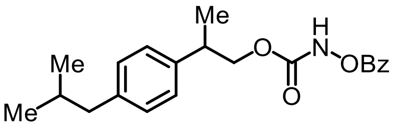

2-(4-Isobutylphenyl)propyl (*N*-benzoyloxy)carbamate (1x)

A colorless oil.

^{1}H NMR (300 MHz, $CDCl_3$) δ 8.30 (s 1H), 8.09-8.06 (m, 2H), 7.67-7.62 (m, 1H), 7.52-7.46 (m, 2H), 7.11 (d, J = 8.1 Hz, 2H), 7.05 (d, J = 8.1 Hz, 2H), 4.35-4.23 (m, 2H), 3.17-3.05 (m, 1H), 2.43 (d, J = 7.2 Hz, 2H), 1.90-1.77 (m, 1H), 1.30 (d, J = 7.0 Hz, 3H), 0.89 (d, J = 6.6 Hz, 6H).

^{13}C NMR (75 MHz, $CDCl_3$) δ 166.0, 156.8, 140.3, 139.9, 134.4, 130.1, 129.4, 128.9, 127.2, 126.9, 71.9, 45.2, 38.8, 30.3, 22.6, 18.0.

IR (film): ν (cm^{-1}) 3270, 2955, 1738, 1601, 1452, 1332, 1226, 1179, 1105, 1044, 1004, 960, 846, 798, 704, 686, 543.

HRMS (ESI, m/z) calcd for $C_{21}H_{25}NO_4Na$ $[M+Na]^+$: 378.1676, found: 378.1686.

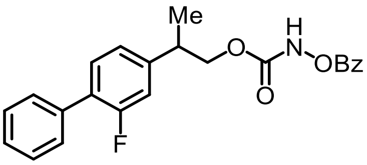

2-(2-Fluoro-[1,1'-biphenyl]-4-yl)propyl (*N*-benzoyloxy)carbamate (1y)

A colorless oil.

^{1}H NMR (300 MHz, $CDCl_3$) δ 8.29 (s 1H), 8.08-8.05 (m, 2H), 7.65-7.60 (m, 1H), 7.53-7.30 (m, 8H), 7.08-7.00 (m, 2H), 4.34 (d, J = 7.0 Hz, 2H), 3.25-3.13 (m, 1H), 1.34 (d, J = 7.0 Hz, 3H).

^{13}C NMR (75 MHz, $CDCl_3$) δ 166.0, 159.5 (d, J = 248.0 Hz), 156.6, 144.4 (d, J = 7.3 Hz), 135.8, 134.4, 130.94, 130.89, 130.1, 129.15, 129.11, 128.9, 128.6, 127.8, 127.7, 127.5, 126.8, 123.5 (d, J = 3.3 Hz), 115.1 (d, J = 23.2 Hz), 71.2, 38.8, 17.8.

^{19}F NMR (235 MHz, $CDCl_3$) δ -117.8.

IR (film): ν (cm^{-1}) 3271, 2970, 1738, 1601, 1582, 1484, 1451, 1417, 1331, 1225, 1106, 1044, 1003, 963, 830, 767, 698, 576.

HRMS (ESI, *m*/*z*) calcd for $C_{23}H_{20}FNO_4Na$ $[M+Na]^+$: 416.1269, found: 416.1279.

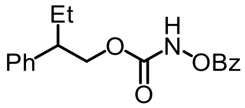

2-Phenylbutyl (*N*-benzoyloxy)carbamate (1z)

A colorless oil.

^{1}H NMR (300 MHz, $CDCl_3$) δ 8.31 (s 1H), 8.07-8.04 (m, 2H), 7.66-7.62 (m, 1H), 7.51-7.46 (m, 2H), 7.29-7.15 (m, 5H), 4.43-4.29 (m, 2H), 2.91-2.82 (m, 1H), 1.87-1.73 (m, 1H), 1.69-1.54 (m, 1H), 0.83 (t, J = 7.4 Hz, 3H).

^{13}C NMR (75 MHz, $CDCl_3$) δ 165.9, 156.8, 141.4, 134.3, 130.1, 128.8, 128.6, 128.0, 126.92, 126.89, 70.5, 46.9, 25.3, 11.9.

IR (film): ν (cm^{-1}) 3270, 2963, 1736, 1601, 1452, 1320, 1225, 1179, 1106, 1045, 1004, 855, 760, 699, 558.

HRMS (ESI, *m*/*z*) calcd for $C_{18}H_{19}NO_4Na$ $[M+Na]^+$: 336.1206, found: 336.1215.

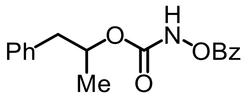

1-Phenylpropan-2-yl (*N*-benzoyloxy)carbamate (1za)

A colorless oil. The *N*-hydroxycarbamate was synthesized at 60 °C.

^{1}H NMR (300 MHz, $CDCl_3$) δ 8.28 (s 1H), 8.11-8.08 (m, 2H), 7.67-7.62 (m, 1H), 7.52-7.47 (m, 2H), 7.29-7.18 (m, 5H), 5.21-5.10 (m, 1H), 3.01 (dd, J = 13.7 Hz, 6.2 Hz, 1H), 2.83 (dd, J = 13.7 Hz, 6.7 Hz, 1H), 1.29 (d, J = 6.3 Hz, 3H).

^{13}C NMR (75 MHz, $CDCl_3$) δ 166.0, 156.4, 137.0, 134.4, 130.1, 129.7, 128.9, 128.6, 127.0, 126.8, 74.9, 42.3, 19.5.

IR (film): ν (cm^{-1}) 3263, 1732, 1601, 1452, 1381, 1319, 1230, 1179, 1105, 1037, 1018, 996, 858, 746, 699, 504.

HRMS (ESI, *m*/*z*) calcd for $C_{17}H_{17}NO_4Na$ $[M+Na]^+$: 322.1050, found: 322.1059.

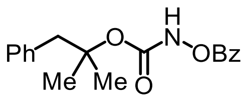

2-Methyl-1-phenylpropan-2-yl (*N*-benzoyloxy)carbamate (1zb)

A white solid. The *N*-hydroxycarbamate was synthesized at 60 °C.

^{1}H NMR (300 MHz, $CDCl_3$) δ 8.20 (s 1H), 8.13-8.10 (m, 2H), 7.67-7.61 (m, 1H), 7.52-7.47 (m, 2H), 7.29-7.18 (m, 5H), 3.12 (s, 2H), 1.51 (s, 6H).

^{13}C NMR (75 MHz, $CDCl_3$) δ 166.2, 155.7, 136.9, 134.3, 130.8, 130.1, 128.9, 128.2, 127.1, 126.8, 85.3, 46.7, 26.2.

IR (film): ν (cm^{-1}) 3191, 2980, 1768, 1708, 1600, 1476, 1451, 1285, 1242, 1213, 1179, 1106, 995, 977, 866, 736, 701, 682, 611, 504, 401.

HRMS (ESI, *m*/*z*) calcd for $C_{18}H_{19}NO_4Na$ $[M+Na]^+$: 336.1206, found: 336.1216.

4. Experimental and Characterization Data of Products

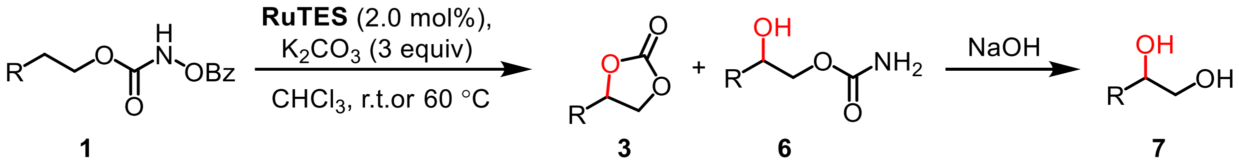

Typical procedure for the intramolecular C(sp^3)-H oxygenation. A dried 10 mL Schlenk flask was charged with substrate **1** (0.2 mmol), K_2CO_3 (0.6 mmol, 82.9 mg) and *rac*-**RuTES** (0.004 mmol, 4.9 mg, 2 mol%). The flask was purged with nitrogen. Then, $CHCl_3$ (4 mL, 0.05 M) was added via syringe and the flask was sealed and stirred at room temperature or 60 °C for 16 hours. The mixture was washed with water to remove the excess base and then hydrolyzed with 1 N HCl (0.2 mL, 1.0 equiv) for 15 min. Thereafter, the mixture was extracted with CH_2Cl_2 for 5 times and the solvent evaporated. The crude product was purified by flash chromatography on silica gel (*n*-hexane/EtOAc) to afford the product carbonate **3** or hydroxylated carbamate **6**.

Additional basic hydrolysis. The crude product was redissolved in dioxane (4 mL) under nitrogen atmosphere, and 1 N NaOH (4 mL) was added. The mixture was stirred at 80 °C for 2 hours. After cooling to room temperature, water (4 mL) was added and extracted with EtOAc for 5 times. Then, the solvent was evaporated and the crude product was purified by flash chromatography on silica gel (*n*-hexane/EtOAc or CH_2Cl_2/EtOAc) to afford the diol product **7**.

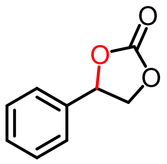

4-Phenyl-1,3-dioxolan-2-one (3a)

According to the typical procedure, a mixture of **1ab** (57.1 mg, 0.2 mmol), K_2CO_3 (82.9 mg, 0.6 mmol) and **RuTES** (4.9 mg, 2 mol%) in $CHCl_3$ (4 mL, 0.05 M) under nitrogen atmosphere at room temperature stirred for 16 hours, afforded **3a** (27.8 mg, 85% yield) as a white solid. Purification conditions: *n*-Hex/EtOAc = 3:1. The analytical data are in accordance with the literature.[7]

^{1}H NMR (300 MHz, $CDCl_3$) δ 7.48-7.42 (m, 3H), 7.40-7.35 (m, 2H), 5.68 (t, J = 8.0 Hz, 1H), 4.80 (t, J = 8.4 Hz, 1H), 4.35 (t, J = 8.2 Hz, 1H).

^{13}C NMR (75 MHz, $CDCl_3$) δ 154.9, 136.0, 129.9, 129.4, 126.0, 78.1, 71.3.

HRMS (ESI, *m/z*) calcd for $C_9H_8O_3Na$ $[M+Na]^+$: 187.0366, found: 187.0370.

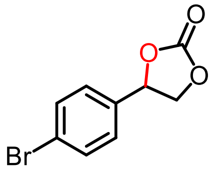

4-(4-Bromophenyl)-1,3-dioxolan-2-one (3d)

According to the typical procedure, a mixture of **1d** (72.8 mg, 0.2 mmol), K_2CO_3 (82.9 mg, 0.6 mmol) and **RuTES** (4.9 mg, 2 mol%) in $CHCl_3$ (4 mL, 0.05 M) under nitrogen atmosphere at room temperature stirred for 16 hours, afforded **3d** (42.8 mg, 88% yield) as a white solid. Purification conditions: *n*-Hex/EtOAc = 3:1. The analytical data are in accordance with the literature.[7]

^{1}H NMR (300 MHz, $CDCl_3$) δ 7.60-7.57 (m, 2H), 7.26-7.23 (m, 2H), 5.64 (t, J = 8.0 Hz, 1H), 4.80 (t, J = 8.4 Hz, 1H), 4.30 (t, J = 8.3 Hz, 1H).

^{13}C NMR (75 MHz, $CDCl_3$) δ 154.6, 135.0, 132.7, 127.6, 124.1, 77.4, 71.1.

HRMS (ESI, *m/z*) calcd for $C_9H_7BrNO_3Na$ $[M+Na]^+$: 264.9471, found: 264.9479.

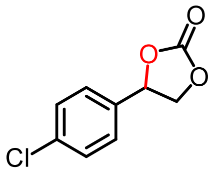

4-(4-Chlorophenyl)-1,3-dioxolan-2-one (3e)

According to the typical procedure, a mixture of **1e** (63.9 mg, 0.2 mmol), K_2CO_3 (82.9 mg, 0.6 mmol) and **RuTES** (4.9 mg, 2 mol%) in $CHCl_3$ (4 mL, 0.05 M) under nitrogen atmosphere at room temperature stirred for 16 hours, afforded **3e** (34.6 mg, 87% yield) as a white solid. Purification conditions: *n*-Hex/EtOAc = 3:1. The analytical data are in accordance with the literature.[7]

^{1}H NMR (300 MHz, $CDCl_3$) δ 7.44-7.41 (m, 2H), 7.32-7.29 (m, 2H), 5.65 (t, J = 8.0 Hz, 1H), 4.80 (t, J = 8.4 Hz, 1H), 4.30 (dd, J = 8.4 Hz, 8.0 Hz, 1H).

^{13}C NMR (75 MHz, $CDCl_3$) δ 154.6, 136.0, 134.5, 129.7, 127.4, 77.4, 71.1.

HRMS (ESI, *m/z*) calcd for $C_9H_7ClO_3Na$ $[M+Na]^+$: 220.9976, found: 220.9982.

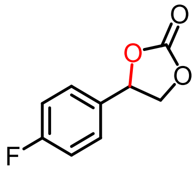

4-(4-Fluorophenyl)-1,3-dioxolan-2-one (3f)

According to the typical procedure, a mixture of **1f** (60.7 mg, 0.2 mmol), K_2CO_3 (82.9 mg, 0.6 mmol) and **RuTES** (4.9 mg, 2 mol%) in $CHCl_3$ (4 mL, 0.05 M) under nitrogen atmosphere at room temperature stirred for 16 hours, afforded **3f** (30.8 mg, 85% yield) as a white solid. Purification conditions: *n*-Hex/EtOAc = 3:1. The analytical data are in accordance with the literature.[7]

^{1}H NMR (300 MHz, $CDCl_3$) δ 7.39-7.34 (m, 2H), 7.17-7.11 (m, 2H), 5.66 (t, J = 8.0 Hz, 1H), 4.79 (t, J = 8.4 Hz, 1H), 4.32 (t, J = 8.3 Hz, 1H).

^{13}C NMR (75 MHz, $CDCl_3$) δ 163.6 (d, J = 248.1 Hz), 154.7, 131.8 (d, J = 3.3 Hz), 128.2 (d, J = 8.5 Hz), 116.6 (d, J = 22.0 Hz), 77.6, 71.2.

^{19}F NMR (235 MHz, $CDCl_3$) δ -110.9.

HRMS (ESI, *m*/*z*) calcd for $C_9H_7FO_3Na$ $[M+Na]^+$: 205.0271, found: 205.0277.

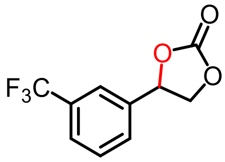

4-(3-(Trifluoromethyl)phenyl)-1,3-dioxolan-2-one (3g)

According to the typical procedure, a mixture of **1g** (70.6 mg, 0.2 mmol), K_2CO_3 (82.9 mg, 0.6 mmol) and **RuTES** (4.9 mg, 2 mol%) in $CHCl_3$ (4 mL, 0.05 M) under nitrogen atmosphere at room temperature stirred for 16 hours, afforded **3g** (32.6 mg, 70% yield) as a pale yellow oil. Purification conditions: *n*-Hex/EtOAc = 3:1. The analytical data are in accordance with the literature.[7]

^{1}H NMR (300 MHz, $CDCl_3$) δ 7.71-7.69 (m, 1H), 7.62-7.56 (m, 3H), 5.75 (t, J = 8.0 Hz, 1H), 4.86 (t, J = 8.4 Hz, 1H), 4.34 (t, J = 8.2 Hz, 1H).

^{13}C NMR (75 MHz, $CDCl_3$) δ 154.5, 137.2, 132.0 (q, J = 32.8 Hz), 130.2, 129.2, 126.7 (q, J = 3.7 Hz), 123.8 (q, J = 272.6 Hz), 122.9 (q, J = 3.8 Hz), 77.2, 71.1.

^{19}F NMR (235 MHz, $CDCl_3$) δ -62.8.

HRMS (ESI, *m*/*z*) calcd for $C_{10}H_7F_3O_3Na$ $[M+Na]^+$: 255.0239, found: 255.0246.

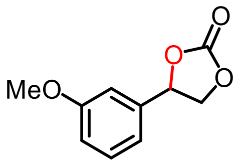

4-(3-Methoxyphenyl)-1,3-dioxolan-2-one (3h)

According to the typical procedure, a mixture of **1h** (63.1 mg, 0.2 mmol), K_2CO_3 (82.9 mg, 0.6 mmol) and **RuTES** (4.9 mg, 2 mol%) in $CHCl_3$ (4 mL, 0.05 M) under nitrogen atmosphere at room temperature stirred for 16 hours, afforded **3h** (24.2 mg, 62% yield) as a colorless oil. Purification conditions: *n*-Hex/EtOAc = 3:1. The analytical data are in accordance with the literature.[7]

^{1}H NMR (300 MHz, $CDCl_3$) δ 7.38-7.32 (m, 1H), 6.96-6.88 (m, 3H), 5.65 (t, J = 8.0 Hz, 1H), 4.79 (t, J = 8.4 Hz, 1H), 4.33 (t, J = 8.2 Hz, 1H), 3.83 (s, 3H).

^{13}C NMR (75 MHz, $CDCl_3$) δ 160.5, 154.9, 137.6, 130.6, 118.0, 115.3, 111.4, 78.0, 71.3, 55.6.

HRMS (ESI, *m*/*z*) calcd for $C_{10}H_{10}O_4Na$ $[M+Na]^+$: 217.0471, found: 217.0477.

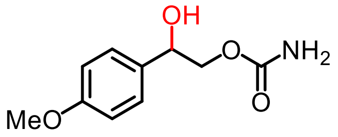

2-Hydroxy-2-(4-methoxyphenyl)ethyl carbamate (6c)

According to the typical procedure, a mixture of **1c** (63.1 mg, 0.2 mmol), K_2CO_3 (82.9 mg, 0.6 mmol) and **RuTES** (4.9 mg, 2 mol%) in $CHCl_3$ (4 mL, 0.05 M) under nitrogen atmosphere at room temperature stirred for 16 hours, afforded **6c** (22.5 mg, 53% yield) as a white solid. Purification conditions: *n*-Hex/EtOAc = 1:1.

^{1}H NMR (300 MHz, $CDCl_3$) δ 7.33-7.30 (m, 2H), 6.91-6.88 (m, 2H), 4.94-4.89 (m, 1H), 4.74 (br s, 2H), 4.25 (dd, J = 11.6 Hz, 3.4 Hz, 1H), 4.16 (dd, J = 11.6 Hz, 8.3 Hz, 1H), 3.81 (s, 3H), 2.73 (d, J = 3.2 Hz, 1H).

^{13}C NMR (75 MHz, $CDCl_3$) δ 159.7, 157.1, 132.2, 127.6, 114.8, 72.6, 70.4, 55.5.

IR (film): ν (cm^{-1}) 3456, 3311, 3184, 1730, 1679, 1607, 1510, 1241, 1172, 1058, 1030, 821, 767, 519, 404.

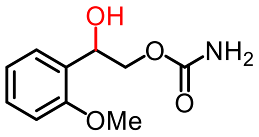

2-Hydroxy-2-(2-methoxyphenyl)ethyl carbamate (6i)

According to the typical procedure, a mixture of **1i** (63.1 mg, 0.2 mmol), K_2CO_3 (82.9 mg, 0.6 mmol) and **RuTES** (4.9 mg, 2 mol%) in $CHCl_3$ (4 mL, 0.05 M) under nitrogen atmosphere at room temperature stirred for 16 hours, afforded **6i** (34.8 mg, 82% yield) as a white solid. Purification conditions: *n*-Hex/EtOAc = 1:1.

^{1}H NMR (300 MHz, $CDCl_3$) δ 7.42 (dd, J = 7.5 Hz, 1.1 Hz, 1H), 7.31-7.26 (m, 1H), 7.01-6.96 (m, 1H), 6.88 (d, J = 8.2 Hz, 1H), 5.17 (dd, J = 7.6 Hz, 3.5 Hz, 1H), 4.78 (br s, 2H), 4.34-4.22 (m, 2H), 3.85 (s, 3H), 3.26 (br s, 1H).

^{13}C NMR (75 MHz, $CDCl_3$) δ 157.5, 156.6, 129.2, 128.0, 127.6, 121.0, 110.6, 69.6, 69.0, 55.5.

IR (film): ν (cm^{-1}) 3475, 3362, 1705, 1665, 1600, 1590, 1490, 1460, 1414, 1350, 1240, 1090, 1068, 1045, 1027, 946, 909, 749, 532, 471.

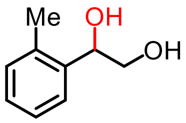

1-(*o*-Tolyl)ethane-1,2-diol (7j)

According to the typical procedure, a mixture of **1j** (59.9 mg, 0.2 mmol), K_2CO_3 (82.9 mg, 0.6 mmol) and **RuTES** (4.9 mg, 2 mol%) in $CHCl_3$ (4 mL, 0.05 M) under nitrogen atmosphere at room temperature stirred for 16 hours, then *additional basic hydrolysis* to afford **7j** (17.9 mg, 59% yield) as a white solid. Purification conditions: CH_2Cl_2/EtOAc = 3:1. The analytical data are in accordance with the literature.[8]

^{1}H NMR (300 MHz, $CDCl_3$) δ 7.51-7.48 (m, 1H), 7.25-7.13 (m, 3H), 5.07 (dd, J = 8.3 Hz, 3.3 Hz, 1H), 3.74 (dd, J = 11.4 Hz, 3.3 Hz, 1H), 3.62 (dd, J = 11.4 Hz, 8.4 Hz, 1H), 2.35 (s, 3H), 2.21 (br s, 2H).

^{13}C NMR (75 MHz, $CDCl_3$) δ 138.7, 135.0, 130.6, 127.9, 126.5, 125.8, 71.6, 67.1, 19.2.

HRMS (ESI, *m*/*z*) calcd for $C_9H_{12}O_2Na$ $[M+Na]^+$: 175.0730, found: 175.0734.

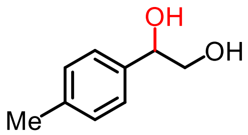

1-(*p*-Tolyl)ethane-1,2-diol (7k)

According to the typical procedure, a mixture of **1k** (59.9 mg, 0.2 mmol), K_2CO_3 (82.9 mg, 0.6 mmol) and **RuTES** (4.9 mg, 2 mol%) in $CHCl_3$ (4 mL, 0.05 M) under nitrogen atmosphere at room temperature stirred for 16 hours, then *additional basic hydrolysis* to afford **7k** (21.6 mg, 71% yield) as a white solid. Purification conditions: CH_2Cl_2/EtOAc = 3:1. The analytical data are in accordance with the literature.[8]

^{1}H NMR (300 MHz, $CDCl_3$) δ 7.26 (d, J = 8.0 Hz, 2H), 7.18 (d, J = 8.0 Hz, 2H), 4.80 (dd, J = 8.0 Hz, 3.7 Hz, 1H), 3.75 (dd, J = 11.3 Hz, 3.7 Hz, 1H), 3.66 (dd, J = 11.3 Hz, 8.0 Hz, 1H), 2.35 (s, 3H), 2.17 (br s, 2H).

^{13}C NMR (75 MHz, $CDCl_3$) δ 138.0, 137.7, 129.4, 126.2, 74.7, 68.3, 21.3.

HRMS (ESI, *m*/*z*) calcd for $C_9H_{12}O_2Na$ $[M+Na]^+$: 175.0730, found: 175.0733.

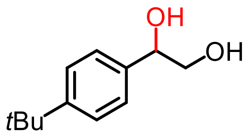

1-(4-(*tert*-Butyl)phenyl)ethane-1,2-diol (7l)

According to the typical procedure, a mixture of **1l** (68.3 mg, 0.2 mmol), K_2CO_3 (82.9 mg, 0.6 mmol) and **RuTES** (4.9 mg, 2 mol%) in $CHCl_3$ (4 mL, 0.05 M) under nitrogen atmosphere at room temperature stirred for 16 hours, then *additional basic hydrolysis* to afford **7l** (25.6 mg, 66% yield) as a white solid. Purification conditions: CH_2Cl_2/EtOAc = 2:1. The analytical data are in accordance with the literature.[9]

^{1}H NMR (300 MHz, $CDCl_3$) δ 7.41-7.38 (m, 2H), 7.32-7.29 (m, 2H), 4.82-4.80 (m, 1H), 3.79-3.66 (m, 2H), 2.39 (br s, 1H), 2.03 (br s, 1H), 1.32 (s, 9H).

^{13}C NMR (75 MHz, $CDCl_3$) δ 151.3, 137.7, 136.5, 126.0, 125.7, 74.7, 68.2, 34.8, 31.5.

HRMS (ESI, *m*/*z*) calcd for $C_{12}H_{18}O_2Na$ $[M+Na]^+$: 217.1199, found: 217.1199.

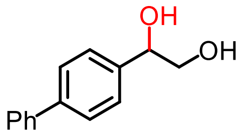

1-([1,1'-Biphenyl]-4-yl)ethane-1,2-diol (7m)

According to the typical procedure, a mixture of **1m** (72.3 mg, 0.2 mmol), K_2CO_3 (82.9 mg, 0.6 mmol) and **RuTES** (4.9 mg, 2 mol%) in $CHCl_3$ (4 mL, 0.05 M) under nitrogen atmosphere at room

temperature stirred for 16 hours, then *additional basic hydrolysis* to afford **7m** (28.7 mg, 67% yield) as a white solid. Purification conditions: CH_2Cl_2/EtOAc = 2:1. The analytical data are in accordance with the literature.[10]

^{1}H NMR (300 MHz, $CDCl_3$) δ 7.61-7.57 (m, 4H), 7.47-7.42 (m, 4H), 7.38-7.33 (m, 1H), 4.91-4.87 (m, 1H), 3.87-3.79 (m, 1H), 3.76-3.68 (m, 1H), 2.52 (d, J = 3.1 Hz, 1H), 2.07-2.03 (m, 1H).

^{13}C NMR (75 MHz, $CDCl_3$) δ 141.3, 140.9, 139.7, 129.0, 127.6, 127.5, 127.3, 126.7, 74.7, 68.3.

HRMS (ESI, *m*/*z*) calcd for $C_{14}H_{14}O_2Na$ [M+Na]$^+$: 237.0886, found: 237.0893.

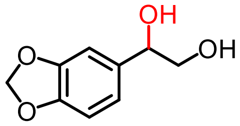

1-(Benzo[d][1,3]dioxol-5-yl)ethane-1,2-diol (7n)

According to the typical procedure, a mixture of **1n** (65.9 mg, 0.2 mmol), K_2CO_3 (82.9 mg, 0.6 mmol) and **RuTES** (4.9 mg, 2 mol%) in $CHCl_3$ (4 mL, 0.05 M) under nitrogen atmosphere at room temperature stirred for 16 hours, then *additional basic hydrolysis* to afford **7n** (21.8 mg, 60% yield) as a white solid. Purification conditions: CH_2Cl_2/EtOAc = 2:1. The analytical data are in accordance with the literature.[11]

^{1}H NMR (300 MHz, $CDCl_3$) δ 6.87 (s, 1H), 6.83-6.77 (m, 2H), 5.95 (s, 2H), 4.73 (dd, J = 8.0 Hz, 3.6 Hz, 1H), 3.76-3.59 (m, 2H), 2.63 (br s, 1H), 2.20 (br s, 1H).

^{13}C NMR (75 MHz, $CDCl_3$) δ 148.1, 147.5, 134.7, 119.7, 108.5, 106.8, 101.3, 74.7, 68.3.

HRMS (ESI, *m*/*z*) calcd for $C_9H_{10}O_4Na$ [M+Na]$^+$: 205.0471, found: 205.0477.

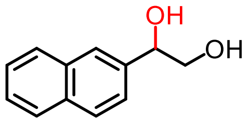

1-(Naphthalen-2-yl)ethane-1,2-diol (7o)

According to the typical procedure, a mixture of **1o** (67.1 mg, 0.2 mmol), K_2CO_3 (82.9 mg, 0.6 mmol) and **RuTES** (4.9 mg, 2 mol%) in $CHCl_3$ (4 mL, 0.05 M) under nitrogen atmosphere at room temperature stirred for 16 hours, then *additional basic hydrolysis* to afford **7o** (29.7 mg, 79% yield) as a white solid. Purification conditions: *n*-Hex/EtOAc = 2:1. The analytical data are in accordance with the literature.[12]

^{1}H NMR (300 MHz, $CDCl_3$) δ 7.86-7.84 (m, 4H), 7.52-7.46 (m, 3H), 5.01 (dd, J = 7.9 Hz, 3.6 Hz, 1H), 3.87 (dd, J = 11.3 Hz, 3.7 Hz, 1H), 3.77 (dd, J = 11.3 Hz, 7.9 Hz, 1H), 2.22 (br s, 2H).

^{13}C NMR (75 MHz, $CDCl_3$) δ 138.1, 133.5, 133.4, 128.6, 128.2, 127.9, 126.5, 126.3, 125.2, 124.1, 75.0, 68.2.

HRMS (ESI, *m*/*z*) calcd for $C_{12}H_{12}O_2Na$ $[M+Na]^+$: 211.0730, found: 211.0730.

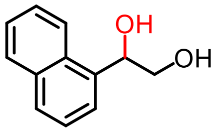

1-(Naphthalen-1-yl)ethane-1,2-diol (7p)

According to the typical procedure, a mixture of **1p** (67.1 mg, 0.2 mmol), K_2CO_3 (82.9 mg, 0.6 mmol) and **RuTES** (4.9 mg, 2 mol%) in $CHCl_3$ (4 mL, 0.05 M) under nitrogen atmosphere at room temperature stirred for 16 hours, then *additional basic hydrolysis* to afford **7p** (26.4 mg, 72% yield) as a white solid. Purification conditions: *n*-Hex/EtOAc = 2:1. The analytical data are in accordance with the literature.[13]

^{1}H NMR (300 MHz, $CDCl_3$) δ 8.09-8.06 (m, 1H), 7.90-7.87 (m, 1H), 7.81 (d, J = 8.3 Hz, 1H), 7.72 (d, J = 7.1 Hz, 1H), 7.57-7.48 (m, 3H), 5.66 (dd, J = 8.1 Hz, 3.1 Hz, 1H), 4.00 (dd, J = 11.4 Hz, 3.2 Hz, 1H), 3.81 (dd, J = 11.4 Hz, 8.1 Hz, 1H), 2.57 (br s, 1H), 2.14 (br s, 1H).

^{13}C NMR (75 MHz, $CDCl_3$) δ 136.2, 133.9, 130.6, 130.1, 129.2, 128.7, 126.5, 125.9, 125.6, 123.7, 122.9, 71.9, 67.7.

HRMS (ESI, *m*/*z*) calcd for $C_{12}H_{12}O_2Na$ $[M+Na]^+$: 211.0730, found: 211.0730.

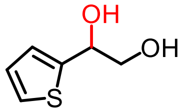

1-(Thiophen-2-yl)ethane-1,2-diol (7q)

According to the typical procedure, a mixture of **1q** (58.3 mg, 0.2 mmol), K_2CO_3 (82.9 mg, 0.6 mmol) and **RuTES** (4.9 mg, 2 mol%) in $CHCl_3$ (4 mL, 0.05 M) under nitrogen atmosphere at room temperature stirred for 16 hours, then *additional basic hydrolysis* to afford **7q** (13.9 mg, 48% yield) as a white solid. Purification conditions: *n*-Hex/EtOAc = 2:1. The analytical data are in accordance with the literature.[13]

^{1}H NMR (300 MHz, $CDCl_3$) δ 7.28 (dd, J = 4.8 Hz, 1.3 Hz, 1H), 7.02-6.98 (m, 2H), 5.06 (dd, J = 7.1

Hz, 3.9 Hz, 1H), 3.89-3.74 (m, 2H), 2.90 (br s, 1H), 2.37 (br s, 1H).

^{13}C NMR (75 MHz, $CDCl_3$) δ 144.1, 127.0, 125.3, 124.6, 70.9, 67.9.

HRMS (ESI, *m*/*z*) calcd for $C_6H_8O_2SNa$ $[M+Na]^+$: 167.0137, found: 167.0137.

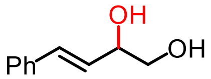

(*E*)-4-Phenylbut-3-ene-1,2-diol (7b)

According to the typical procedure, a mixture of (*E*)-**1b** (62.3 mg, 0.2 mmol), K_2CO_3 (82.9 mg, 0.6 mmol) and **RuTES** (4.9 mg, 2 mol%) in $CHCl_3$ (4 mL, 0.05 M) under nitrogen atmosphere at room temperature stirred for 16 hours, then *additional basic hydrolysis* to afford **7b** (25.6 mg, 78% yield) as a white solid. Purification conditions: *n*-Hex/EtOAc = 2:1. The analytical data are in accordance with the literature.[14]

^{1}H NMR (300 MHz, $CDCl_3$) δ 7.40-7.23 (m, 5H), 6.70 (d, *J* = 16.0 Hz, 1H), 6.20 (dd, *J* = 16.0 Hz, 6.3 Hz, 1H), 4.47-4.41 (m, 1H), 3.76 (dd, *J* = 11.1 Hz, 3.4 Hz, 1H), 3.61 (dd, *J* = 11.2 Hz, 7.3 Hz, 1H), 2.29 (br s, 1H), 2.05 (br s, 1H).

^{13}C NMR (75 MHz, $CDCl_3$) δ 136.5, 132.4, 128.8, 128.1, 127.9, 126.7, 73.4, 66.7.

HRMS (ESI, *m*/*z*) calcd for $C_{10}H_{12}O_2Na$ $[M+Na]^+$: 187.0730, found: 187.0735.

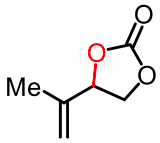

4-(Prop-1-en-2-yl)-1,3-dioxolan-2-one (3r)

According to the typical procedure, a mixture of **1r** (49.9 mg, 0.2 mmol), K_2CO_3 (82.9 mg, 0.6 mmol) and **RuTES** (4.9 mg, 2 mol%) in $CHCl_3$ (4 mL, 0.05 M) under nitrogen atmosphere at room temperature stirred for 16 hours, afforded **3r** (9.8 mg, 38% yield) as a colorless oil. Purification conditions: *n*-Hex/EtOAc = 3:1.

^{1}H NMR (300 MHz, $CDCl_3$) δ 5.16-5.15 (m, 1H), 5.13-5.07 (m, 2H), 4.56 (t, *J* = 8.5 Hz, 1H), 4.19 (dd, *J* = 8.5 Hz, 7.4 Hz, 1H), 1.80 (s, 3H).

^{13}C NMR (75 MHz, $CDCl_3$) δ 155.0, 139.3, 115.9, 79.4, 68.2, 16.6.

IR (film): ν (cm^{-1}) 2922, 2853, 1727, 1453, 1238, 1082, 1013, 894, 708.

HRMS (ESI, *m*/*z*) calcd for $C_6H_8O_3Na$ $[M+Na]^+$: 151.0366, found: 151.0370.

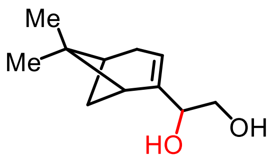

1-(6,6-Dimethylbicyclo[3.1.1]hept-2-en-2-yl)ethane-1,2-diol (7s)

According to the typical procedure, a mixture of **1s** (65.9 mg, 0.2 mmol), K_2CO_3 (82.9 mg, 0.6 mmol) and **RuTES** (4.9 mg, 2 mol%) in $CHCl_3$ (4 mL, 0.05 M) under nitrogen atmosphere at room temperature stirred for 16 hours, then *additional basic hydrolysis* to afford **7s** (16.4 mg, 45% yield) as an inseparable mixture of diastereomers (1:1 d.r.) and as a pale yellow oil. Purification conditions: *n*-Hex/EtOAc = 1:1.

^{1}H NMR (300 MHz, $CDCl_3$) δ 5.56-5.55 (m, 2H), 4.13-4.10 (m, 2H), 3.62-3.55 (m, 2H), 3.49-3.41 (m, 2H), 2.44-2.10 (m, 14H), 1.28 (s, 6H), 1.14 (dd, J = 8.7 Hz, 1.7 Hz, 2H), 0.82 (s, 3H), 0.81 (s, 3H).

^{13}C NMR (75 MHz, $CDCl_3$) δ 147.3, 147.1, 119.7, 119.0, 75.3, 75.0, 65.21, 65.17, 43.0, 42.6, 41.2 (2C), 38.1, 38.0, 32.0, 31.9, 31.39, 31.36, 26.33, 26.29, 21.51, 21.48.

IR (film): ν (cm^{-1}) 3352, 2914, 1719, 1467, 1382, 1365, 1264, 1204, 1161, 1125, 1076, 1025, 964, 877, 801, 565.

HRMS (ESI, *m*/*z*) calcd for $C_{11}H_{18}O_2Na$ [M+Na]$^+$: 205.1199, found: 205.1205.

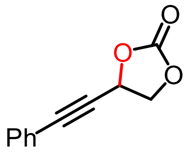

4-(Phenylethynyl)-1,3-dioxolan-2-one (3t)

According to the typical procedure, a mixture of **1t** (61.9 mg, 0.2 mmol), K_2CO_3 (82.9 mg, 0.6 mmol) and **RuTES** (4.9 mg, 2 mol%) in $CHCl_3$ (4 mL, 0.05 M) under nitrogen atmosphere at room temperature stirred for 16 hours, afforded **3t** (25.3 mg, 67% yield) as a white solid. Purification conditions: *n*-Hex/EtOAc = 3:1. The analytical data are in accordance with the literature.[15]

^{1}H NMR (300 MHz, $CDCl_3$) δ 7.49-7.46 (m, 2H), 7.43-7.32 (m, 3H), 5.55 (dd, J = 8.0 Hz, 7.0 Hz, 1H), 4.71 (t, J = 8.2 Hz, 1H), 4.49 (dd, J = 8.2 Hz, 6.9 Hz, 1H).

^{13}C NMR (75 MHz, $CDCl_3$) δ 154.1, 132.2, 129.9, 128.7, 120.9, 90.0, 82.0, 69.9, 66.9.

HRMS (ESI, *m*/*z*) calcd for $C_{11}H_8O_3Na$ [M+Na]$^+$: 211.0366, found: 211.0370.

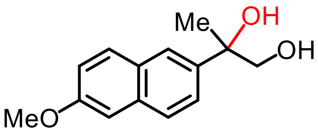

2-(6-Methoxynaphthalen-2-yl)propane-1,2-diol (7w)

According to the typical procedure, a mixture of **1w** (75.9 mg, 0.2 mmol), K_2CO_3 (82.9 mg, 0.6 mmol) and **RuTES** (4.9 mg, 2 mol%) in $CHCl_3$ (4 mL, 0.05 M) under nitrogen atmosphere at 60 °C stirred for 16 hours, then *additional basic hydrolysis* to afford **7w** (28.7 mg, 62% yield) as a white solid. Purification conditions: CH_2Cl_2/EtOAc = 2:1.

^{1}H NMR (300 MHz, $CDCl_3$) δ 7.88 (s, 1H), 7.74 (d, J = 8.6 Hz, 2H), 7.50 (dd, J = 8.6 Hz, 1.7 Hz, 1H), 7.18-7.13 (m, 2H), 3.92 (s, 3H), 3.89 (d, J = 11.1 Hz, 1H), 3.71 (d, J = 11.1 Hz, 1H), 2.66 (br s, 1H), 1.78 (br s, 1H), 1.61 (s, 3H).

^{13}C NMR (75 MHz, $CDCl_3$) δ 158.0, 140.2, 133.9, 129.8, 128.9, 127.3, 124.0, 119.3, 105.7, 75.1, 71.3, 55.5, 26.3.

IR (film): v (cm^{-1}) 3236, 2923, 1604, 1503, 1482, 1390, 1262, 1193, 1163, 1028, 959, 853, 801, 673, 469.

HRMS (ESI, m/z) calcd for $C_{14}H_{16}O_3Na$ [M+Na]$^+$: 255.0992, found: 255.0999.

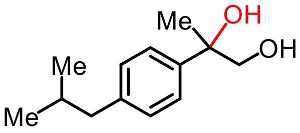

2-(4-Isobutylphenyl)propane-1,2-diol (7x)

According to the typical procedure, a mixture of **1x** (71.1 mg, 0.2 mmol), K_2CO_3 (82.9 mg, 0.6 mmol) and **RuTES** (4.9 mg, 2 mol%) in $CHCl_3$ (4 mL, 0.05 M) under nitrogen atmosphere at 60 °C stirred for 16 hours, then *additional basic hydrolysis* to afford **7x** (21.6 mg, 52% yield) as a white solid. Purification conditions: CH_2Cl_2/EtOAc = 2:1. The analytical data are in accordance with the literature.[16]

^{1}H NMR (300 MHz, $CDCl_3$) δ 7.37-7.34 (m, 2H), 7.16-7.13 (m, 2H), 3.79 (d, J = 11.1 Hz, 1H), 3.62 (d, J = 11.1 Hz, 1H), 2.47 (d, J = 7.2 Hz, 2H), 2.04 (br s, 2H), 1.93-1.74 (m, 1H), 1.53 (s, 3H), 0.91 (d, J = 6.6 Hz, 6H).

^{13}C NMR (75 MHz, $CDCl_3$) δ 142.4, 140.9, 129.4, 125.0, 74.9, 71.4, 45.2, 30.4, 26.2, 22.6.

HRMS (ESI, m/z) calcd for $C_{13}H_{20}O_2Na$ [M+Na]$^+$: 231.1356, found: 231.1362.

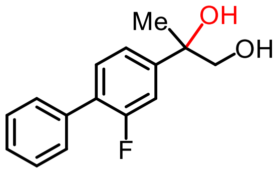

2-(2-Fluoro-[1,1'-biphenyl]-4-yl)propane-1,2-diol (7y)

According to the typical procedure, a mixture of **1y** (78.7 mg, 0.2 mmol), K_2CO_3 (82.9 mg, 0.6 mmol) and **RuTES** (4.9 mg, 2 mol%) in $CHCl_3$ (4 mL, 0.05 M) under nitrogen atmosphere at 60 °C stirred for 16 hours, then *additional basic hydrolysis* to afford **7y** (24.7 mg, 50% yield) as a pale yellow solid. Purification conditions: CH_2Cl_2/EtOAc = 2:1.

^{1}H NMR (300 MHz, $CDCl_3$) δ 7.56-7.53 (m, 2H), 7.47-7.34 (m, 4H), 7.32-7.27 (m, 2H), 3.83 (d, J = 11.1 Hz, 1H), 3.68 (d, J = 11.1 Hz, 1H), 2.67 (br s, 1H), 1.88 (br s, 1H), 1.57 (s, 3H).

^{13}C NMR (75 MHz, $CDCl_3$) δ 160.0 (d, J = 248.2 Hz), 147.0 (d, J = 7.0 Hz), 135.7, 130.92, 130.87, 129.2, 129.1, 128.7, 127.9, 121.2 (d, J = 3.3 Hz), 113.7, 113.3, 74.7, 71.1, 26.2.

^{19}F NMR (235 MHz, $CDCl_3$) δ -117.4.

IR (film): ν (cm^{-1}) 3325, 2922, 2852, 1482, 1405, 1268, 1182, 1120, 1047, 969, 890, 836, 765, 726, 696, 645, 579, 462.

HRMS (ESI, *m/z*) calcd for $C_{15}H_{15}FO_2Na$ $[M+Na]^+$: 269.0948, found: 269.0955.

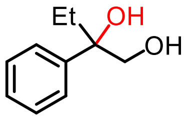

2-Phenylbutane-1,2-diol (7z)

According to the typical procedure, a mixture of **1z** (62.7 mg, 0.2 mmol), K_2CO_3 (82.9 mg, 0.6 mmol) and **RuTES** (4.9 mg, 2 mol%) in $CHCl_3$ (4 mL, 0.05 M) under nitrogen atmosphere at 60 °C stirred for 16 hours, then *additional basic hydrolysis* to afford **7z** (13.4 mg, 40% yield) as a colorless oil. Purification conditions: CH_2Cl_2/EtOAc = 2:1. The analytical data are in accordance with the literature.[17]

^{1}H NMR (300 MHz, $CDCl_3$) δ 7.43-7.32 (m, 4H), 7.30-7.24 (m, 1H), 3.83 (d, J = 11.1 Hz, 1H), 3.68 (d, J = 7.8 Hz, 1H), 2.65 (br s, 1H), 1.93-1.74 (m, 3H), 0.77 (t, J = 7.5 Hz, 3H).

^{13}C NMR (75 MHz, $CDCl_3$) δ 143.4, 128.6, 127.2, 125.8, 77.6, 70.6, 31.3, 7.6.

HRMS (ESI, *m/z*) calcd for $C_{10}H_{14}O_2Na$ $[M+Na]^+$: 189.0886, found: 189.0891.

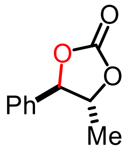

4-Methyl-5-phenyl-1,3-dioxolan-2-one (3za)

According to the typical procedure, a mixture of **1za** (59.9 mg, 0.2 mmol), K_2CO_3 (82.9 mg, 0.6 mmol) and **RuTES** (4.9 mg, 2 mol%) in $CHCl_3$ (4 mL, 0.05 M) under nitrogen atmosphere at room temperature stirred for 16 hours, afforded **3za** (13.1 mg, 37% yield, 5:1 d.r.) as a colorless oil. Purification conditions: *n*-Hex/EtOAc = 3:1. The d.r. value was determined through ^{1}H NMR of crude materials. The configuration was assigned by comparison with the reported compound.[18] The analytical data are in accordance with the literature.[18]

^{1}H NMR (300 MHz, $CDCl_3$) δ 7.46-7.34 (m, 5H), 5.13 (d, J = 8.0 Hz, 1H), 4.65-4.56 (m, 1H), 1.56 (d, J = 6.2 Hz, 3H).

^{13}C NMR (75 MHz, $CDCl_3$) δ 154.4, 135.3, 129.9, 129.4, 126.2, 85.1, 80.9, 18.5.

HRMS (ESI, *m/z*) calcd for $C_{10}H_{10}O_3Na$ [M+Na]$^+$: 201.0522, found: 201.0528.

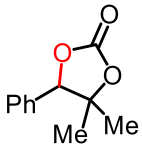

4,4-Dimethyl-5-phenyl-1,3-dioxolan-2-one (3zb)

According to the typical procedure, a mixture of **1zb** (62.7 mg, 0.2 mmol), K_2CO_3 (82.9 mg, 0.6 mmol) and **RuTES** (4.9 mg, 2 mol%) in $CHCl_3$ (4 mL, 0.05 M) under nitrogen atmosphere at room temperature stirred for 16 hours, afforded **3zb** (26.2 mg, 68% yield) as a colorless oil. Purification conditions: *n*-Hex/EtOAc = 3:1.

^{1}H NMR (300 MHz, $CDCl_3$) δ 7.45-7.36 (m, 3H), 7.33-7.27 (m, 2H), 5.36 (s, 1H), 1.66 (s, 3H), 1.01 (s, 3H).

^{13}C NMR (75 MHz, $CDCl_3$) δ 154.2, 133.6, 129.4, 129.0, 125.6, 86.5, 85.4, 26.8, 23.0.

IR (film): ν (cm^{-1}) 2984, 1752, 1456, 1329, 1265, 1236, 1115, 1044, 1028, 1000, 775, 746, 700, 633, 494.

HRMS (ESI, *m/z*) calcd for $C_{11}H_{12}O_3Na$ [M+Na]$^+$: 215.0679, found: 215.0685.

5. Stereochemical Aspects of Intramolecular C(sp^3)-H Oxygenation *versus* Nitrogenation

5.1 Enantioselective Cyclic Carbonate Formation by C(sp^3)-H Oxygenation

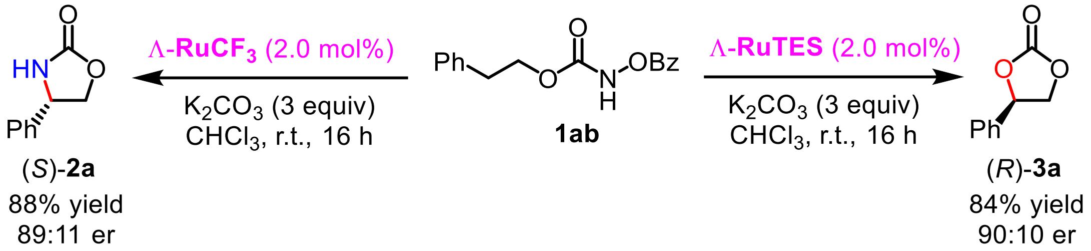

C(sp^3)-H oxygenation with Λ-RuTES: According to the standard procedure, a mixture of **1ab** (57.1 mg, 0.2 mmol), K_2CO_3 (82.9 mg, 0.6 mmol) and enantiomerically pure Λ-**RuTES** (4.9 mg, 2 mol%) in $CHCl_3$ (4 mL, 0.05 M) under nitrogen atmosphere at room temperature was stirred for 16 hours to afford **3a** (27.5 mg, 84% yield, 90:10 er) as a white solid. Purification conditions: *n*-Hex/EtOAc = 3:1. The enantiomeric ratio of 90:10 er was established by HPLC analysis (Chiralpak OD-H column, absorbance at 216 nm, mobile phase *n*-hexane/isopropanol = 80:20, flow rate: 1.0 mL/min, 25 °C, t_r(major) = 12.4 min, t_r(minor) = 14.8 min; $[\alpha]_D^{22}$ = -34.2° (*c* 1.0, CH_2Cl_2, 90:10 er). (Lit.[19] 74% ee for *R*-configuration, HPLC: OD-H, 216 nm, *n*-hexane/isopropanol = 80:20, flow rate: 1.0 mL/min, 25 °C, t_r(major) = 13.0 min, t_r(minor) = 15.7 min). The analytical data are in accordance with the racemic product.

C(sp^3)-H amination with Λ-RuCF$_3$: According to the standard procedure, a mixture of **1ab** (57.1 mg, 0.2 mmol), K_2CO_3 (82.9 mg, 0.6 mmol) and enantiomerically pure Λ-**RuCF$_3$** (4.4 mg, 2 mol%) in $CHCl_3$ (4 mL, 0.05 M) under nitrogen atmosphere at room temperature was stirred for 16 hours to afford (*S*)-**2a** (28.7 mg, 88% yield, 89:11 er) as a white solid. Purification conditions: *n*-Hex/EtOAc = 1:1. The enantiomeric ratio of 89:11 er was established by HPLC analysis (Chiralpak OD-H column, absorbance at 210 nm, mobile phase *n*-hexane/isopropanol = 80:20, flow rate: 1.0 mL/min, 25 °C, t_r(minor) = 13.8 min, t_r(major) = 15.5 min; $[\alpha]_D^{22}$ = +20.3° (*c* 1.0, CH_2Cl_2, 89:11 er). (Lit.[20] $[\alpha]_D^{25}$ = -40.8° (*c* 0.86, $CHCl_3$, 91:9 er) for *R*-configuration, HPLC: OD-H, 254 nm, *n*-hexane/isopropanol = 93:7, flow rate: 0.9 mL/min, 25 °C, t_r(major) = 14.0 min, t_r(minor) = 17.2 min). The analytical data are in accordance with the literature.[20]

^{1}H NMR (300 MHz, $CDCl_3$) δ 7.44-7.33 (m, 5H), 5.61 (s, 1H), 4.96 (t, *J* = 7.8 Hz, 1H), 4.74 (t, *J* =

8.6 Hz, 1H), 4.20 (dd, J = 8.5 Hz, 7.0 Hz, 1H).

^{13}C NMR (75 MHz, $CDCl_3$) δ 159.6, 139.6, 129.4, 129.1, 126.2, 72.7, 56.6.

HRMS (ESI, *m/z*) calcd for $C_9H_9NO_2Na$ $[M+Na]^+$: 186.0525, found: 186.0531.

Further examples of catalytic enantioselective C(sp^3)-H oxygenations to form cyclic carbonates using Λ-RuTES:

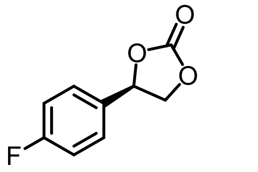

(*R*)-**3f**
85% yield, 88.5:11.5 er

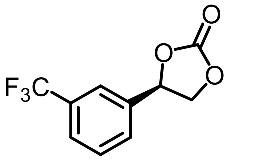

(*R*)-**3g**
70% yield, 65:35 er

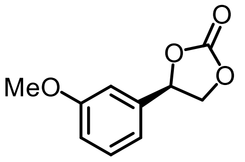

(*R*)-**3h**
61% yield, 87:13 er

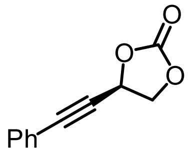

(*R*)-**3t**
67% yield, 71.5:28.5 er

Synthetic procedures and analytical data:

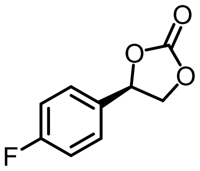

(*R*)-4-(4-Fluorophenyl)-1,3-dioxolan-2-one (3f)

According to the standard procedure, a mixture of **1f** (60.7 mg, 0.2 mmol), K_2CO_3 (82.9 mg, 0.6 mmol) and Λ-**RuTES** (4.9 mg, 2 mol%) in $CHCl_3$ (4 mL, 0.05 M) under nitrogen atmosphere at room temperature was stirred for 16 hours to afford **3f** (30.8 mg, 85% yield, 88.5:11.5 er) as a white solid. Purification conditions: *n*-Hex/EtOAc = 3:1. Enantiomeric ratio of er = 88.5:11.5 established by HPLC analysis (Chiralpak IG column, absorbance at 216 nm, *n*-hexane/isopropanol = 80:20, flow rate: 1.0 mL/min, 25 °C, t_r(major) = 10.8 min, t_r(minor) = 12.0 min). The analytical data are in accordance with the racemic product.

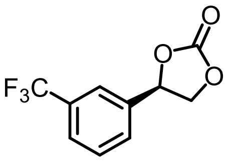

(*R*)-4-(3-(Trifluoromethyl)phenyl)-1,3-dioxolan-2-one (3g)

According to the standard procedure, a mixture of **1g** (70.6 mg, 0.2 mmol), K_2CO_3 (82.9 mg, 0.6 mmol) and Λ-**RuTES** (4.9 mg, 2 mol%) in $CHCl_3$ (4 mL, 0.05 M) under nitrogen atmosphere at room temperature was stirred for 16 hours to afford **3g** (32.6 mg, 70% yield, 65:35 er) as a white solid.

Purification conditions: *n*-Hex/EtOAc = 3:1. Enantiomeric ratio of 65:35 er established by HPLC analysis (Chiralpak OD-H column, absorbance at 216 nm, *n*-hexane/isopropanol = 90:10, flow rate: 1.0 mL/min, 25 °C, t_r(major) = 17.1 min, t_r(minor) = 18.5 min). The analytical data are in accordance with the racemic product.

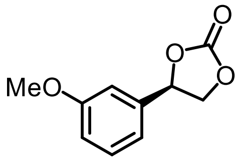

(*R*)-4-(3-Methoxyphenyl)-1,3-dioxolan-2-one (3h)

According to the standard procedure, a mixture of **1h** (63.1 mg, 0.2 mmol), K_2CO_3 (82.9 mg, 0.6 mmol) and Λ-**RuTES** (4.9 mg, 2 mol%) in $CHCl_3$ (4 mL, 0.05 M) under nitrogen atmosphere at room temperature was stirred for 16 hours to afford **3h** (23.6 mg, 61% yield, 87:13 er) as a white solid. Purification conditions: *n*-Hex/EtOAc = 3:1. Enantiomeric ratio of 87:13 er established by HPLC analysis (Chiralpak OD-H column, absorbance at 216 nm, *n*-hexane/isopropanol = 80:20, flow rate: 1.0 mL/min, 25 °C, t_r(major) = 15.9 min, t_r(minor) = 24.0 min). The analytical data are in accordance with the racemic product.

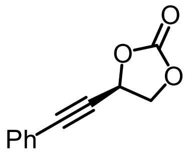

(*R*)-4-(Phenylethynyl)-1,3-dioxolan-2-one (3t)

According to the standard procedure, a mixture of **1t** (61.9 mg, 0.2 mmol), K_2CO_3 (82.9 mg, 0.6 mmol) and Λ-**RuTES** (4.9 mg, 2 mol%) in $CHCl_3$ (4 mL, 0.05 M) under nitrogen atmosphere at room temperature was stirred for 16 hours to afford **3t** (25.2 mg, 67% yield, 71.5:28.5) as a white solid. Purification conditions: *n*-Hex/EtOAc = 3:1. Enantiomeric ratio of 71.5:28.5 er established by HPLC analysis (Chiralpak OD-H column, absorbance at 216 nm, *n*-hexane/isopropanol = 80:20, flow rate: 1.0 mL/min, 25 °C, t_r(minor) = 11.9 min, t_r(major) = 21.6 min). The analytical data are in accordance with the racemic product.

5.2 Racemic Formation of Acyclic Carbamate by C(sp^3)-H Oxygenation

In contrast to the enantioselective formation of the cyclic carbonate products **3**, the acyclic carbamates **6** form in a racemic or almost racemic fashion. Below, an example is shown for the *p*-tolyl substrate **1k**, which generates both the cyclic carbonate and the acyclic carbamate. The cyclic carbonate (*R*)-**3k** is formed in an enantioselective fashion with 86.5:13.5 er, while the acyclic carbamate **6k** is form in an almost racemic fashion with 54:46 er.

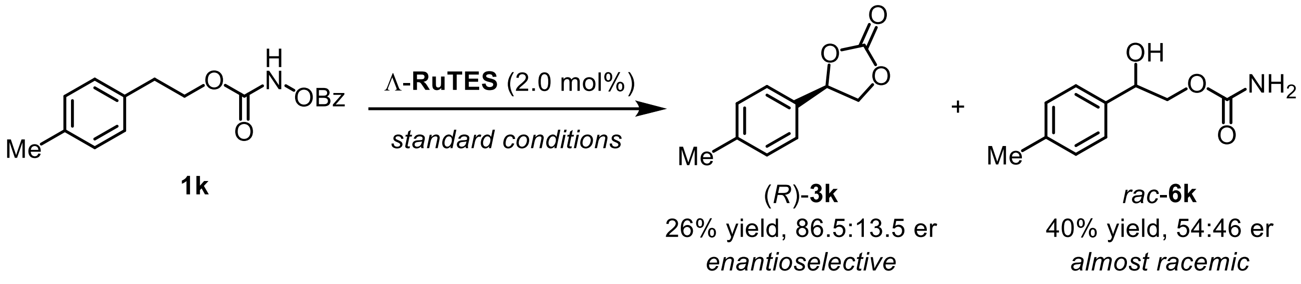

According to the standard procedure, a mixture of **1k** (59.9 mg, 0.2 mmol), K_2CO_3 (82.9 mg, 0.6 mmol) and Λ-**RuTES** (4.9 mg, 2 mol%) in $CHCl_3$ (4 mL, 0.05 M) under nitrogen atmosphere at room temperature was stirred for 16 hours to afford (*R*)-**3k** (9.2 mg, 26% yield, 86.5:13.5 er) and *rac*-**6k** (15.7 mg, 40% yield, 54:46 er) as white solids. Purification conditions: *n*-Hex/EtOAc = 3:1 to 1:1. Enantiomeric excess of (*R*)-**3k** was established by HPLC analysis (Chiralpak IG column, absorbance at 216 nm, *n*-hexane/isopropanol = 80:20, flow rate: 1.0 mL/min, 25 °C, t_r(major) = 12.3 min, t_r(minor) = 14.2 min). The analytical data are in accordance with the literature.[7]

^{1}H NMR (300 MHz, $CDCl_3$) δ 7.25 (s, 4H), 5.64 (t, *J* = 8.0 Hz, 1H), 4.77 (t, *J* = 8.4 Hz, 1H), 4.34 (dd, *J* = 8.5 Hz, 8.0 Hz, 1H), 2.38 (s, 3H).

^{13}C NMR (75 MHz, $CDCl_3$) δ 155.0, 140.1, 132.9, 130.1, 126.2, 78.3, 71.3, 21.4.

The almost racemic nature of **6k** was established by HPLC analysis (Chiralpak OD-H column, absorbance at 216 nm, *n*-hexane/isopropanol = 80:20, flow rate: 1.0 mL/min, 25 °C, t_r(major) = 8.3 min, t_r(minor) = 9.7 min).

^{1}H NMR (300 MHz, $CDCl_3$) δ 7.29-7.25 (m, 2H), 7.19-7.16 (m, 2H), 4.94-4.91 (m, 1H), 4.79 (br s, 2H), 4.26 (dd, *J* = 11.6 Hz, 3.3 Hz, 1H), 4.16 (dd, *J* = 11.6 Hz, 8.3 Hz, 1H), 2.83 (s, 1H), 2.35 (s, 3H).

^{13}C NMR (75 MHz, $CDCl_3$) δ 157.3, 138.1, 137.1, 129.4, 126.3, 72.8, 70.3, 21.3.

IR (film): ν (cm^{-1}) 3455, 3381, 3202, 1731, 1697, 1598, 1404, 1338, 1085, 1048, 844, 811, 769, 552, 452.

Further examples of the racemic formation of acyclic carbamates using Λ-RuTES:

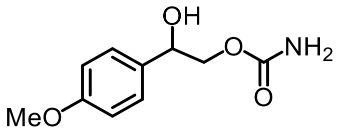

2-Hydroxy-2-(4-methoxyphenyl)ethyl carbamate (6c)

According to the standard procedure, a mixture of **1c** (63.1 mg, 0.2 mmol), K_2CO_3 (82.9 mg, 0.6 mmol) and Λ-**RuTES** (4.9 mg, 2 mol%) in $CHCl_3$ (4 mL, 0.05 M) under nitrogen atmosphere at room temperature was stirred for 16 hours to afford **6c** (22.0 mg, 52% yield, 50:50 er) as a white solid. Purification conditions: *n*-Hex/EtOAc = 1:1. The racemic nature was established by HPLC analysis (Chiralpak OD-H column, absorbance at 216 nm, *n*-hexane/isopropanol = 80:20, flow rate: 1.0 mL/min, 25 °C, t_r(major) = 10.7 min, t_r(minor) = 12.8 min). The analytical data are in accordance with the racemic product.

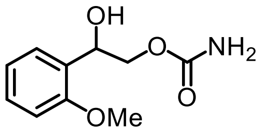

2-Hydroxy-2-(2-methoxyphenyl)ethyl carbamate (6i)

According to the standard procedure, a mixture of **1i** (63.1 mg, 0.2 mmol), K_2CO_3 (82.9 mg, 0.6 mmol) and Λ-**RuTES** (4.9 mg, 2 mol%) in $CHCl_3$ (4 mL, 0.05 M) under nitrogen atmosphere at room temperature was stirred for 16 hours to afford **6i** (34.8 mg, 82% yield, 55:45 er) as a white solid. Purification conditions: *n*-Hex/EtOAc = 1:1. The almost racemic nature was established by HPLC analysis (Chiralpak OD-H column, absorbance at 216 nm, *n*-hexane/isopropanol = 85:15, flow rate: 1.0 mL/min, 25 °C, t_r(minor) = 19.3 min, t_r(major) = 20.8 min). The analytical data are in accordance with the racemic product.

Mechanistic explanation: The racemic formation of the acyclic carbamate is fully consistent with the proposed mechanism through an intermediate iminocarbonate (**IV** in Figure 2). Both the cyclic carbonate products and the acyclic carbamate products are generated from the iminocarbonate intermediate **IV** by hydrolysis. The cyclic carbonates are formed by *exocyclic hydrolysis* of the imine without affecting the stereocenter but the acyclic carbamates are formed by *ring-opening hydrolysis* via an S_N1 mechanism through an intermediate carbocation which destroys the stereochemical

information. This mechanism also explains why the acyclic carbonate product formation is favored for substrates with electron rich substituents since they can better stabilize the intermediate carbocation.

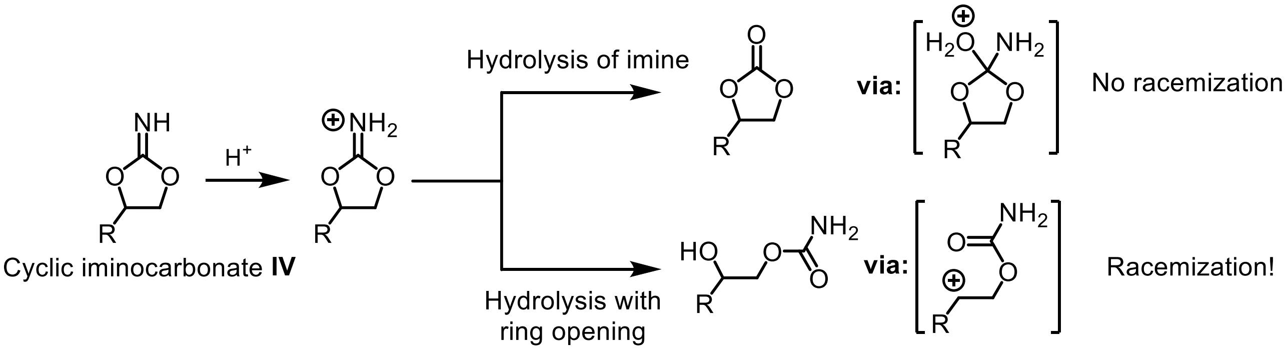

5.3 C-H Oxygenation and C-H Amination at a Stereocenter with Racemic Catalyst

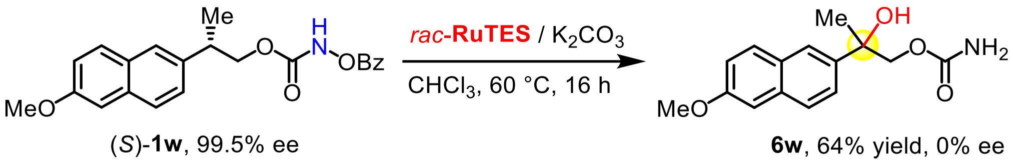

The chiral substrate (*S*)-**1w** was synthesized starting from commercially available Naproxen (CAS number 22204-53-1). The enantiopurity of the substrate was determined by HPLC analysis using a Chiralpak OD-H column, ee = 99.5% (HPLC: Chiralpak OD-H column, 254 nm, *n*-hexane/isopropanol = 75:25, flow rate: 1.0 mL/min, 25 °C, t_r(minor) = 10.9 min, t_r(major) = 14.2 min).

C(sp^3)-H oxygenation: According to the standard procedure, (*S*)-**1w** (75.9 mg, 0.2 mmol, 99.5% ee), K_2CO_3 (82.9 mg, 0.6 mmol) and *rac*-**RuTES** (4.9 mg, 2 mol%) in $CHCl_3$ (4 mL, 0.05 M) under nitrogen atmosphere at 60 °C stirred for 16 hours to afford **6w** (35.1 mg, 64% yield, 0% ee). Enantiomeric excess was established by HPLC analysis (Chiralpak OD-H column, absorbance at 220 nm, *n*-hexane/isopropanol = 80:20, flow rate: 1.0 mL/min, 25 °C, t_r(major) = 9.2 min, t_r(minor) = 11.0 min).

^{1}H NMR (300 MHz, $CDCl_3$) δ 7.89 (m, 1H), 7.74 (dd, J = 8.7 Hz, 2.6 Hz, 2H), 7.52 (dd, J = 8.7 Hz, 1.8 Hz, 1H), 7.17-7.13 (m, 2H), 4.65 (br s, 2H), 4.41-4.32 (m, 2H), 3.92 (s, 3H), 1.63 (s, 3H).

^{13}C NMR (75 MHz, $CDCl_3$) δ 158.0, 157.2, 139.7, 133.9, 129.9, 128.8, 127.1, 124.1, 124.0, 119.2, 105.7, 74.3, 72.8, 55.5, 26.8.

IR (film): ν (cm^{-1}) 3473, 3323, 1690, 1596, 14010, 1391, 1375, 1327, 1260, 1201, 1164, 1133, 1057,

1028, 853, 815, 747, 659, 517, 475, 449.

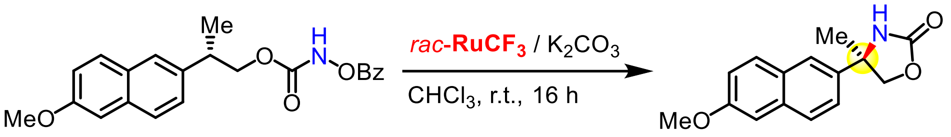

(*S*)-**1w** 99.5% ee (*R*)-**2w** 88% yield, 91% ee

C(sp^3)-H amination: According to the standard procedure, (*S*)-**1w** (75.9 mg, 0.2 mmol, 99.5% ee), K_2CO_3 (82.9 mg, 0.6 mmol) and *rac*-**RuCF$_3$** (4.4 mg, 2 mol%) in $CHCl_3$ (4 mL, 0.05 M) under nitrogen atmosphere at room temperature was stirred for 16 hours to afford (*R*)-**2w** (45 mg, 88% yield, 91% ee) as a white solid. Purification conditions: *n*-Hex/EtOAc = 2:1. Enantiomeric excess established by HPLC analysis (Chiralpak OD-H column, absorbance at 210 nm, *n*-hexane/isopropanol = 80:20, flow rate: 1.0 mL/min, 25 °C, t_r(minor) = 10.3 min, t_r(major) = 13.8 min. The analytical data are in accordance with the literature.[21]

^{1}H NMR (300 MHz, $CDCl_3$) δ 7.79-7.71 (m, 3H), 7.43 (dd, *J* = 8.6 Hz, 1.9 Hz, 1H), 7.20-7.12 (m, 2H), 6.02 (s, 1H), 4.42 (s, 2H), 3.92 (s, 3H), 1.84 (s, 3H).

^{13}C NMR (75 MHz, $CDCl_3$) δ 159.2, 158.4, 138.4, 134.1, 129.8, 128.6, 128.1, 123.5, 123.4, 119.8, 105.8, 78.3, 60.5, 55.5, 27.7.

Discussion: The C-H amination occurs under retention of configuration with a slight deterioration of the enantiomeric excess, which is consistent with the proposed mechanism through a diradical intermediate (**II** in Figure 2) followed by a rapid radical-radical recombination. On the other hand, the C-H oxygenation reaction does not provide any further insight because it has been already established that the acyclic carbamate products always form in a racemic fashion.

6. Mechanistic Experiments

6.1 Trapping of Ru-Nitrenoid Intermediate

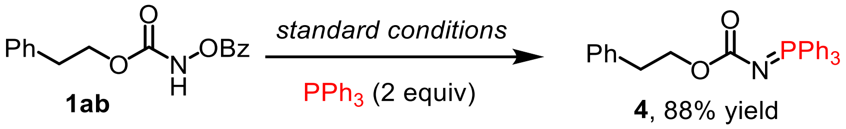

According to the typical procedure, a mixture of **1ab** (57.1 mg, 0.2 mmol), K_2CO_3 (82.9 mg, 0.6 mmol), triphenylphosphine (104.9 mg, 0.4 mmol) and **RuTES** (4.9 mg, 2 mol%) in $CHCl_3$ (4 mL, 0.05 M) under nitrogen atmosphere at room temperature was stirred for 16 hours to afford **4** (72.9 mg, 88% yield) as a white solid. Purification conditions: *n*-Hex/EtOAc = 2:1 to 1:2.

^{1}H NMR (300 MHz, CD_3CN) δ 7.73-7.59 (m, 9H), 7.54-7.48 (m, 6H), 7.30-7.17 (m, 5H), 4.15 (t, *J* = 6.8 Hz, 2H), 2.84 (t, *J* = 6.8 Hz, 2H).

^{13}C NMR (75 MHz, CD_3CN) δ 162.49, 162.46, 140.2, 133.8, 133.7, 133.6, 133.5, 130.1, 129.88, 129.87, 129.7, 129.3, 128.7, 127.1, 66.62, 66.58, 36.4.

HRMS (ESI, *m*/*z*) calcd for $C_{27}H_{24}NO_2PH$ $[M+H]^+$: 426.1617, found: 426.1628.

6.2 Olefin Isomerization

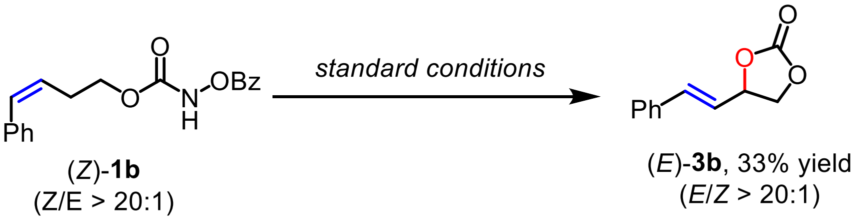

According to the typical procedure, a mixture of (*Z*)-**1b** (62.3 mg, 0.2 mmol), K_2CO_3 (82.9 mg, 0.6 mmol) and **RuTES** (4.9 mg, 2 mol%) in $CHCl_3$ (4 mL, 0.05 M) under nitrogen atmosphere at room temperature was stirred for 16 hours to afford (*E*)-**3b** (12.6 mg, 33% yield) as a white solid. Purification conditions: *n*-Hex/EtOAc = 3:1. The analytical data are in accordance with the literature.[14]

^{1}H NMR (500 MHz, $CDCl_3$) δ 7.43-7.30 (m, 5H), 6.79 (d, *J* = 15.8 Hz, 1H), 6.18 (dd, *J* = 15.8 Hz, 7.8 Hz, 1H), 5.30 (q, *J* = 7.7 Hz, 1H), 4.65 (t, *J* = 8.3 Hz, 1H), 4.24 (t, *J* = 8.2 Hz, 1H).

^{13}C NMR (125 MHz, $CDCl_3$) δ 154.9, 137.0, 135.0, 129.3, 129.0, 127.2, 122.5, 77.9, 69.5.

6.3 Trapping of Radical Intermediate with 4-MeO-TEMPO

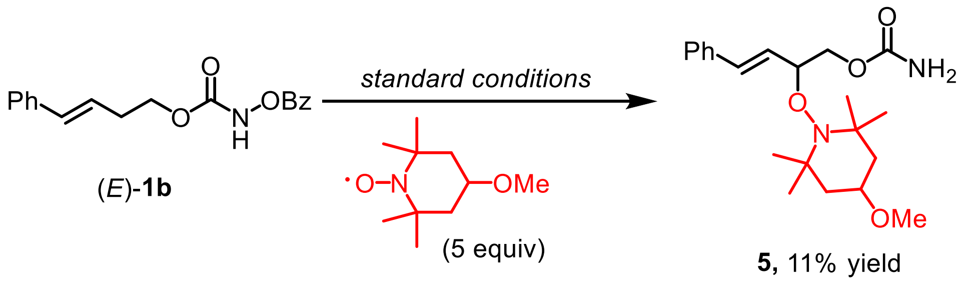

According to the typical procedure, a mixture of (*E*)-**1b** (62.3 mg, 0.2 mmol), K_2CO_3 (82.9 mg, 0.6 mmol), 4-MeO-TEMPO (186.3 mg, 1.0 mmol) and **RuTES** (4.9 mg, 2 mol%) in $CHCl_3$ (4 mL, 0.05 M) under nitrogen atmosphere at room temperature was stirred for 16 hours to afford **5** (8.4 mg, 11% yield) as a white solid. Purification conditions: *n*-Hex/EtOAc = 5:1.

^{1}H NMR (500 MHz, $CDCl_3$) δ 7.40-7.22 (m, 5H), 6.55 (d, J = 16.0 Hz, 1H), 6.15 (dd, J = 16.0 Hz, 8.4 Hz, 1H), 4.59-4.52 (m, 3H), 4.33 (dd, J = 11.2 Hz, 5.7 Hz, 1H), 4.19 (dd, J = 11.2 Hz, 5.5 Hz, 1H), 3.48-3.38 (m, 1H), 3.31 (s, 3H), 1.87-1.80 (m, 2H), 1.45-1.37 (m, 2H), 1.29 (s, 3H), 1.21 (s, 3H), 1.16 (s, 3H), 1.15 (s, 3H).

^{13}C NMR (125 MHz, $CDCl_3$) δ 156.6, 136.9, 133.0, 128.7, 128.3, 127.8, 126.6, 82.9, 71.8, 66.1, 60.8, 60.1, 55.8, 45.2, 45.1, 35.3, 34.1, 21.5, 21.4.

HRMS (ESI, *m*/*z*) calcd for $C_{21}H_{32}N_2O_4H$ $[M+H]^+$: 377.2435, found: 377.2447.

7. Single Crystal X-Ray Diffraction

7.1 Single Crystal X-Ray Analysis of *rac*-RuTES

Single crystals of *rac*-**RuTES** suitable for X-ray diffraction were obtained by slow diffusion from the solution in CH_3CN layered with THF and Et_2O at room temperature for several days in a NMR tube. Intensity data of the selected crystal were recorded with a STADIVARI diffractometer (Stoe & Cie). The diffractometer was operated with Cu-Kα radiation (1.54186 Å, microfocus source) and equipped with a Dectris PILATUS 300K detector. Evaluation, integration and reduction of the diffraction data was carried out using the X-Area software suite.[22] Multi-scan and numerical absorption corrections were applied with the X-Red32 and LANA modules of the X-Area software suite.[23,24] The structure was solved using dual-space methods (SHELXT-2014/5) and refined against F2 (SHELXL-2018/3 using ShelXle interface).[25-27] The dataset in HKLF5 format was employed for the refinement using non-overlapping reflections of the first twin component merged in the point group 1 and all overlapping reflections merged in the point group 1 (HKLF5Tools program).[28] All non-hydrogen atoms were refined with anisotropic displacement parameters. The hydrogen atoms were refined using the "riding model" approach with isotropic displacement parameters 1.2 times (for CH_3 groups 1.5 times) of that of the preceding carbon atom. One of the terminal ethyl groups was disordered between two positions and was refined accordingly. One of the two $[PF_6]^-$ was rotationally disordered in a complicated manner and was refined after splitting it into two positions using the DSR plugin in the ShelXle program.[27,29] CCDC 1981337 contains the supplementary crystallographic data for this paper. These data can be obtained free of charge from The Cambridge Crystallographic Data Centre via www.ccdc.cam.ac.uk/structures.

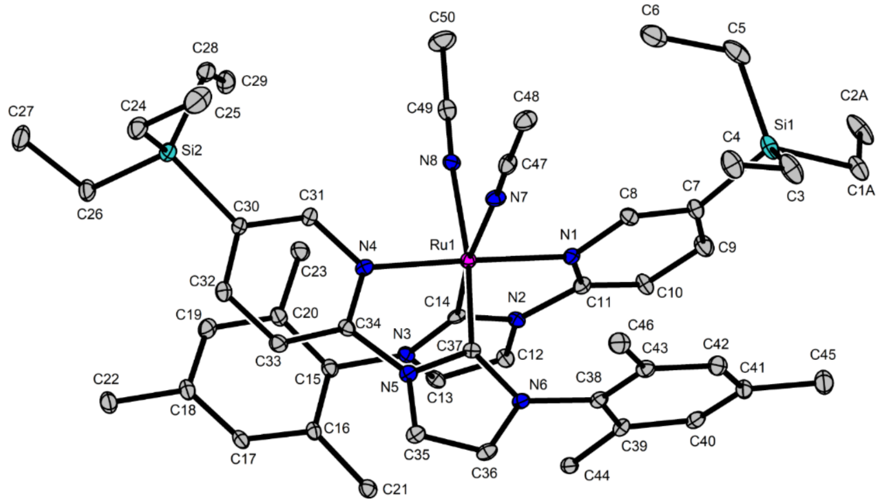

Selected crystallographic data and details of the structure determination for *rac*-**RuTES**.

Identification code	TYQI88
Empirical formula	$C_{52}H_{71}F_{12}N_9P_2RuSi_2$
Molar mass / g·mol^{-1}	1269.36
Space group (No.)	$P\bar{1}$ (2)
a / Å	11.7453(4)
b / Å	13.9640(5)
c / Å	19.9058(6)
α / °	85.293(3)
β / °	78.389(2)
γ / °	67.668(3)
V / Å^3	2958.06(18)
Z	2
$\rho_{calc.}$ / g·cm^{-3}	1.425
μ / mm^{-1}	3.749
Color	yellow
Crystal habitus	needle
Crystal size / mm^3	0.251 x 0.035 x 0.020
T / K	100
λ / Å	1.54186 (Cu-K$_\alpha$)
θ range / °	4.094 to 75.824
Range of Miller indices	$-14 \leq h \leq 14$
	$-17 \leq k \leq 17$
	$-24 \leq l \leq 14$
Absorption correction	multi-scan and numerical
T_{min}, T_{max}	0.1587, 0.8224
R_{int}, R_σ	0.0693, 0.1848
Completeness of the data set	0.986
No. of measured reflections	110383
No. of independent reflections	17776
No. of parameters	758
No. of restrains	215
S (all data)	0.803
$R(F)$ ($I \geq 2\sigma(I)$, all data)	0.0583, 0.0877
$wR(F^2)$ ($I \geq 2\sigma(I)$, all data)	0.1121, 0.1195
Extinction coefficient	not refined
Volume fraction of the 2nd twin component (BASF)	0.3487(9)
$\Delta\rho_{max}$, $\Delta\rho_{min}$ / e·Å^{-3}	1.006, –1.388

7.2 Single Crystal X-Ray Analysis of 6c

Single crystals of **6c** suitable for X-ray diffraction were obtained by slow diffusion from the solution in CH_2Cl_2 layered with THF and *n*-Hexane at room temperature for several days in a NMR tube. Intensity data of the crystal were recorded with a STADIVARI diffractometer (Stoe & Cie). The diffractometer was operated with Cu-Kα radiation (1.54186 Å, microfocus source) and equipped with a Dectris PILATUS 300K detector. Evaluation, integration and reduction of the diffraction data was carried out using the X-Area software suite.[22] Multi-scan and numerical absorption corrections were applied with the X-Red32 and LANA modules of the X-Area software suite.[23,24] The structure was solved using dual-space methods (SHELXT-2014/5) and refined against F2 (SHELXL-2018/3).[25,26] All non-hydrogen atoms were refined with anisotropic displacement parameters. The hydrogen atoms next to carbon atoms were refined using the "riding model" approach with isotropic displacement parameters 1.2 times (for CH_3 groups 1.5 times) of that of the preceding carbon atom. The hydrogen atoms of the $–NH_2$ and –OH groups were refined using the "riding model" and freely refined isotropic displacement parameters. CCDC 1981338 contains the supplementary crystallographic data for this paper. These data can be obtained free of charge from The Cambridge Crystallographic Data Centre via www.ccdc.cam.ac.uk/structures.

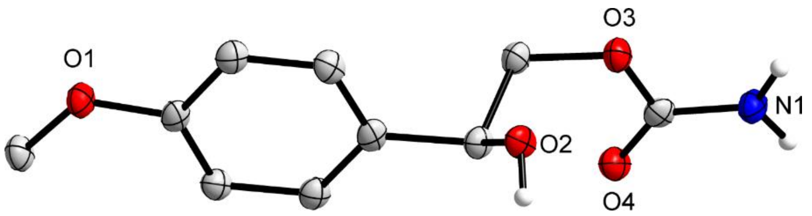

Selected crystallographic data and details of the structure determination for **6c**.

Identification code	TYQI104c
Empirical formula	$C_{10}H_{13}NO_4$
Molar mass / g·mol^{-1}	211.21
Space group (No.)	$P\bar{1}$ (2)
a / Å	6.6904(13)
b / Å	8.4110(17)
c / Å	9.1107(18)
α / °	97.76(3)
β / °	99.48(3)
γ / °	100.49(3)
V / Å^3	489.97(18)
Z	2
$\rho_{calc.}$ / g·cm^{-3}	1.432
μ / mm^{-1}	0.937
Color	colorless
Crystal habitus	block
Crystal size / mm^3	0.166 x 0.081 x 0.064
T / K	100
λ / Å	1.54186 (Cu-K$_\alpha$)
θ range / °	4.995 to 75.270
Range of Miller indices	$-6 \leq h \leq 8$
	$-10 \leq k \leq 9$
	$-11 \leq l \leq 9$
Absorption correction	multi-scan and numerical
$T_{\min}$, $T_{\max}$	0.3150, 0.9255
R_{int}, R_σ	0.0220, 0.0159
Completeness of the data set	0.994
No. of measured reflections	8903
No. of independent reflections	2001
No. of parameters	141
No. of restrains	0
No. of constrains	0
S (all data)	1.071
$R(F)$ ($I \geq 2\sigma(I)$, all data)	0.0423, 0.0463
$wR(F^2)$ ($I \geq 2\sigma(I)$, all data)	0.1213, 0.1241
Extinction coefficient	not refined
$\Delta\rho_{\max}$, $\Delta\rho_{\min}$ / e·Å^{-3}	0.263, –0.338

8. Enantioselectivities as Determined by Chiral HPLC

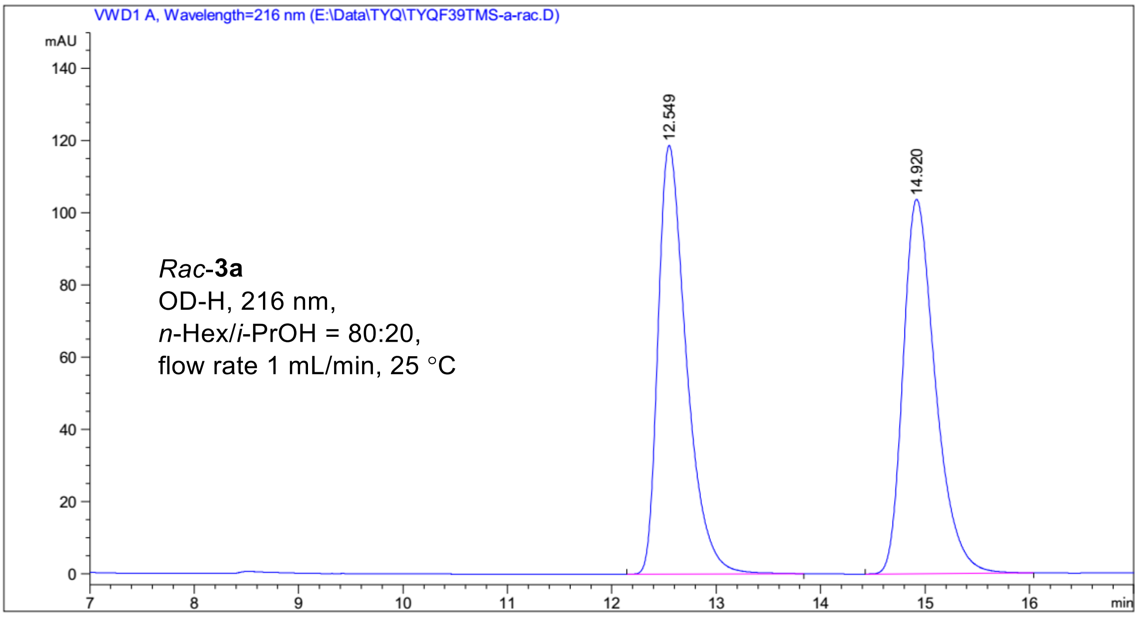

Peak #	RetTime [min]	Type	Width [min]	Area [mAU*s]	Height [mAU]	Area %
1	12.549	BB	0.2861	2241.75732	118.81087	50.0708
2	14.920	BB	0.3282	2235.41870	103.80527	49.9292

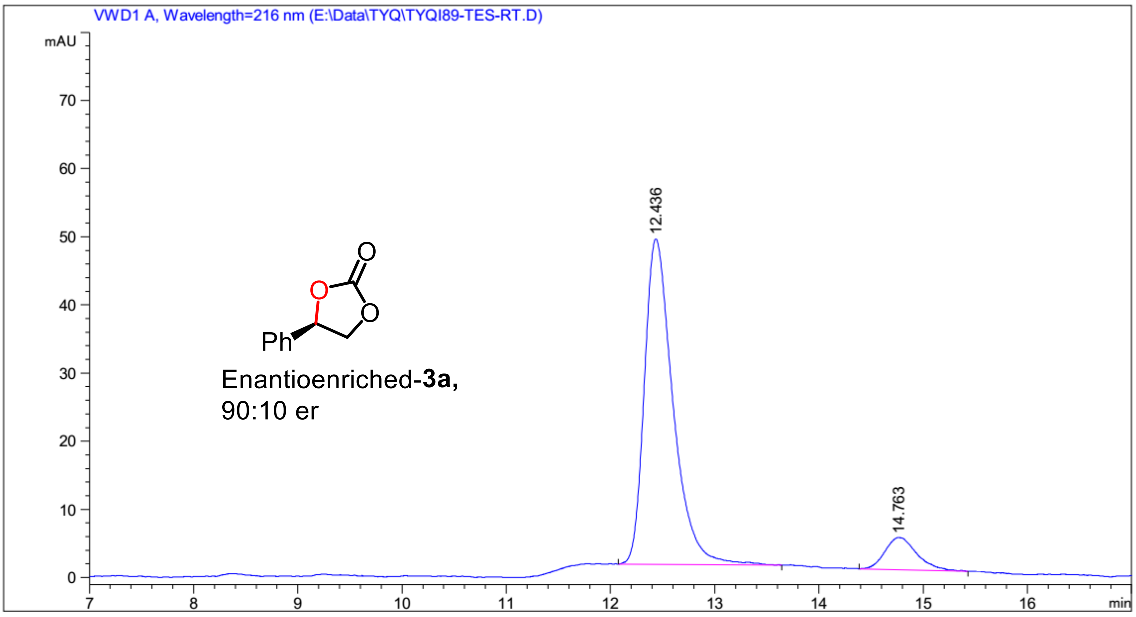

Peak #	RetTime [min]	Type	Width [min]	Area [mAU*s]	Height [mAU]	Area %
1	12.436	BV R	0.2851	908.63745	47.72469	89.9891
2	14.763	BV R	0.2820	101.08231	4.71784	10.0109

Figure S1. HPLC traces of *rac*-**3a** (reference) and enantioenriched-**3a**.

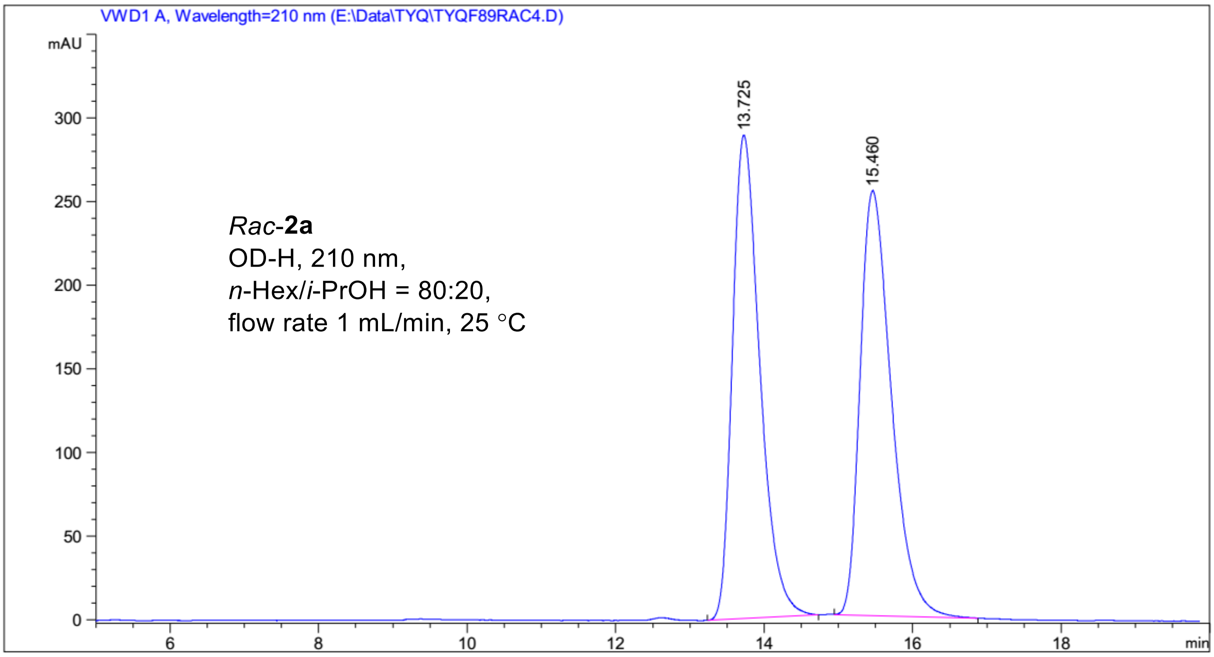

Peak #	RetTime [min]	Type	Width [min]	Area [mAU*s]	Height [mAU]	Area %
1	13.725	BB	0.3928	7401.32959	288.97668	49.9284
2	15.460	MM R	0.4873	7422.56006	253.87312	50.0716

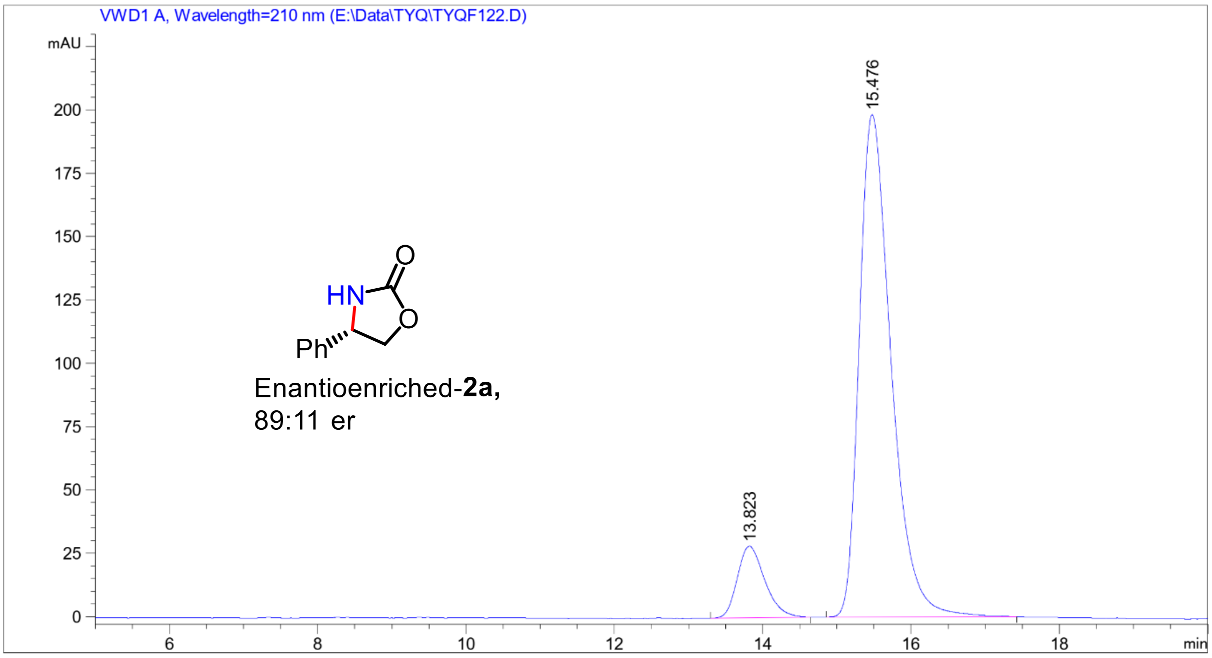

Peak #	RetTime [min]	Type	Width [min]	Area [mAU*s]	Height [mAU]	Area %
1	13.823	MM R	0.4307	732.28986	28.33900	10.9948
2	15.476	MM R	0.4980	5928.01465	198.38997	89.0052

Figure S2. HPLC traces of *rac*-**2a** (reference) and enantioenriched-**2a**.

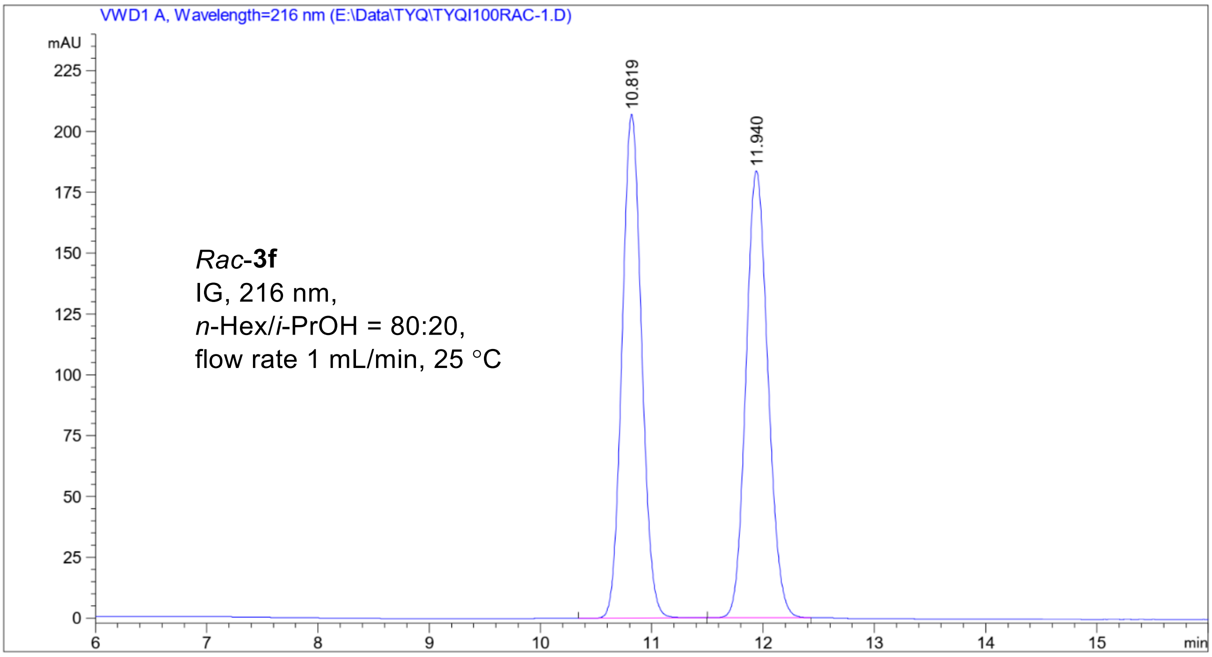

Peak #	RetTime [min]	Type	Width [min]	Area [mAU*s]	Height [mAU]	Area %
1	10.819	BB	0.1902	2522.97241	207.02525	50.0420
2	11.940	BB	0.2141	2518.73438	183.55788	49.9580

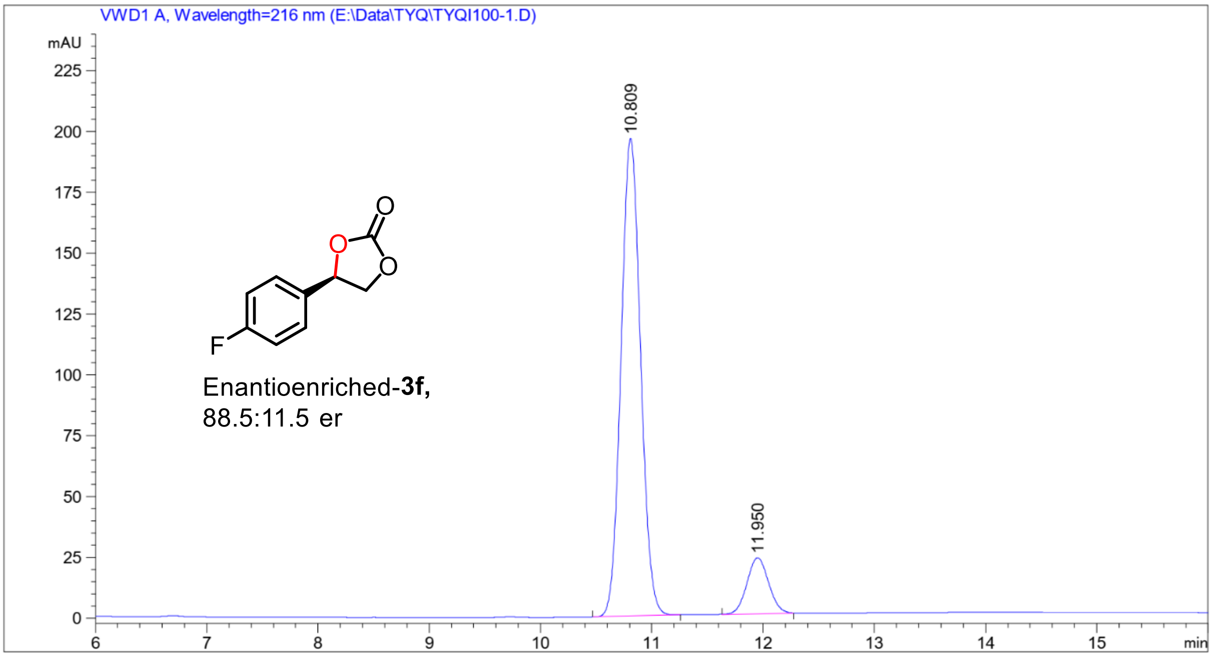

Peak #	RetTime [min]	Type	Width [min]	Area [mAU*s]	Height [mAU]	Area %
1	10.809	BB	0.1901	2388.54199	196.15846	88.4579
2	11.950	MM R	0.2257	311.65979	23.01066	11.5421

Figure S3. HPLC traces of *rac*-**3f** (reference) and enantioenriched-**3f**.

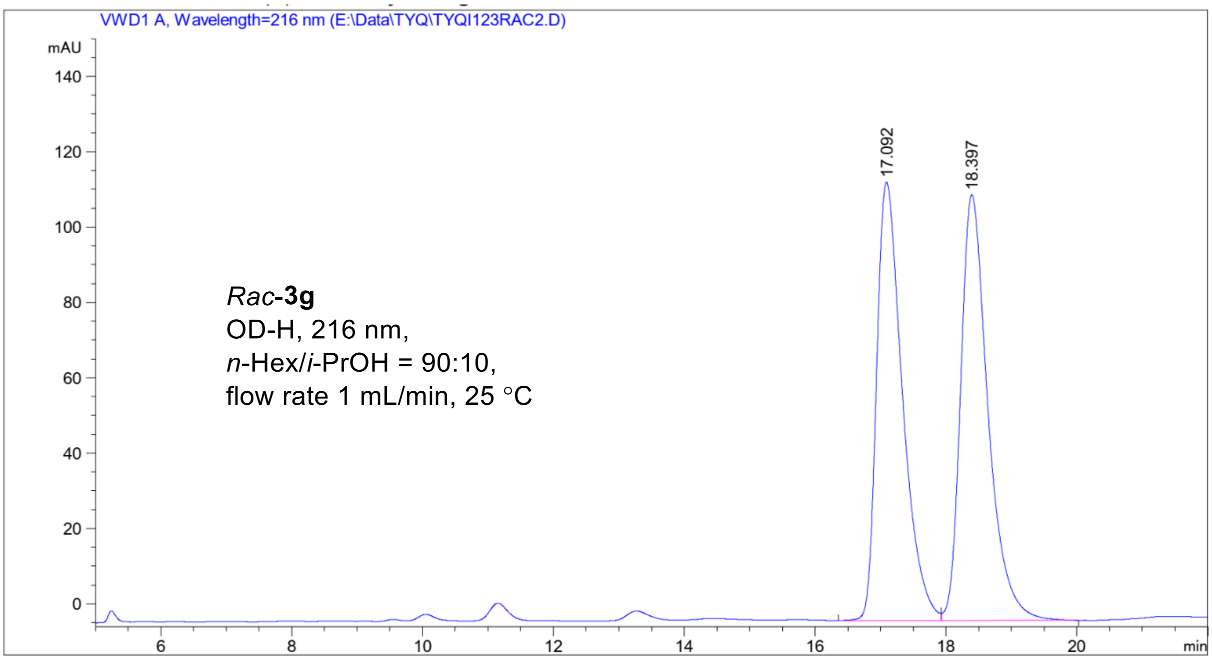

Peak #	RetTime [min]	Type	Width [min]	Area [mAU*s]	Height [mAU]	Area %
1	17.092	BV	0.4179	3190.42725	116.35553	49.3543
2	18.397	VB	0.4382	3273.90479	112.94375	50.6457

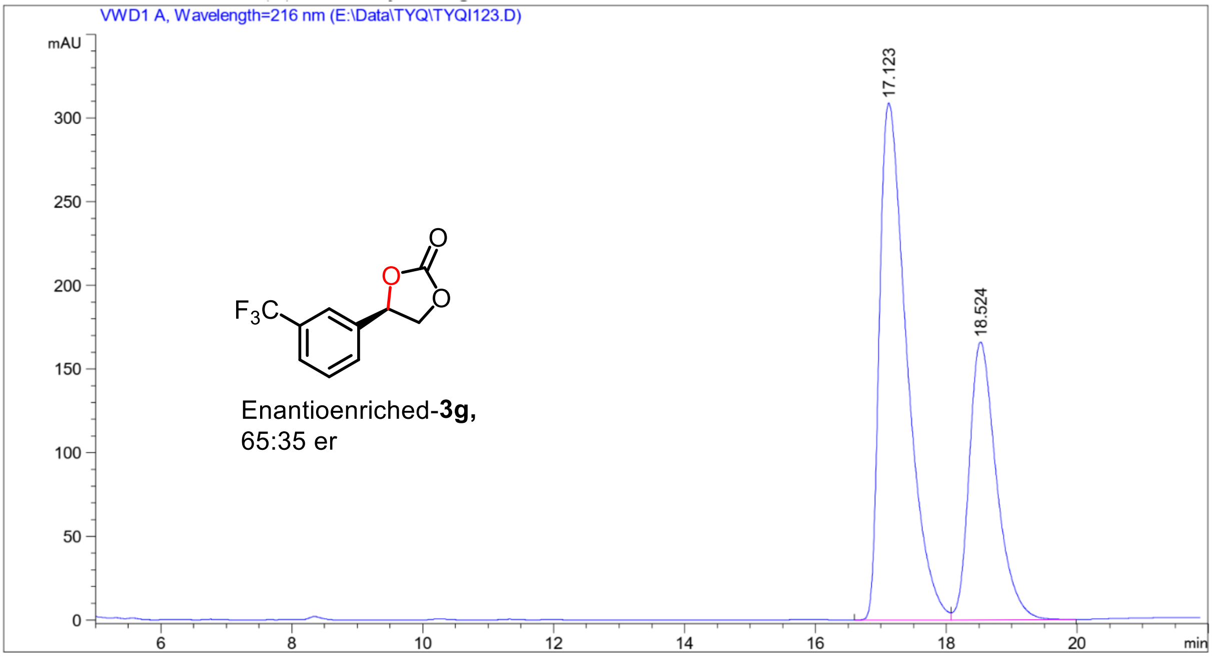

Peak #	RetTime [min]	Type	Width [min]	Area [mAU*s]	Height [mAU]	Area %
1	17.123	BV	0.4424	8983.92090	308.81171	65.1972
2	18.524	VB	0.4391	4795.68555	165.97446	34.8028

Figure S4. HPLC traces of *rac*-**3g** (reference) and enantioenriched-**3g**.

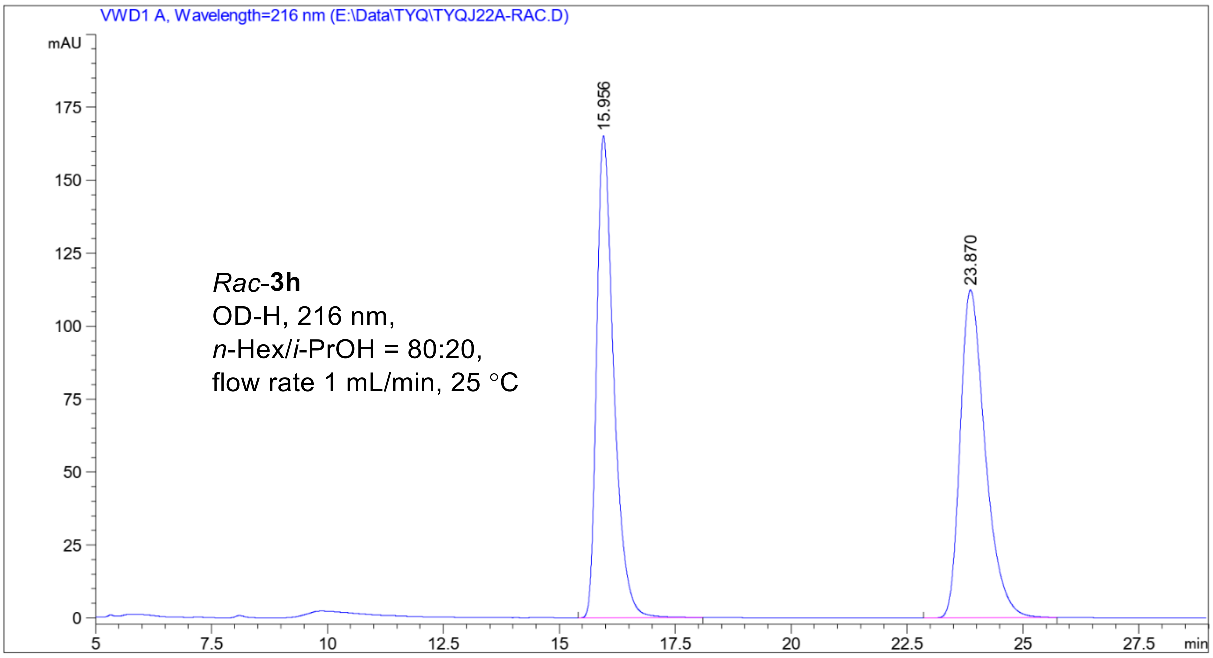

Peak #	RetTime [min]	Type	Width [min]	Area [mAU*s]	Height [mAU]	Area %
1	15.956	BB	0.3881	4235.36133	165.19463	50.0605
2	23.870	BB	0.5727	4225.12451	112.34937	49.9395

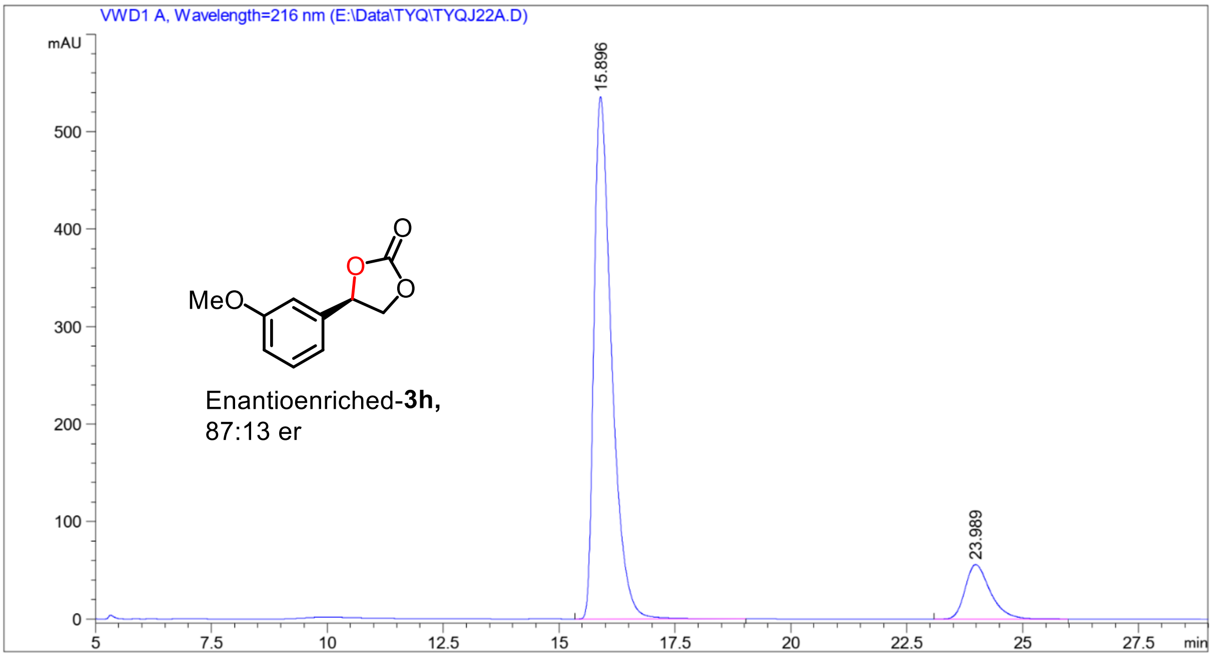

Peak #	RetTime [min]	Type	Width [min]	Area [mAU*s]	Height [mAU]	Area %
1	15.896	BB	0.4017	1.42188e4	535.58521	87.1059
2	23.989	BB	0.5702	2104.77026	55.78346	12.8941

Figure S5. HPLC traces of *rac*-**3h** (reference) and enantioenriched-**3h**.

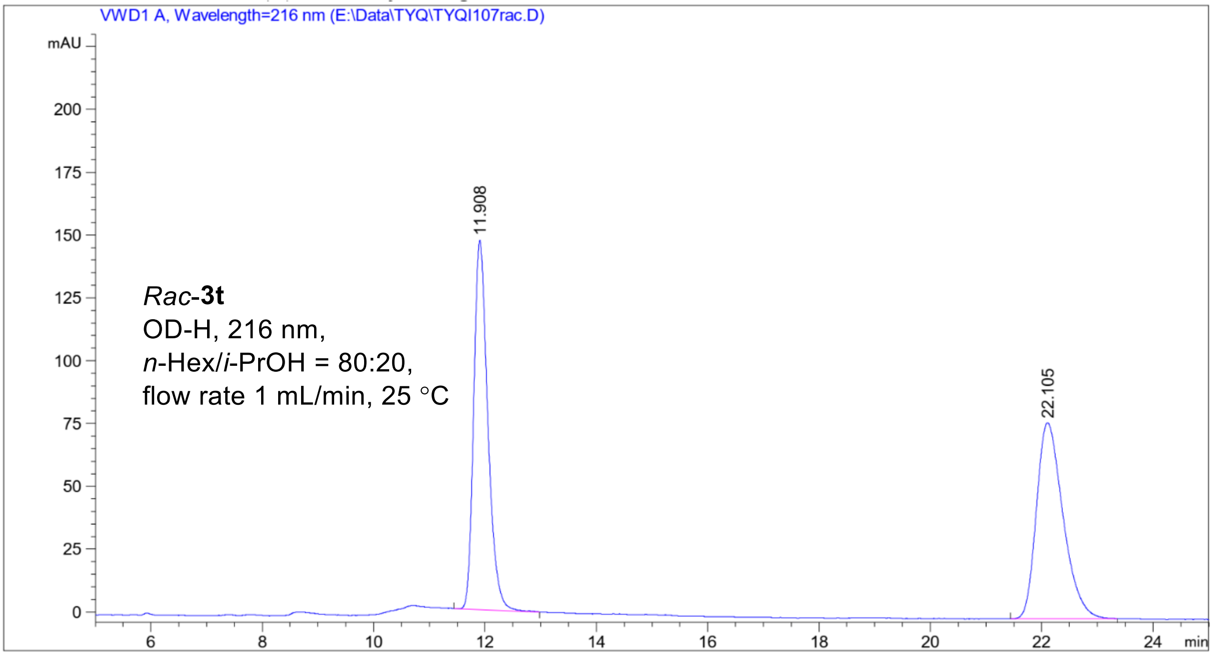

Peak #	RetTime [min]	Type	Width [min]	Area [mAU*s]	Height [mAU]	Area %
1	11.908	VV R	0.2681	2583.26660	146.97606	49.9365
2	22.105	BV R	0.5022	2589.83594	78.03764	50.0635

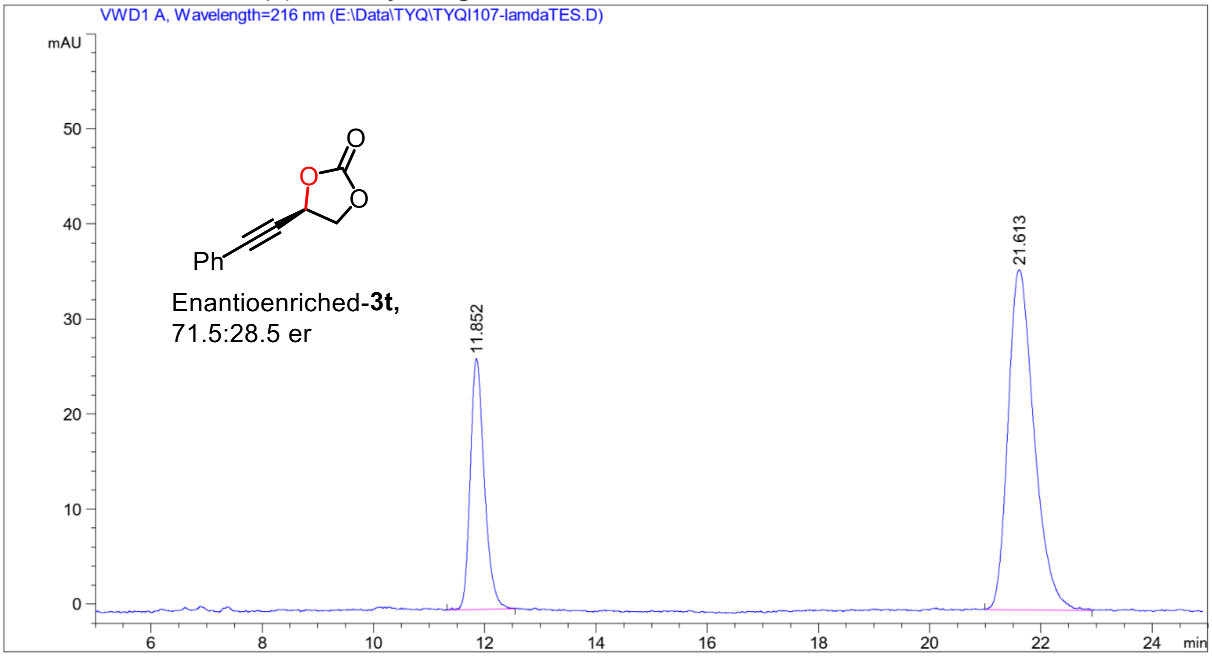

Peak #	RetTime [min]	Type	Width [min]	Area [mAU*s]	Height [mAU]	Area %
1	11.852	VB R	0.2639	463.60202	26.39375	28.4179
2	21.613	BV R	0.4887	1167.76904	35.78291	71.5821

Figure S6. HPLC traces of *rac*-**3t** (reference) and enantioenriched-**3t**.

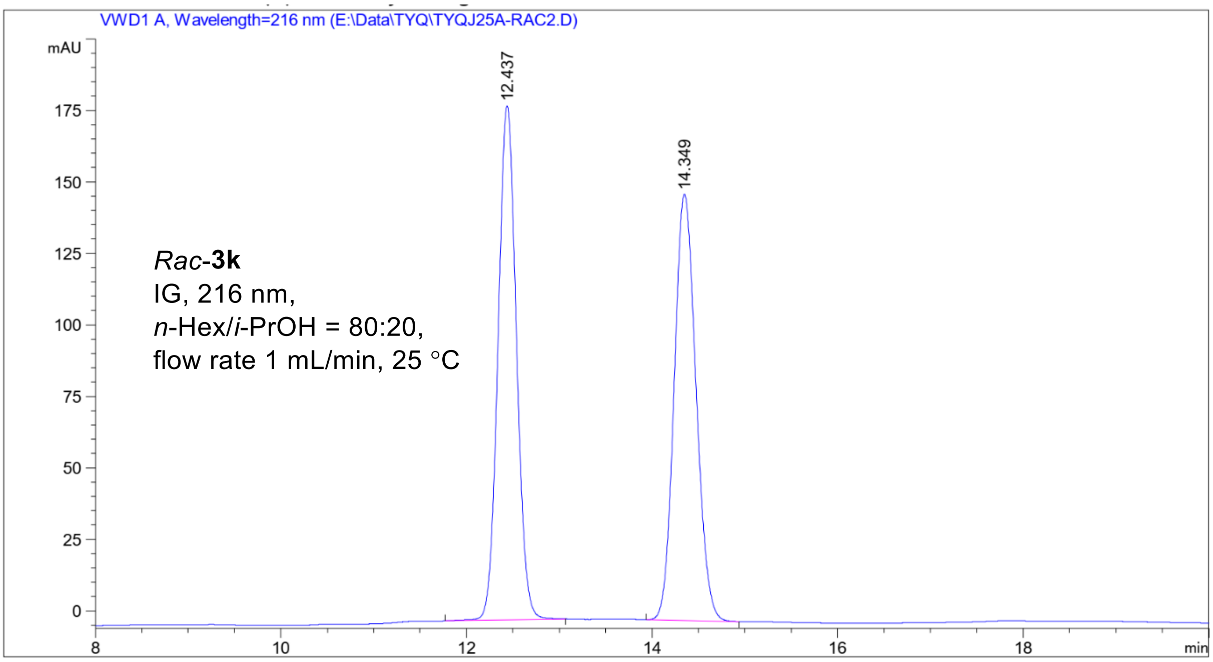

Peak #	RetTime [min]	Type	Width [min]	Area [mAU*s]	Height [mAU]	Area %
1	12.437	MM R	0.2293	2472.07544	179.67522	50.3921
2	14.349	BB	0.2545	2433.60425	149.02390	49.6079

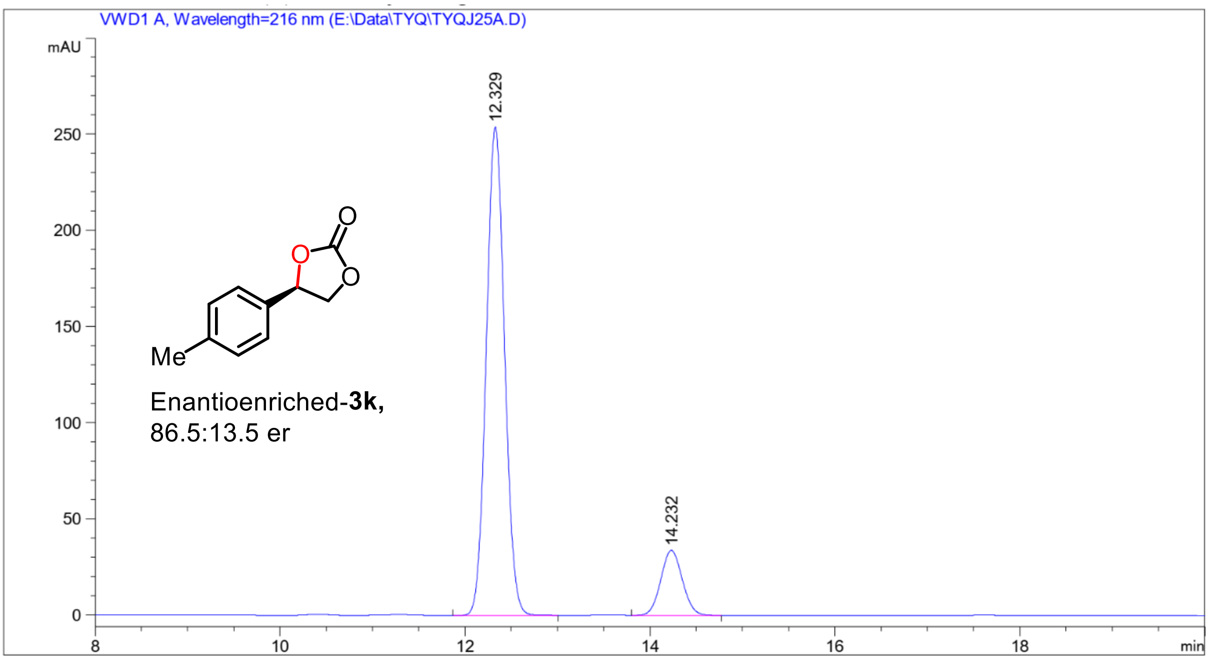

Peak #	RetTime [min]	Type	Width [min]	Area [mAU*s]	Height [mAU]	Area %
1	12.329	BB	0.2141	3484.61938	254.00301	86.4649
2	14.232	BB	0.2523	545.47717	33.78783	13.5351

Figure S7. HPLC traces of *rac*-**3k** (reference) and enantioenriched-**3k**.

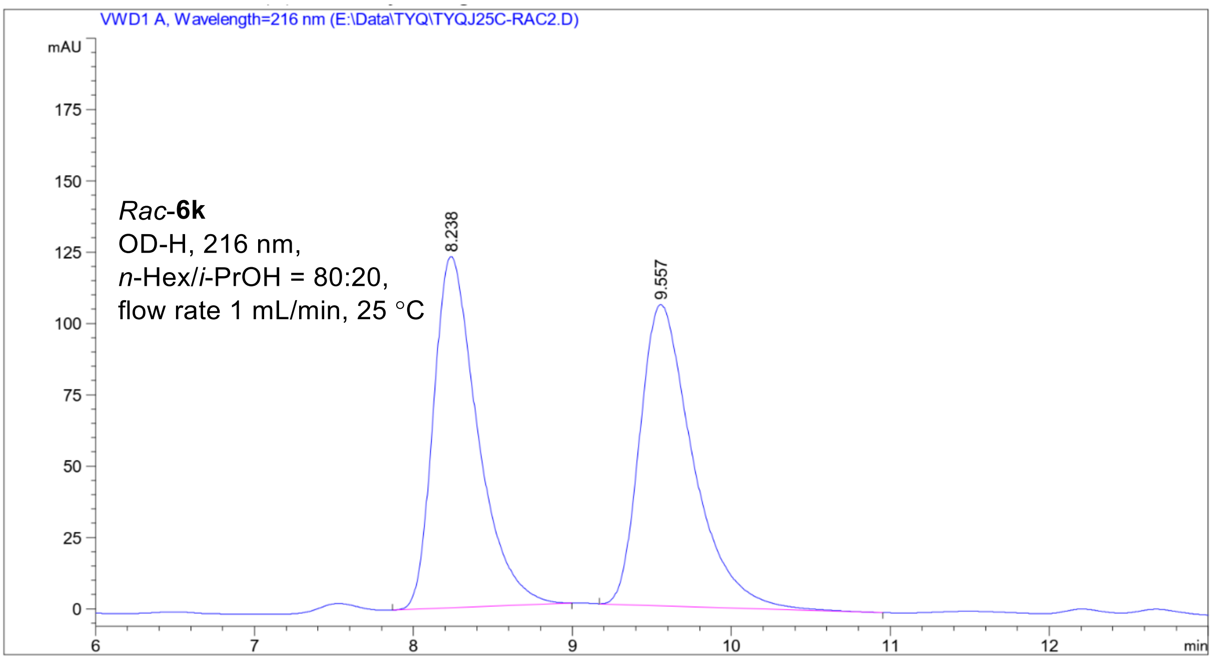

Peak #	RetTime [min]	Type	Width [min]	Area [mAU*s]	Height [mAU]	Area %
1	8.238	BB	0.2918	2369.72485	122.99841	49.2116
2	9.557	BB	0.3504	2445.65576	105.51378	50.7884

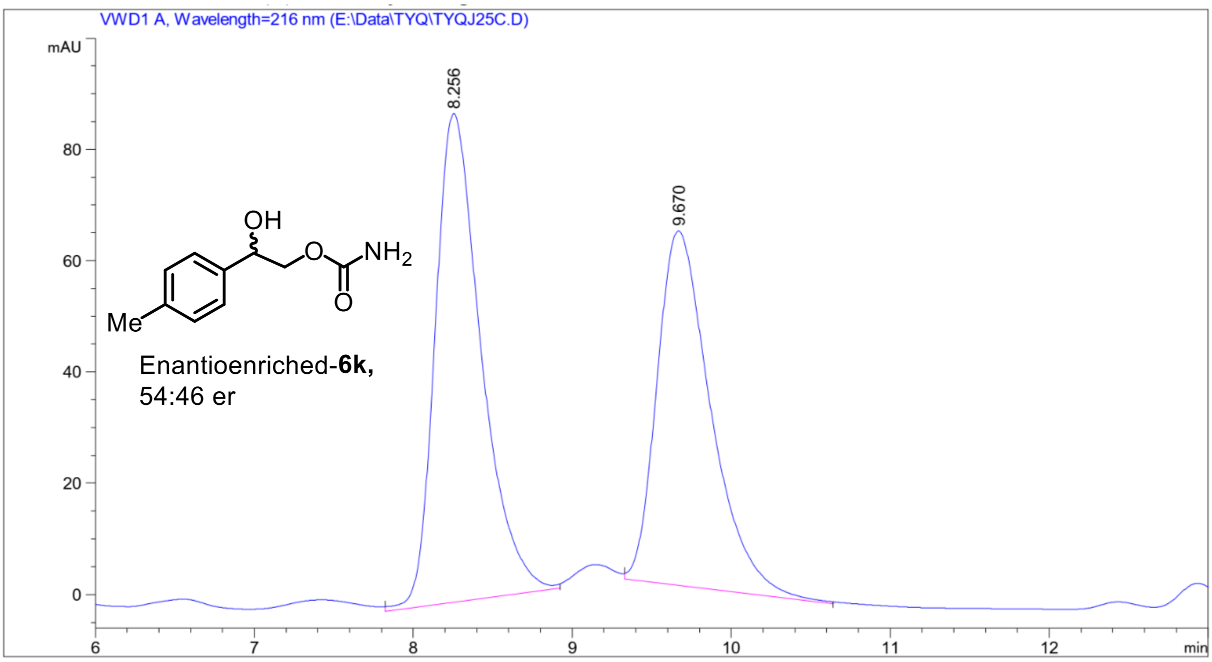

Peak #	RetTime [min]	Type	Width [min]	Area [mAU*s]	Height [mAU]	Area %
1	8.256	MM R	0.3356	1768.68005	87.84589	54.1318
2	9.670	MM R	0.3923	1498.67664	63.67462	45.8682

Figure S8. HPLC traces of *rac*-**6k** (reference) and enantioenriched-**6k**.

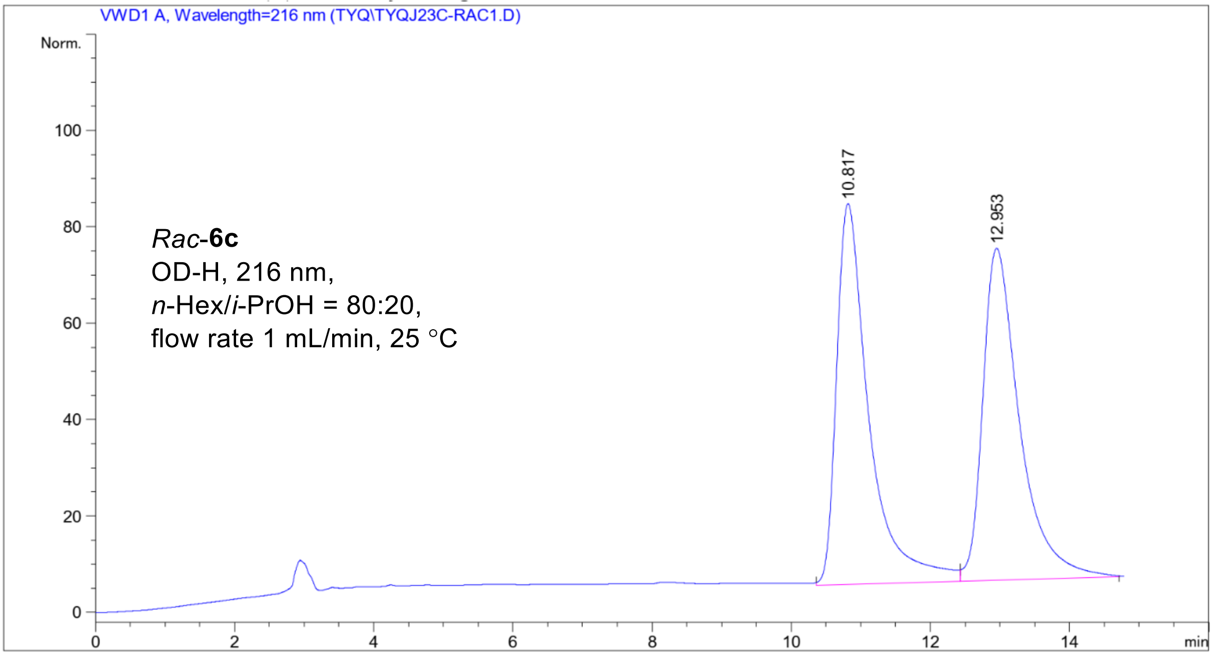

Peak #	RetTime [min]	Type	Width [min]	Area mAU *s	Height [mAU]	Area %
1	10.817	MF R	0.5413	2566.31470	79.01414	50.2346
2	12.953	FM R	0.6149	2542.34863	68.90549	49.7654

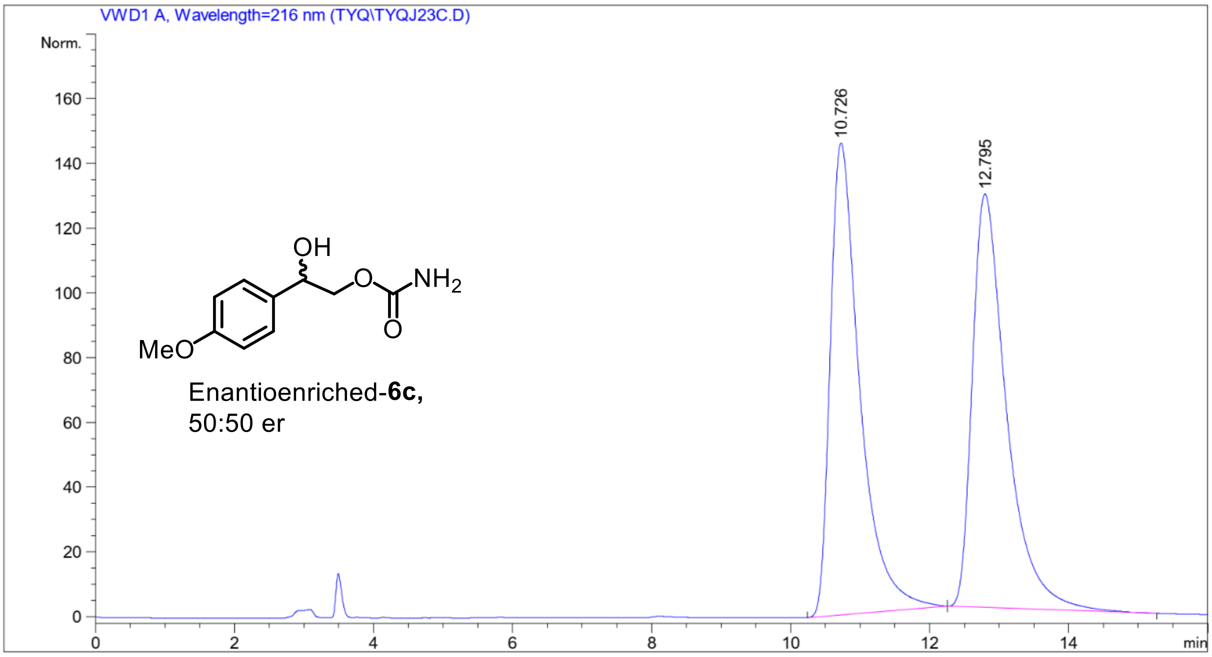

Peak #	RetTime [min]	Type	Width [min]	Area mAU *s	Height [mAU]	Area %
1	10.726	BB	0.4405	4302.34912	145.74687	49.6096
2	12.795	BBA	0.5155	4370.07178	127.75885	50.3904

Figure S9. HPLC traces of *rac*-**6c** (reference) and enantioenriched-**6c**.

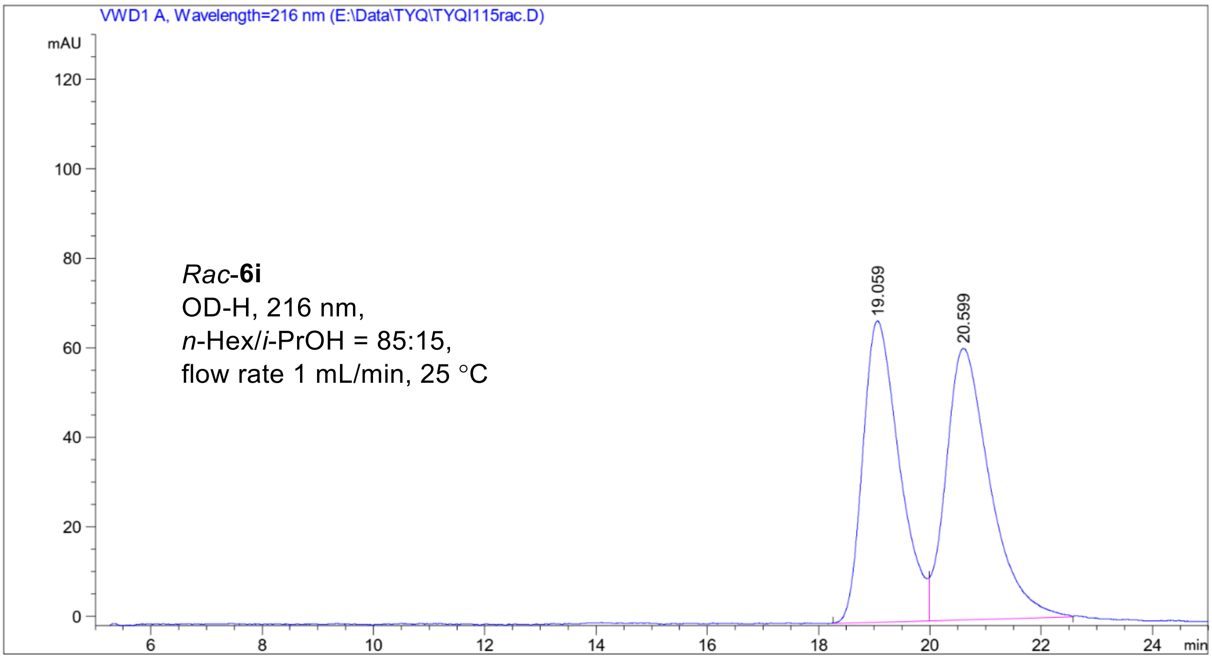

Peak #	RetTime [min]	Type	Width [min]	Area [mAU*s]	Height [mAU]	Area %
1	19.059	MF R	0.7619	3077.87671	67.32693	48.0912
2	20.599	FM R	0.9138	3322.20581	60.59423	51.9088

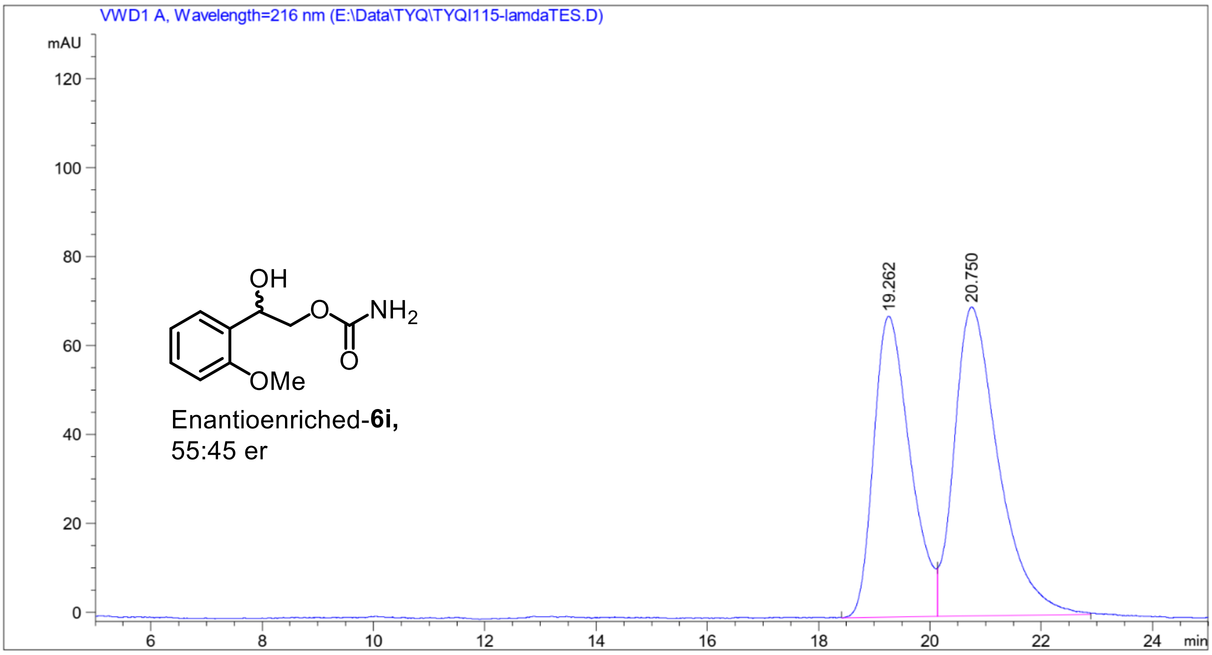

Peak #	RetTime [min]	Type	Width [min]	Area [mAU*s]	Height [mAU]	Area %
1	19.262	MF R	0.7609	3089.71411	67.67490	44.6146
2	20.750	FM R	0.9208	3835.62720	69.42342	55.3854

Figure S10. HPLC traces of *rac*-**6i** (reference) and enantioenriched-**6i**.

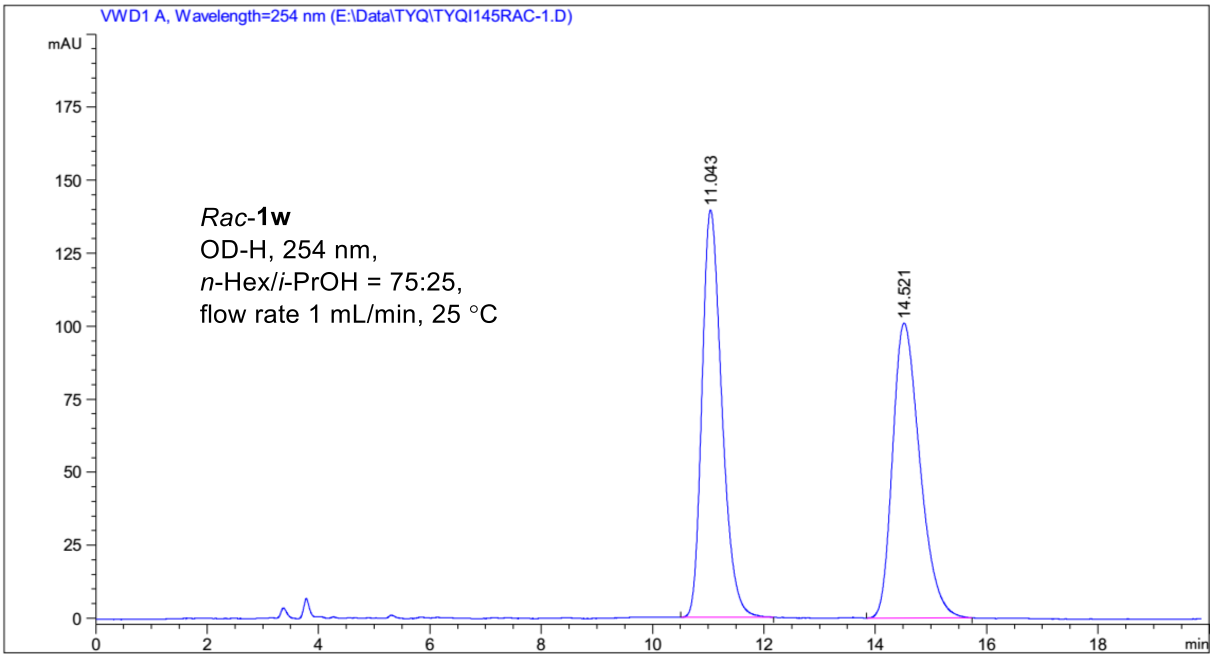

Peak #	RetTime [min]	Type	Width [min]	Area [mAU*s]	Height [mAU]	Area %
1	11.043	MM R	0.4135	3460.00391	139.46498	50.1540
2	14.521	BB	0.5152	3438.76001	100.99265	49.8460

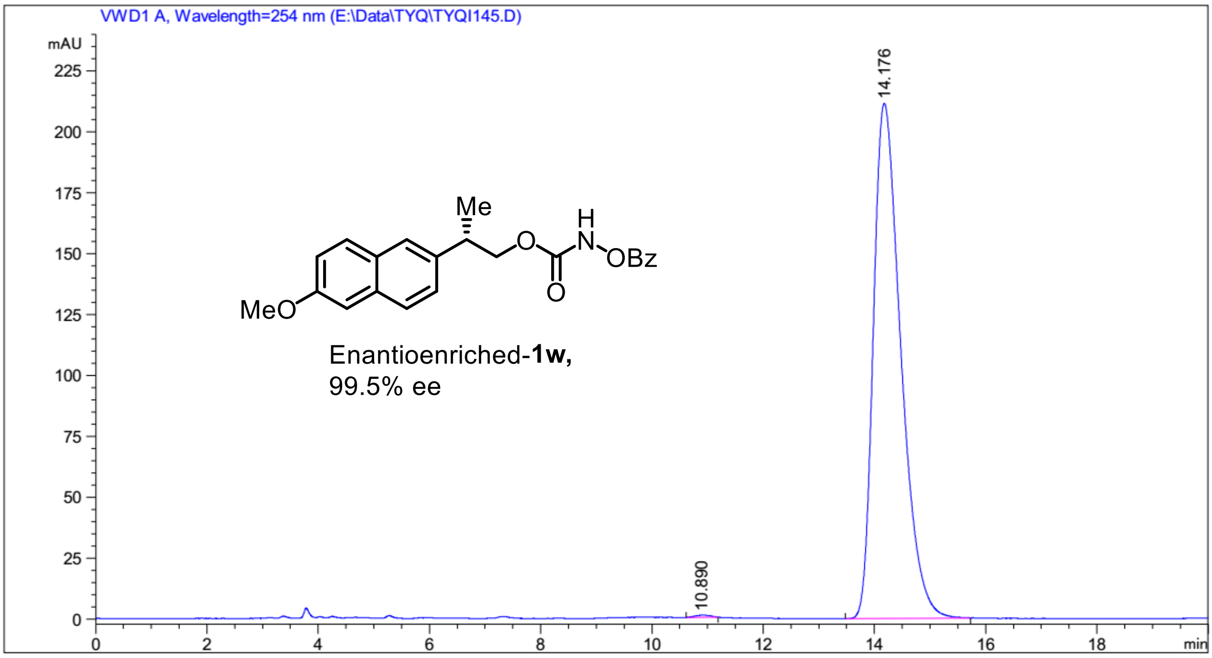

Peak #	RetTime [min]	Type	Width [min]	Area [mAU*s]	Height [mAU]	Area %
1	10.890	MM R	0.3234	17.76672	9.15667e-1	0.2455
2	14.176	BB	0.5246	7220.18799	211.34663	99.7545

Figure S11. HPLC traces of *rac*-**1w** (reference) and enantioenriched-**1w**.

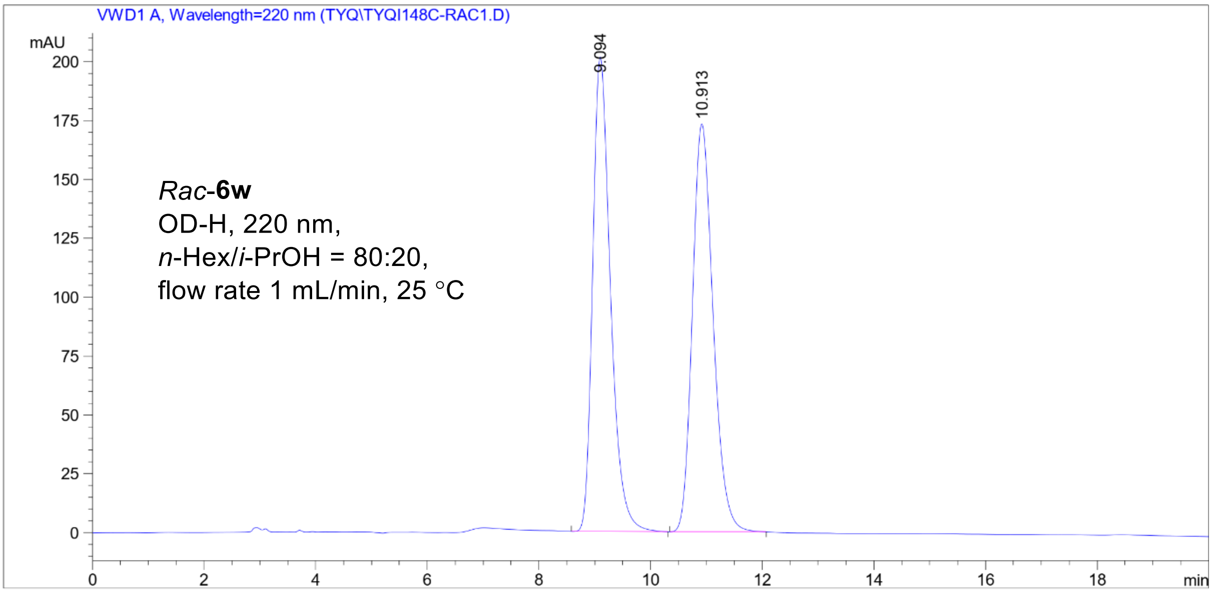

Peak #	RetTime [min]	Type	Width [min]	Area mAU *s	Height [mAU]	Area %
1	9.094	MM R	0.3695	4468.35400	201.56075	50.0320
2	10.913	MM R	0.4293	4462.64014	173.24644	49.9680

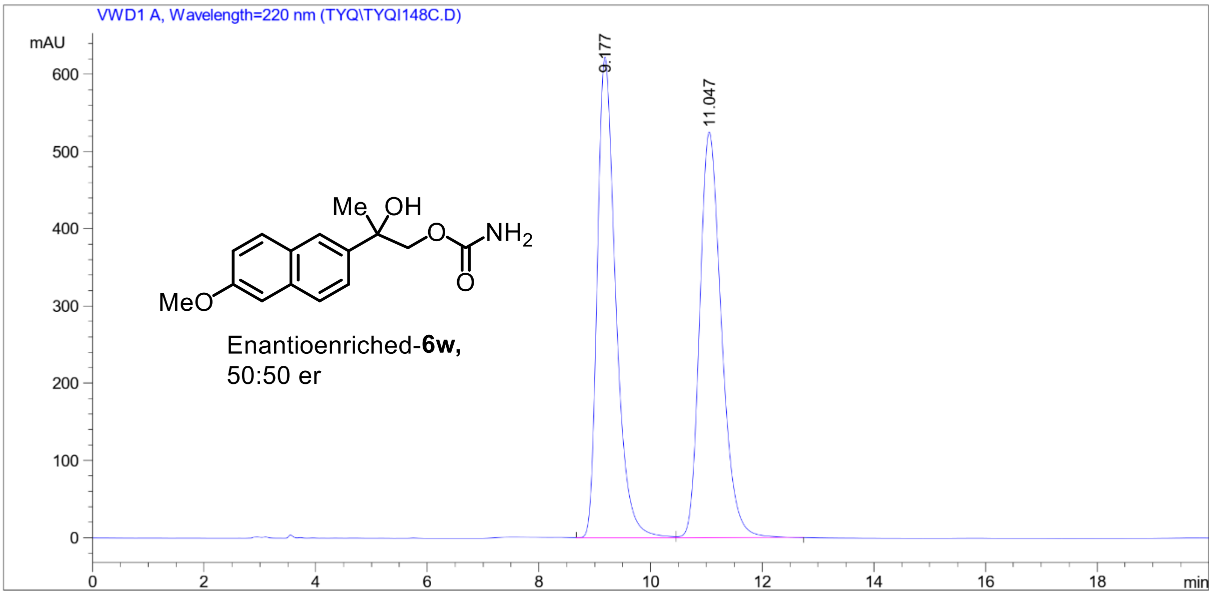

Peak #	RetTime [min]	Type	Width [min]	Area mAU *s	Height [mAU]	Area %
1	9.177	VV	0.3511	1.43090e4	622.66840	50.0520
2	11.047	VB	0.4174	1.42793e4	525.55304	49.9480

Figure S12. HPLC traces of *rac*-**6w** (reference) and enantioenriched-**6w**.

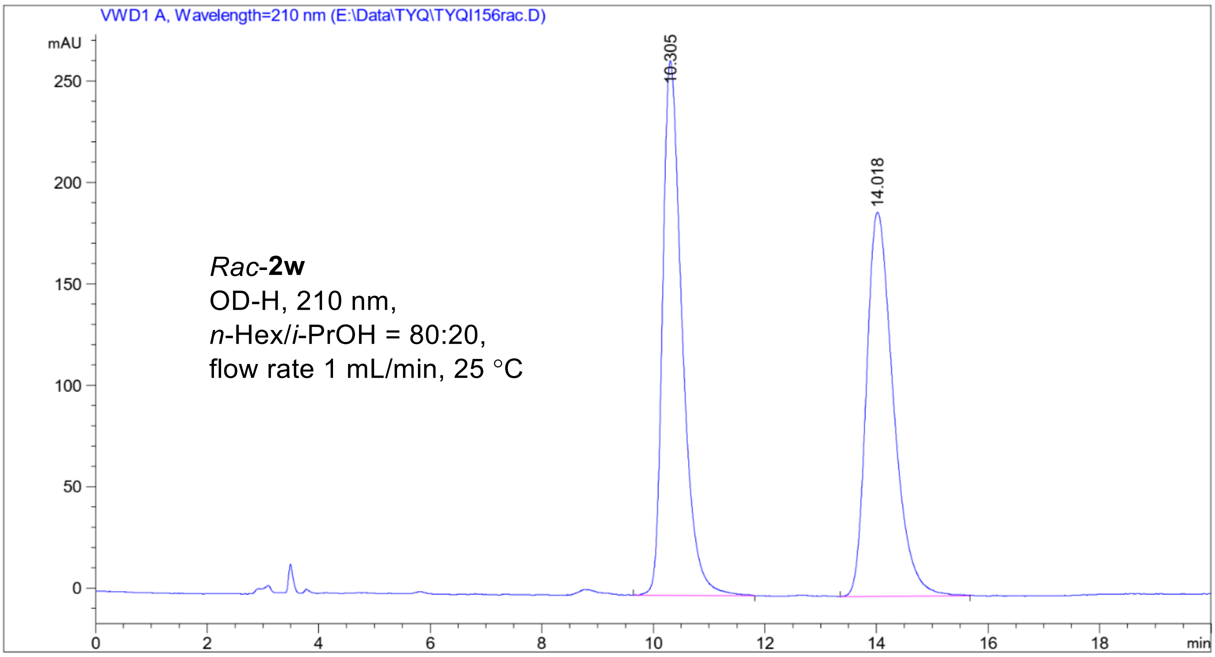

Peak #	RetTime [min]	Type	Width [min]	Area [mAU*s]	Height [mAU]	Area %
1	10.305	VV R	0.3693	6418.44141	263.39984	50.3623
2	14.018	BV R	0.5121	6326.09961	189.20157	49.6377

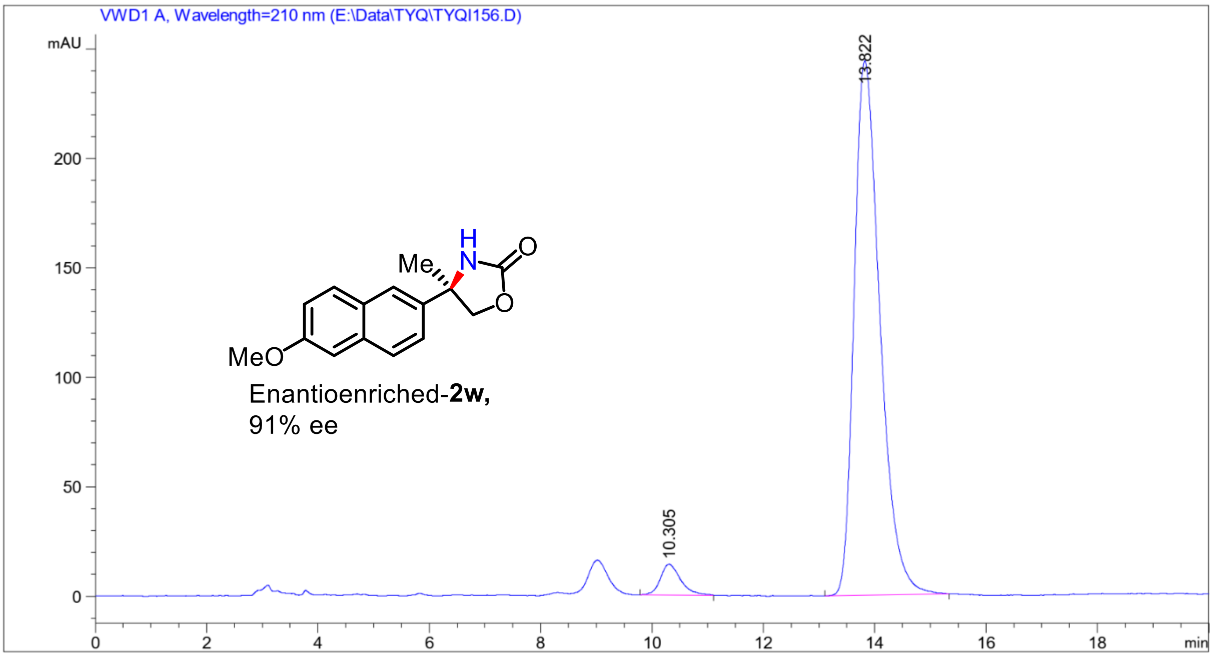

Peak #	RetTime [min]	Type	Width [min]	Area [mAU*s]	Height [mAU]	Area %
1	10.305	VV R	0.3926	355.33084	14.07131	4.3141
2	13.822	BV R	0.4955	7881.23828	244.20801	95.6859

Figure S13. HPLC traces of *rac*-**2w** (reference) and enantioenriched-**2w**.

9. NMR Spectra

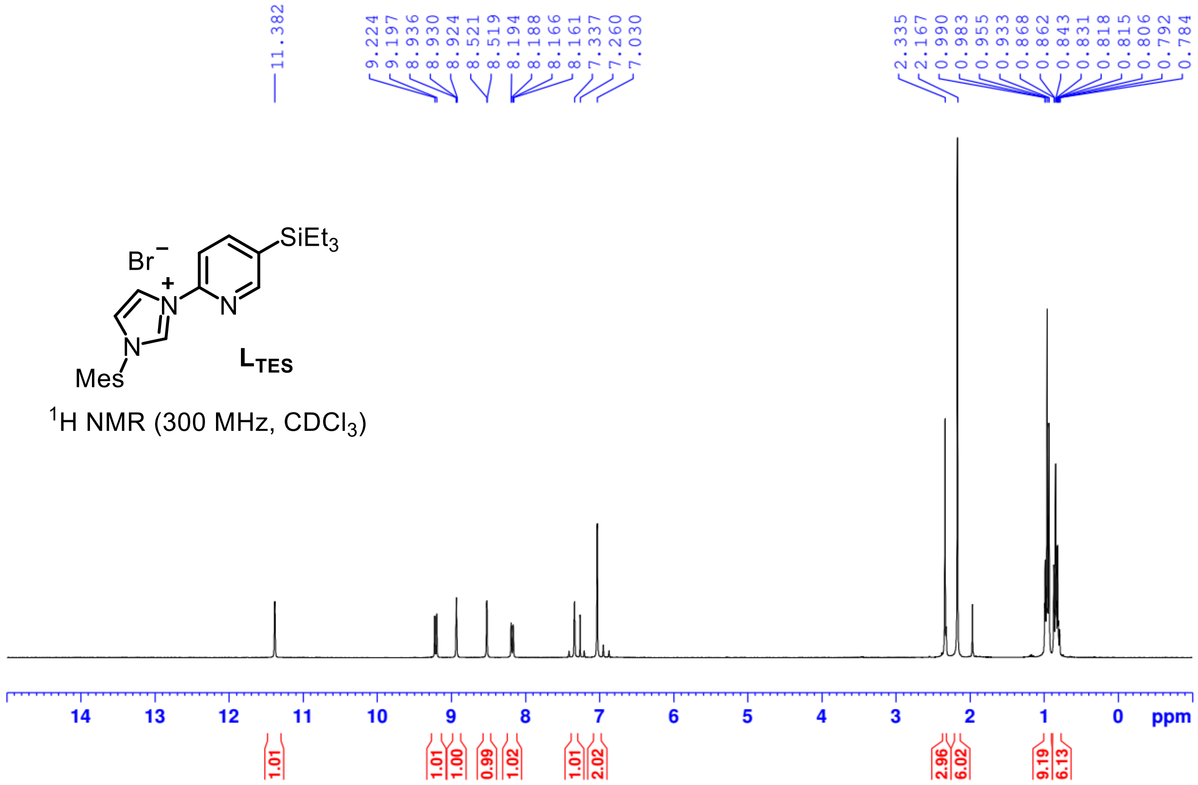

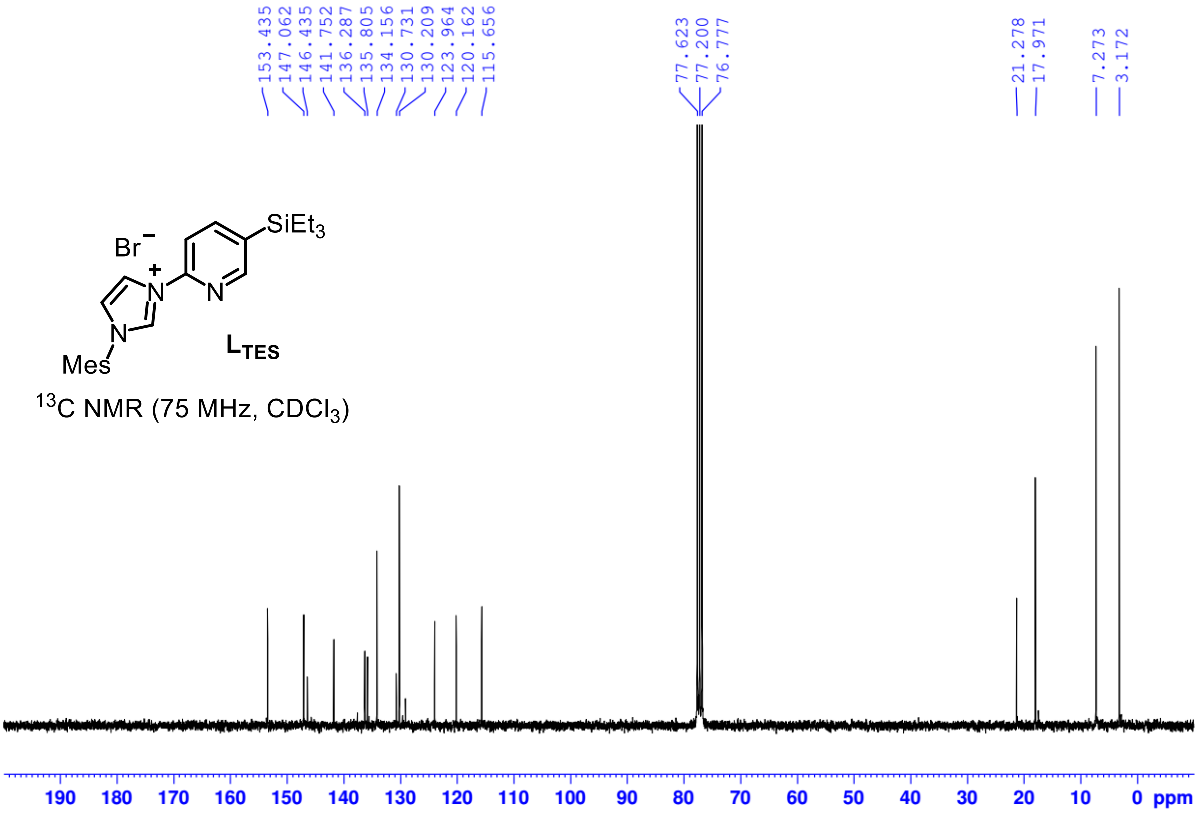

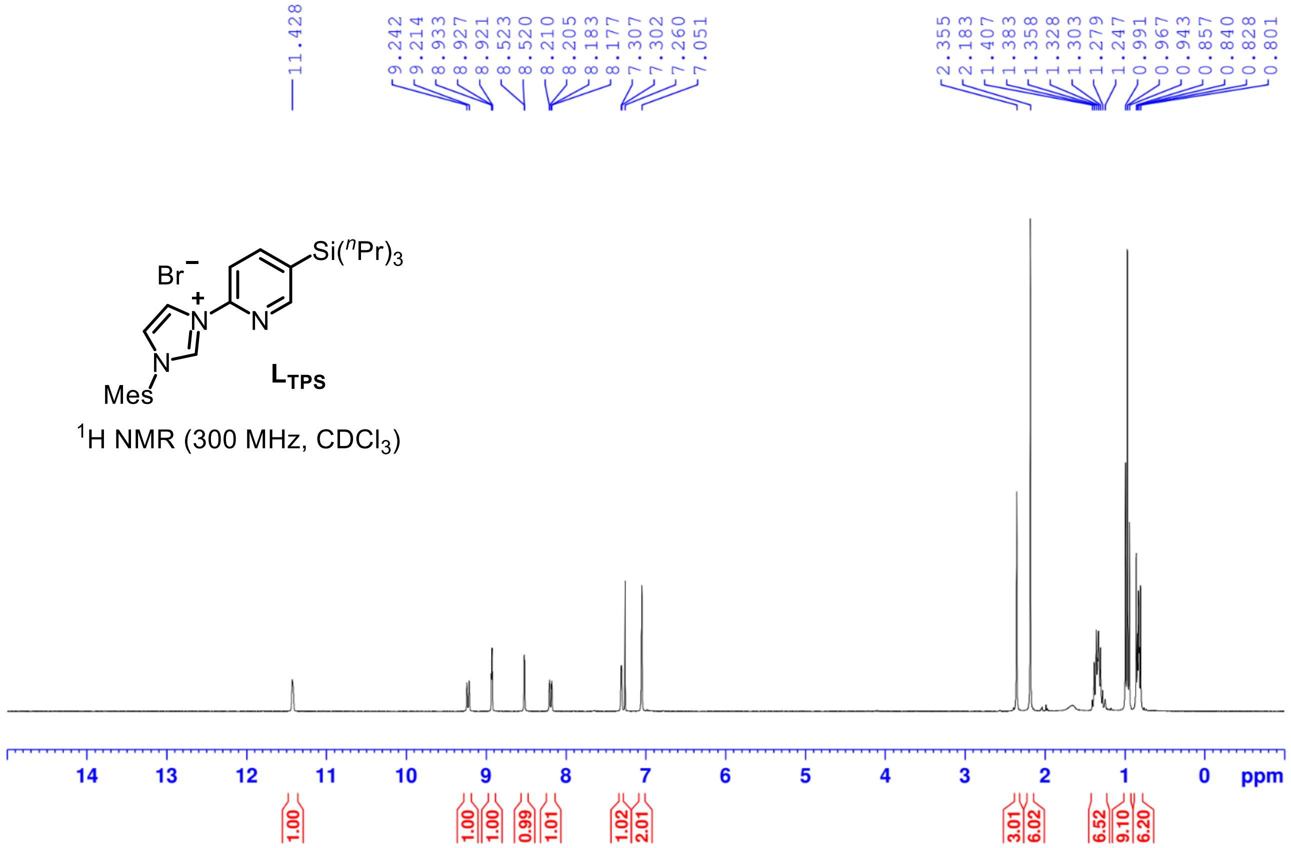
Br⁻
Si(ⁿPr)3
N
Mes
L_TPS
1H NMR (300 MHz, CDCl3)
11.428
9.242
9.214
8.933
8.927
8.921
8.523
8.520
8.210
8.205
8.183
8.177
7.307
7.302
7.260
7.051
2.355
2.183
1.407
1.383
1.358
1.328
1.303
1.279
1.247
0.991
0.967
0.943
0.857
0.840
0.828
0.801
1.00
1.00
1.00
0.99
1.01
1.02
2.01
3.01
6.02
6.52
9.10
6.20
ppm

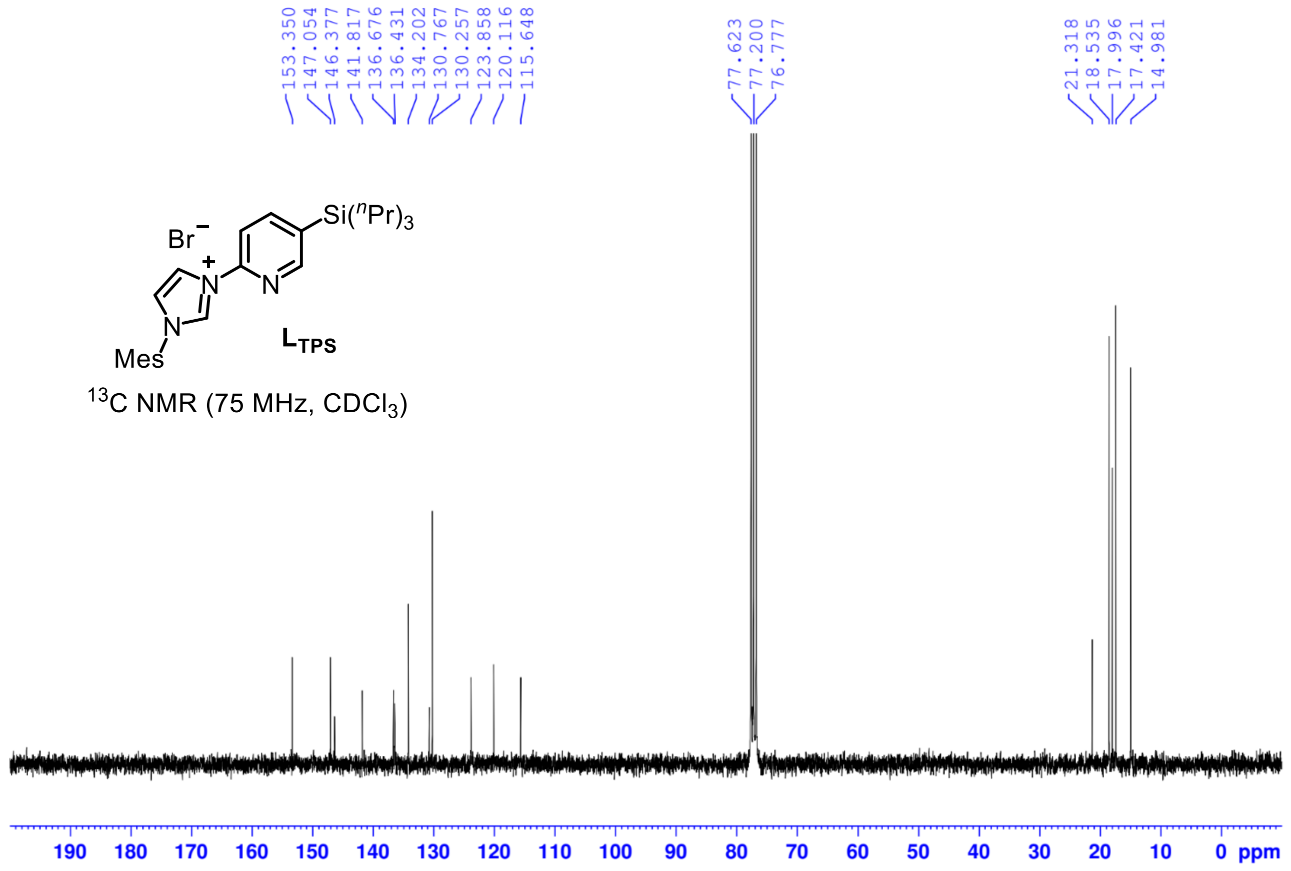
Br⁻
Si(ⁿPr)3
N
Mes
L_TPS
13C NMR (75 MHz, CDCl3)
153.350
147.054
146.377
141.817
136.676
136.431
134.202
130.767
130.257
123.858
120.116
115.648
77.623
77.200
76.777
21.318
18.535
17.996
17.421
14.981
ppm

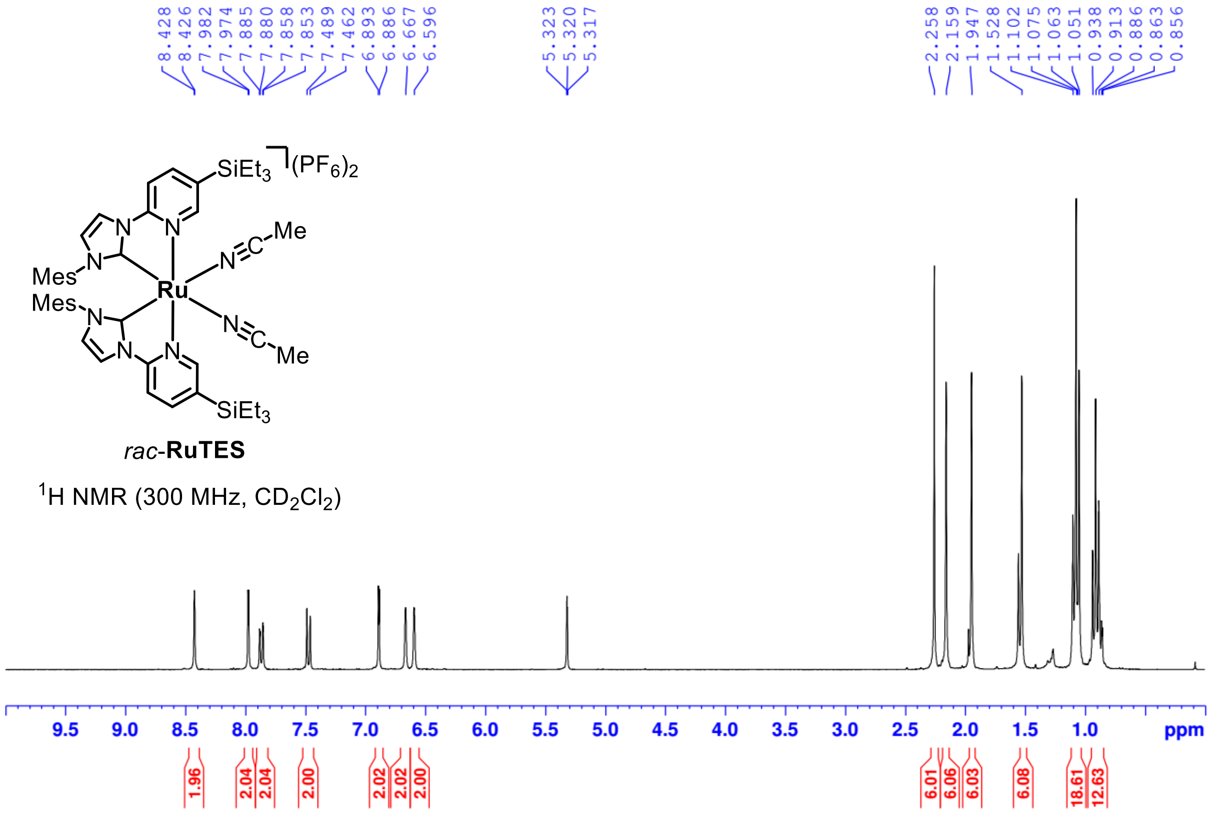
8.428 8.426 7.982 7.974 7.885 7.880 7.858 7.853 7.489 7.462 6.893 6.886 6.667 6.596
5.323 5.320 5.317
2.258 2.159 1.947 1.528 1.102 1.075 1.063 1.051 0.938 0.913 0.886 0.863 0.856
SiEt3 (PF6)2
Mes
Mes
Ru
N≡C–Me
SiEt3
rac-RuTES
1H NMR (300 MHz, CD2Cl2)
9.5 9.0 8.5 8.0 7.5 7.0 6.5 6.0 5.5 5.0 4.5 4.0 3.5 3.0 2.5 2.0 1.5 1.0 ppm
1.96 2.04 2.04 2.00 2.02 2.02 2.00 6.01 6.06 6.03 6.08 18.61 12.63

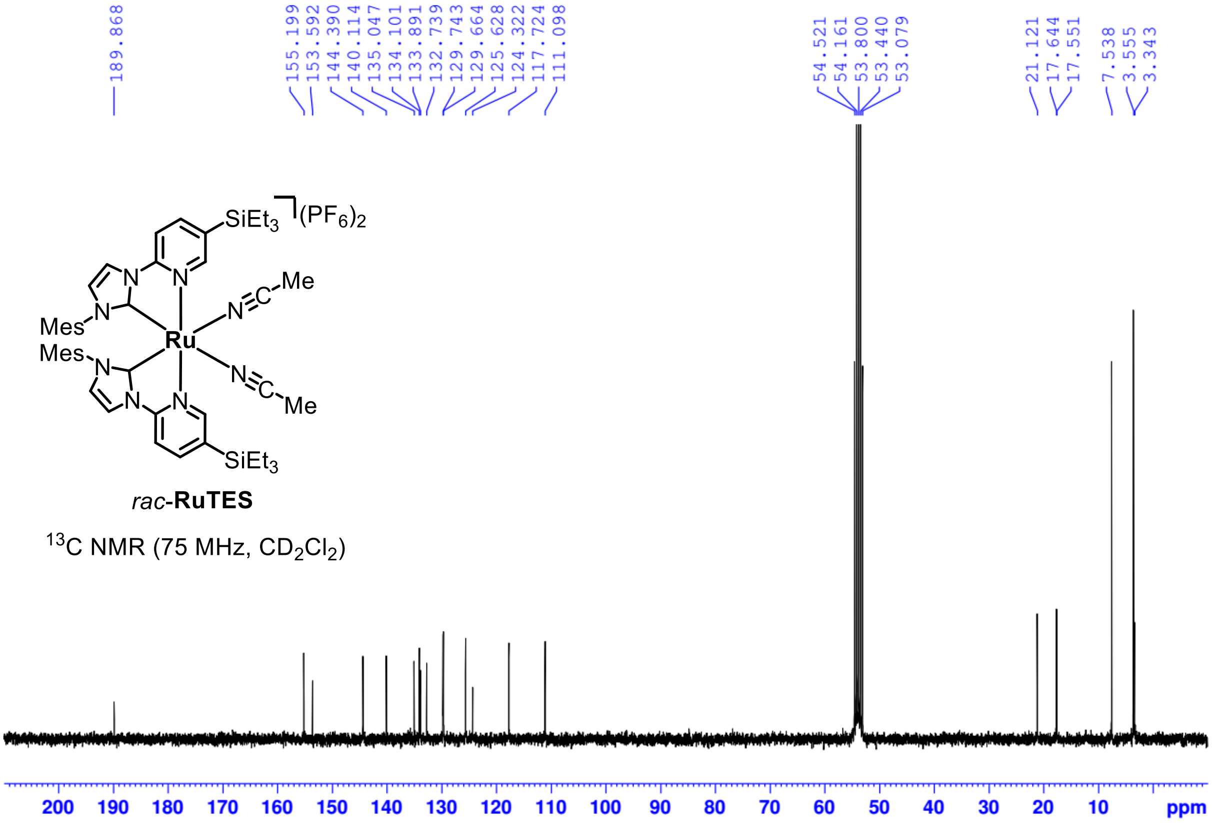
189.868
155.199 153.592 144.390 140.114 135.047 134.101 133.891 132.739 129.743 129.664 125.628 124.322 117.724 111.098
54.521 54.161 53.800 53.440 53.079
21.121 17.644 17.551 7.538 3.555 3.343
SiEt3 (PF6)2
Mes
Mes
Ru
N≡C–Me
SiEt3
rac-RuTES
13C NMR (75 MHz, CD2Cl2)
200 190 180 170 160 150 140 130 120 110 100 90 80 70 60 50 40 30 20 10 ppm

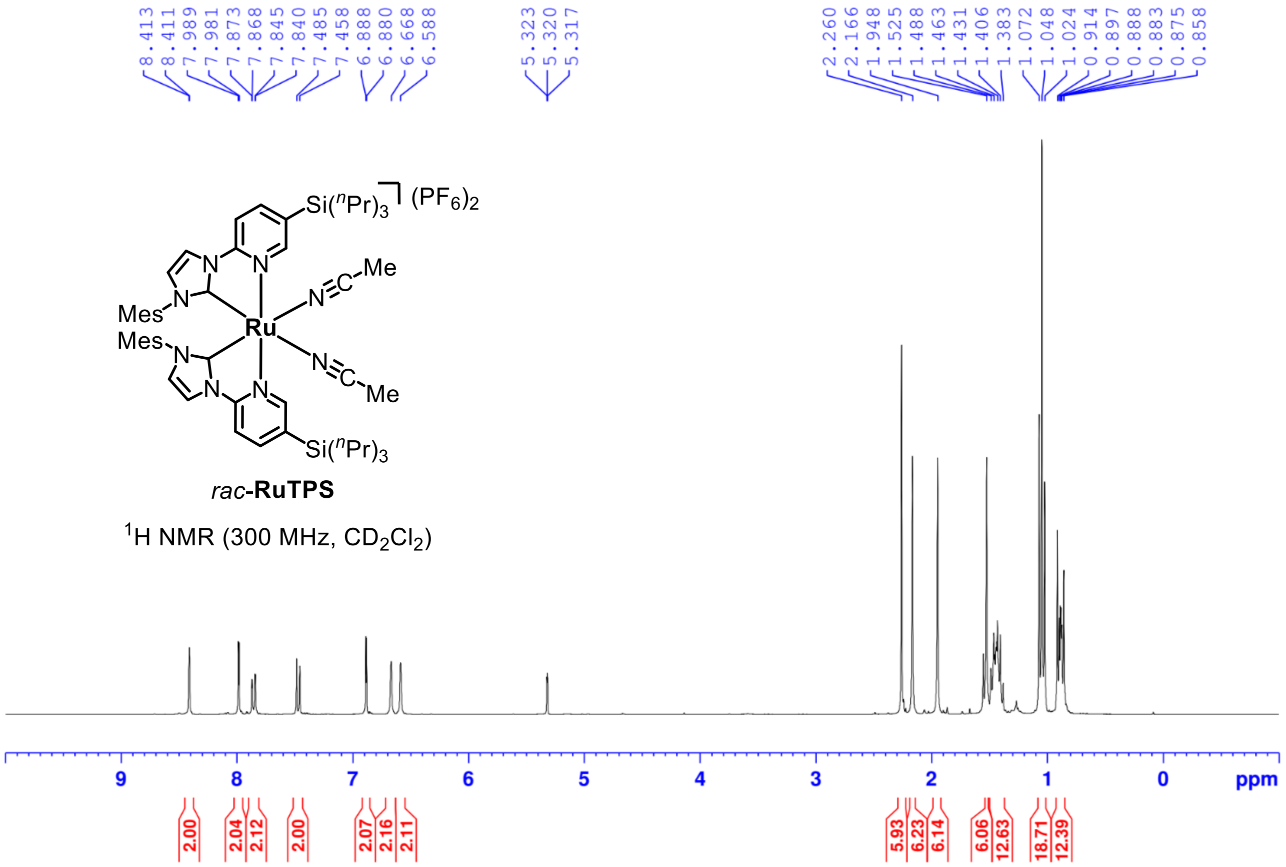
8.413 8.411 7.989 7.981 7.873 7.868 7.845 7.840 7.485 7.458 6.888 6.880 6.668 6.588
5.323 5.320 5.317
2.260 2.166 1.948 1.525 1.488 1.463 1.431 1.406 1.383 1.072 1.048 1.024 0.914 0.897 0.888 0.883 0.875 0.858
Si(nPr)3 (PF6)2
N≡C–Me
Mes
Ru
Si(nPr)3
rac-RuTPS
1H NMR (300 MHz, CD2Cl2)
9 8 7 6 5 4 3 2 1 0 ppm
2.00 2.04 2.12 2.00 2.07 2.16 2.11 5.93 6.23 6.14 6.06 12.63 18.71 12.39

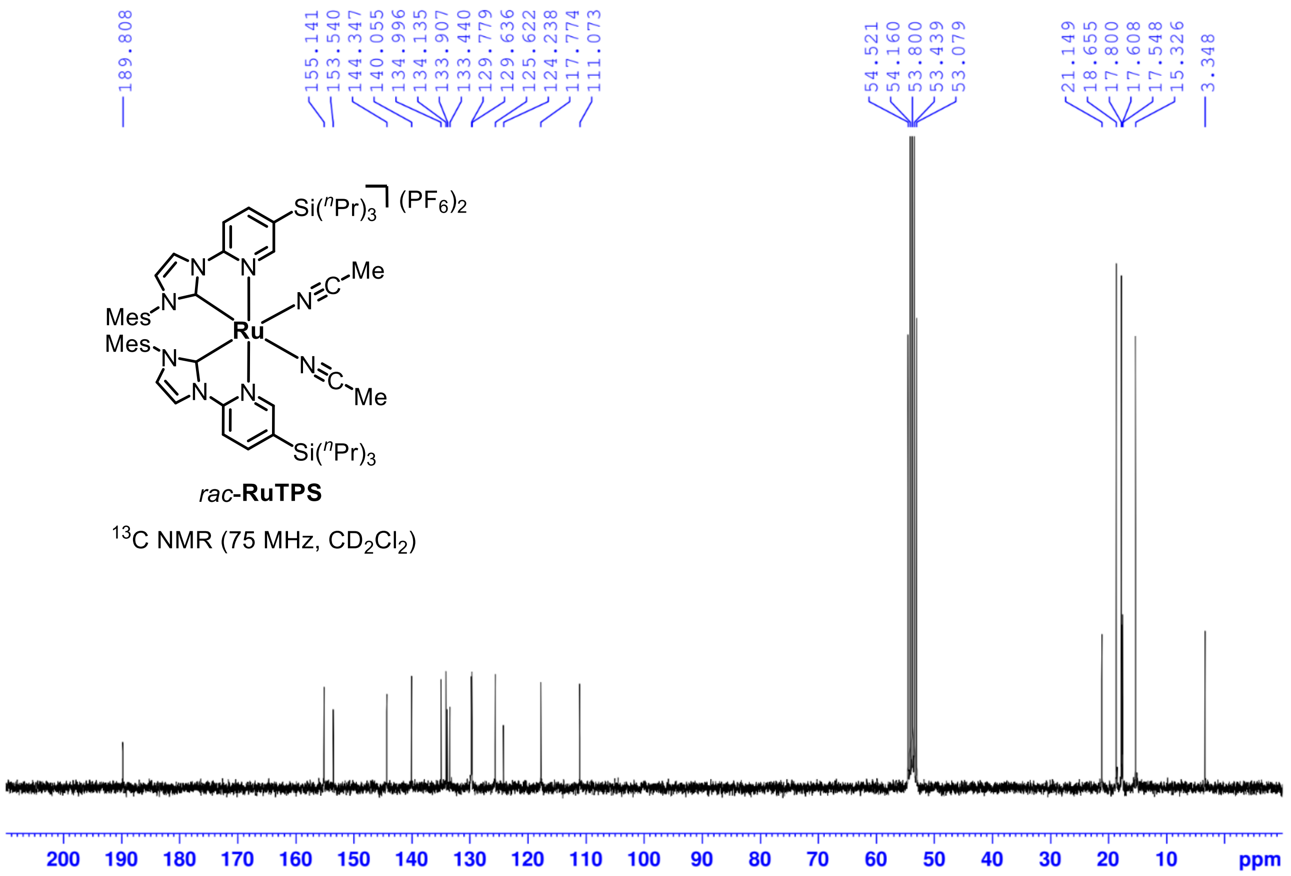
189.808
155.141 153.540 144.347 140.055 134.996 134.135 133.907 133.440 129.779 129.636 125.622 124.238 117.774 111.073
54.521 54.160 53.800 53.439 53.079
21.149 18.655 17.800 17.608 17.548 15.326 3.348
Si(nPr)3 (PF6)2
N≡C–Me
Mes
Ru
Si(nPr)3
rac-RuTPS
13C NMR (75 MHz, CD2Cl2)
200 190 180 170 160 150 140 130 120 110 100 90 80 70 60 50 40 30 20 10 ppm

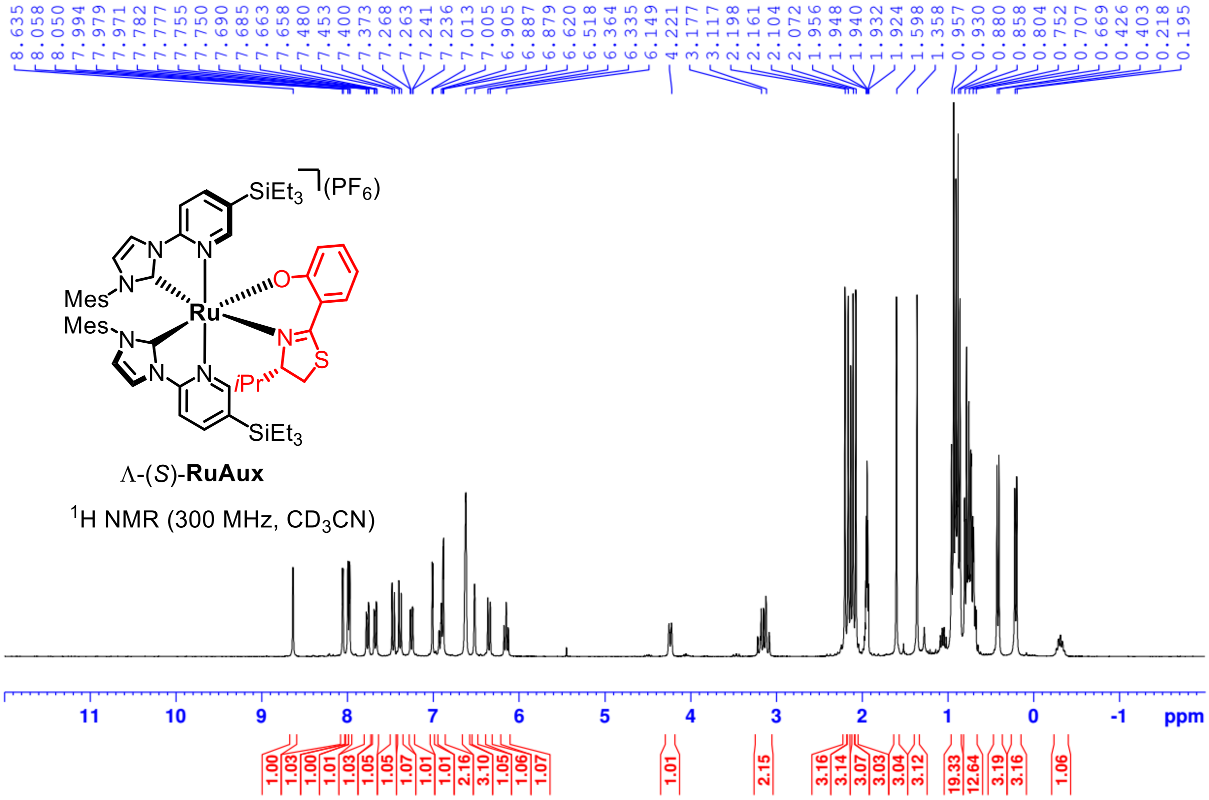
SiEt3
(PF6)
Mes
Mes
Ru
iPr
Λ-(S)-RuAux
1H NMR (300 MHz, CD3CN)
ppm

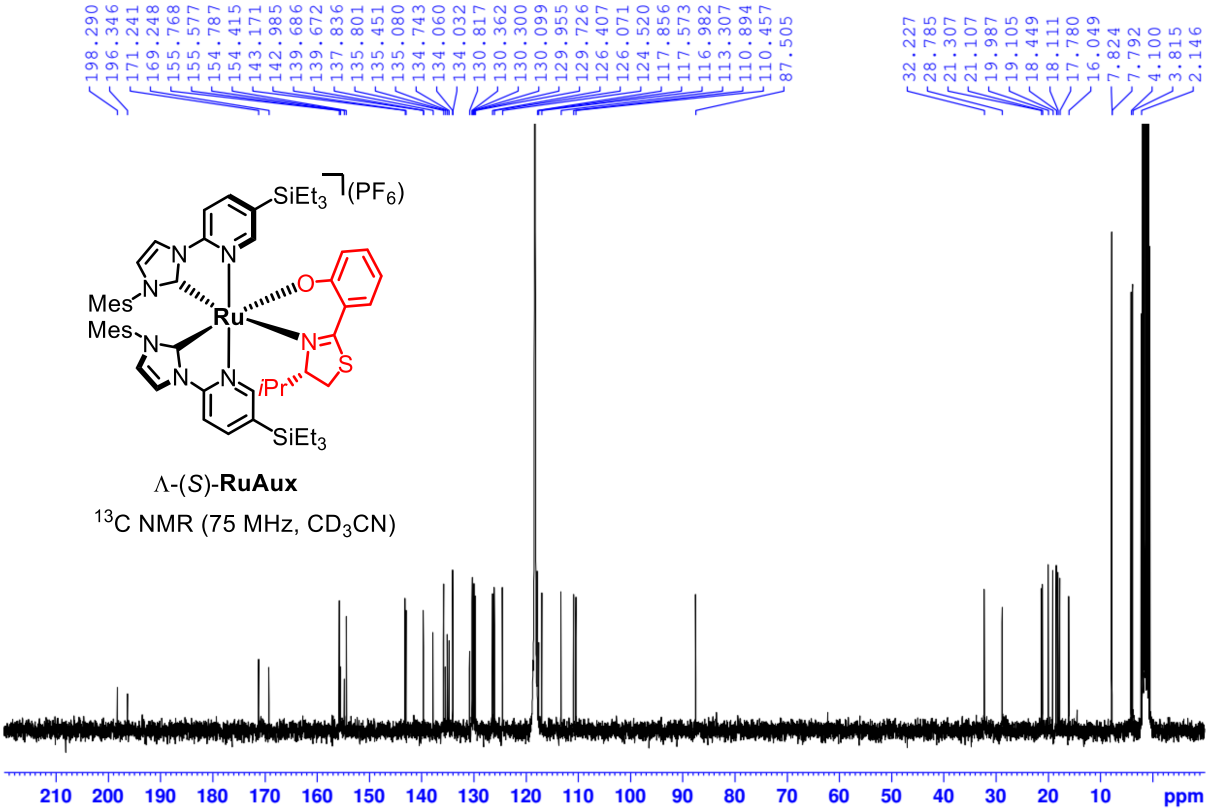
SiEt3
(PF6)
Mes
Mes
Ru
iPr
Λ-(S)-RuAux
13C NMR (75 MHz, CD3CN)
ppm

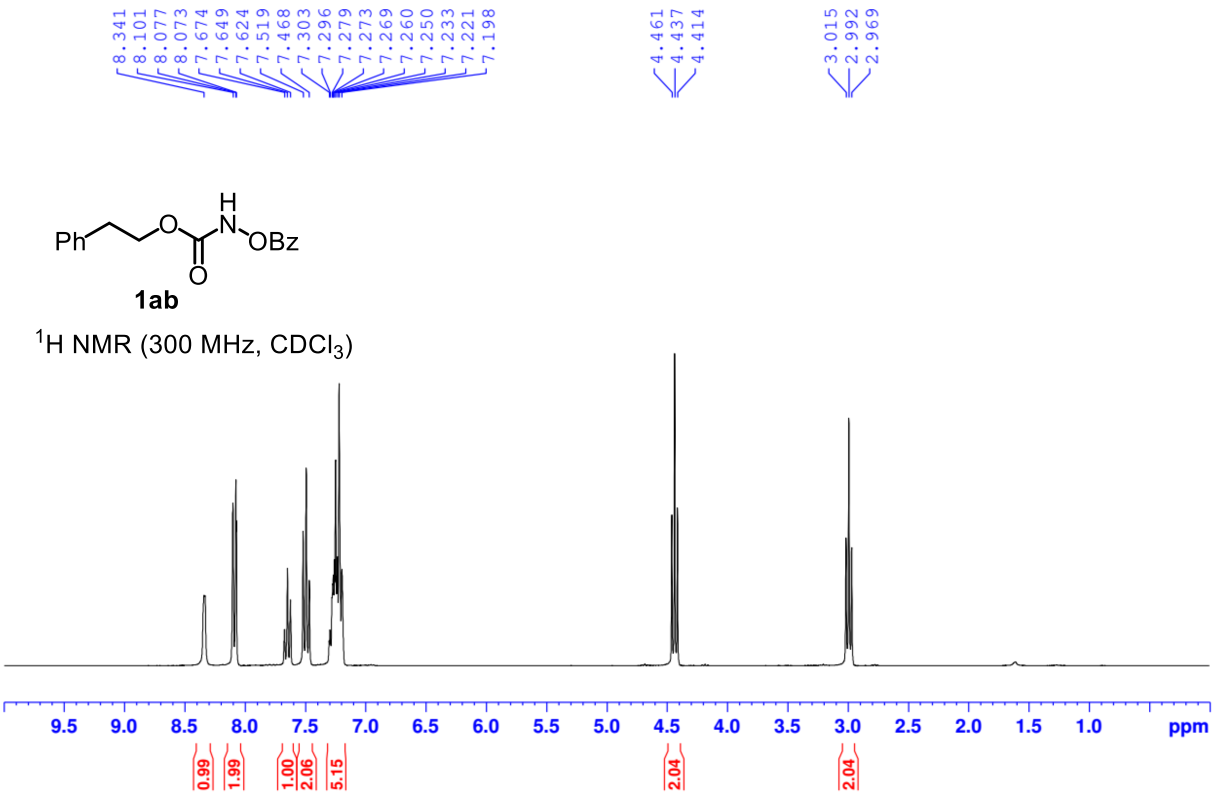

8.341
8.101
8.077
8.073
7.674
7.649
7.624
7.519
7.468
7.303
7.296
7.279
7.273
7.269
7.260
7.250
7.233
7.221
7.198
4.461
4.437
4.414
3.015
2.992
2.969
Ph
O
H
N
OBz
O
1ab
1H NMR (300 MHz, CDCl3)
9.5 9.0 8.5 8.0 7.5 7.0 6.5 6.0 5.5 5.0 4.5 4.0 3.5 3.0 2.5 2.0 1.5 1.0 ppm
0.99
1.99
1.00
2.06
5.15
2.04
2.04

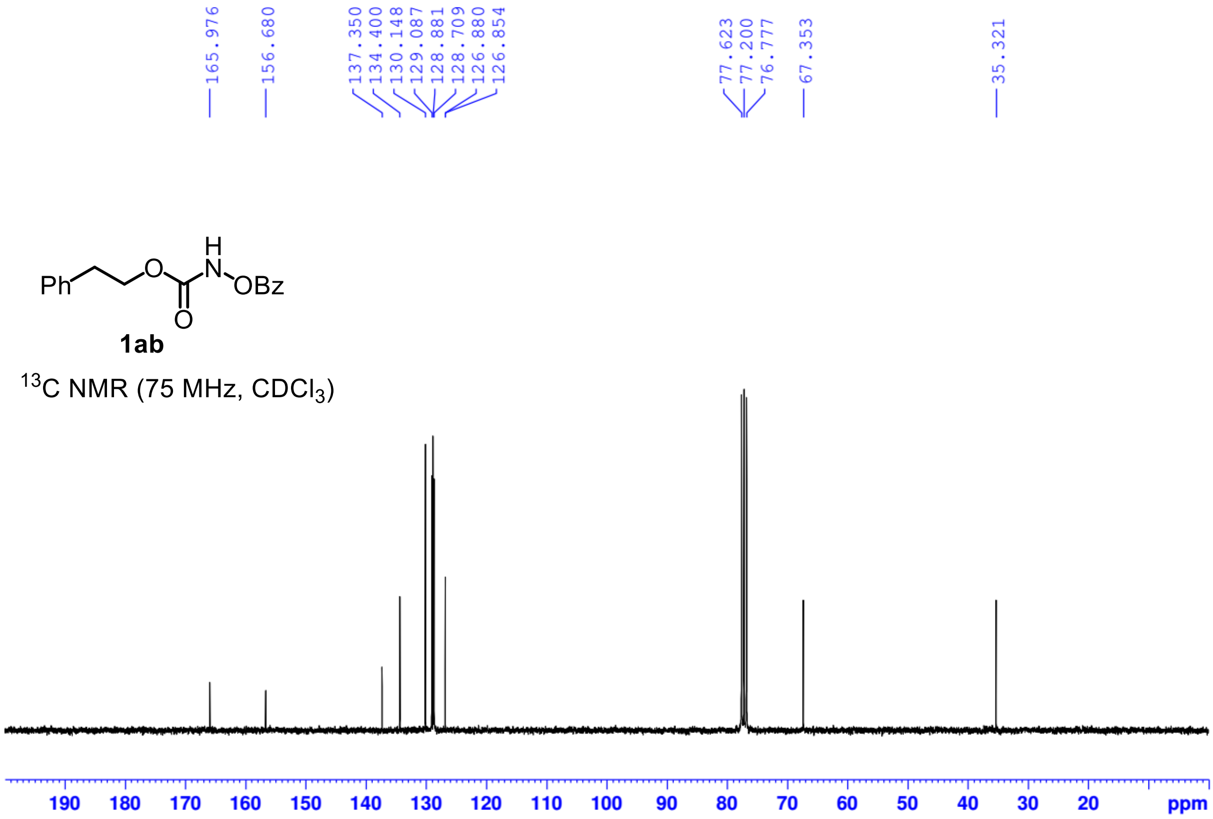

165.976
156.680
137.350
134.400
130.148
129.087
128.881
128.709
126.880
126.854
77.623
77.200
76.777
67.353
35.321
Ph
O
H
N
OBz
O
1ab
13C NMR (75 MHz, CDCl3)
190 180 170 160 150 140 130 120 110 100 90 80 70 60 50 40 30 20 ppm

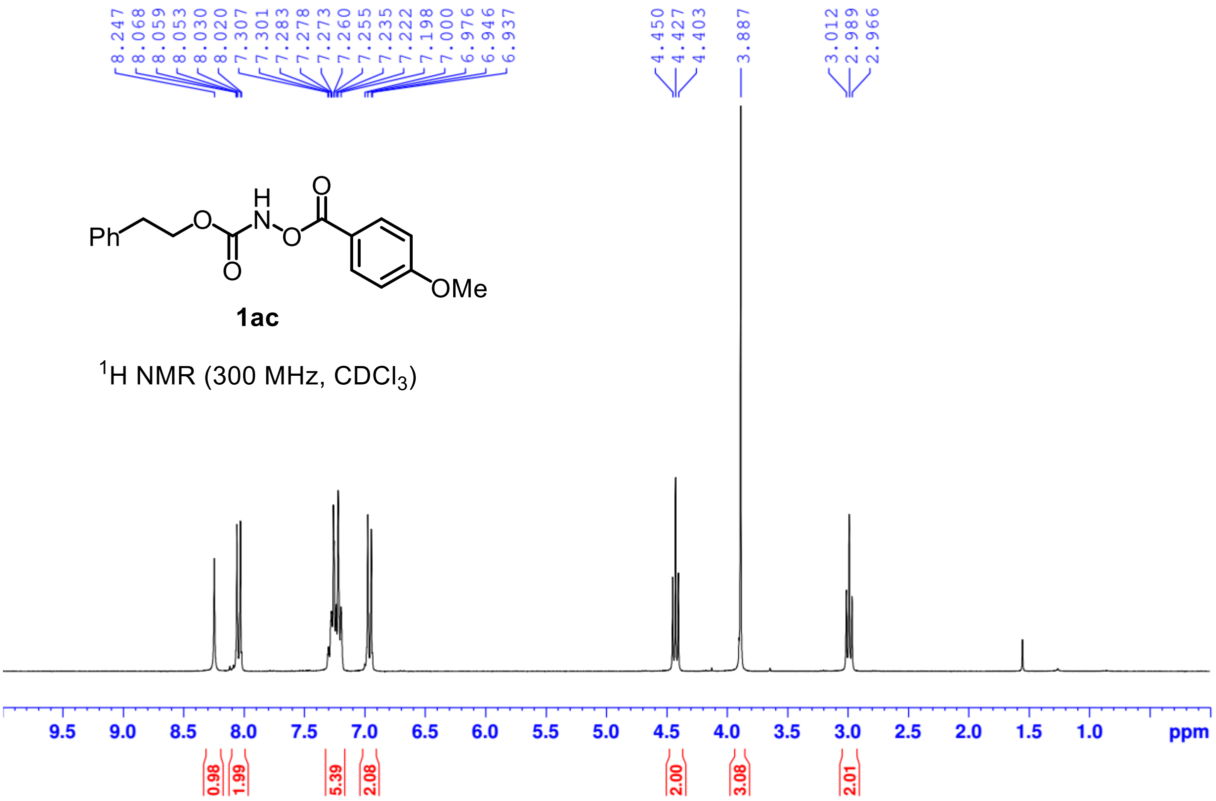
8.247
8.068
8.059
8.053
8.030
8.020
7.307
7.301
7.283
7.278
7.273
7.260
7.255
7.235
7.222
7.198
7.000
6.976
6.946
6.937
4.450
4.427
4.403
3.887
3.012
2.989
2.966
Ph
O
H
N
O
O
O
OMe
1ac
¹H NMR (300 MHz, CDCl3)
9.5 9.0 8.5 8.0 7.5 7.0 6.5 6.0 5.5 5.0 4.5 4.0 3.5 3.0 2.5 2.0 1.5 1.0 ppm
0.98
1.99
5.39
2.08
2.00
3.08
2.01

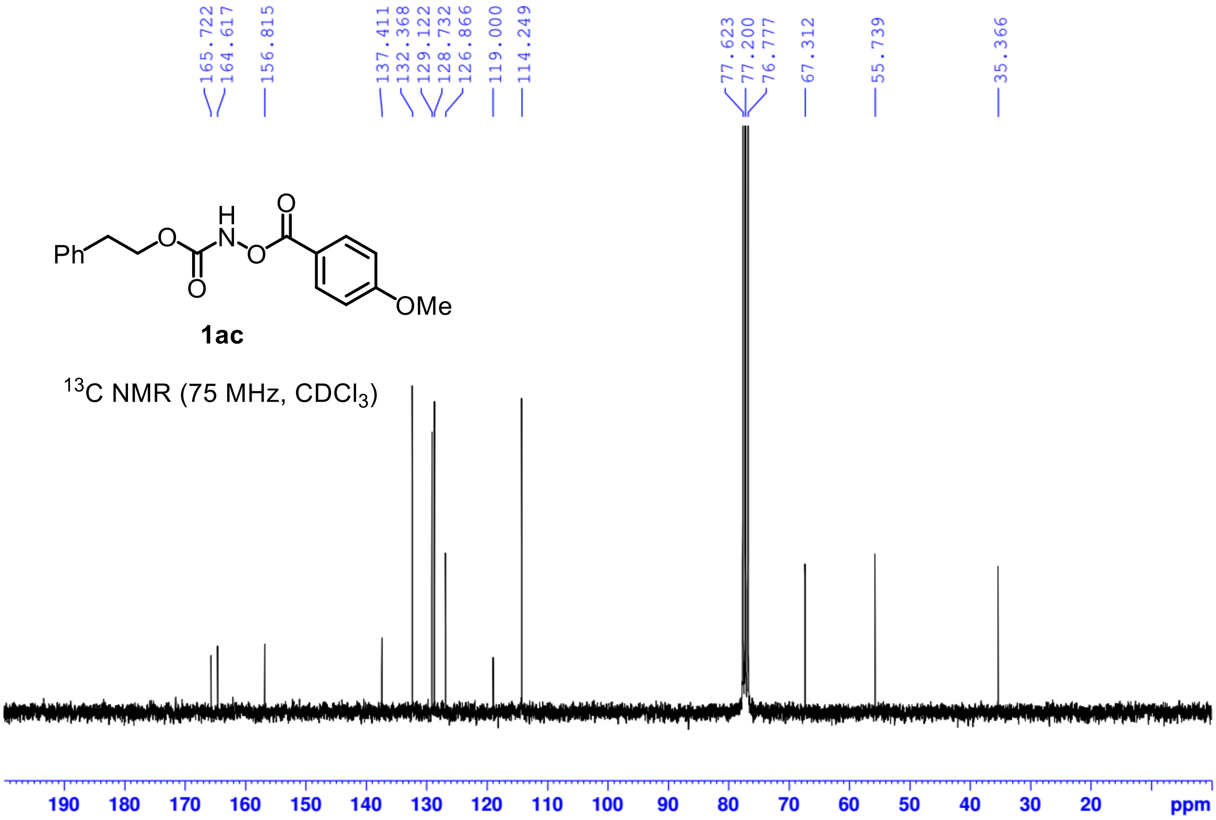
165.722
164.617
156.815
137.411
132.368
129.122
128.732
126.866
119.000
114.249
77.623
77.200
76.777
67.312
55.739
35.366
Ph
O
H
N
O
O
O
OMe
1ac
¹³C NMR (75 MHz, CDCl3)
190 180 170 160 150 140 130 120 110 100 90 80 70 60 50 40 30 20 ppm

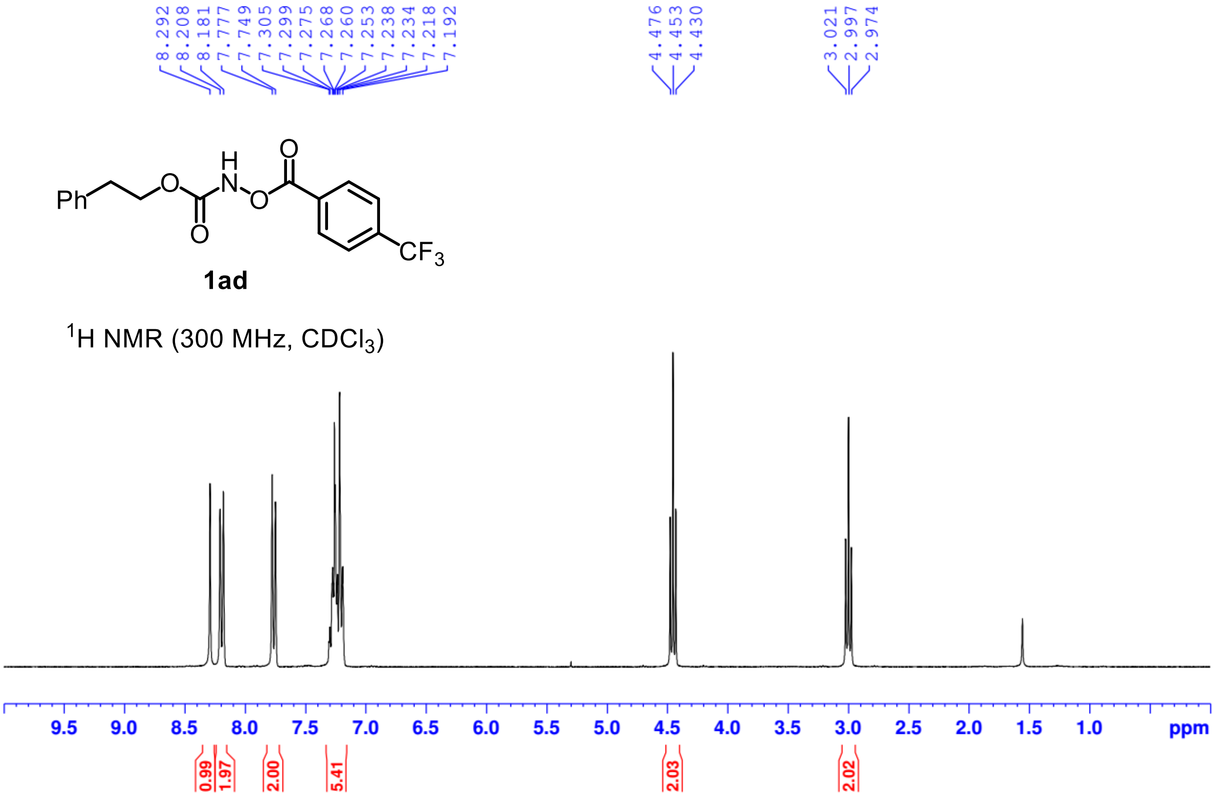
8.292
8.208
8.181
7.777
7.749
7.305
7.299
7.275
7.268
7.260
7.253
7.238
7.234
7.218
7.192
4.476
4.453
4.430
3.021
2.997
2.974
Ph
H
N
O
O
O
O
CF3
1ad
1H NMR (300 MHz, CDCl3)
0.99
1.97
2.00
5.41
2.03
2.02
ppm

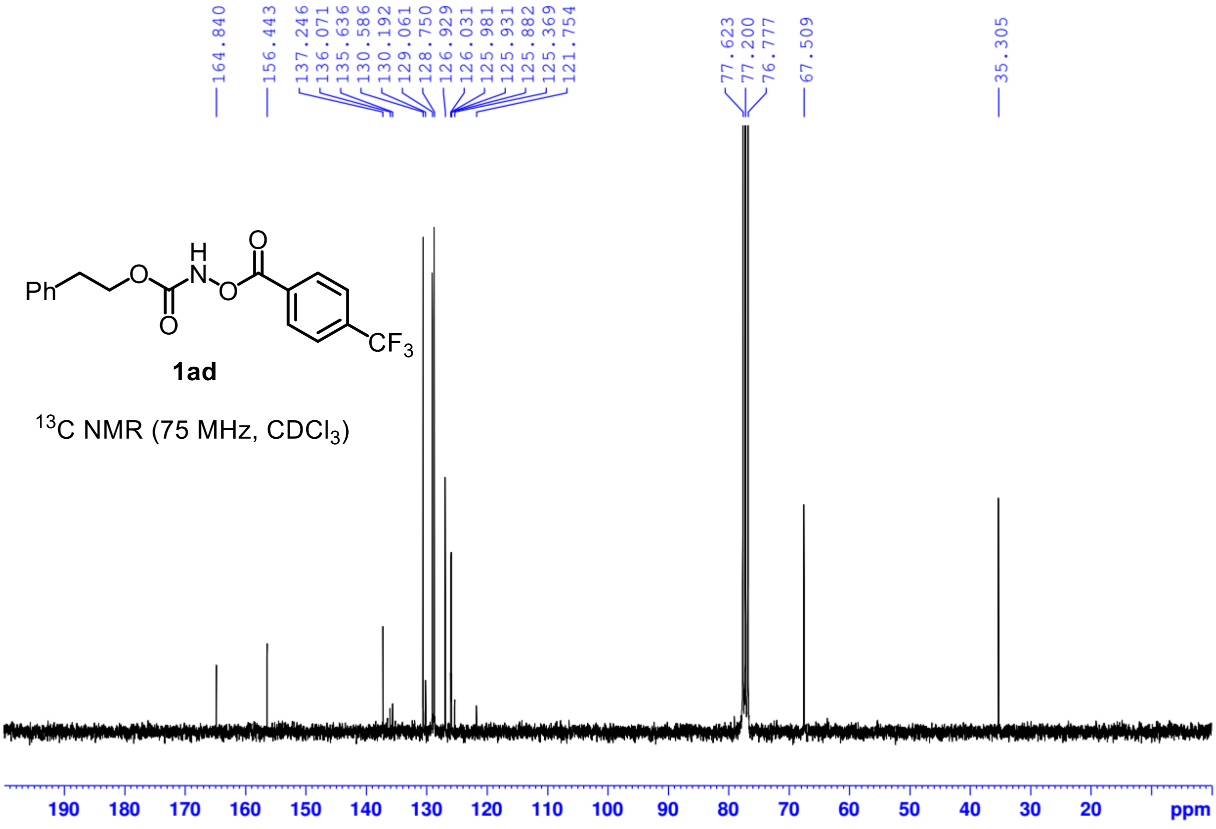
164.840
156.443
137.246
136.071
135.636
130.586
130.192
129.061
128.750
126.929
126.031
125.981
125.931
125.882
125.369
121.754
77.623
77.200
76.777
67.509
35.305
Ph
H
N
O
O
O
O
CF3
1ad
13C NMR (75 MHz, CDCl3)
ppm

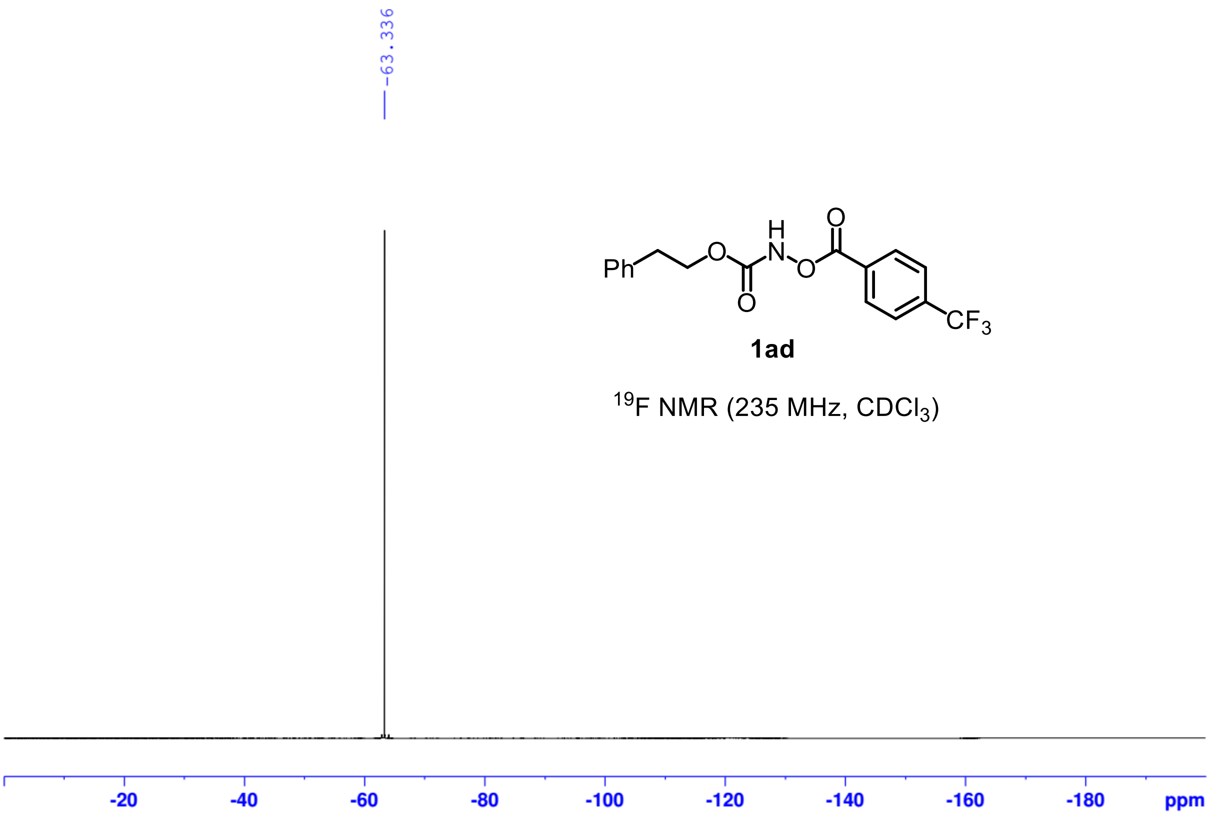
-63.336
Ph
H
N
O
O
O
O
CF3
1ad
19F NMR (235 MHz, CDCl3)
-20
-40
-60
-80
-100
-120
-140
-160
-180
ppm

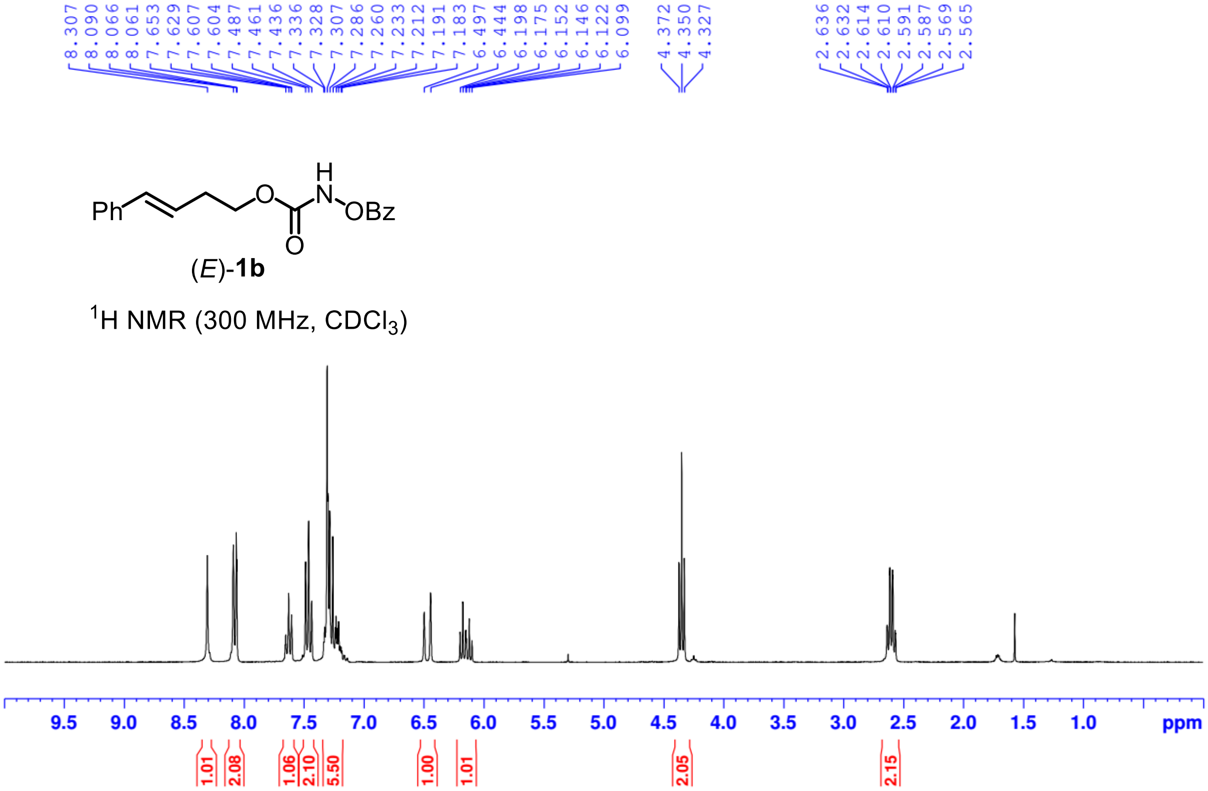
8.307
8.090
8.066
8.061
7.653
7.629
7.607
7.604
7.487
7.461
7.436
7.336
7.328
7.307
7.286
7.260
7.233
7.212
7.191
7.183
6.497
6.444
6.198
6.175
6.152
6.146
6.122
6.099
4.372
4.350
4.327
2.636
2.632
2.614
2.610
2.591
2.587
2.569
2.565
Ph
H
N
O
OBz
O
(E)-1b
1H NMR (300 MHz, CDCl3)
9.5
9.0
8.5
8.0
7.5
7.0
6.5
6.0
5.5
5.0
4.5
4.0
3.5
3.0
2.5
2.0
1.5
1.0
ppm
1.01
2.08
1.06
2.10
5.50
1.00
1.01
2.05
2.15

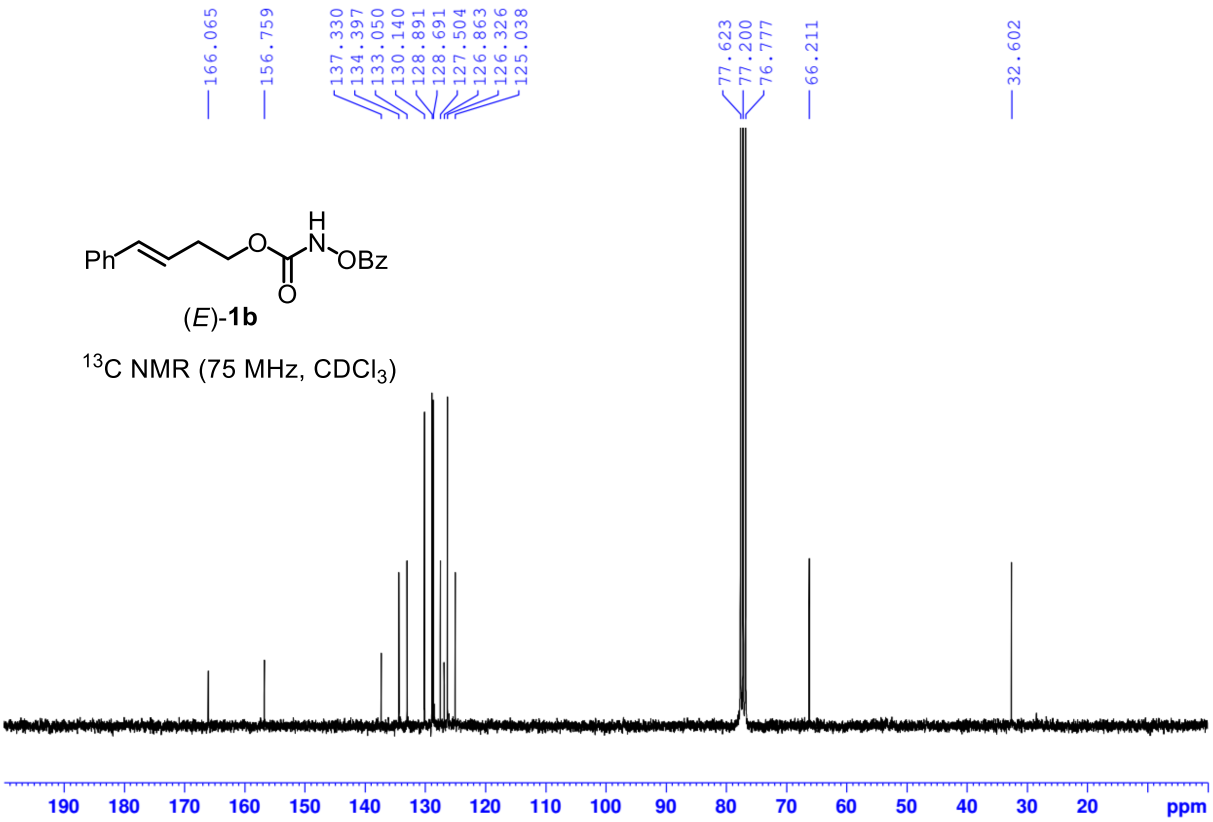

(*E*)-**1b**

^{13}C NMR (75 MHz, $CDCl_3$)

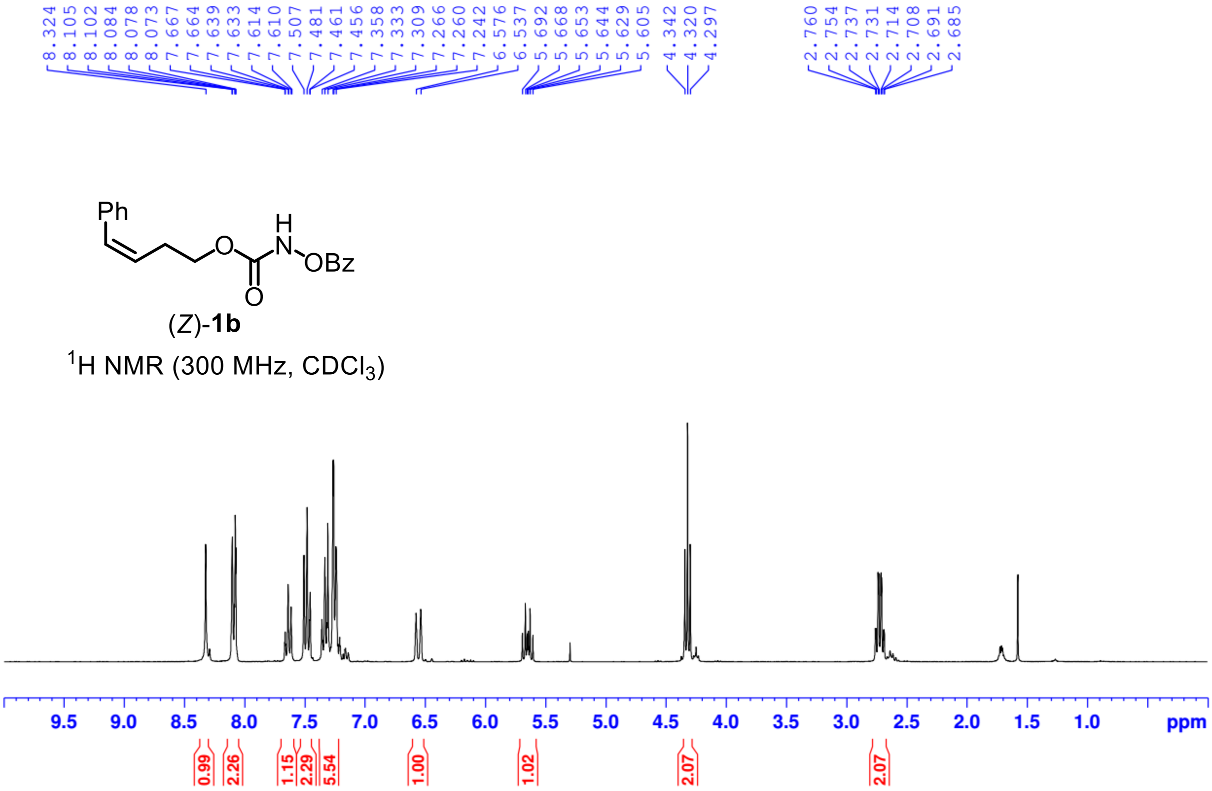

(*Z*)-**1b**

^{1}H NMR (300 MHz, $CDCl_3$)

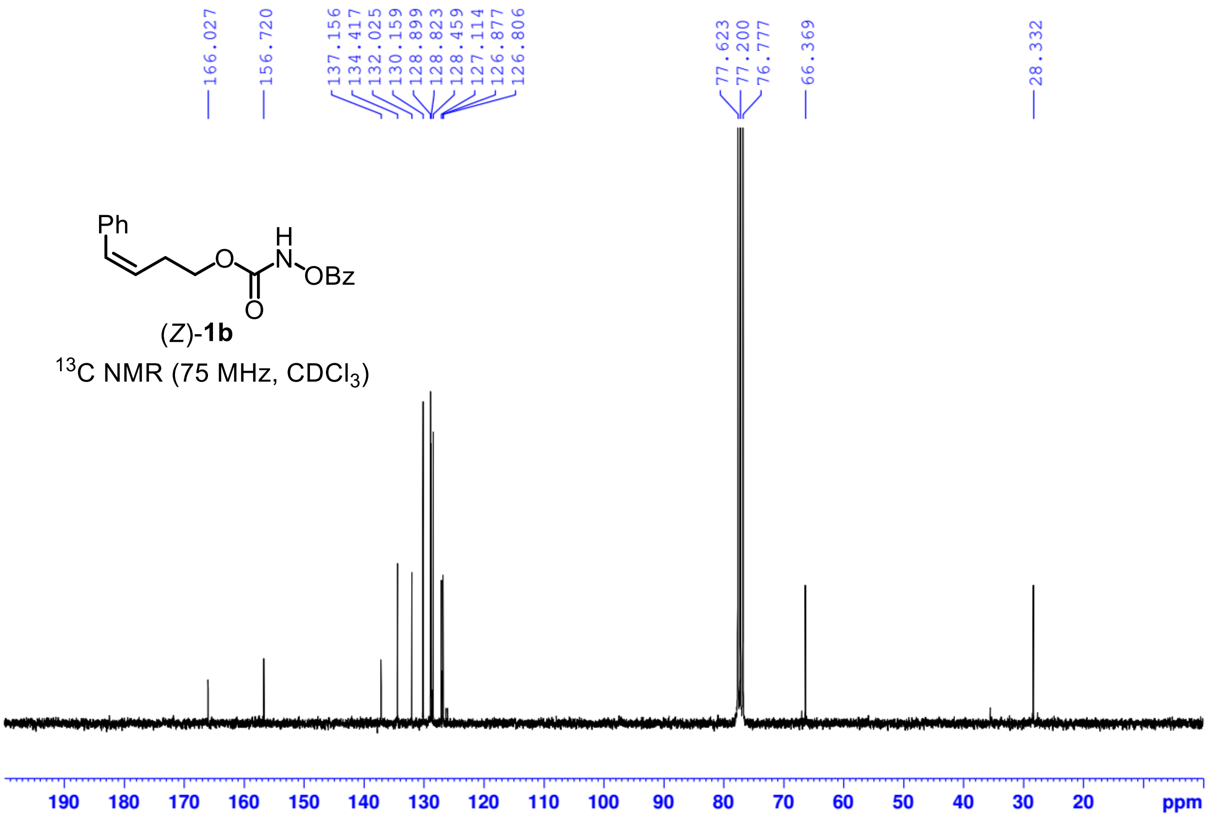
166.027
156.720
137.156
134.417
132.025
130.159
128.899
128.823
128.459
127.114
126.877
126.806
77.623
77.200
76.777
66.369
28.332
Ph
H
N
O
OBz
O
(Z)-1b
13C NMR (75 MHz, CDCl3)
190 180 170 160 150 140 130 120 110 100 90 80 70 60 50 40 30 20 ppm

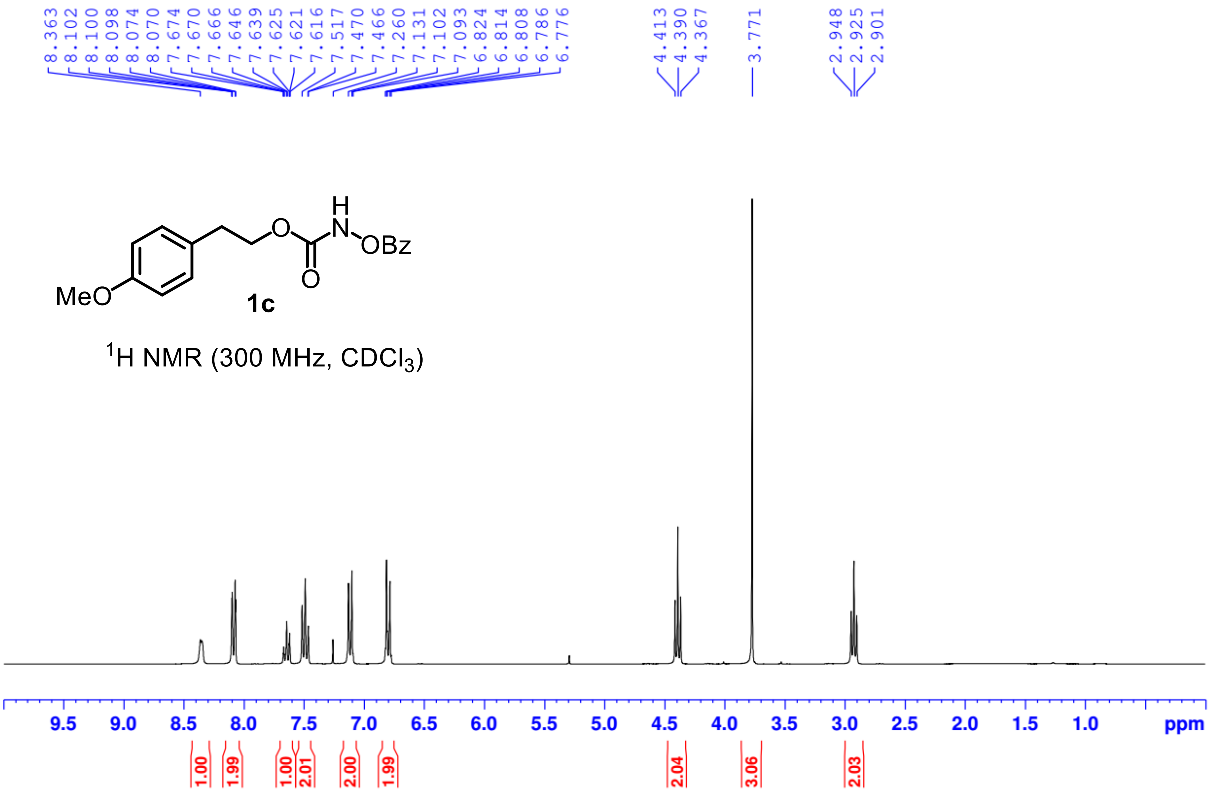
8.363
8.102
8.100
8.098
8.074
8.070
7.674
7.670
7.666
7.646
7.639
7.625
7.621
7.616
7.517
7.470
7.466
7.260
7.131
7.102
7.093
6.824
6.814
6.808
6.786
6.776
4.413
4.390
4.367
3.771
2.948
2.925
2.901
MeO
H
N
O
OBz
O
1c
1H NMR (300 MHz, CDCl3)
9.5 9.0 8.5 8.0 7.5 7.0 6.5 6.0 5.5 5.0 4.5 4.0 3.5 3.0 2.5 2.0 1.5 1.0 ppm
1.00
1.99
1.00
2.01
2.00
1.99
2.04
3.06
2.03

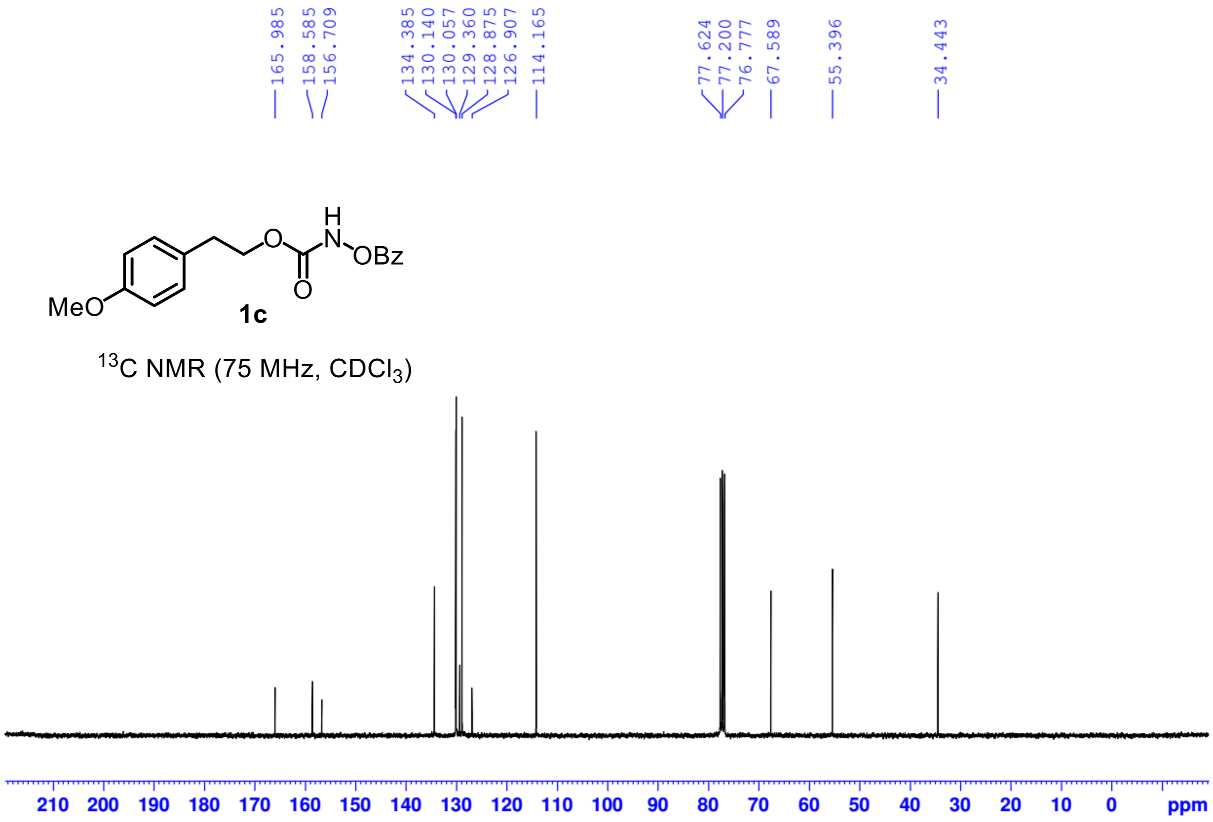
165.985
158.585
156.709
134.385
130.140
130.057
129.360
128.875
126.907
114.165
77.624
77.200
76.777
67.589
55.396
34.443
MeO
H
N
O
OBz
O
1c
^{13}C NMR (75 MHz, CDCl$_3$)
210 200 190 180 170 160 150 140 130 120 110 100 90 80 70 60 50 40 30 20 10 0 ppm

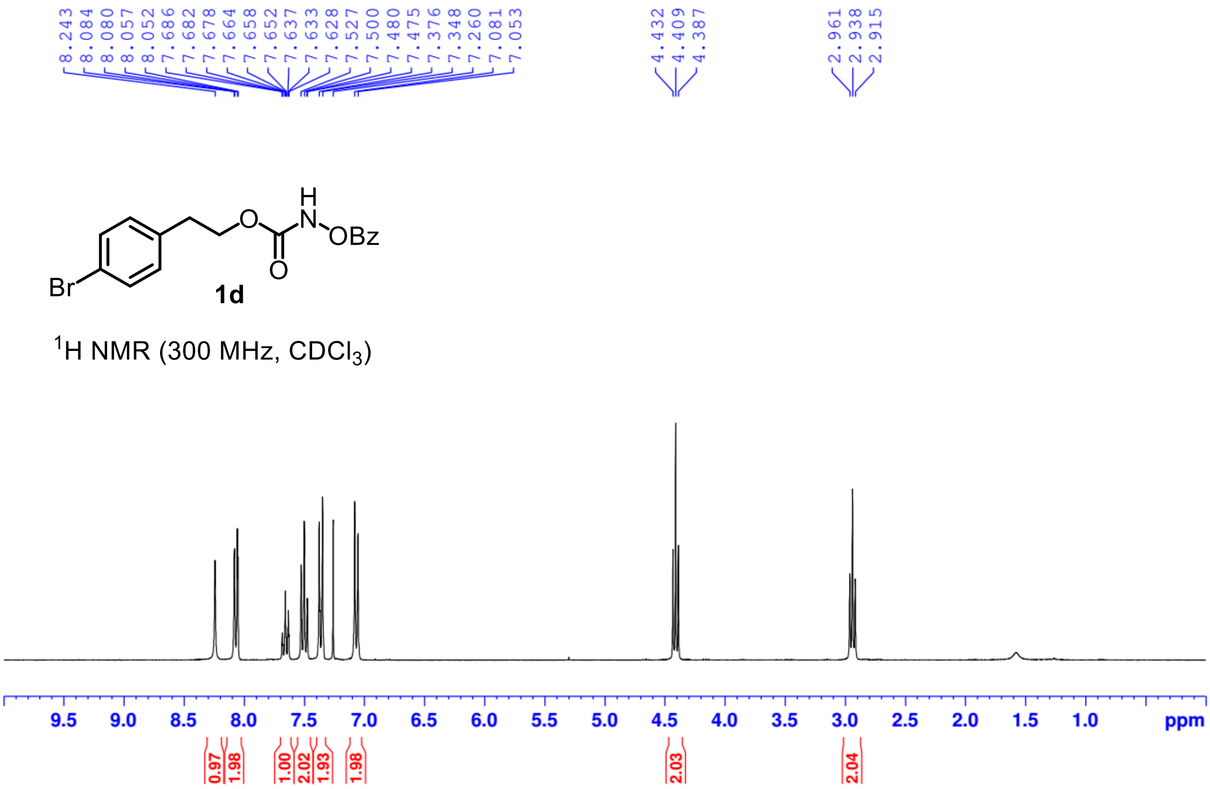
8.243
8.084
8.080
8.057
8.052
7.686
7.682
7.678
7.664
7.658
7.652
7.637
7.633
7.628
7.527
7.500
7.480
7.475
7.376
7.348
7.260
7.081
7.053
4.432
4.409
4.387
2.961
2.938
2.915
Br
H
N
O
OBz
O
1d
^{1}H NMR (300 MHz, CDCl$_3$)
9.5 9.0 8.5 8.0 7.5 7.0 6.5 6.0 5.5 5.0 4.5 4.0 3.5 3.0 2.5 2.0 1.5 1.0 ppm
0.97
1.98
1.00
2.02
1.93
1.98
2.03
2.04

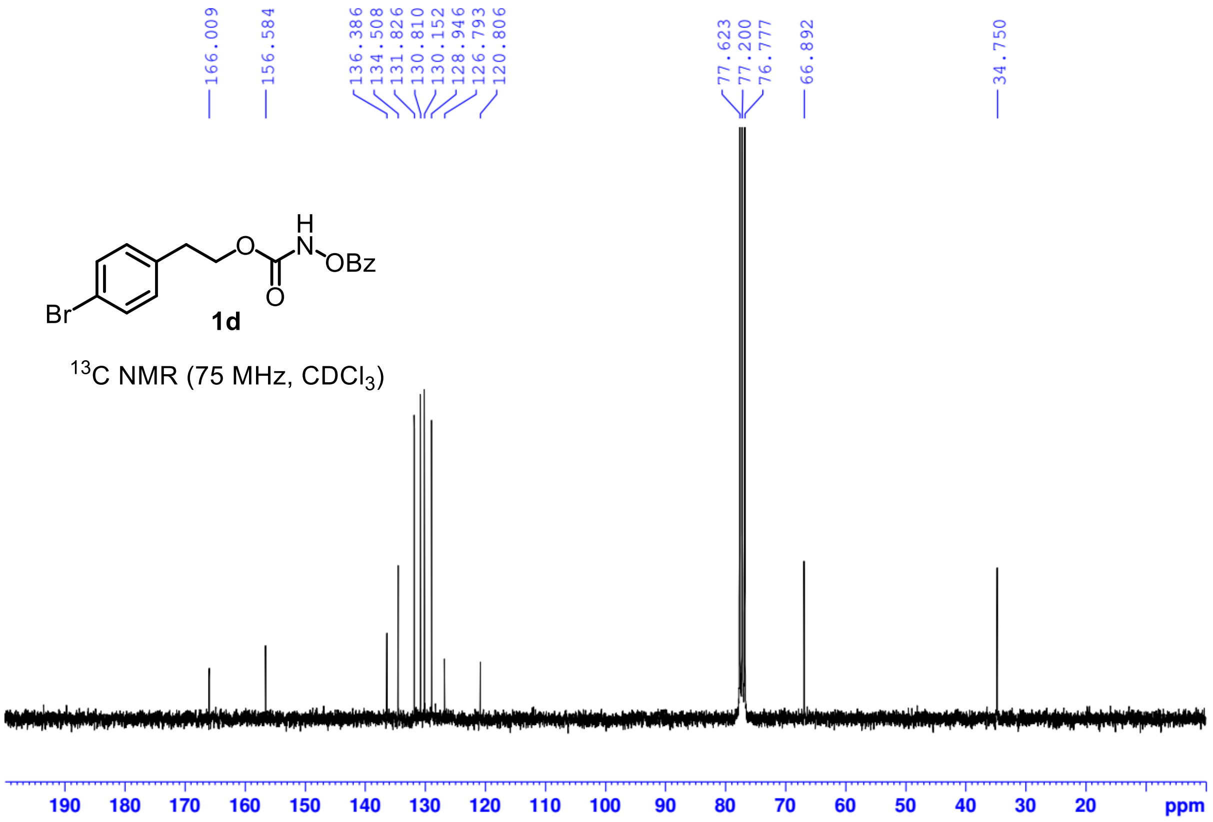
166.009
156.584
136.386
134.508
131.826
130.810
130.152
128.946
126.793
120.806
77.623
77.200
76.777
66.892
34.750
OBz
Br
1d
13C NMR (75 MHz, CDCl3)
190 180 170 160 150 140 130 120 110 100 90 80 70 60 50 40 30 20 ppm

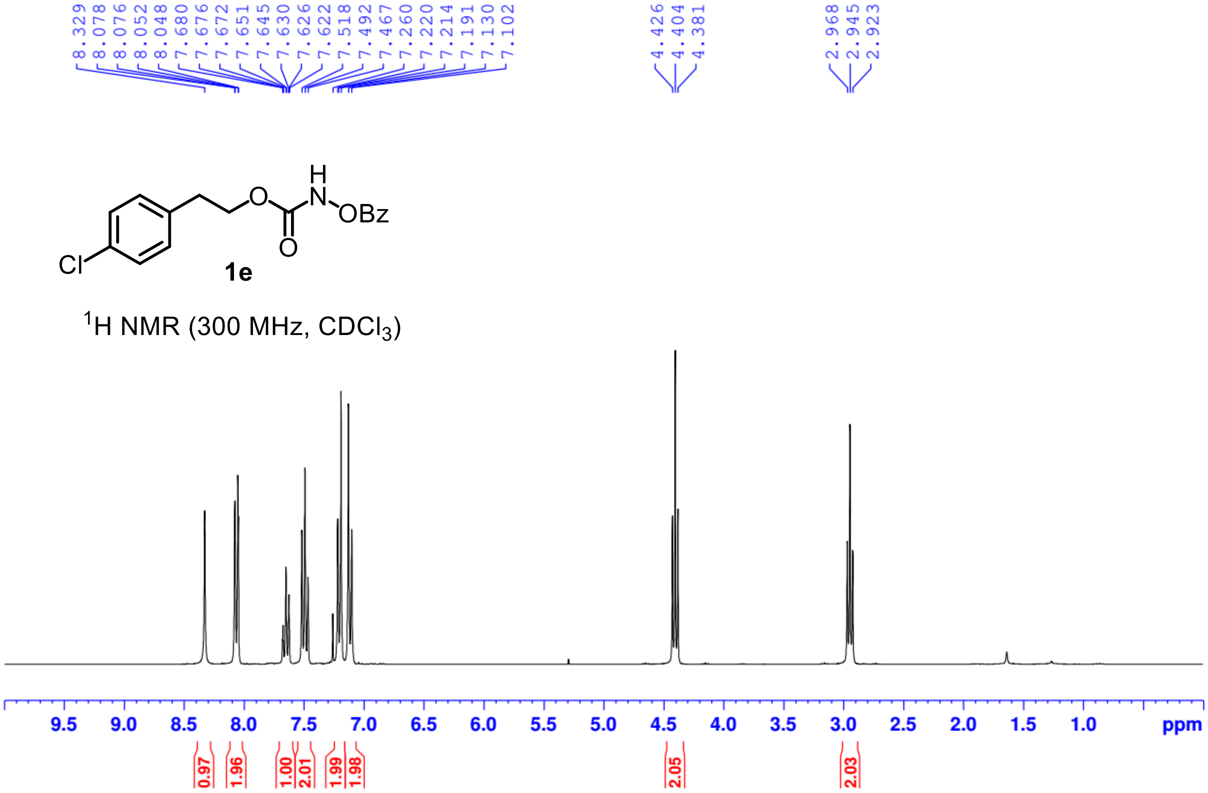
8.329
8.078
8.076
8.052
8.048
7.680
7.676
7.672
7.651
7.645
7.630
7.626
7.622
7.518
7.492
7.467
7.260
7.220
7.214
7.191
7.130
7.102
4.426
4.404
4.381
2.968
2.945
2.923
OBz
Cl
1e
1H NMR (300 MHz, CDCl3)
9.5 9.0 8.5 8.0 7.5 7.0 6.5 6.0 5.5 5.0 4.5 4.0 3.5 3.0 2.5 2.0 1.5 1.0 ppm
0.97
1.96
1.00
2.01
1.99
1.98
2.05
2.03

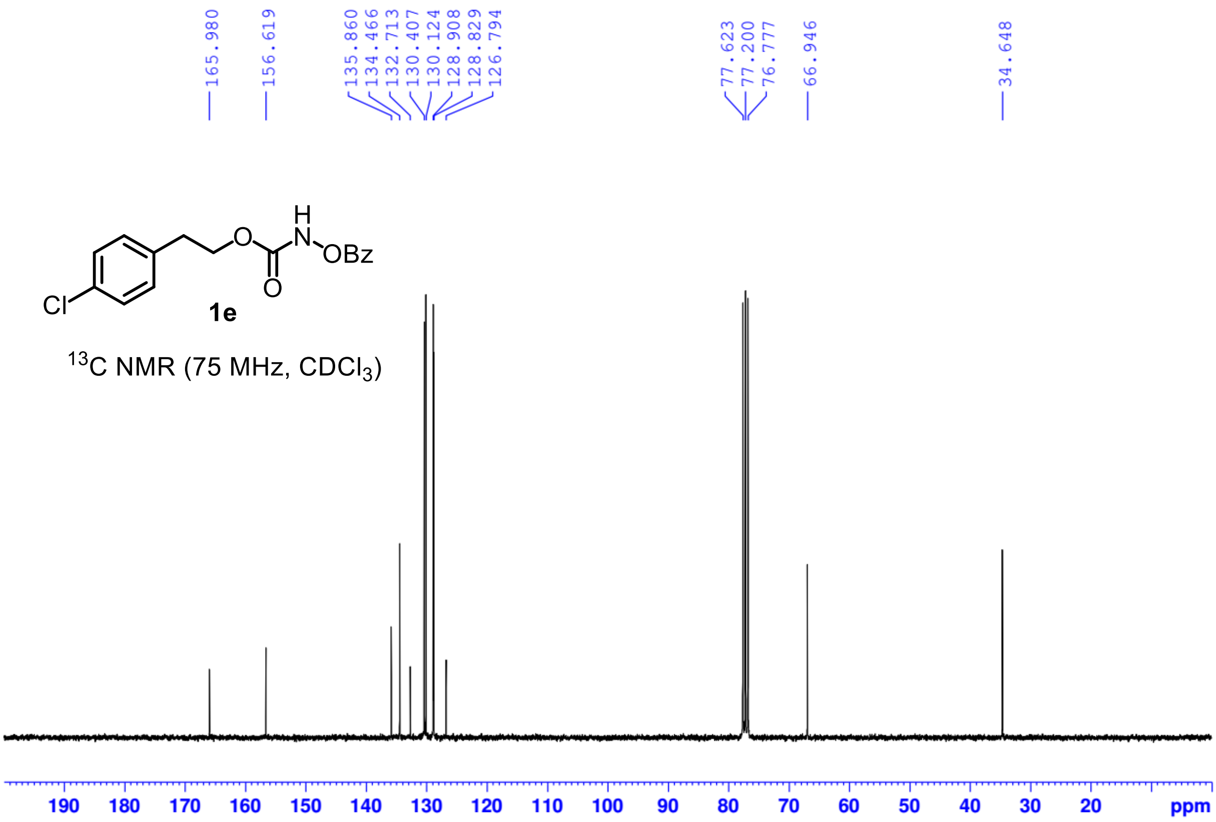
165.980
156.619
135.860
134.466
132.713
130.407
130.124
128.908
128.829
126.794
77.623
77.200
76.777
66.946
34.648
Cl
O
H
N
OBz
O
1e
13C NMR (75 MHz, CDCl3)
ppm

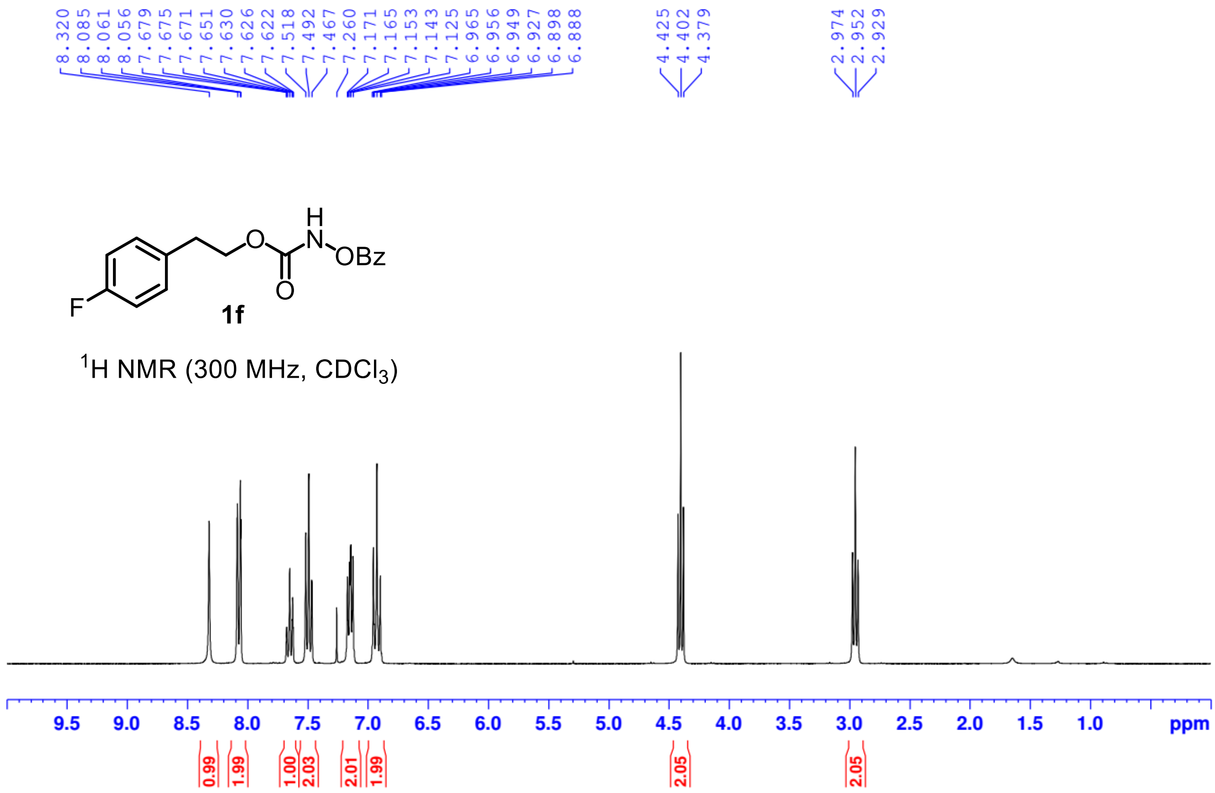
8.320
8.085
8.061
8.056
7.679
7.675
7.671
7.651
7.630
7.626
7.622
7.518
7.492
7.467
7.260
7.171
7.165
7.153
7.143
7.125
6.965
6.956
6.949
6.927
6.898
6.888
4.425
4.402
4.379
2.974
2.952
2.929
F
O
H
N
OBz
O
1f
1H NMR (300 MHz, CDCl3)
ppm
0.99
1.99
1.00
2.03
2.01
1.99
2.05
2.05

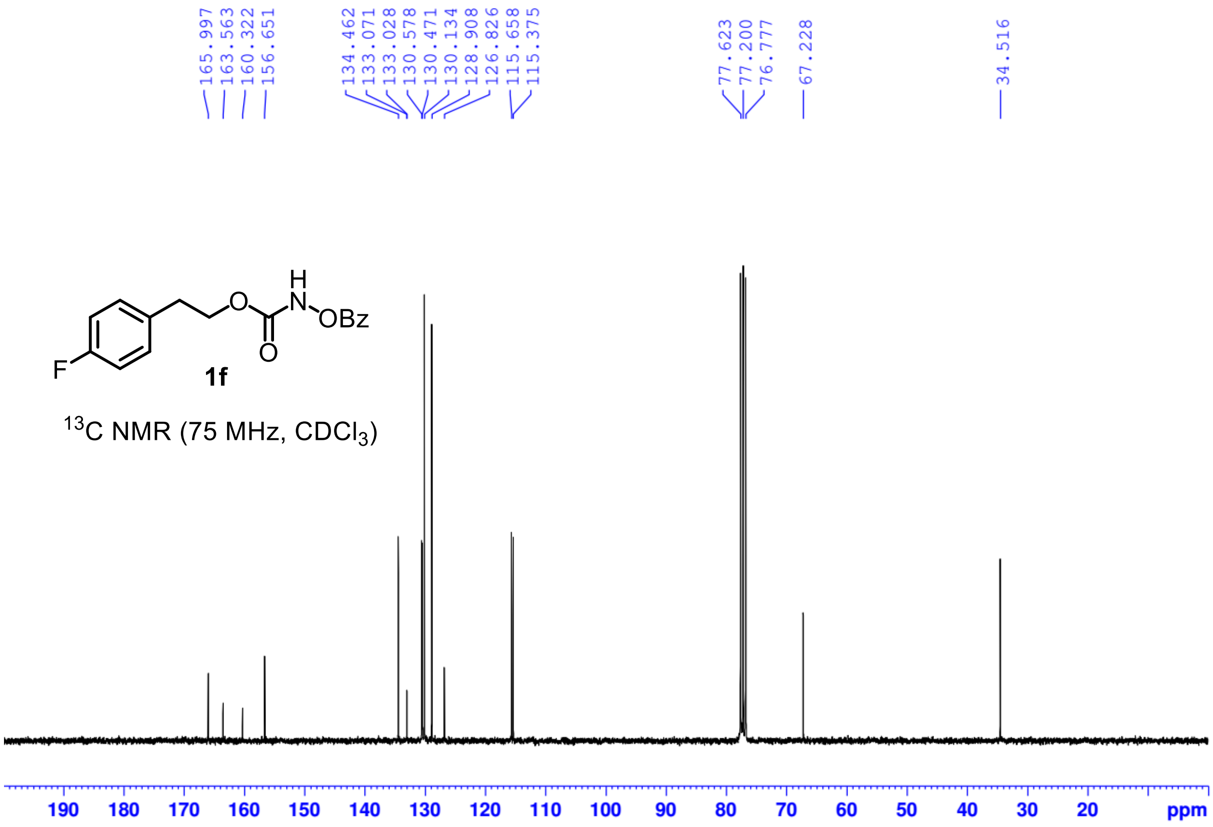
165.997
163.563
160.322
156.651
134.462
133.071
133.028
130.578
130.471
130.134
128.908
126.826
115.658
115.375
77.623
77.200
76.777
67.228
34.516
H
N
O
OBz
O
F
1f
^{13}C NMR (75 MHz, $CDCl_3$)
190 180 170 160 150 140 130 120 110 100 90 80 70 60 50 40 30 20 ppm

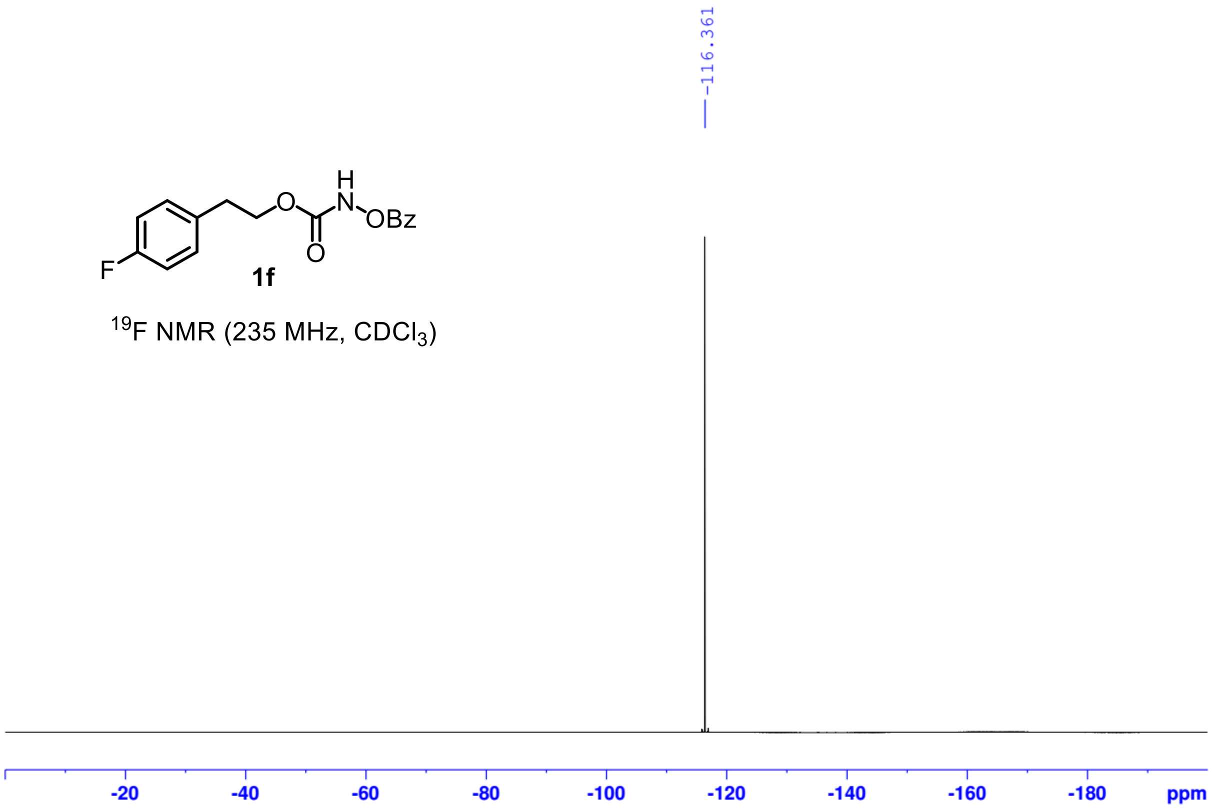
-116.361
H
N
O
OBz
O
F
1f
^{19}F NMR (235 MHz, $CDCl_3$)
-20 -40 -60 -80 -100 -120 -140 -160 -180 ppm

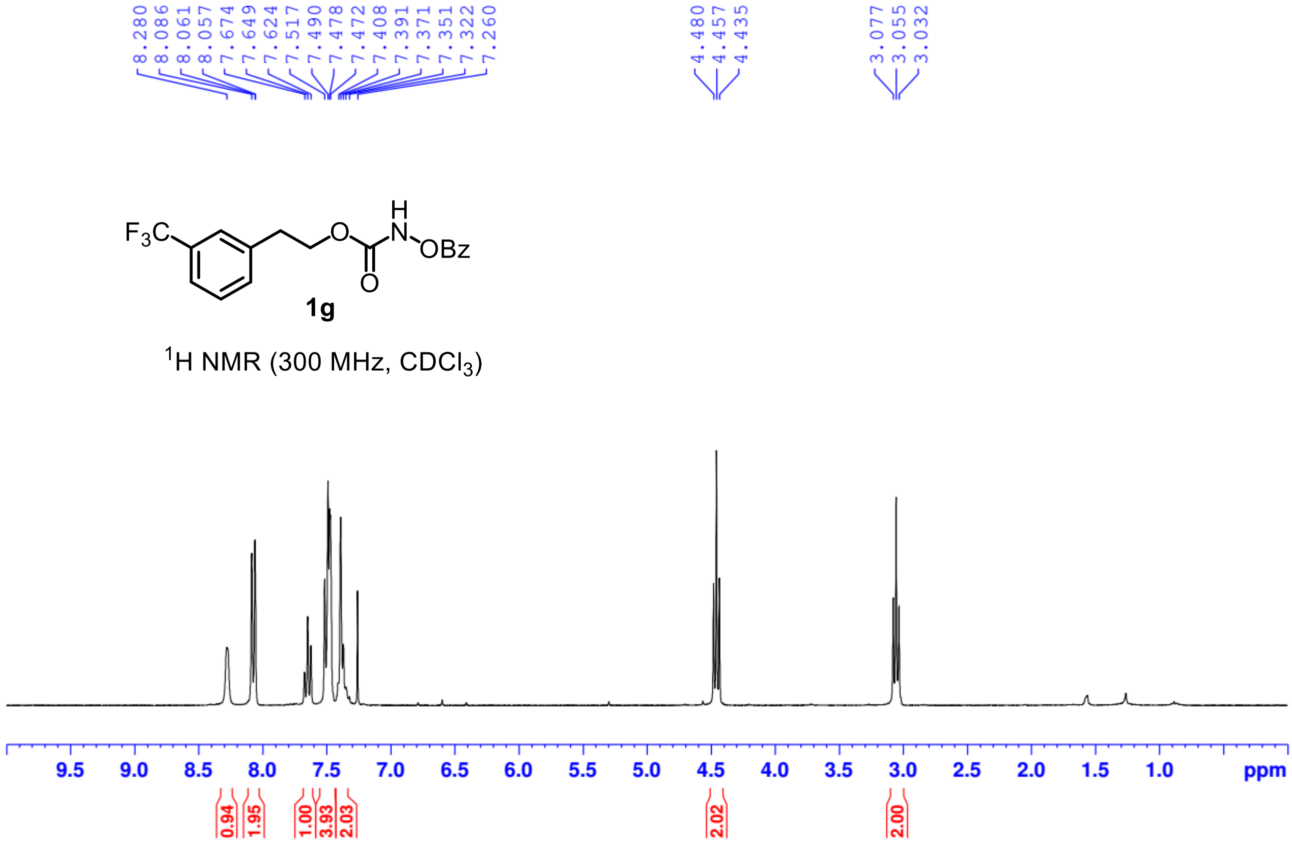

8.280
8.086
8.061
8.057
7.674
7.649
7.624
7.517
7.490
7.478
7.472
7.408
7.391
7.371
7.351
7.322
7.260
4.480
4.457
4.435
3.077
3.055
3.032
F3C
H
N
O
OBz
O
1g
1H NMR (300 MHz, CDCl3)
9.5 9.0 8.5 8.0 7.5 7.0 6.5 6.0 5.5 5.0 4.5 4.0 3.5 3.0 2.5 2.0 1.5 1.0 ppm
0.94
1.95
1.00
3.93
2.03
2.02
2.00

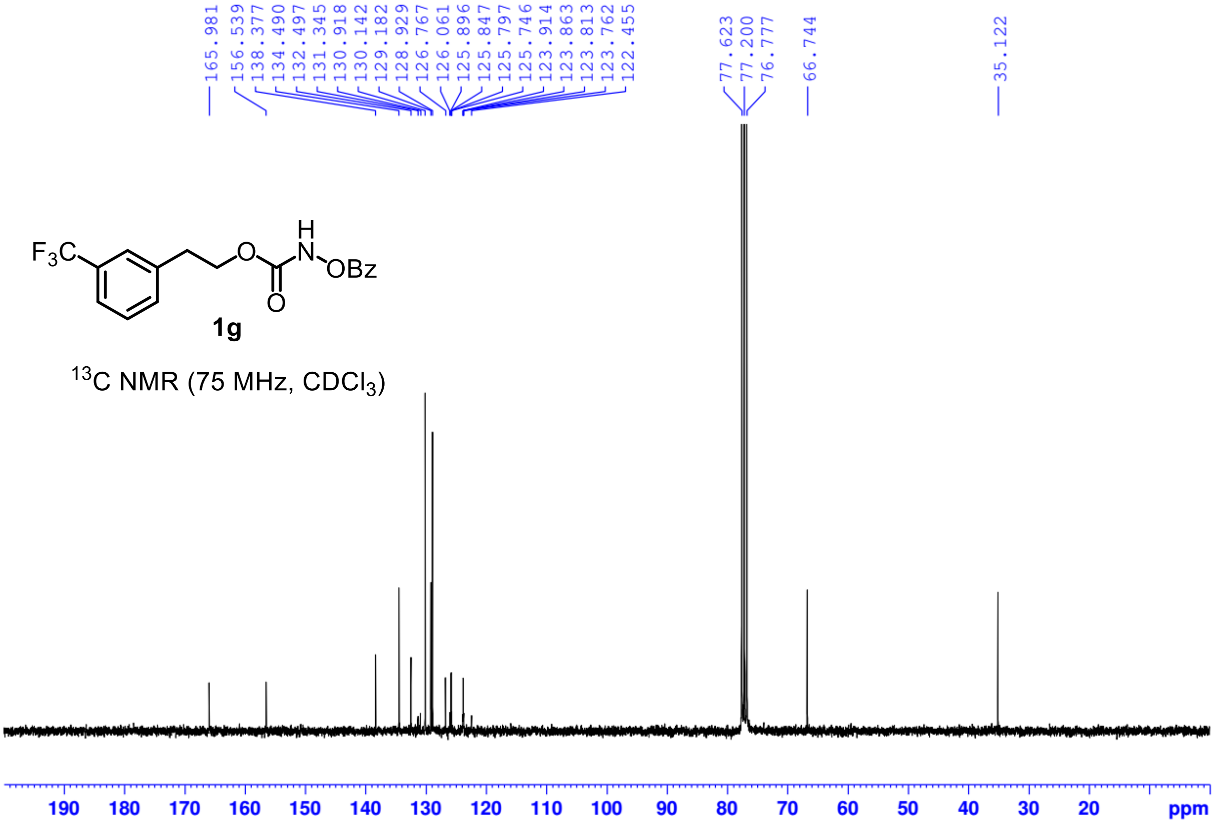

165.981
156.539
138.377
134.490
132.497
131.345
130.918
130.142
129.182
128.929
126.767
126.061
125.896
125.847
125.797
125.746
123.914
123.863
123.813
123.762
122.455
77.623
77.200
76.777
66.744
35.122
F3C
H
N
O
OBz
O
1g
13C NMR (75 MHz, CDCl3)
190 180 170 160 150 140 130 120 110 100 90 80 70 60 50 40 30 20 ppm

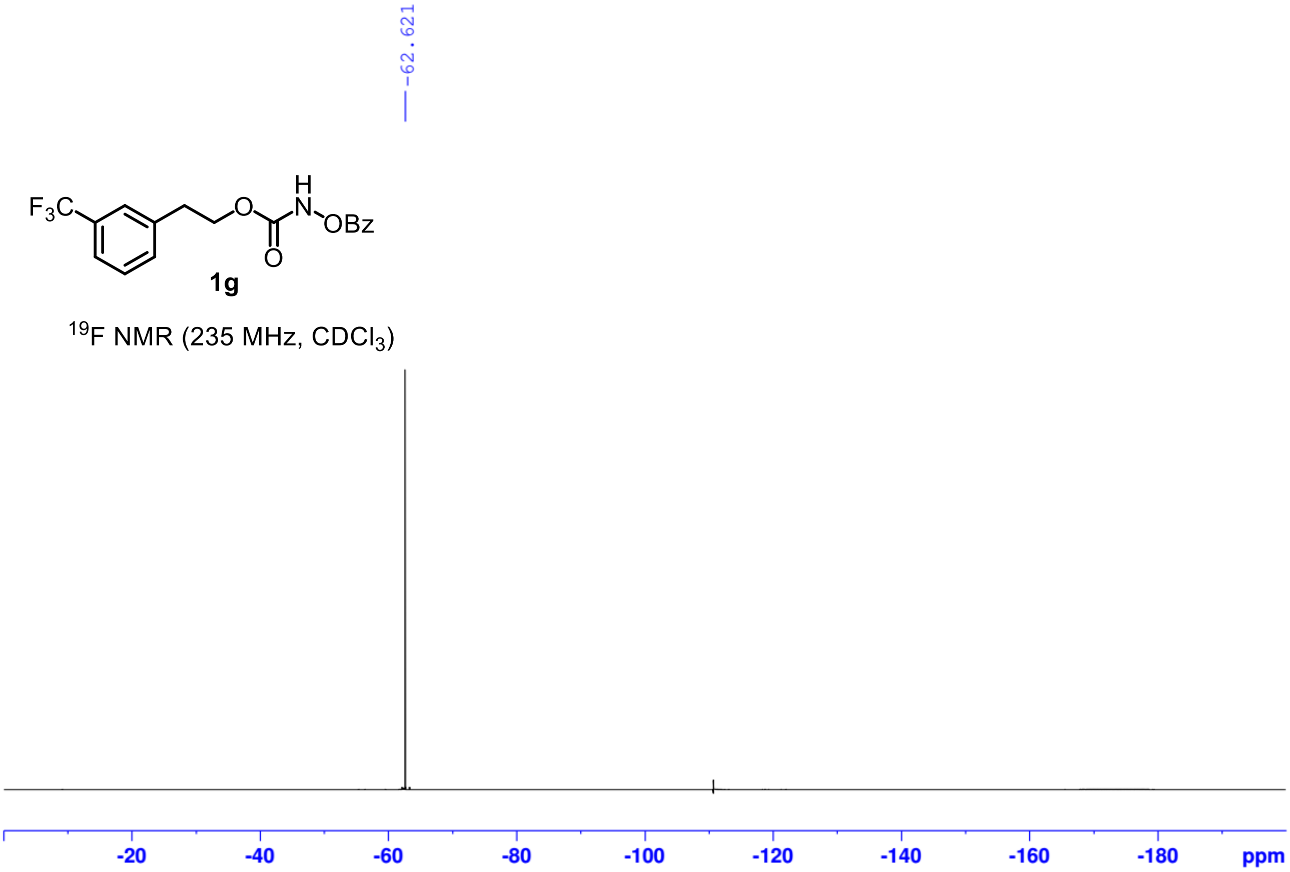

1g

^{19}F NMR (235 MHz, $CDCl_3$)

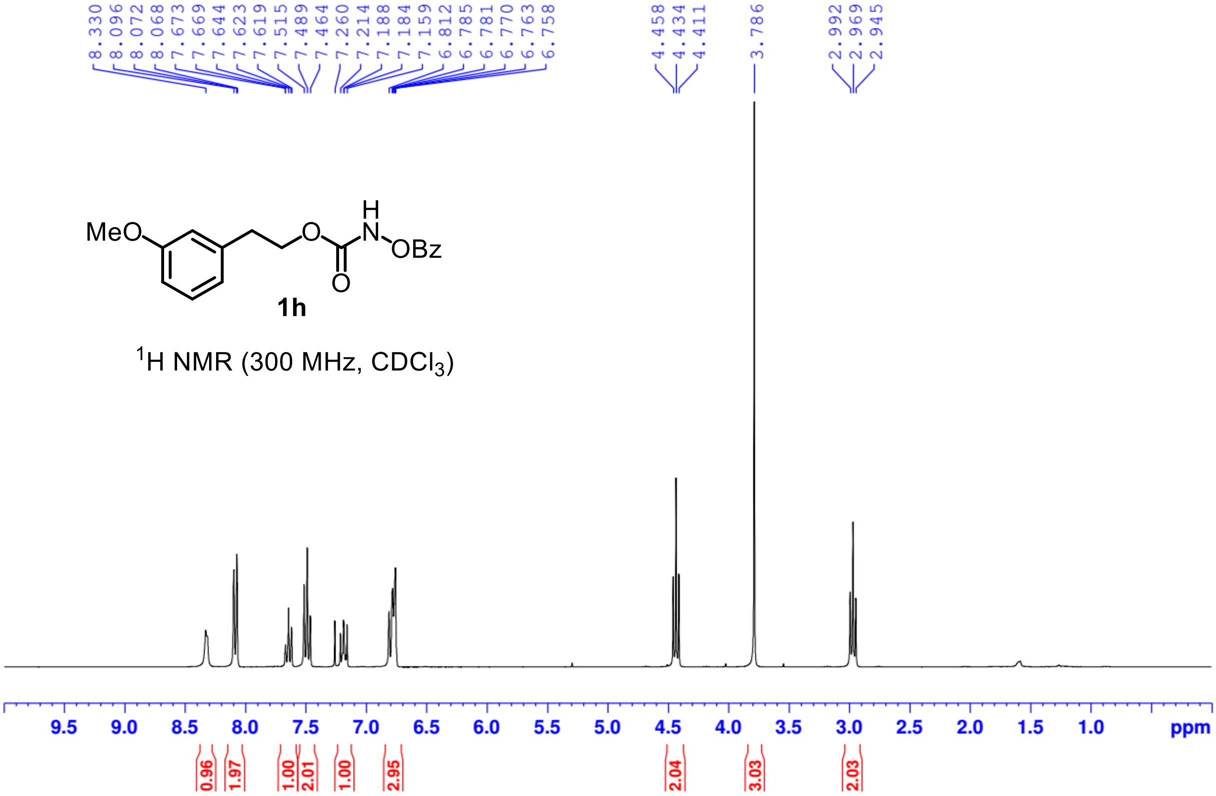

1h

^{1}H NMR (300 MHz, $CDCl_3$)

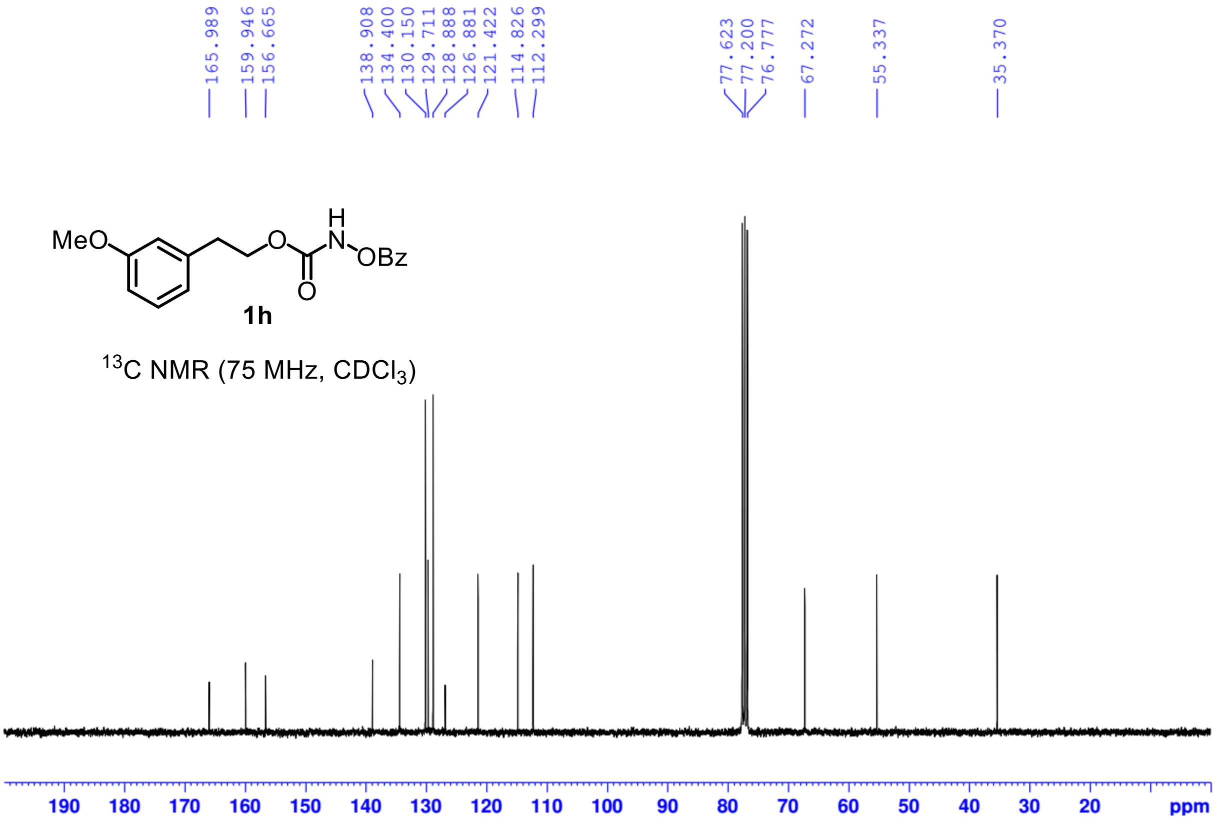
165.989
159.946
156.665
138.908
134.400
130.150
129.711
128.888
126.881
121.422
114.826
112.299
77.623
77.200
76.777
67.272
55.337
35.370
MeO
O
H
N
OBz
O
1h
13C NMR (75 MHz, CDCl3)
190 180 170 160 150 140 130 120 110 100 90 80 70 60 50 40 30 20 ppm

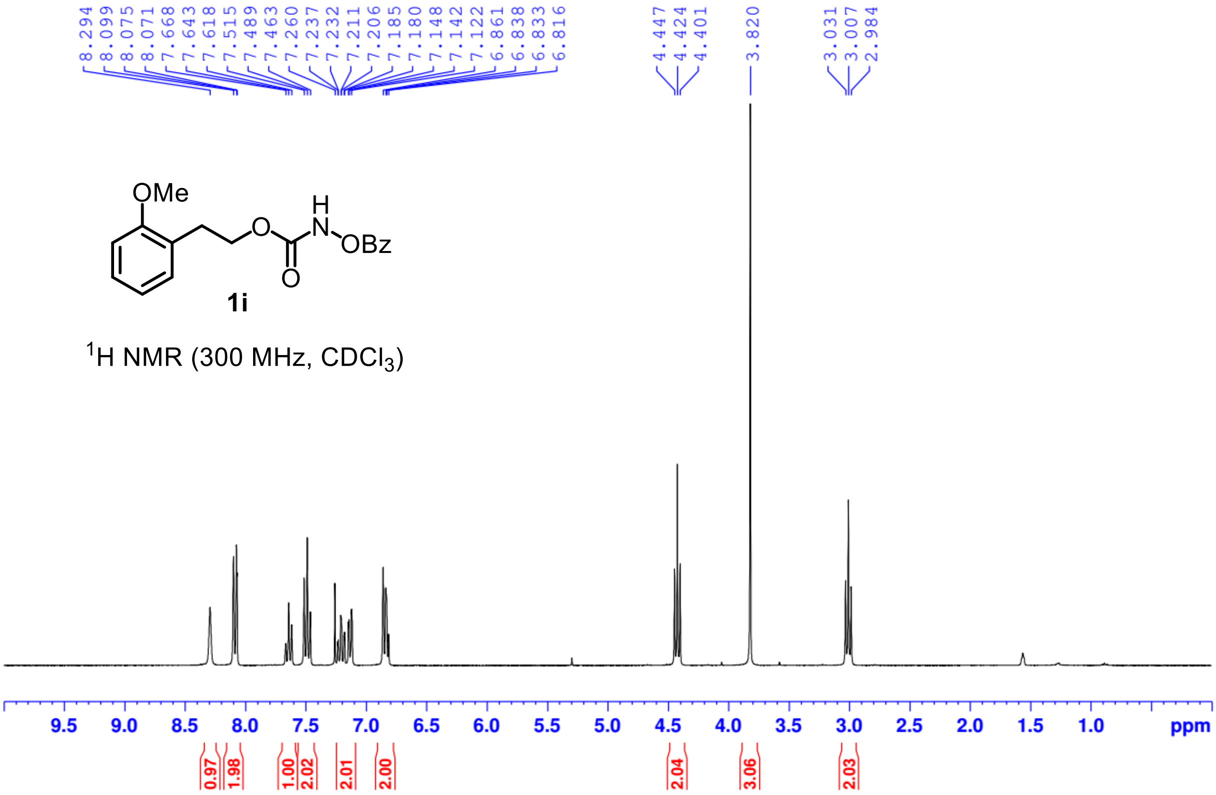
8.294
8.099
8.075
8.071
7.668
7.643
7.618
7.515
7.489
7.463
7.260
7.237
7.232
7.211
7.206
7.185
7.180
7.148
7.142
7.122
6.861
6.838
6.833
6.816
4.447
4.424
4.401
3.820
3.031
3.007
2.984
OMe
O
H
N
OBz
O
1i
1H NMR (300 MHz, CDCl3)
9.5 9.0 8.5 8.0 7.5 7.0 6.5 6.0 5.5 5.0 4.5 4.0 3.5 3.0 2.5 2.0 1.5 1.0 ppm
0.97
1.98
1.00
2.02
2.01
2.00
2.04
3.06
2.03

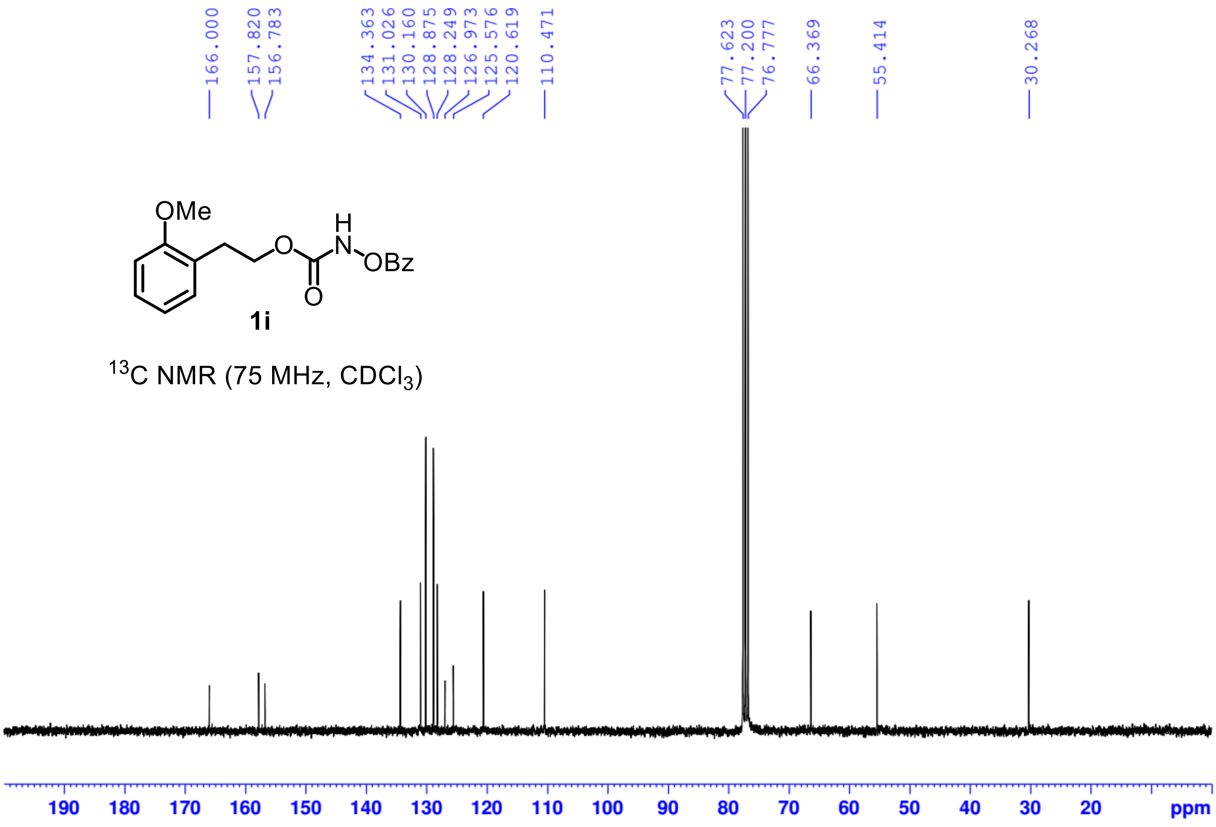

1i

^{13}C NMR (75 MHz, $CDCl_3$)

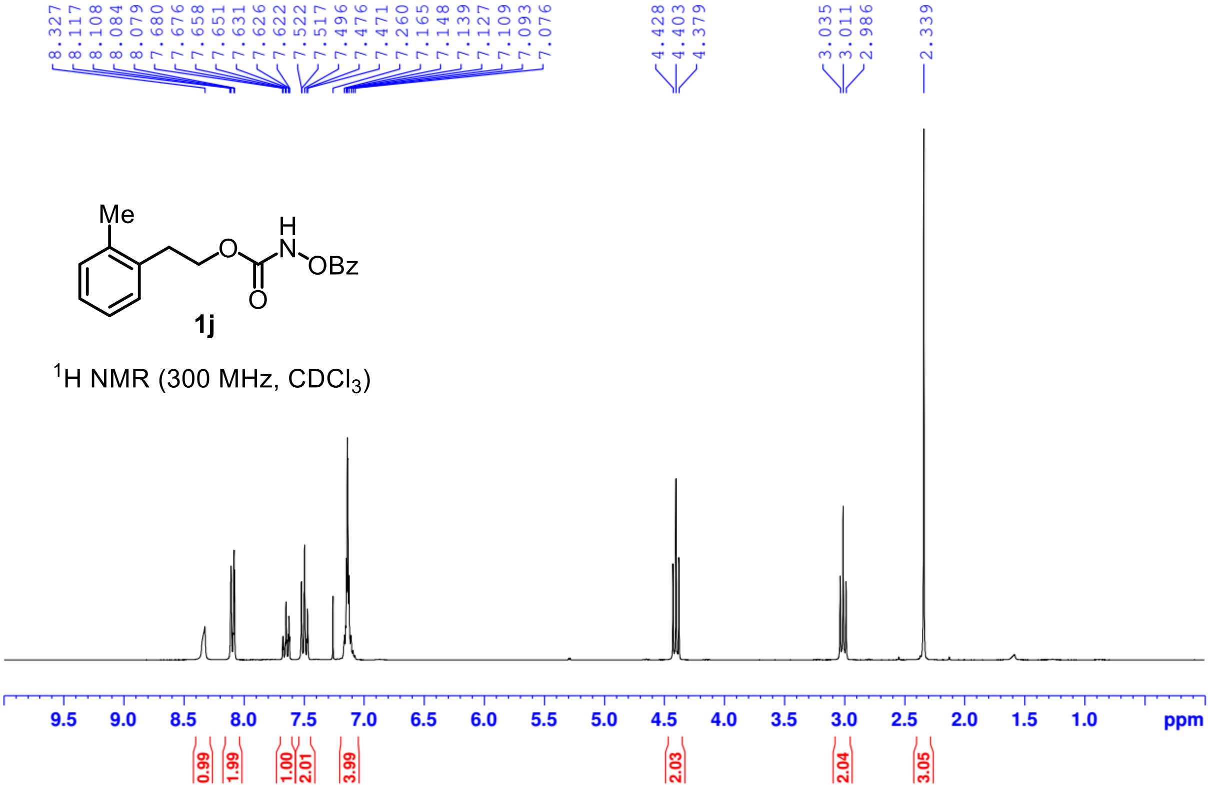

1j

^{1}H NMR (300 MHz, $CDCl_3$)

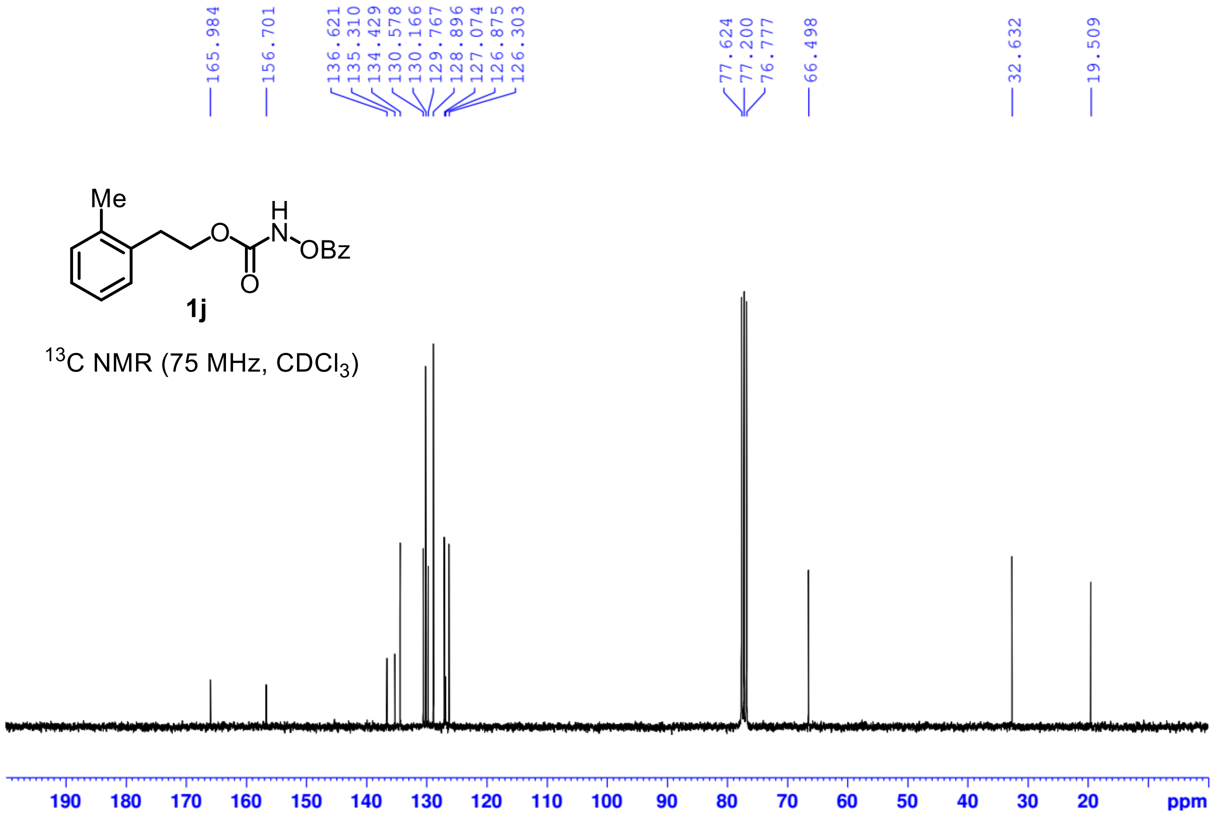
165.984
156.701
136.621
135.310
134.429
130.578
130.166
129.767
128.896
127.074
126.875
126.303
77.624
77.200
76.777
66.498
32.632
19.509
Me
H
N
O
OBz
O
1j
13C NMR (75 MHz, CDCl3)
190 180 170 160 150 140 130 120 110 100 90 80 70 60 50 40 30 20 ppm

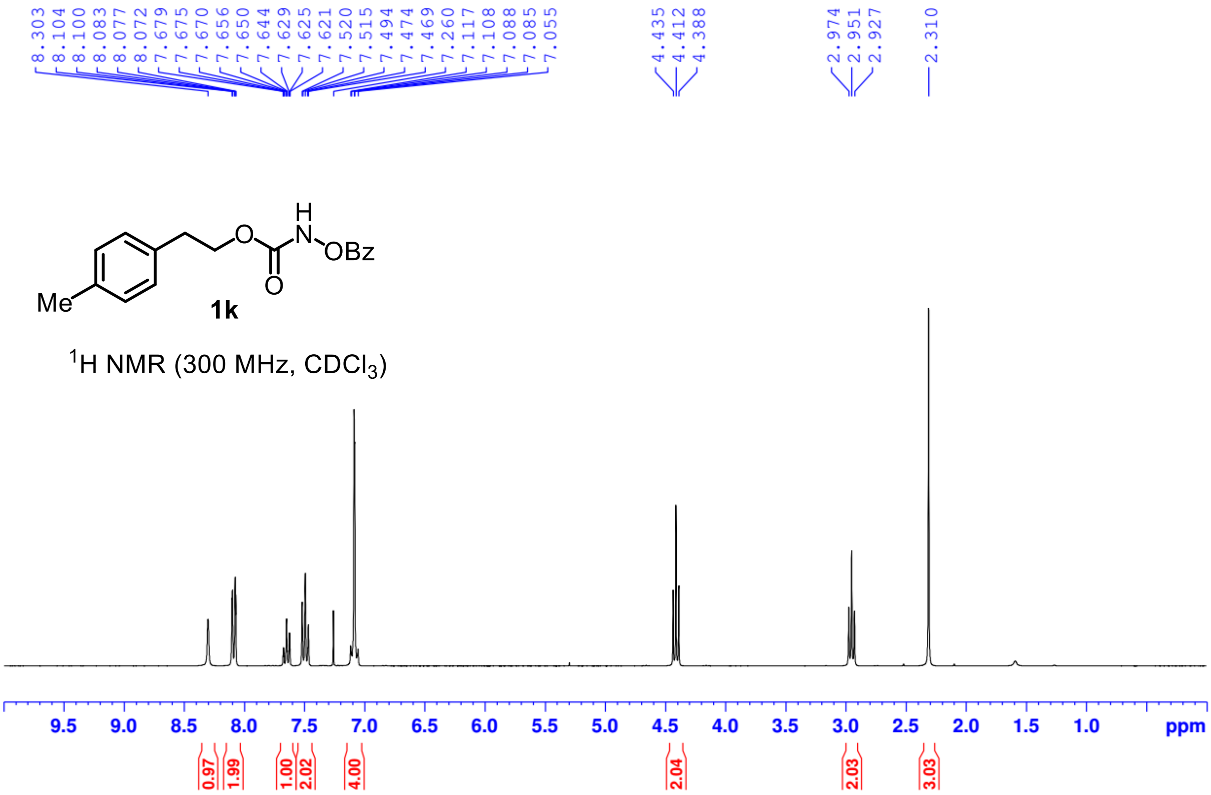
8.303
8.104
8.100
8.083
8.077
8.072
7.679
7.675
7.670
7.656
7.650
7.644
7.629
7.625
7.621
7.520
7.515
7.494
7.474
7.469
7.260
7.117
7.108
7.088
7.085
7.055
4.435
4.412
4.388
2.974
2.951
2.927
2.310
H
N
O
OBz
O
Me
1k
1H NMR (300 MHz, CDCl3)
9.5 9.0 8.5 8.0 7.5 7.0 6.5 6.0 5.5 5.0 4.5 4.0 3.5 3.0 2.5 2.0 1.5 1.0 ppm
0.97
1.99
1.00
2.02
4.00
2.04
2.03
3.03

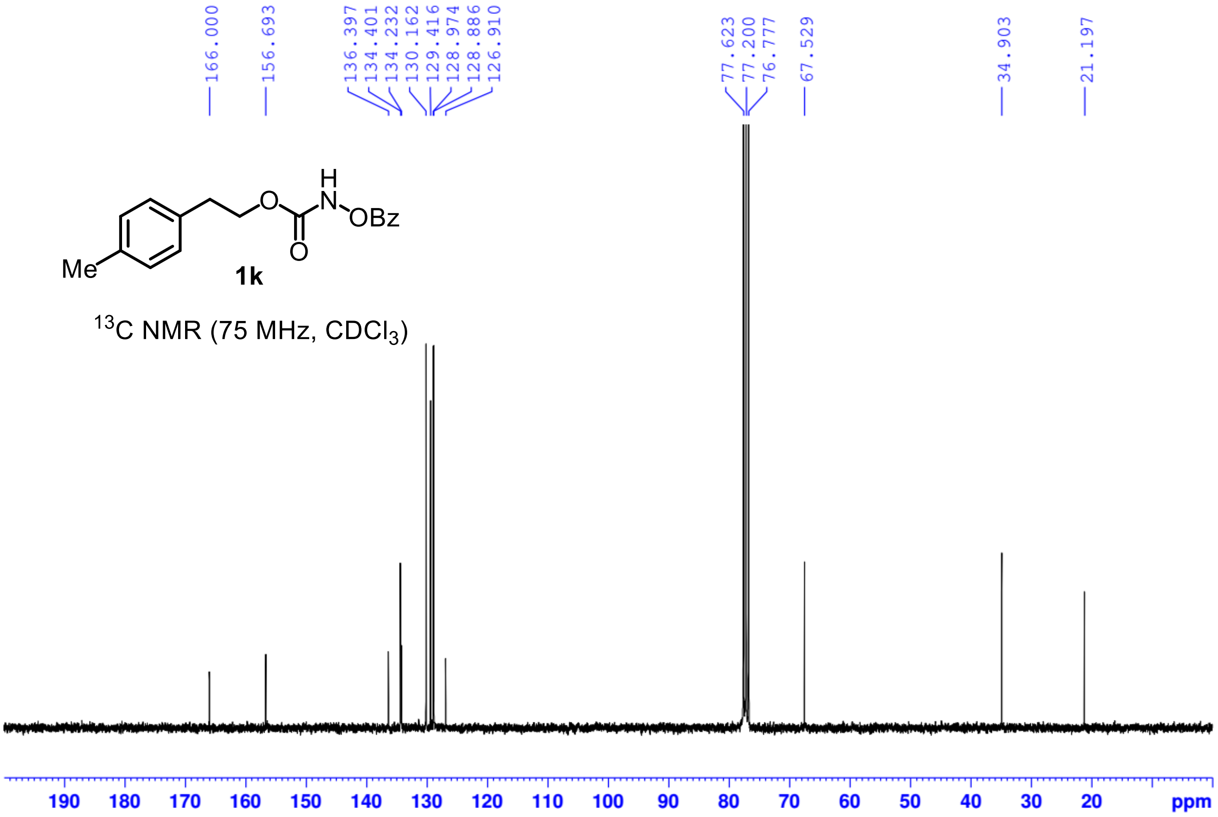
166.000
156.693
136.397
134.401
134.232
130.162
129.416
128.974
128.886
126.910
77.623
77.200
76.777
67.529
34.903
21.197
H
N
O
OBz
O
Me
1k
^{13}C NMR (75 MHz, $CDCl_3$)
ppm

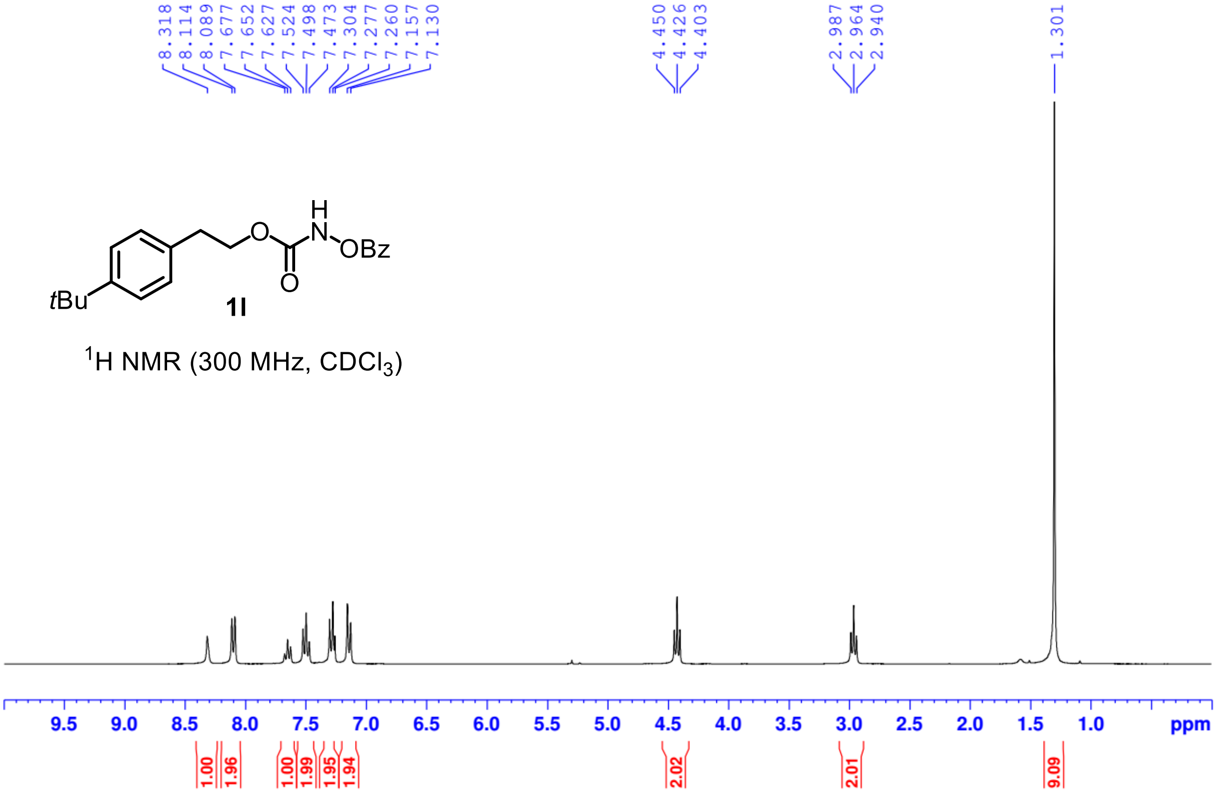
8.318
8.114
8.089
7.677
7.652
7.627
7.524
7.498
7.473
7.304
7.277
7.260
7.157
7.130
4.450
4.426
4.403
2.987
2.964
2.940
1.301
H
N
O
OBz
O
tBu
1l
^{1}H NMR (300 MHz, $CDCl_3$)
ppm
1.00
1.96
1.00
1.99
1.95
1.94
2.02
2.01
9.09

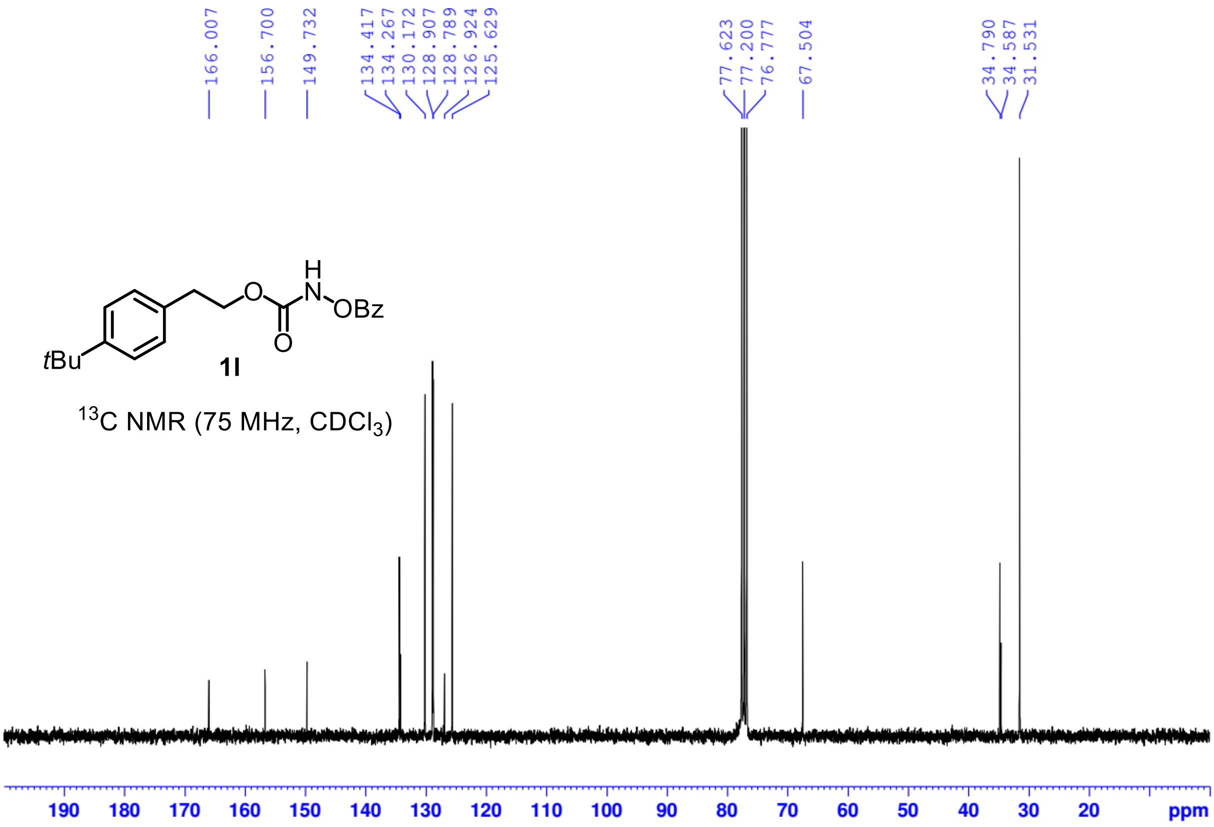

166.007
156.700
149.732
134.417
134.267
130.172
128.907
128.789
126.924
125.629
77.623
77.200
76.777
67.504
34.790
34.587
31.531
OBz
tBu
1l
13C NMR (75 MHz, CDCl3)
190
180
170
160
150
140
130
120
110
100
90
80
70
60
50
40
30
20
ppm

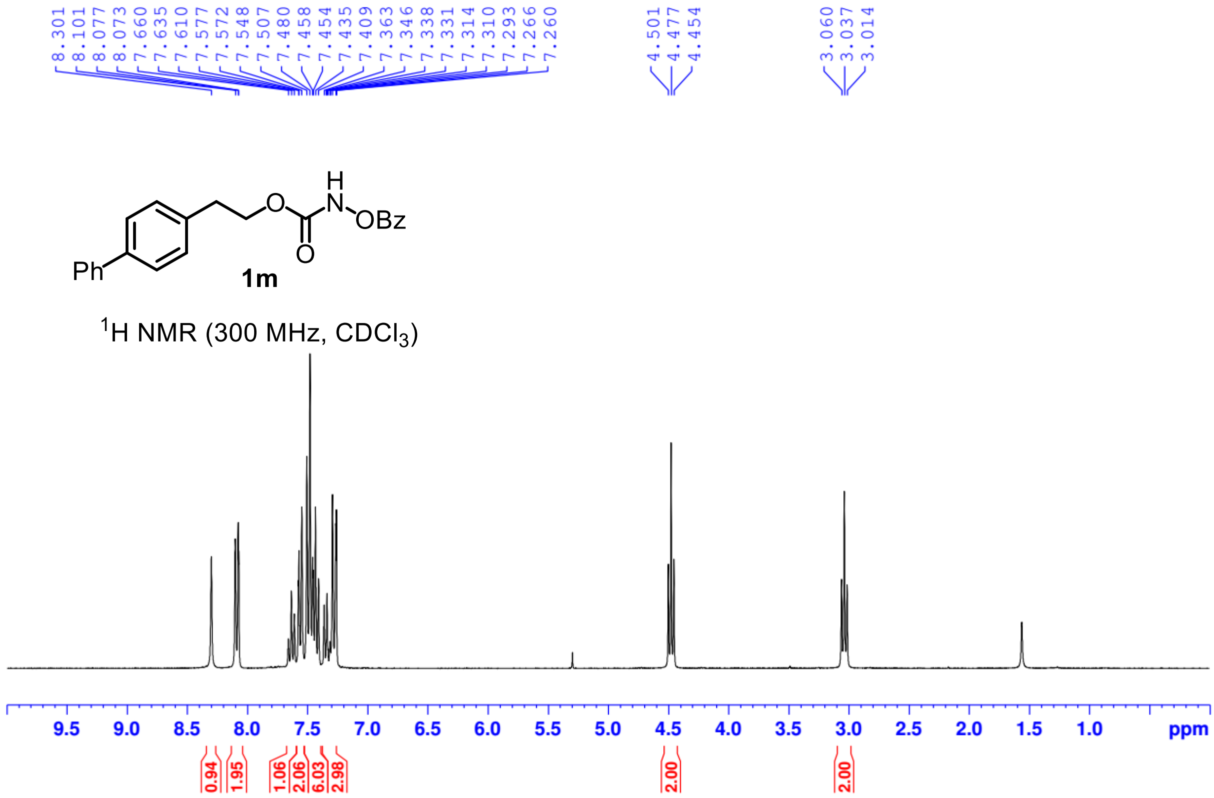

8.301
8.101
8.077
8.073
7.660
7.635
7.610
7.577
7.572
7.548
7.507
7.480
7.458
7.454
7.435
7.409
7.363
7.346
7.338
7.331
7.314
7.310
7.293
7.266
7.260
4.501
4.477
4.454
3.060
3.037
3.014
OBz
Ph
1m
1H NMR (300 MHz, CDCl3)
9.5
9.0
8.5
8.0
7.5
7.0
6.5
6.0
5.5
5.0
4.5
4.0
3.5
3.0
2.5
2.0
1.5
1.0
ppm
0.94
1.95
1.06
2.06
6.03
2.98
2.00
2.00

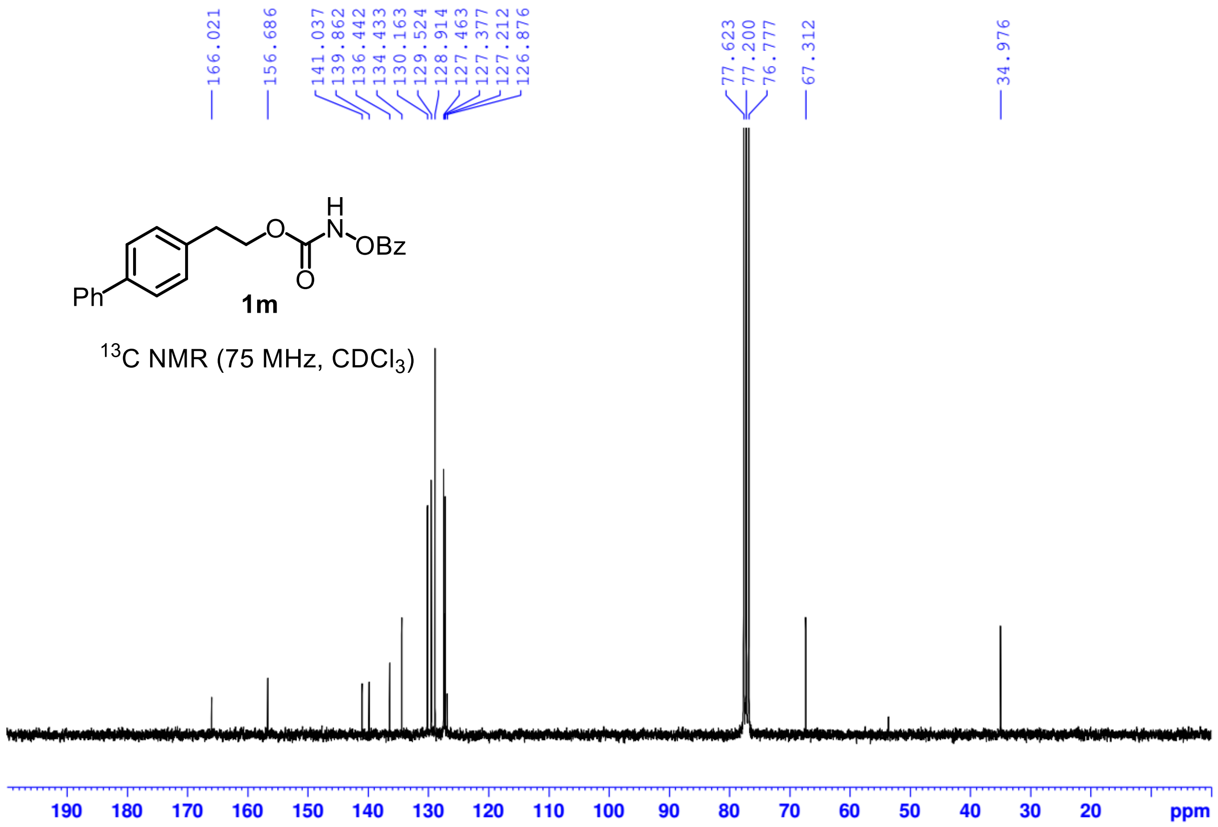
166.021
156.686
141.037
139.862
136.442
134.433
130.163
129.524
128.914
127.463
127.377
127.212
126.876
77.623
77.200
76.777
67.312
34.976
H
N
O
OBz
Ph
1m
13C NMR (75 MHz, CDCl3)
190 180 170 160 150 140 130 120 110 100 90 80 70 60 50 40 30 20 ppm

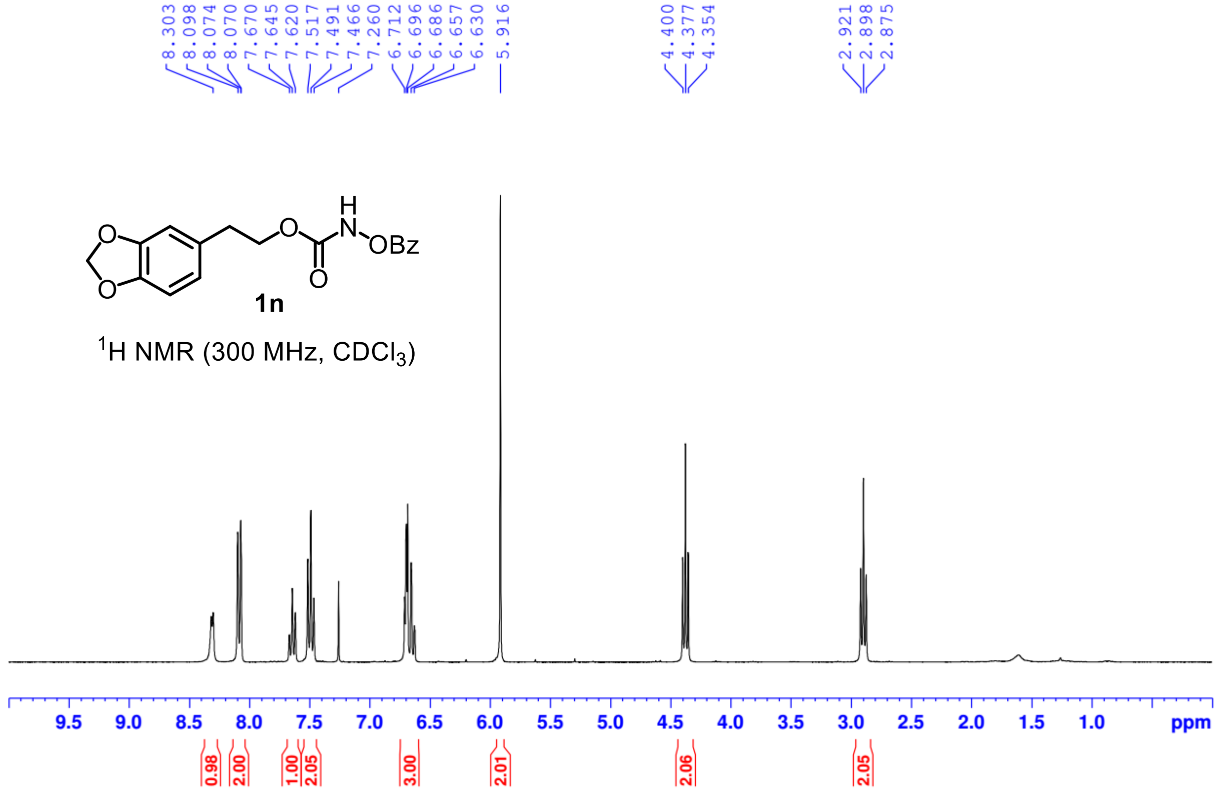
8.303
8.098
8.074
8.070
7.670
7.645
7.620
7.517
7.491
7.466
7.260
6.712
6.696
6.686
6.657
6.630
5.916
4.400
4.377
4.354
2.921
2.898
2.875
H
N
O
OBz
1n
1H NMR (300 MHz, CDCl3)
9.5 9.0 8.5 8.0 7.5 7.0 6.5 6.0 5.5 5.0 4.5 4.0 3.5 3.0 2.5 2.0 1.5 1.0 ppm
0.98
2.00
1.00
2.05
3.00
2.01
2.06
2.05

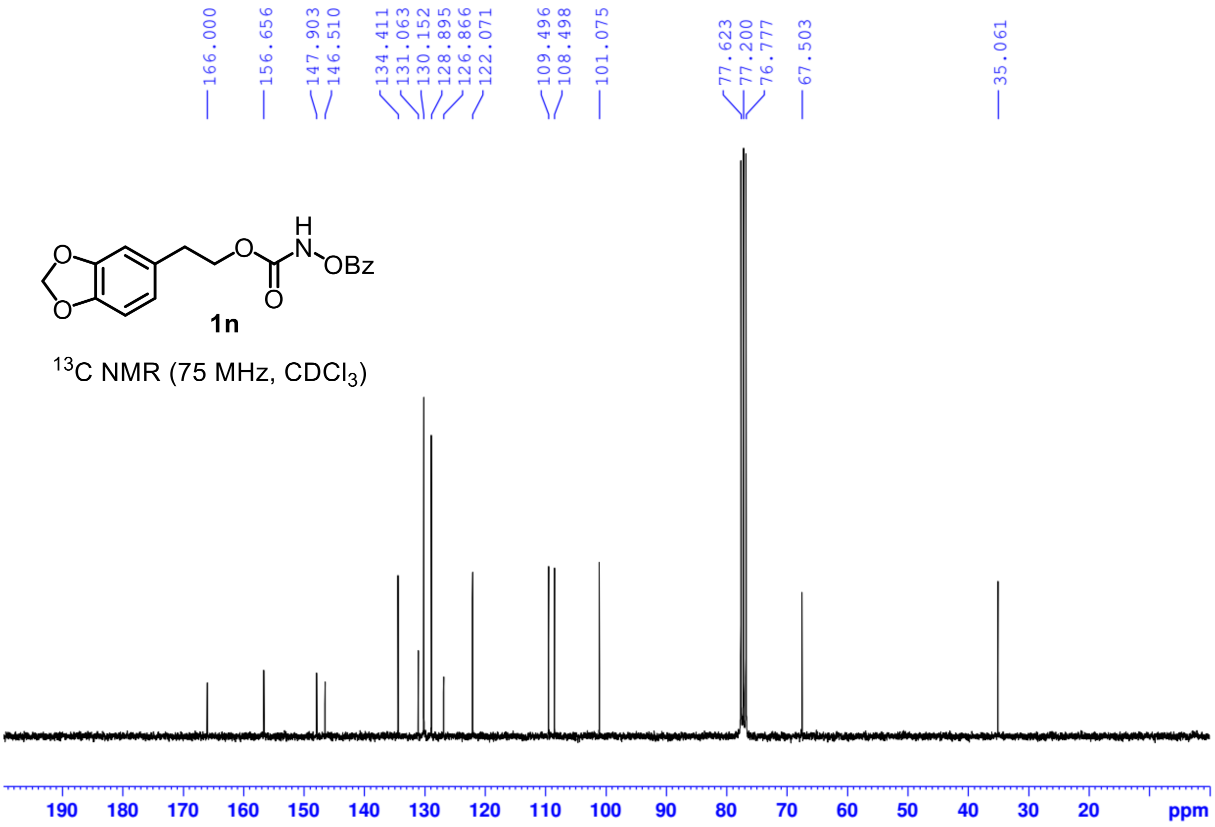

1n

^{13}C NMR (75 MHz, CDCl$_3$)

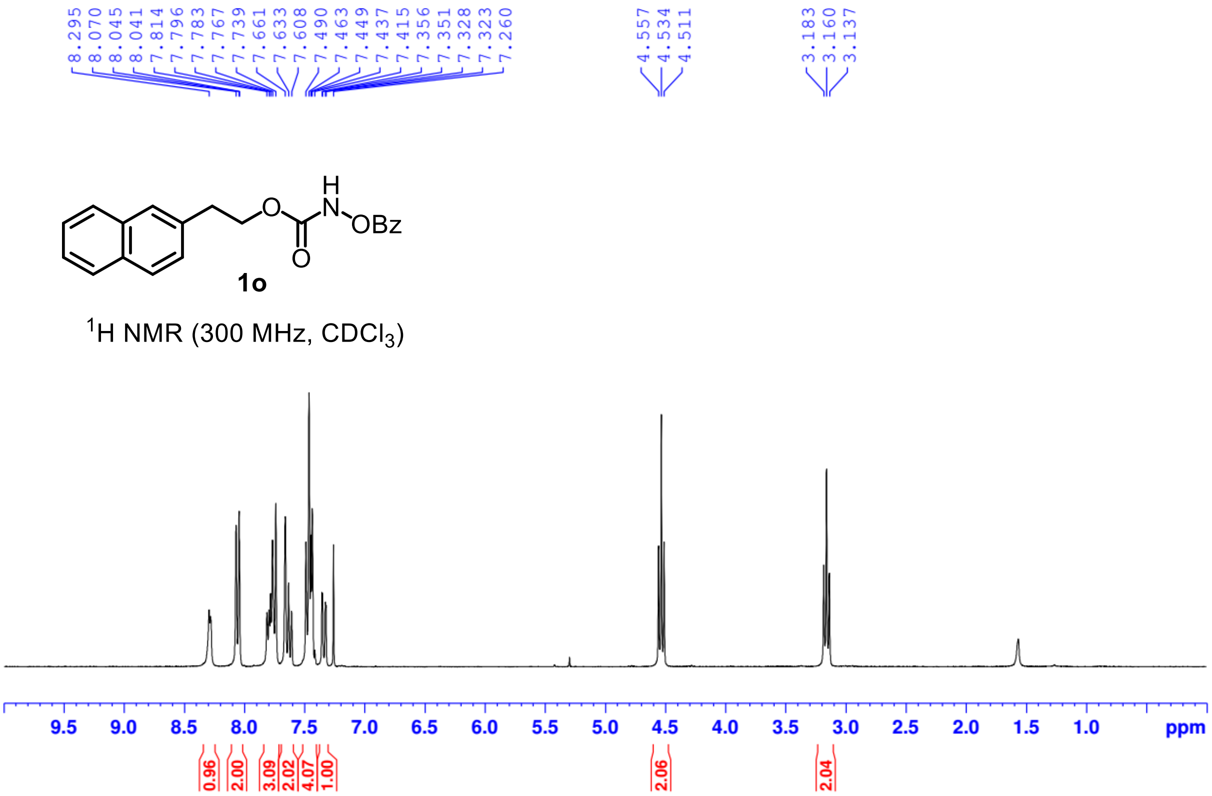

1o

^{1}H NMR (300 MHz, CDCl$_3$)

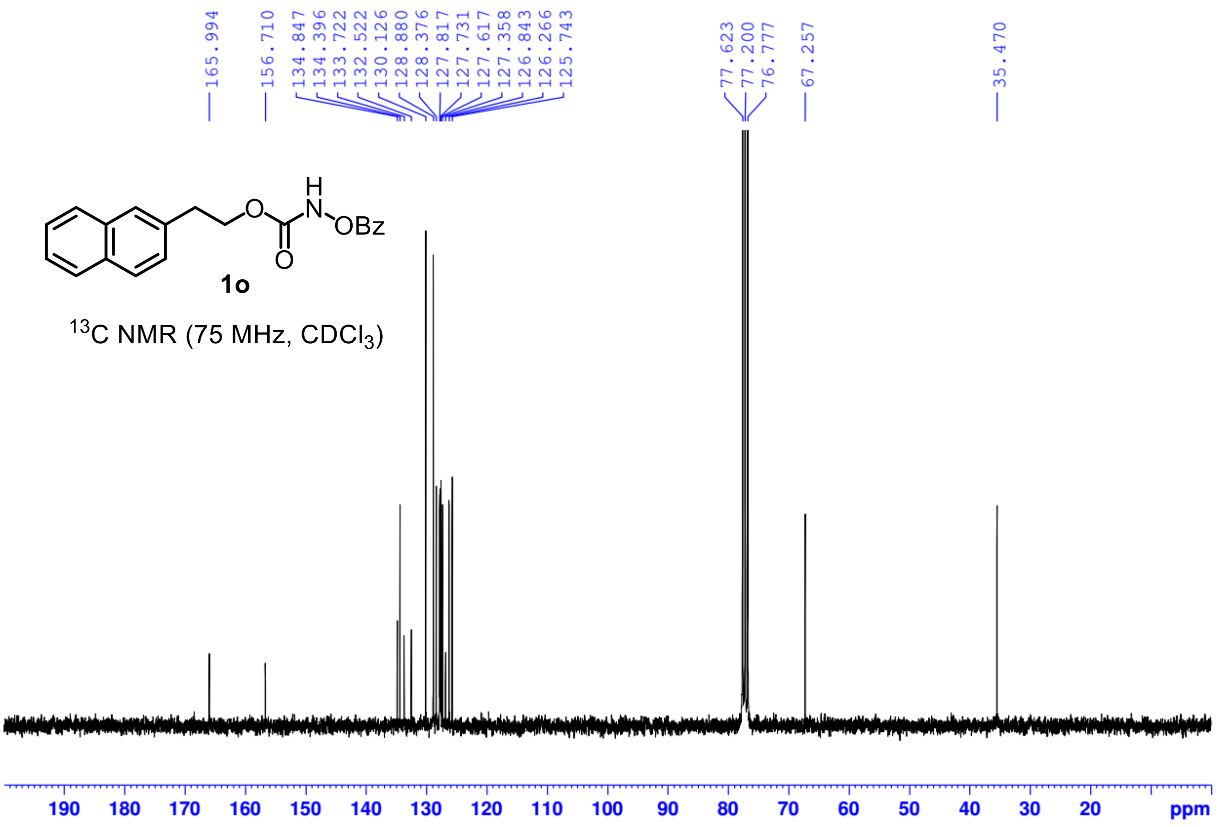
165.994
156.710
134.847
134.396
133.722
132.522
130.126
128.880
128.376
127.817
127.731
127.617
127.358
126.843
126.266
125.743
77.623
77.200
76.777
67.257
35.470
H
N
O
OBz
O
1o
^{13}C NMR (75 MHz, $CDCl_3$)
190 180 170 160 150 140 130 120 110 100 90 80 70 60 50 40 30 20 ppm

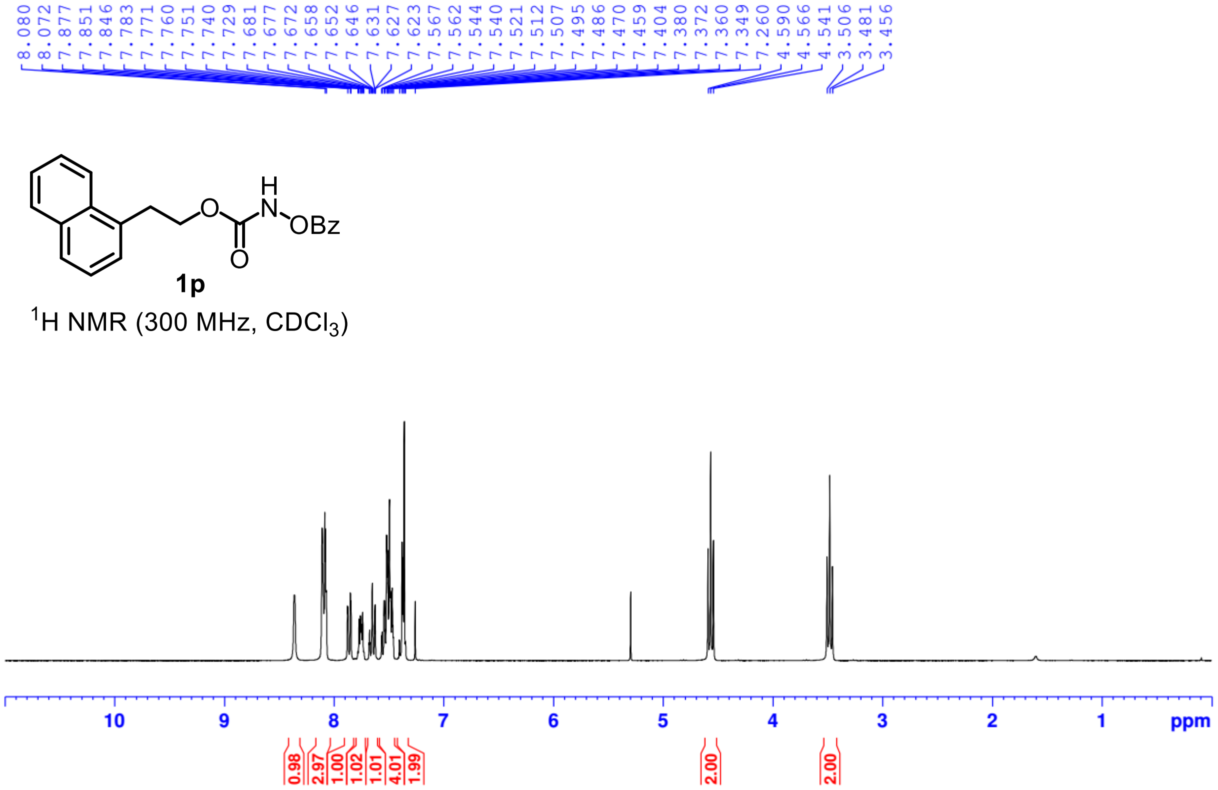
8.080
8.072
7.877
7.851
7.846
7.783
7.771
7.760
7.751
7.740
7.729
7.681
7.677
7.672
7.658
7.652
7.646
7.631
7.627
7.623
7.567
7.562
7.544
7.540
7.521
7.512
7.507
7.495
7.486
7.470
7.459
7.404
7.380
7.372
7.360
7.349
7.260
4.590
4.566
4.541
3.506
3.481
3.456
H
N
O
OBz
O
1p
^{1}H NMR (300 MHz, $CDCl_3$)
10 9 8 7 6 5 4 3 2 1 ppm
0.98
2.97
1.00
1.02
1.01
4.01
1.99
2.00
2.00

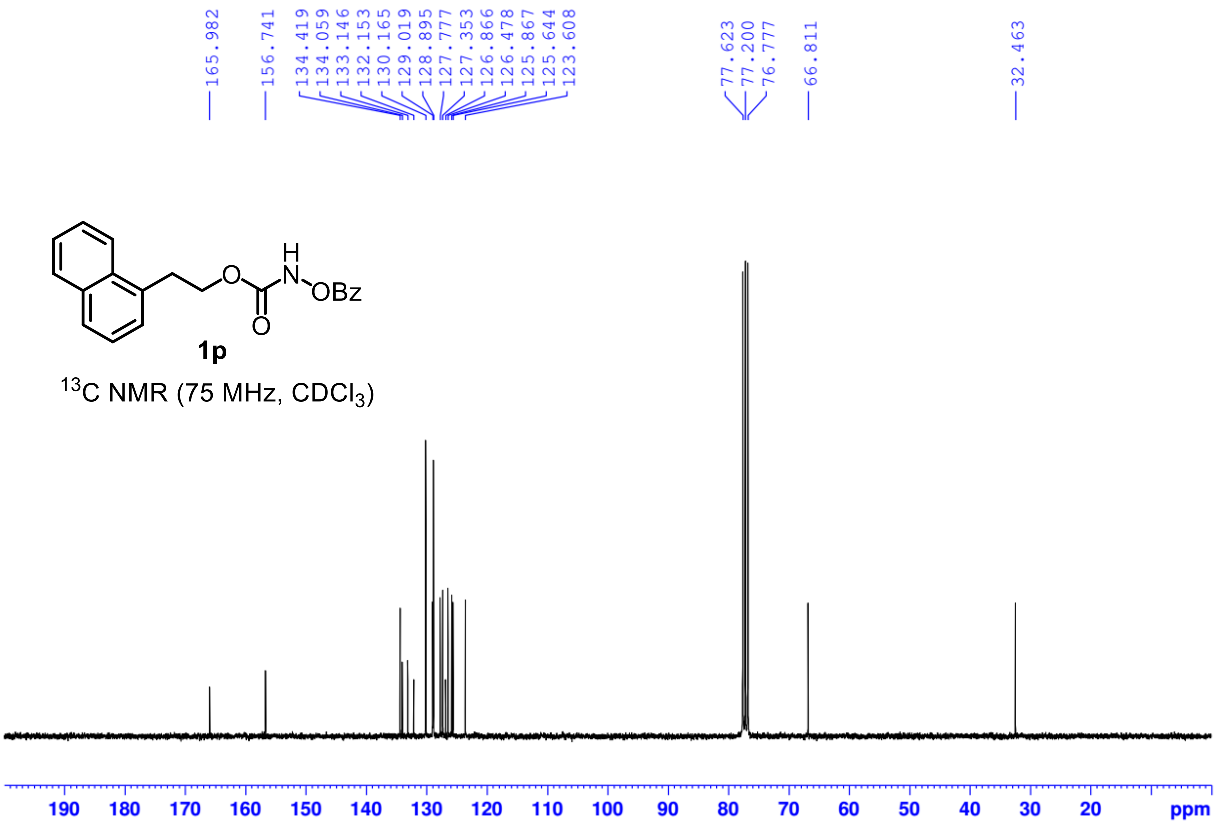
165.982
156.741
134.419
134.059
133.146
132.153
130.165
129.019
128.895
127.777
127.353
126.866
126.478
125.867
125.644
123.608
77.623
77.200
76.777
66.811
32.463
H
N
O
OBz
O
1p
13C NMR (75 MHz, CDCl3)
190 180 170 160 150 140 130 120 110 100 90 80 70 60 50 40 30 20 ppm

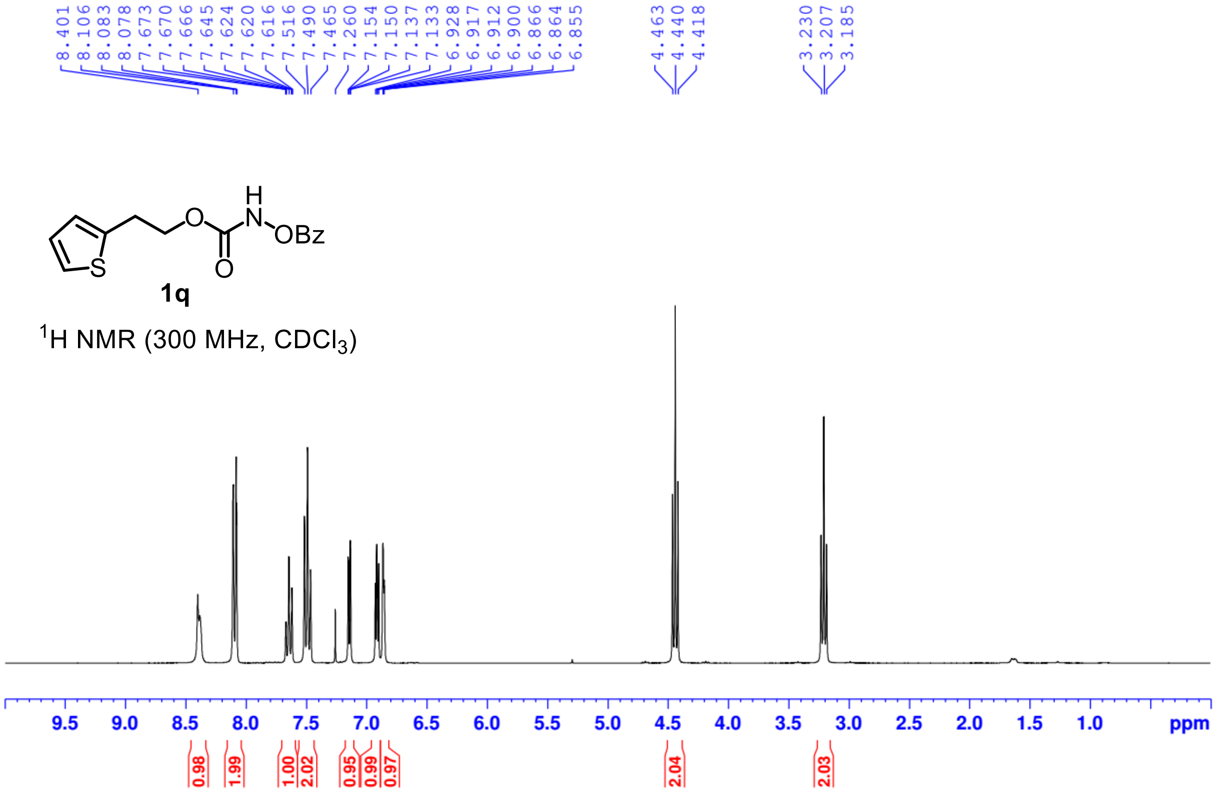
8.401
8.106
8.083
8.078
7.673
7.670
7.666
7.645
7.624
7.620
7.616
7.516
7.490
7.465
7.260
7.154
7.150
7.137
7.133
6.928
6.917
6.912
6.900
6.866
6.864
6.855
4.463
4.440
4.418
3.230
3.207
3.185
H
N
O
OBz
O
S
1q
1H NMR (300 MHz, CDCl3)
9.5 9.0 8.5 8.0 7.5 7.0 6.5 6.0 5.5 5.0 4.5 4.0 3.5 3.0 2.5 2.0 1.5 1.0 ppm
0.98
1.99
1.00
2.02
0.95
0.99
0.97
2.04
2.03

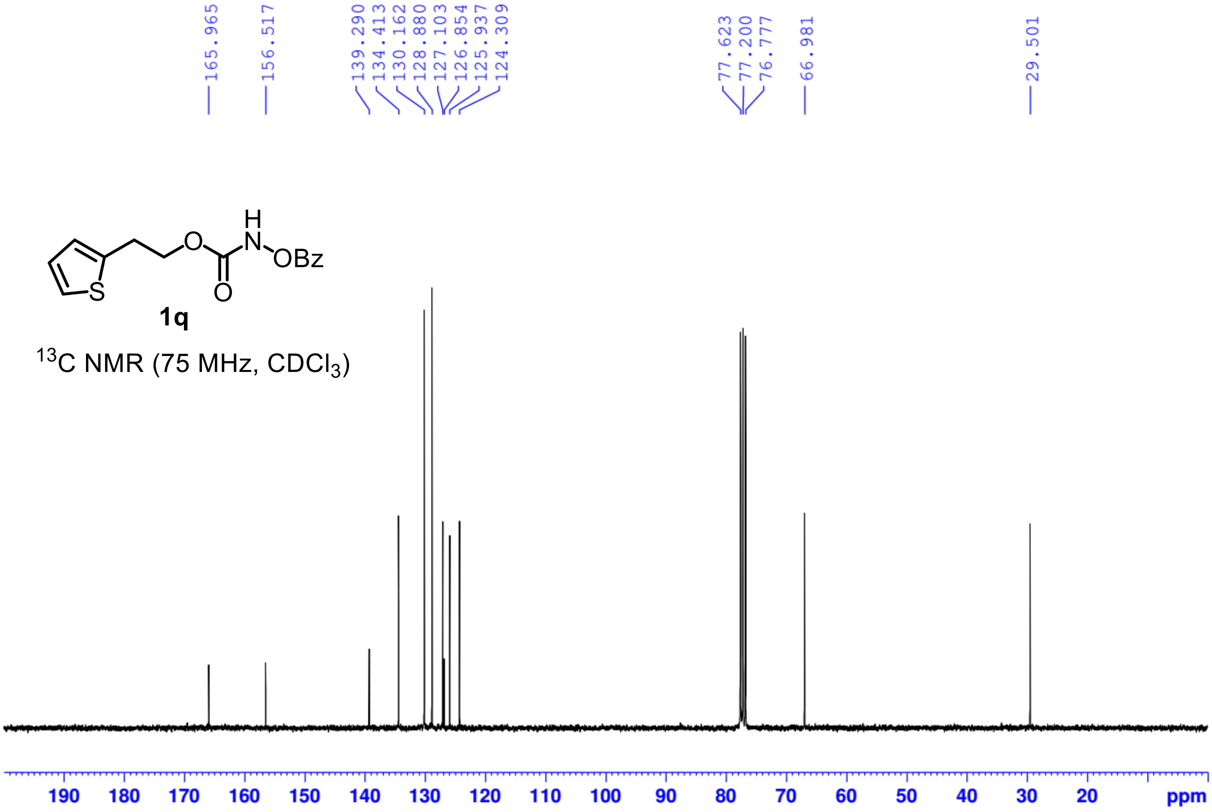

OBz
1q
^{13}C NMR (75 MHz, CDCl$_3$)
165.965
156.517
139.290
134.413
130.162
128.880
127.103
126.854
125.937
124.309
77.623
77.200
76.777
66.981
29.501
ppm

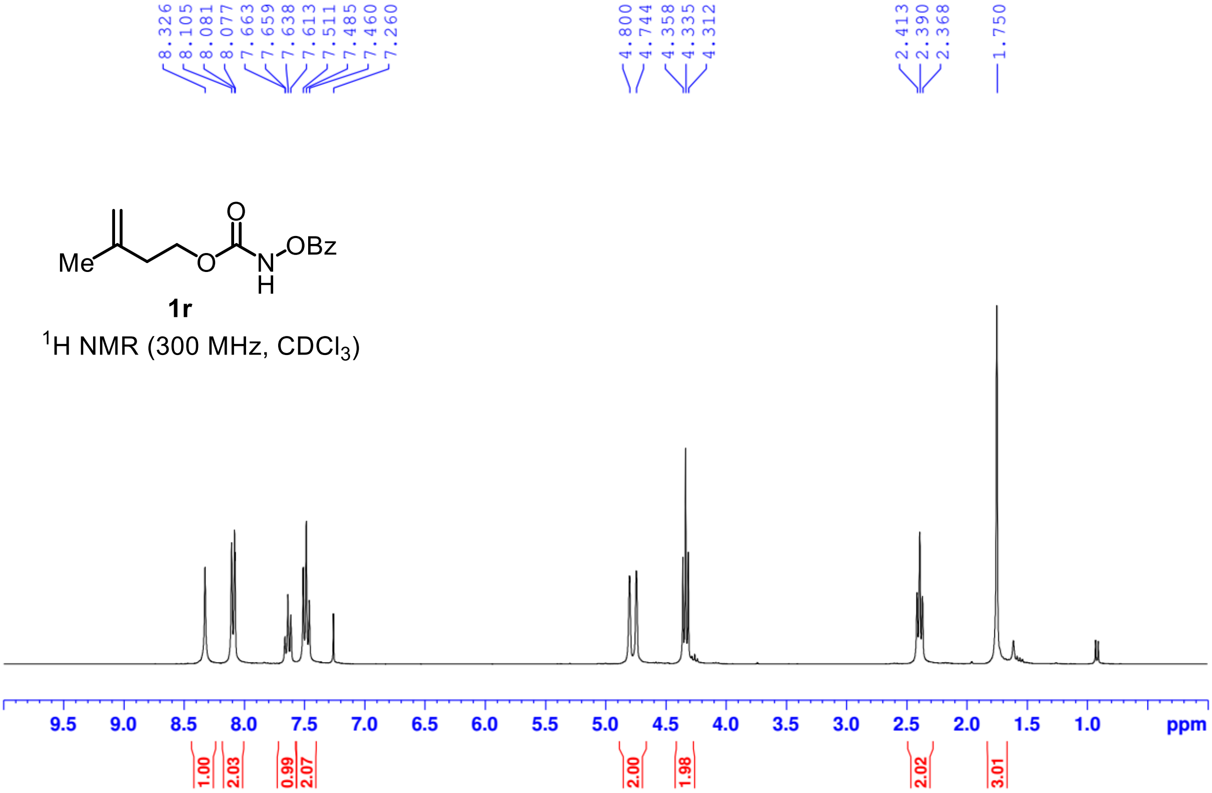

Me
OBz
1r
^{1}H NMR (300 MHz, CDCl$_3$)
8.326
8.105
8.081
8.077
7.663
7.659
7.638
7.613
7.511
7.485
7.460
7.260
4.800
4.744
4.358
4.335
4.312
2.413
2.390
2.368
1.750
ppm
1.00
2.03
0.99
2.07
2.00
1.98
2.02
3.01

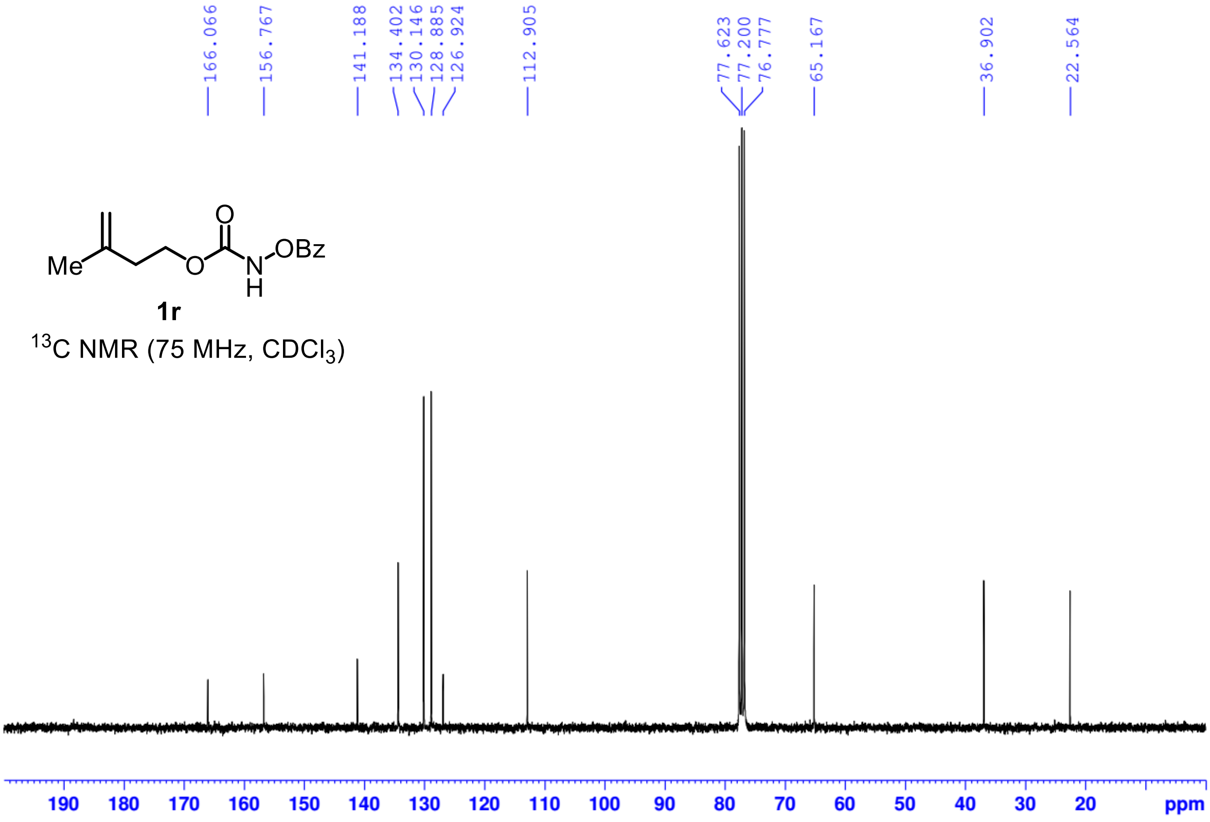
166.066
156.767
141.188
134.402
130.146
128.885
126.924
112.905
77.623
77.200
76.777
65.167
36.902
22.564
Me
O
O
N
OBz
H
1r
13C NMR (75 MHz, CDCl3)
190 180 170 160 150 140 130 120 110 100 90 80 70 60 50 40 30 20 ppm

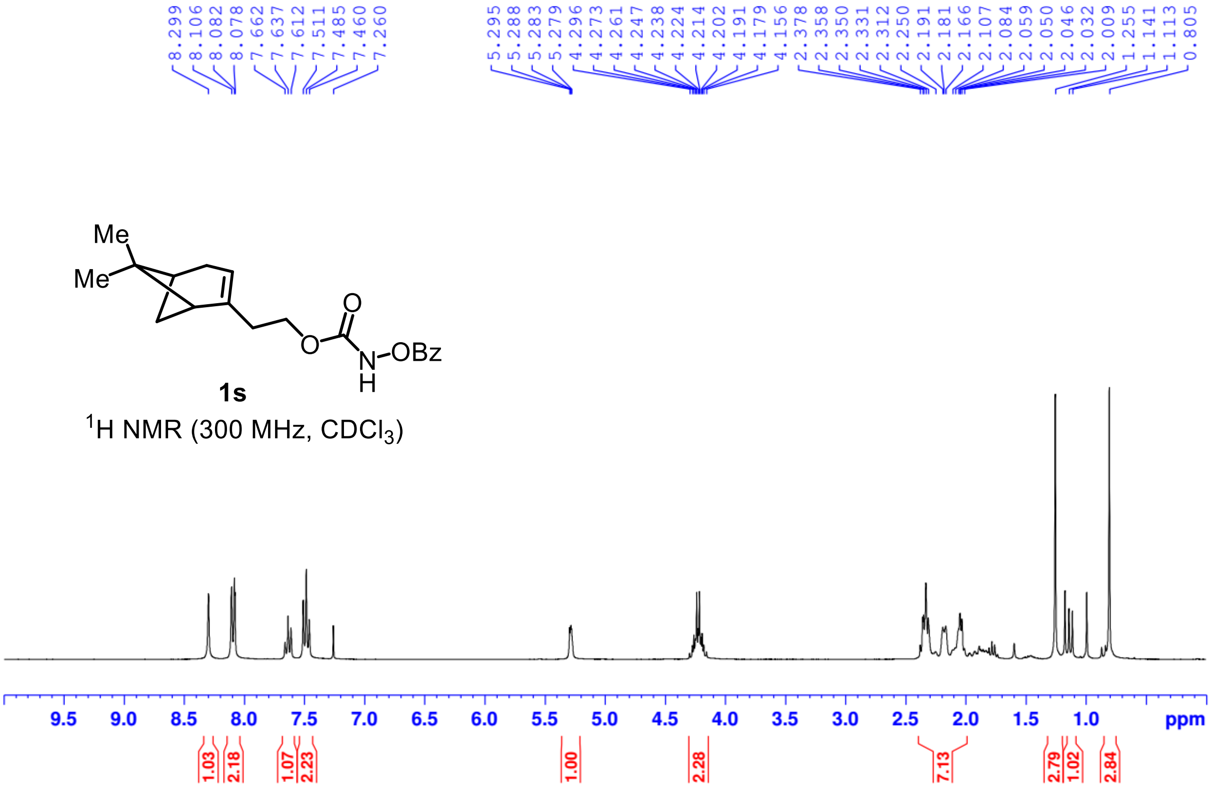
8.299
8.106
8.082
8.078
7.662
7.637
7.612
7.511
7.485
7.460
7.260
5.295
5.288
5.283
5.279
4.296
4.273
4.261
4.247
4.238
4.224
4.214
4.202
4.191
4.179
4.156
2.378
2.358
2.350
2.331
2.312
2.250
2.191
2.181
2.166
2.107
2.084
2.059
2.050
2.046
2.032
2.009
1.255
1.141
1.113
0.805
Me
Me
O
O
N
OBz
H
1s
1H NMR (300 MHz, CDCl3)
9.5 9.0 8.5 8.0 7.5 7.0 6.5 6.0 5.5 5.0 4.5 4.0 3.5 3.0 2.5 2.0 1.5 1.0 ppm
1.03
2.18
1.07
2.23
1.00
2.28
7.13
2.79
1.02
2.84

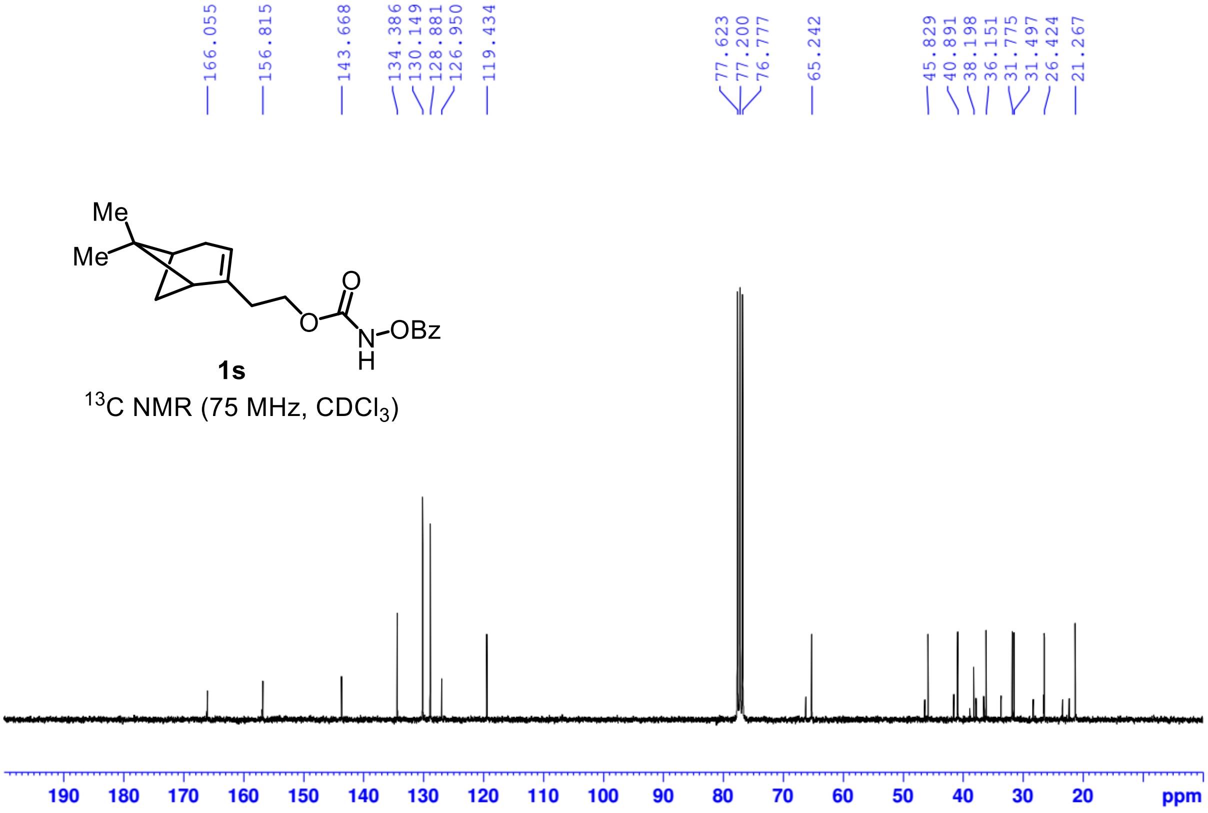

166.055
156.815
143.668
134.386
130.149
128.881
126.950
119.434
77.623
77.200
76.777
65.242
45.829
40.891
38.198
36.151
31.775
31.497
26.424
21.267
Me
Me
O
N
OBz
H
O
1s
^{13}C NMR (75 MHz, CDCl$_3$)
190
180
170
160
150
140
130
120
110
100
90
80
70
60
50
40
30
20
ppm

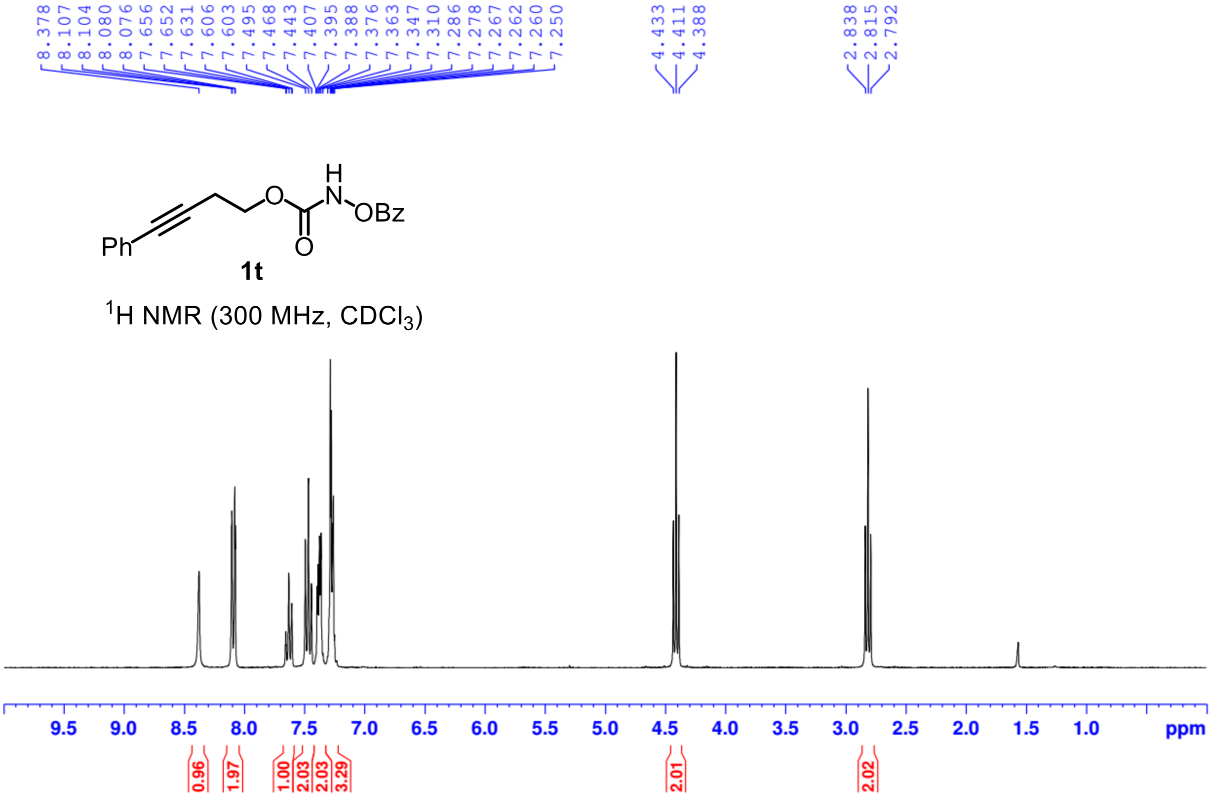

8.378
8.107
8.104
8.080
8.076
7.656
7.652
7.631
7.606
7.603
7.495
7.468
7.443
7.407
7.395
7.388
7.376
7.363
7.347
7.310
7.286
7.278
7.267
7.262
7.260
7.250
4.433
4.411
4.388
2.838
2.815
2.792
H
N
O
OBz
Ph
O
1t
^{1}H NMR (300 MHz, CDCl$_3$)
9.5
9.0
8.5
8.0
7.5
7.0
6.5
6.0
5.5
5.0
4.5
4.0
3.5
3.0
2.5
2.0
1.5
1.0
ppm
0.96
1.97
1.00
2.03
2.03
3.29
2.01
2.02

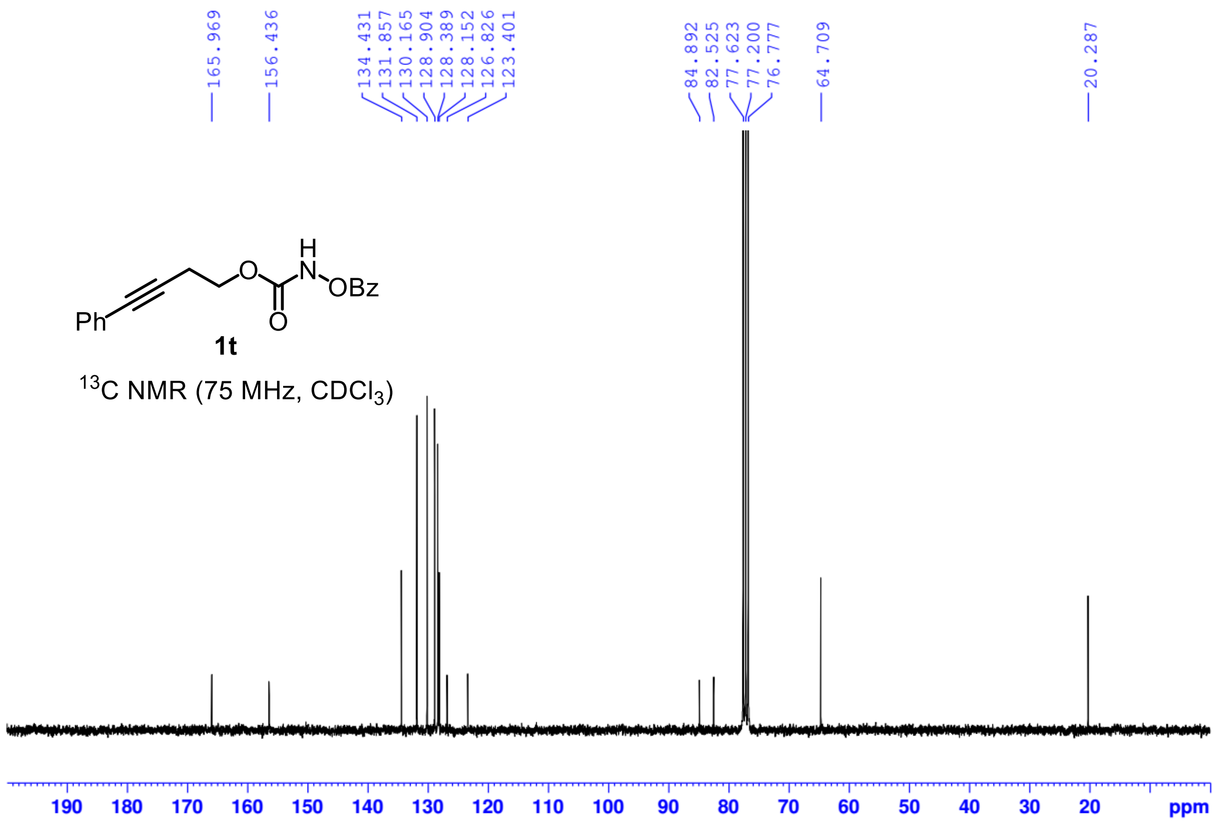
165.969
156.436
134.431
131.857
130.165
128.904
128.389
128.152
126.826
123.401
84.892
82.525
77.623
77.200
76.777
64.709
20.287
H
N
O
OBz
Ph
O
1t
^{13}C NMR (75 MHz, CDCl$_3$)
190 180 170 160 150 140 130 120 110 100 90 80 70 60 50 40 30 20 ppm

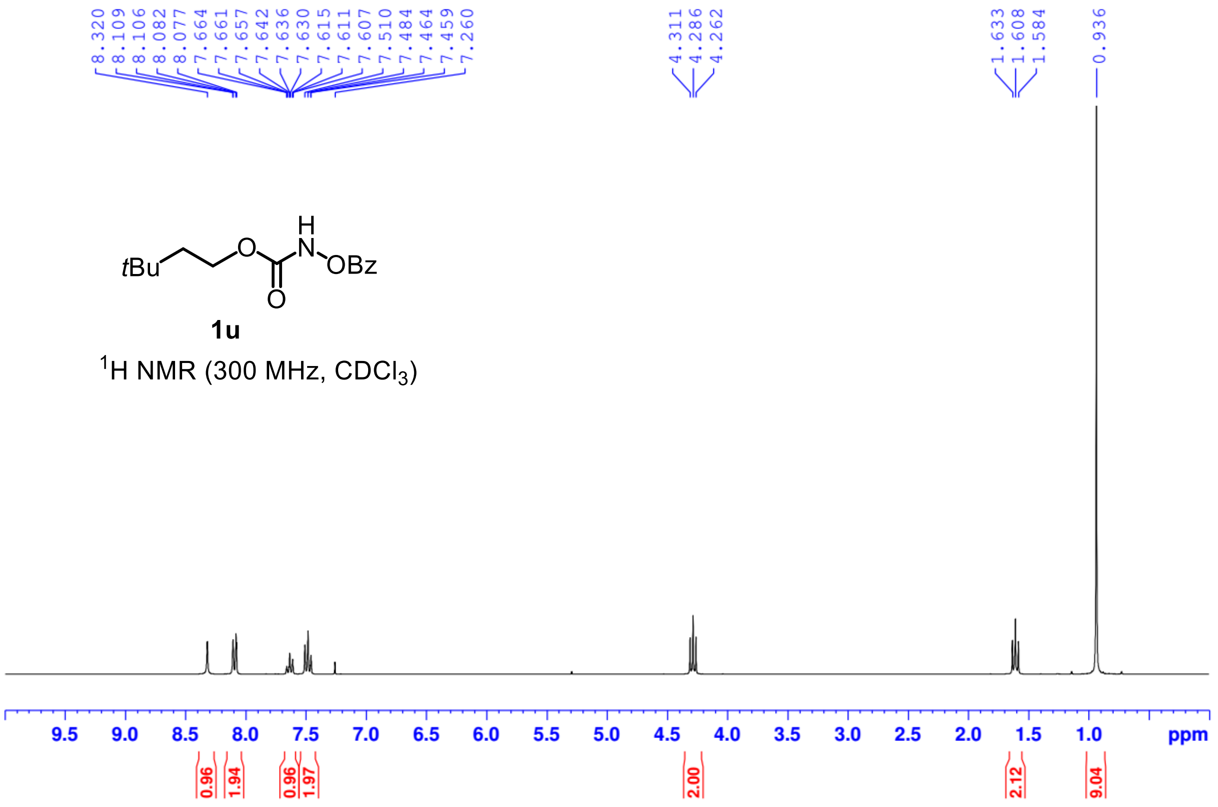
8.320
8.109
8.106
8.082
8.077
7.664
7.661
7.657
7.642
7.636
7.630
7.615
7.611
7.607
7.510
7.484
7.464
7.459
7.260
4.311
4.286
4.262
1.633
1.608
1.584
0.936
H
N
tBu
O
OBz
O
1u
^{1}H NMR (300 MHz, CDCl$_3$)
9.5 9.0 8.5 8.0 7.5 7.0 6.5 6.0 5.5 5.0 4.5 4.0 3.5 3.0 2.5 2.0 1.5 1.0 ppm
0.96
1.94
0.96
1.97
2.00
2.12
9.04

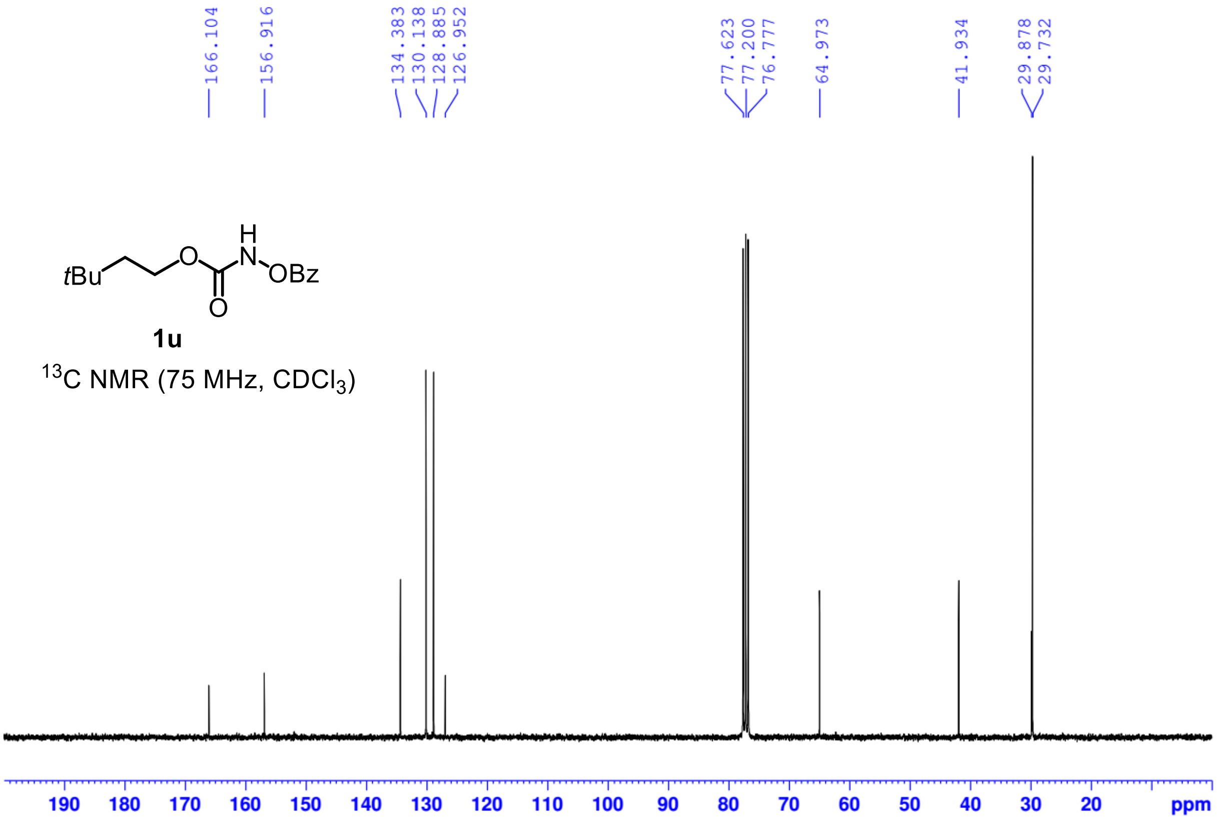
166.104
156.916
134.383
130.138
128.885
126.952
77.623
77.200
76.777
64.973
41.934
29.878
29.732
tBu
O
H
N
OBz
O
1u
13C NMR (75 MHz, CDCl3)
190
180
170
160
150
140
130
120
110
100
90
80
70
60
50
40
30
20
ppm

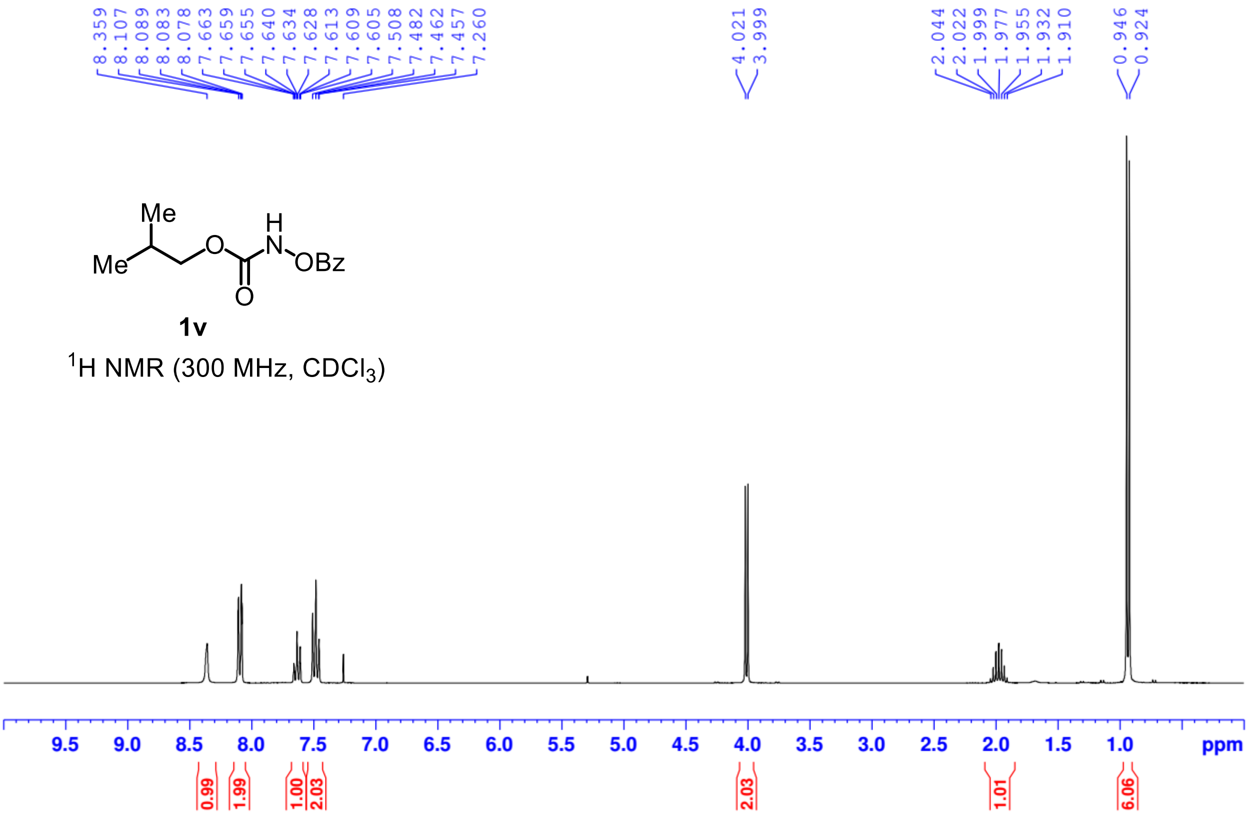
8.359
8.107
8.089
8.083
8.078
7.663
7.659
7.655
7.640
7.634
7.628
7.613
7.609
7.605
7.508
7.482
7.462
7.457
7.260
4.021
3.999
2.044
2.022
1.999
1.977
1.955
1.932
1.910
0.946
0.924
Me
Me
O
H
N
OBz
O
1v
1H NMR (300 MHz, CDCl3)
9.5
9.0
8.5
8.0
7.5
7.0
6.5
6.0
5.5
5.0
4.5
4.0
3.5
3.0
2.5
2.0
1.5
1.0
ppm
0.99
1.99
1.00
2.03
2.03
1.01
6.06

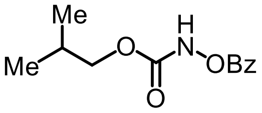

1v

^{13}C NMR (75 MHz, $CDCl_3$)

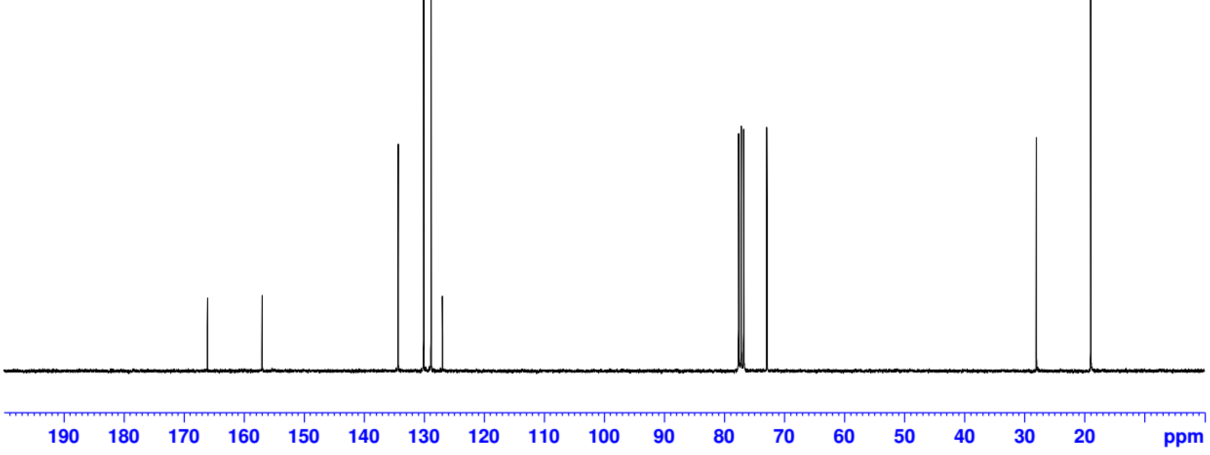

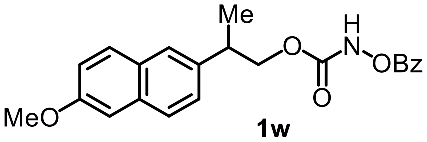

1w

^{1}H NMR (300 MHz, $CDCl_3$)

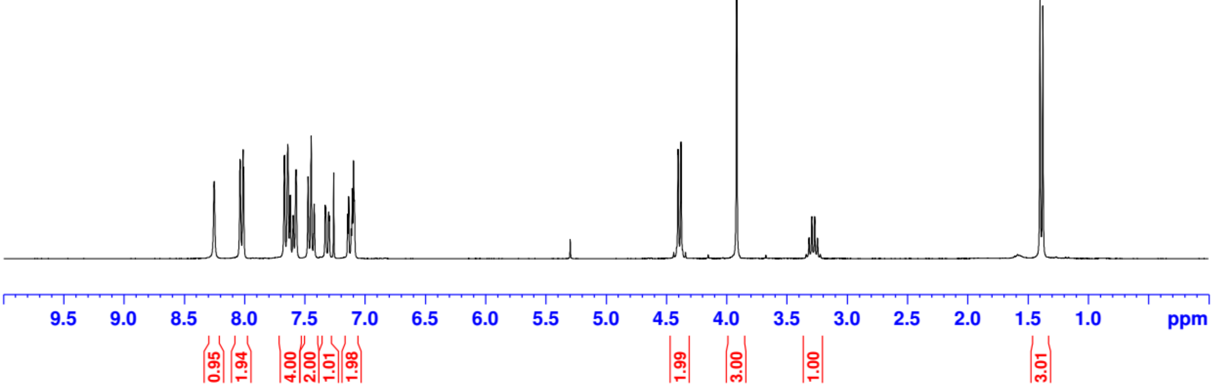

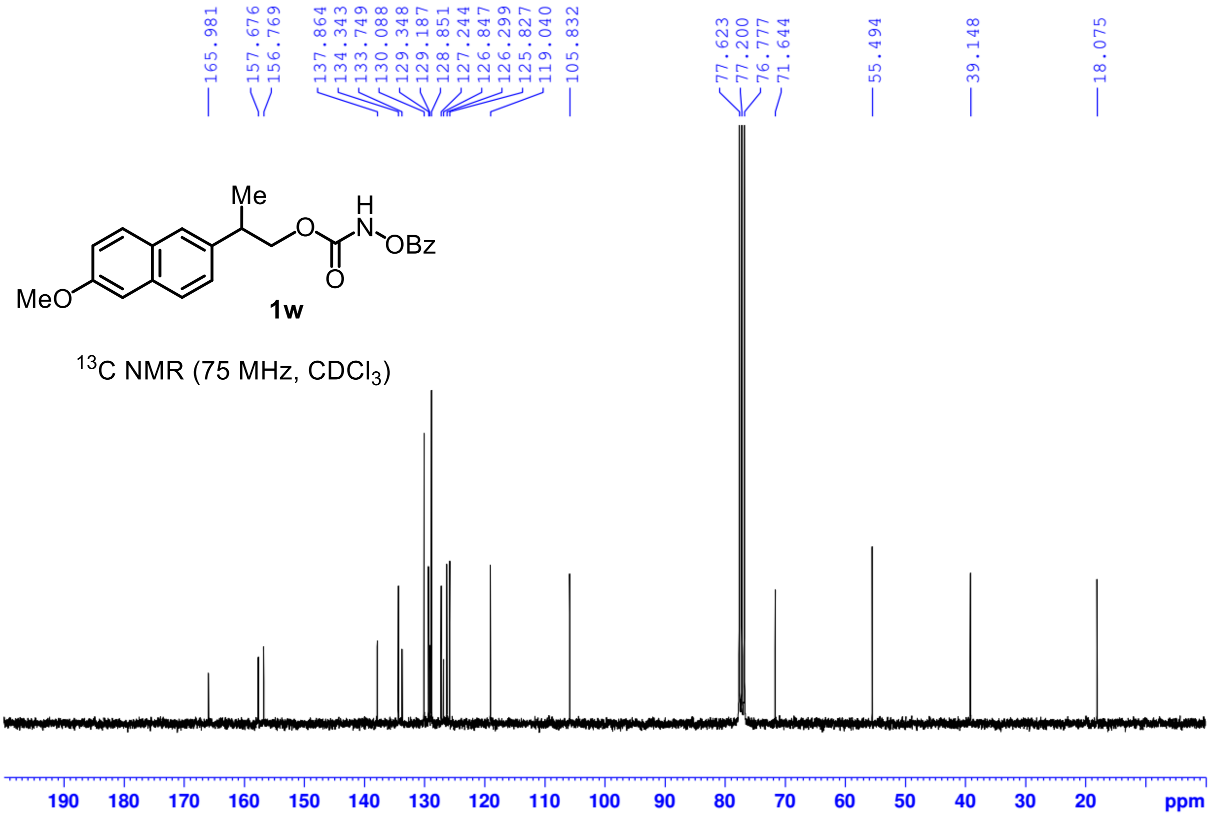
165.981
157.676
156.769
137.864
134.343
133.749
130.088
129.348
129.187
128.851
127.244
126.847
126.299
125.827
119.040
105.832
77.623
77.200
76.777
71.644
55.494
39.148
18.075
Me
O
H
N
OBz
MeO
O
1w
^{13}C NMR (75 MHz, $CDCl_3$)
190
180
170
160
150
140
130
120
110
100
90
80
70
60
50
40
30
20
ppm

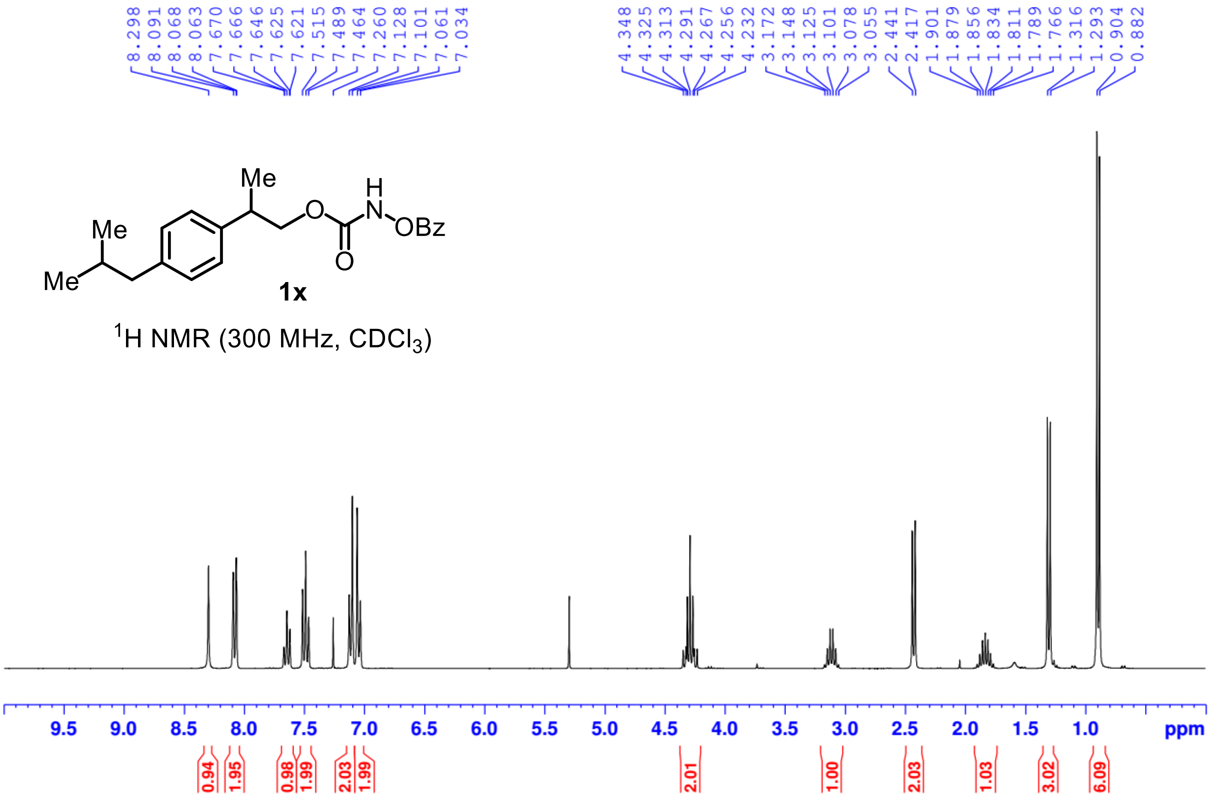
8.298
8.091
8.068
8.063
7.670
7.666
7.646
7.625
7.621
7.515
7.489
7.464
7.260
7.128
7.101
7.061
7.034
4.348
4.325
4.313
4.291
4.267
4.256
4.232
3.172
3.148
3.125
3.101
3.078
3.055
2.441
2.417
1.901
1.879
1.856
1.834
1.811
1.789
1.766
1.316
1.293
0.904
0.882
Me
Me
Me
O
H
N
OBz
O
1x
^{1}H NMR (300 MHz, $CDCl_3$)
9.5
9.0
8.5
8.0
7.5
7.0
6.5
6.0
5.5
5.0
4.5
4.0
3.5
3.0
2.5
2.0
1.5
1.0
ppm
0.94
1.95
0.98
1.99
2.03
1.99
2.01
1.00
2.03
1.03
3.02
6.09

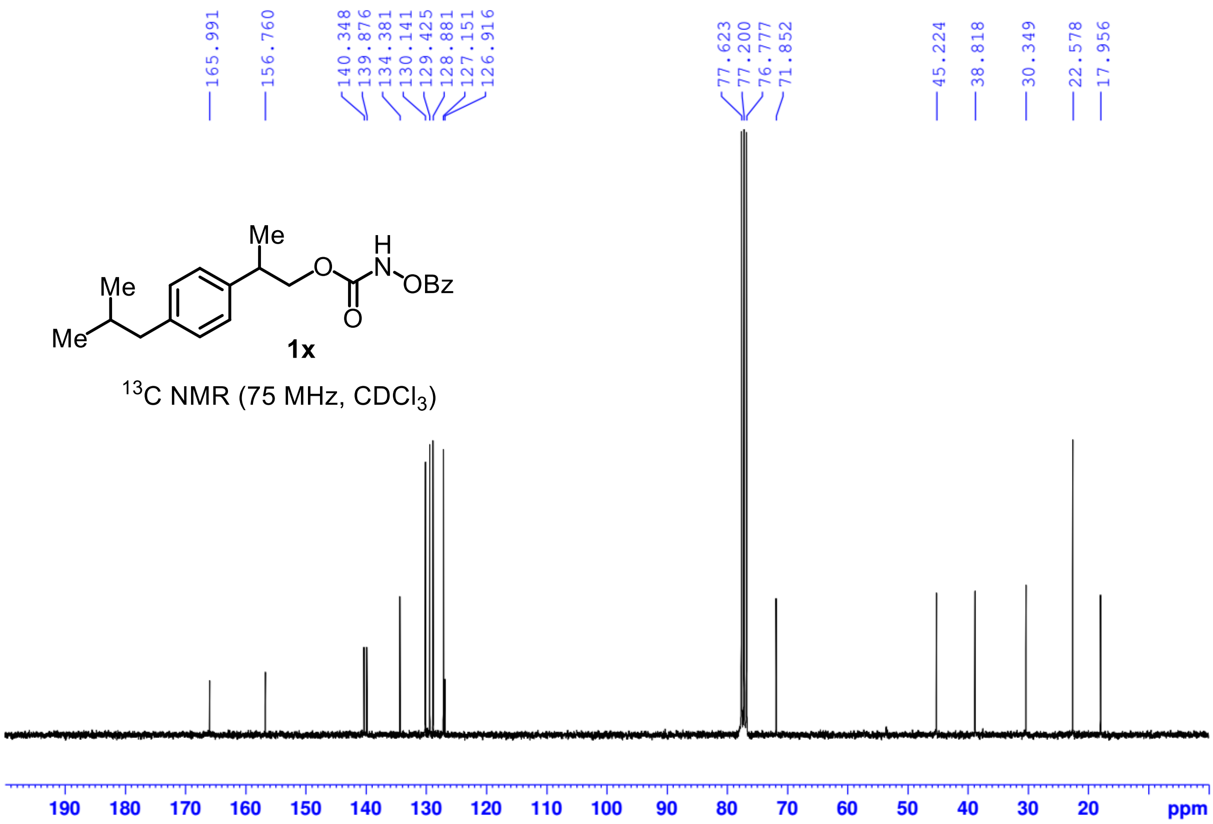

1x

^{13}C NMR (75 MHz, $CDCl_3$)

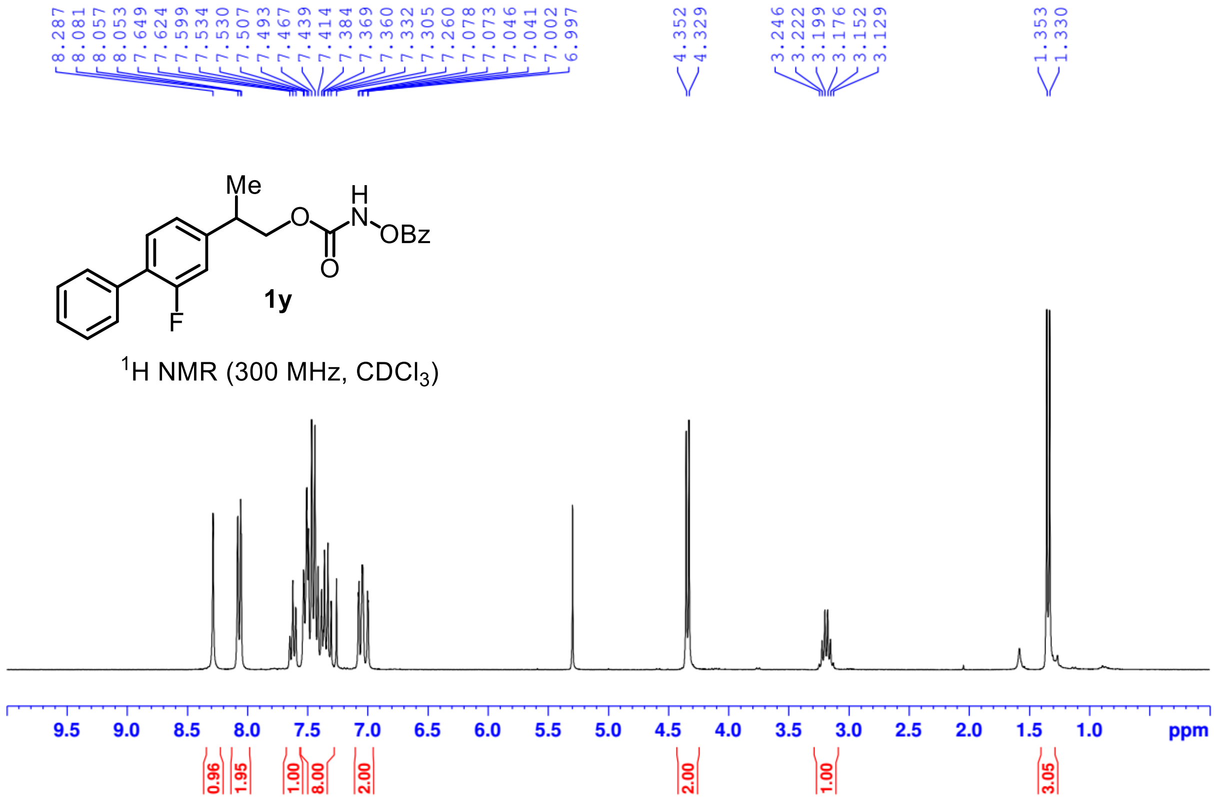

1y

^{1}H NMR (300 MHz, $CDCl_3$)

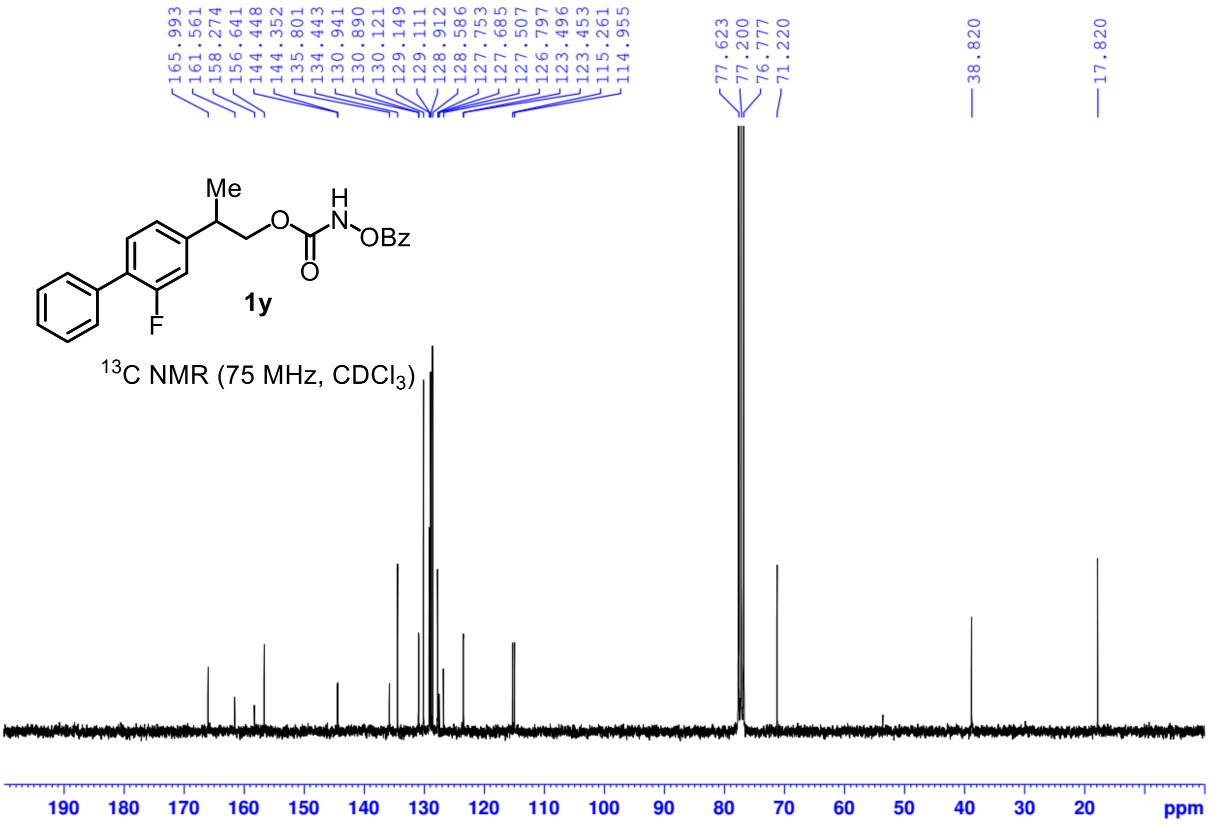
165.993
161.561
158.274
156.641
144.448
144.352
135.801
134.443
130.941
130.890
130.121
129.149
129.111
128.912
128.586
127.753
127.685
127.507
126.797
123.496
123.453
115.261
114.955
77.623
77.200
76.777
71.220
38.820
17.820
Me
H
N
O
OBz
F
1y
^{13}C NMR (75 MHz, $CDCl_3$)
190 180 170 160 150 140 130 120 110 100 90 80 70 60 50 40 30 20 ppm

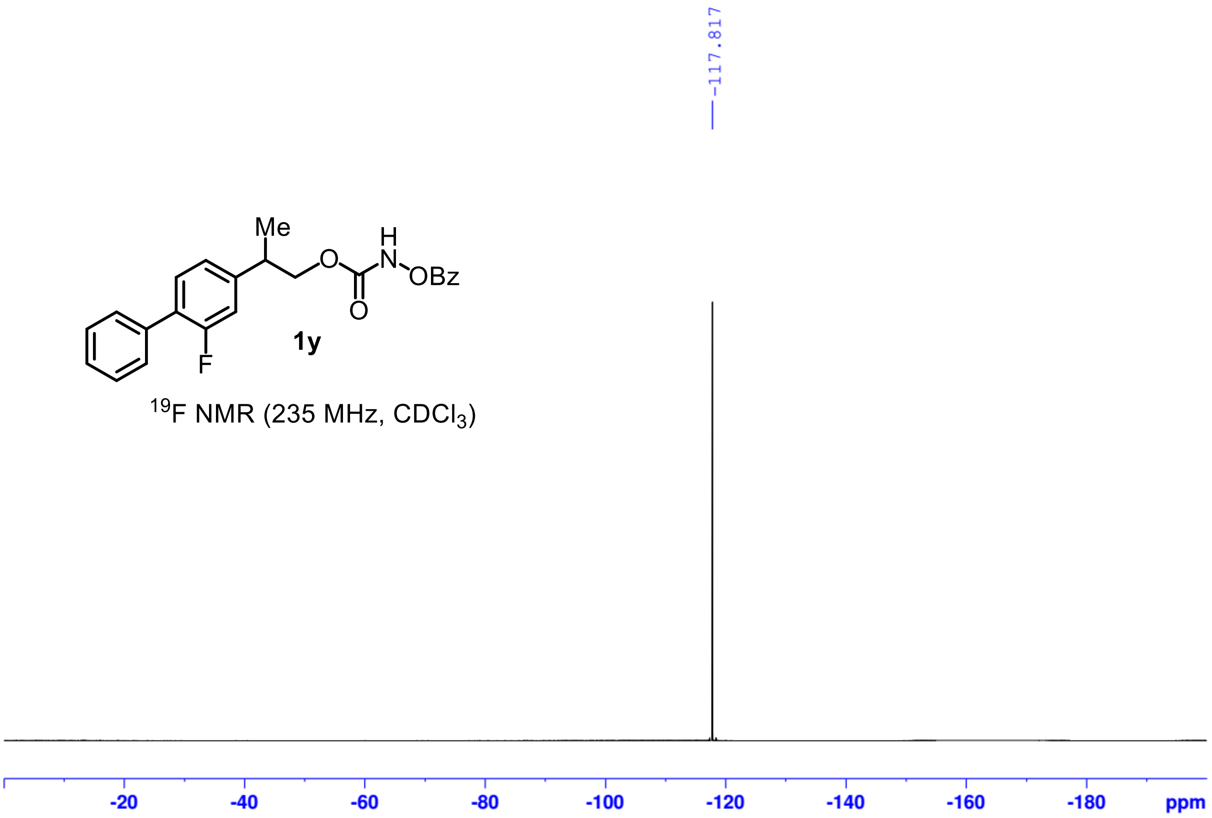
-117.817
Me
H
N
O
OBz
F
1y
^{19}F NMR (235 MHz, $CDCl_3$)
-20 -40 -60 -80 -100 -120 -140 -160 -180 ppm

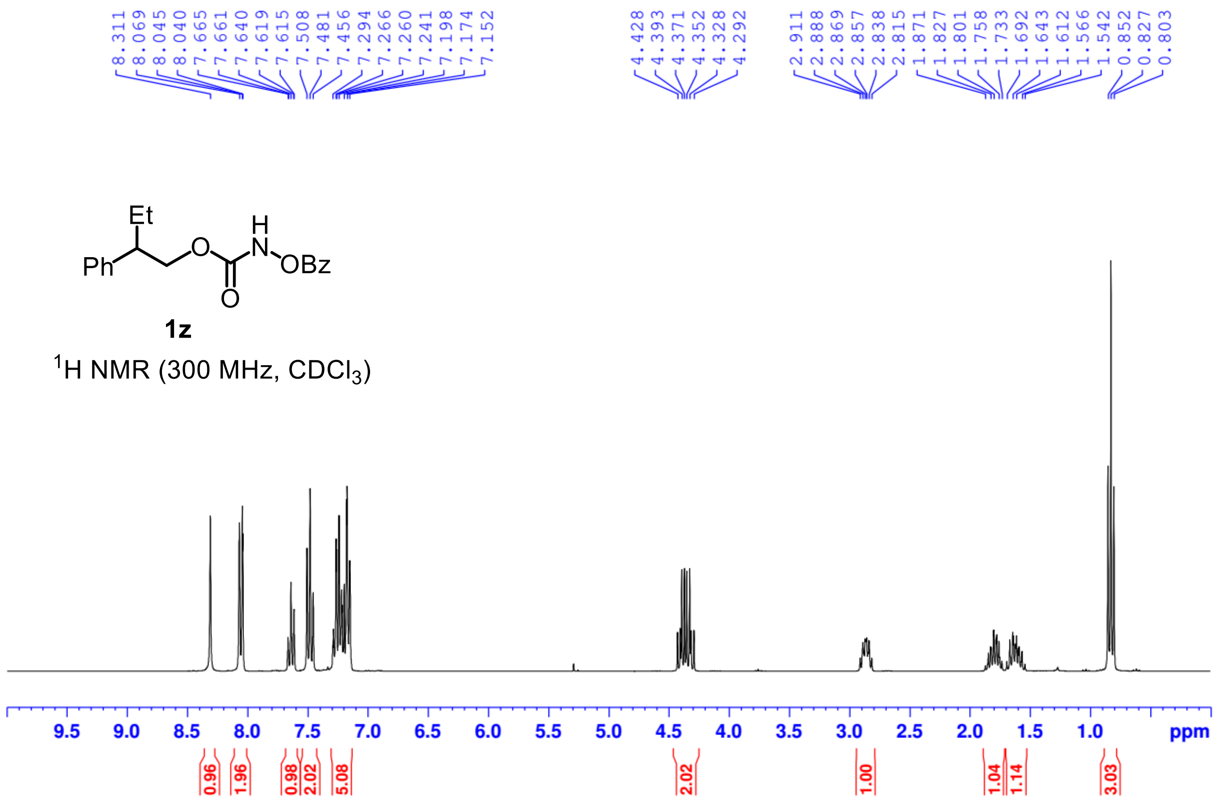
8.311 8.069 8.045 8.040 7.665 7.661 7.640 7.619 7.615 7.508 7.481 7.456 7.294 7.266 7.260 7.241 7.198 7.174 7.152
4.428 4.393 4.371 4.352 4.328 4.292
2.911 2.888 2.869 2.857 2.838 2.815 1.871 1.827 1.801 1.758 1.733 1.692 1.643 1.612 1.566 1.542 0.852 0.827 0.803
Et
H
Ph
O
N
OBz
O
1z
1H NMR (300 MHz, CDCl3)
9.5 9.0 8.5 8.0 7.5 7.0 6.5 6.0 5.5 5.0 4.5 4.0 3.5 3.0 2.5 2.0 1.5 1.0 ppm
0.96 1.96 0.98 2.02 5.08 2.02 1.00 1.04 1.14 3.03

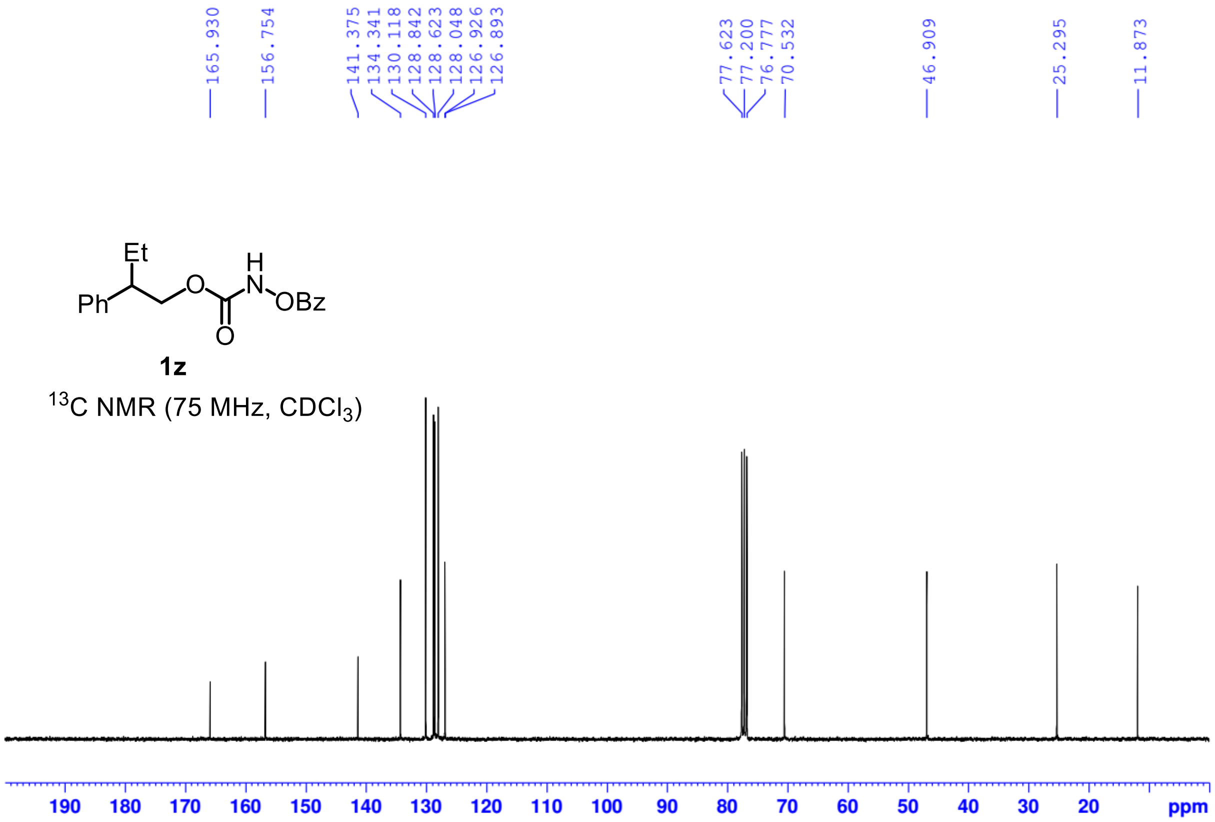
165.930
156.754
141.375 134.341 130.118 128.842 128.623 128.048 126.926 126.893
77.623 77.200 76.777 70.532
46.909
25.295
11.873
Et
H
Ph
O
N
OBz
O
1z
13C NMR (75 MHz, CDCl3)
190 180 170 160 150 140 130 120 110 100 90 80 70 60 50 40 30 20 ppm

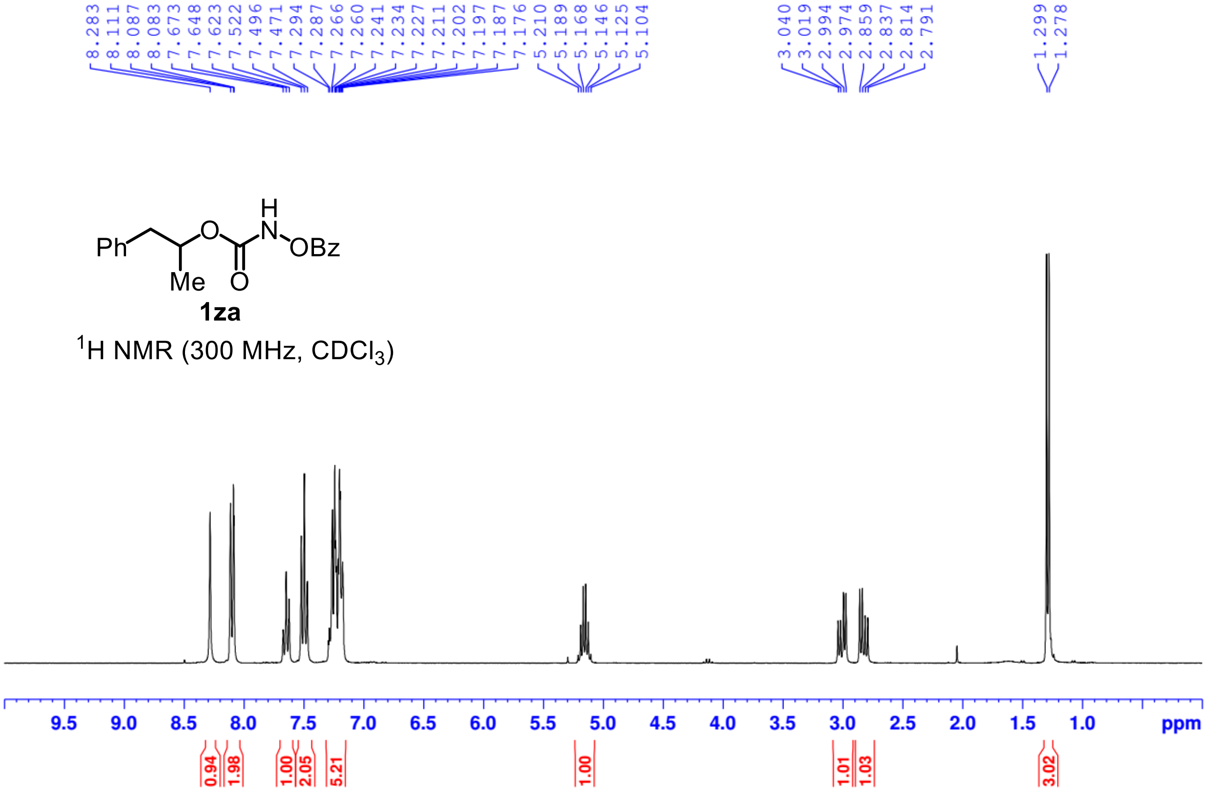
8.283 8.111 8.087 8.083 7.673 7.648 7.623 7.522 7.496 7.471 7.294 7.287 7.266 7.260 7.241 7.234 7.227 7.211 7.202 7.197 7.187 7.176 5.210 5.189 5.168 5.146 5.125 5.104
3.040 3.019 2.994 2.974 2.859 2.837 2.814 2.791
1.299 1.278
H
Ph
O
N
OBz
Me
O
1za
^{1}H NMR (300 MHz, CDCl$_3$)
9.5 9.0 8.5 8.0 7.5 7.0 6.5 6.0 5.5 5.0 4.5 4.0 3.5 3.0 2.5 2.0 1.5 1.0 ppm
0.94 1.98 1.00 2.05 5.21 1.00 1.01 1.03 3.02

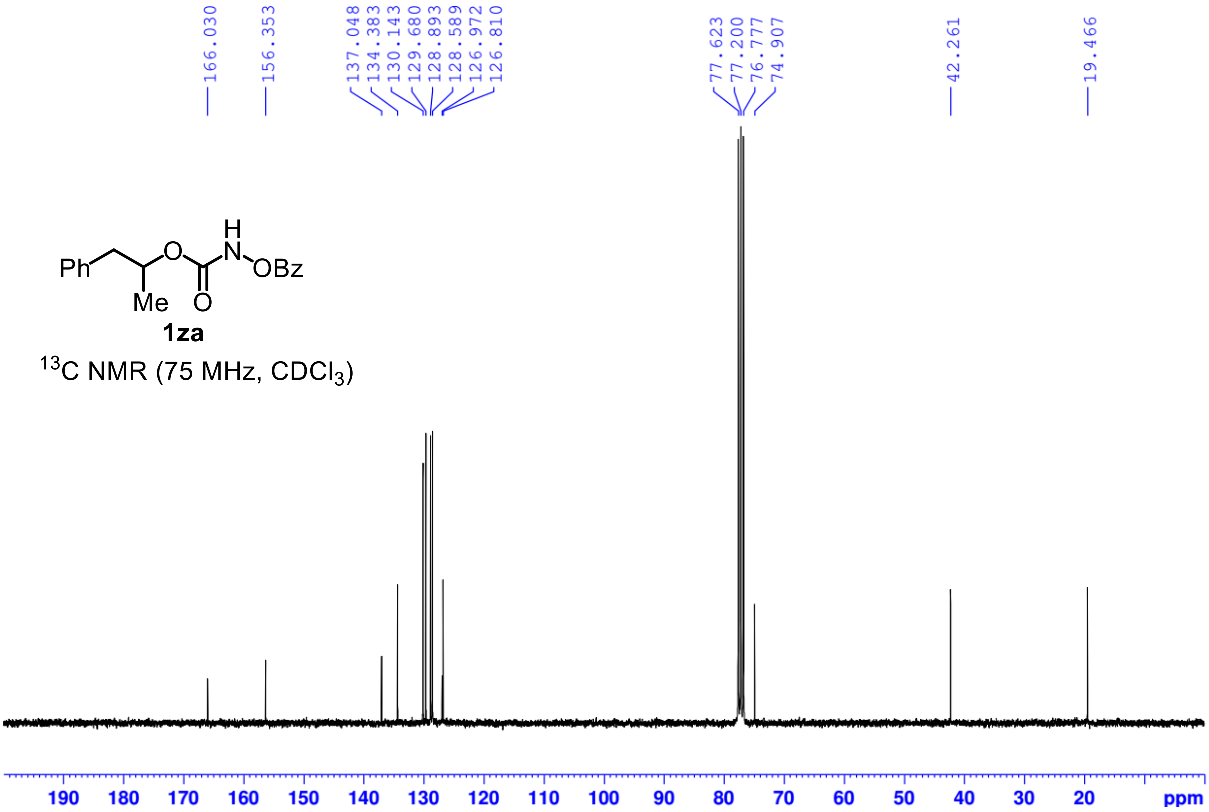
166.030
156.353
137.048 134.383 130.143 129.680 128.893 128.589 126.972 126.810
77.623 77.200 76.777 74.907
42.261
19.466
H
Ph
O
N
OBz
Me
O
1za
^{13}C NMR (75 MHz, CDCl$_3$)
190 180 170 160 150 140 130 120 110 100 90 80 70 60 50 40 30 20 ppm

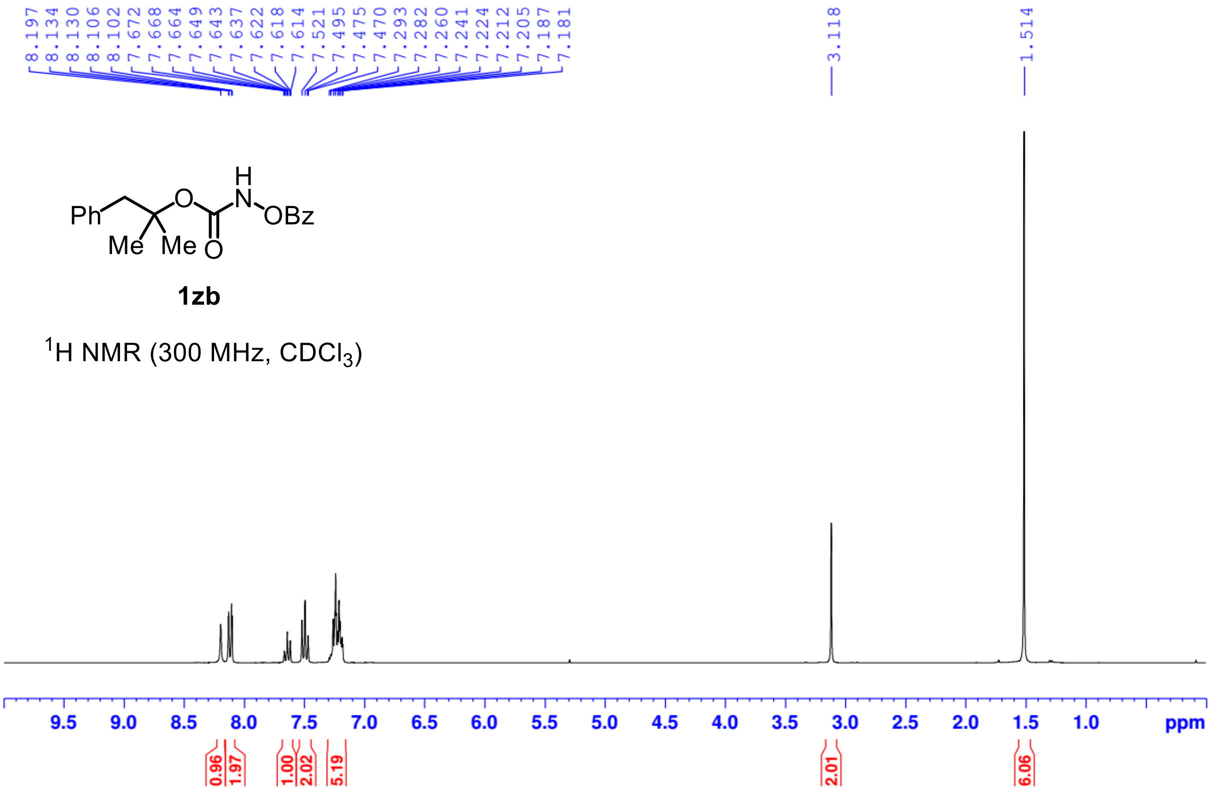

1zb

^{1}H NMR (300 MHz, $CDCl_3$)

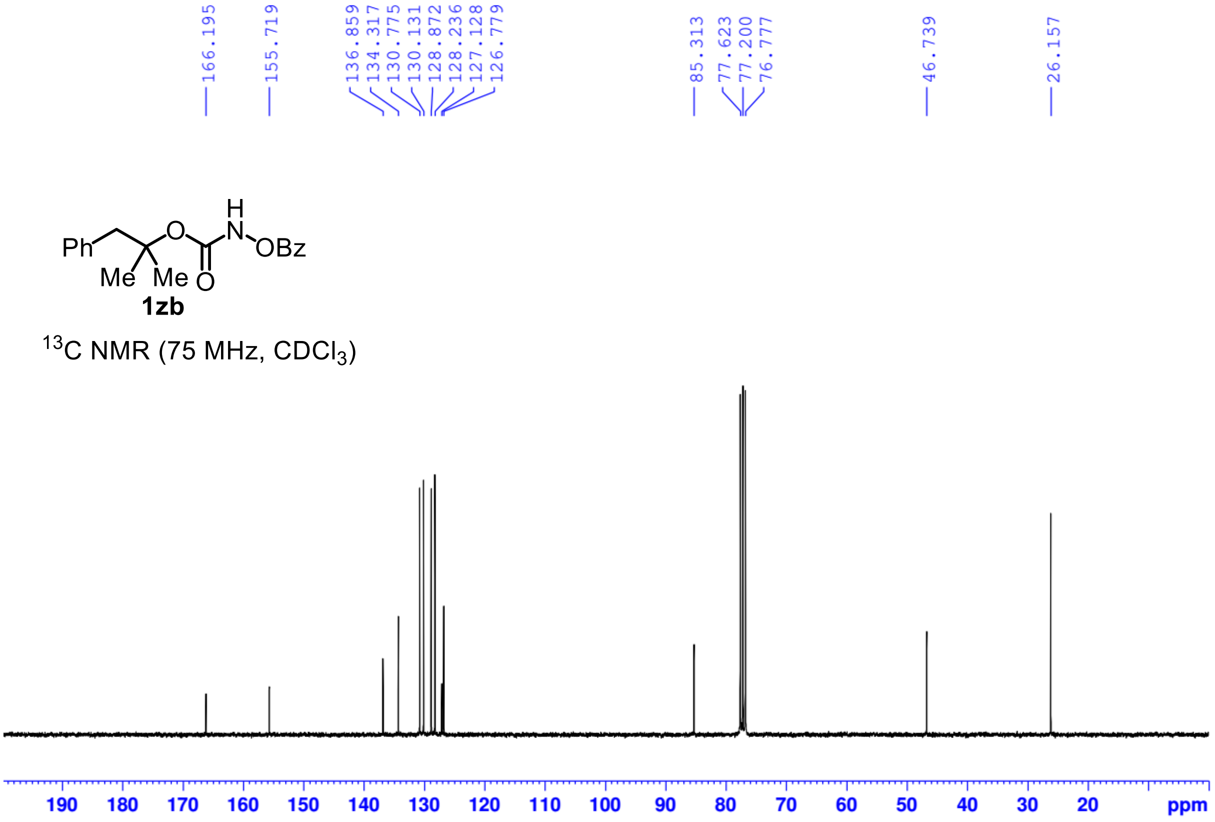

1zb

^{13}C NMR (75 MHz, $CDCl_3$)

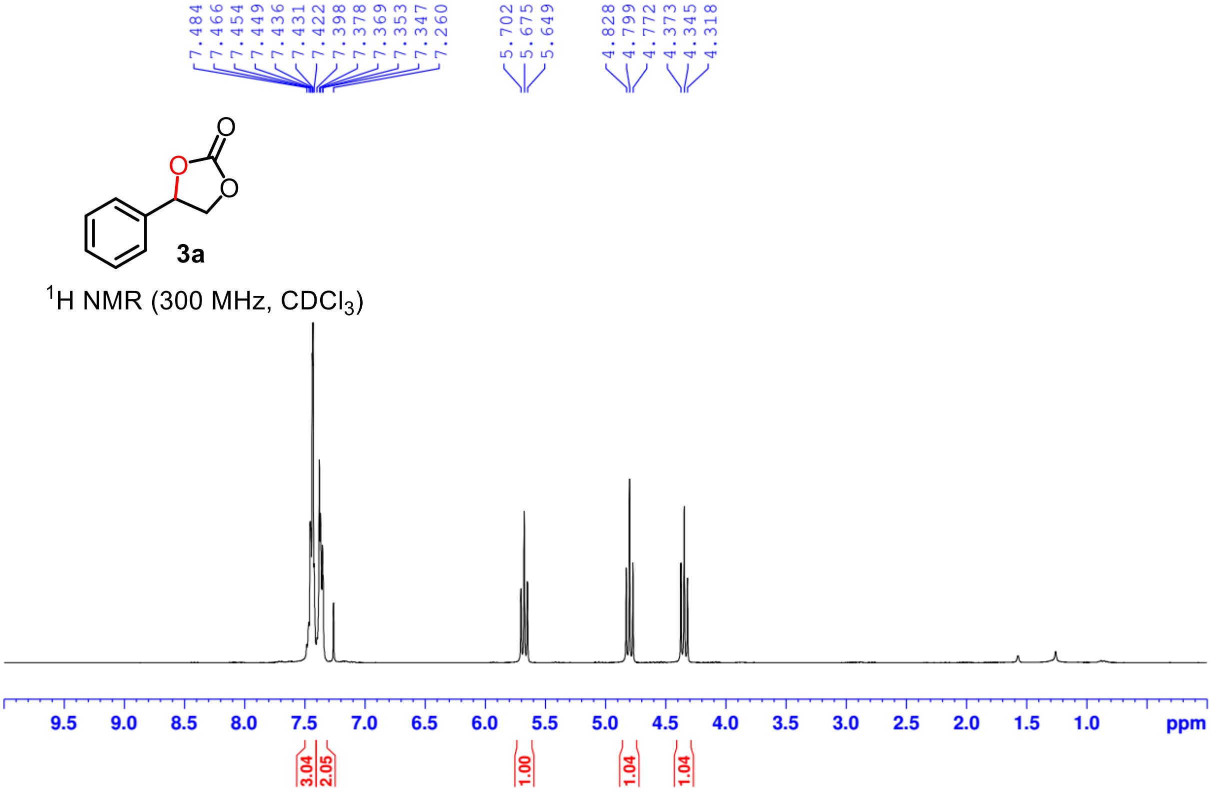

3a

^{1}H NMR (300 MHz, $CDCl_3$)

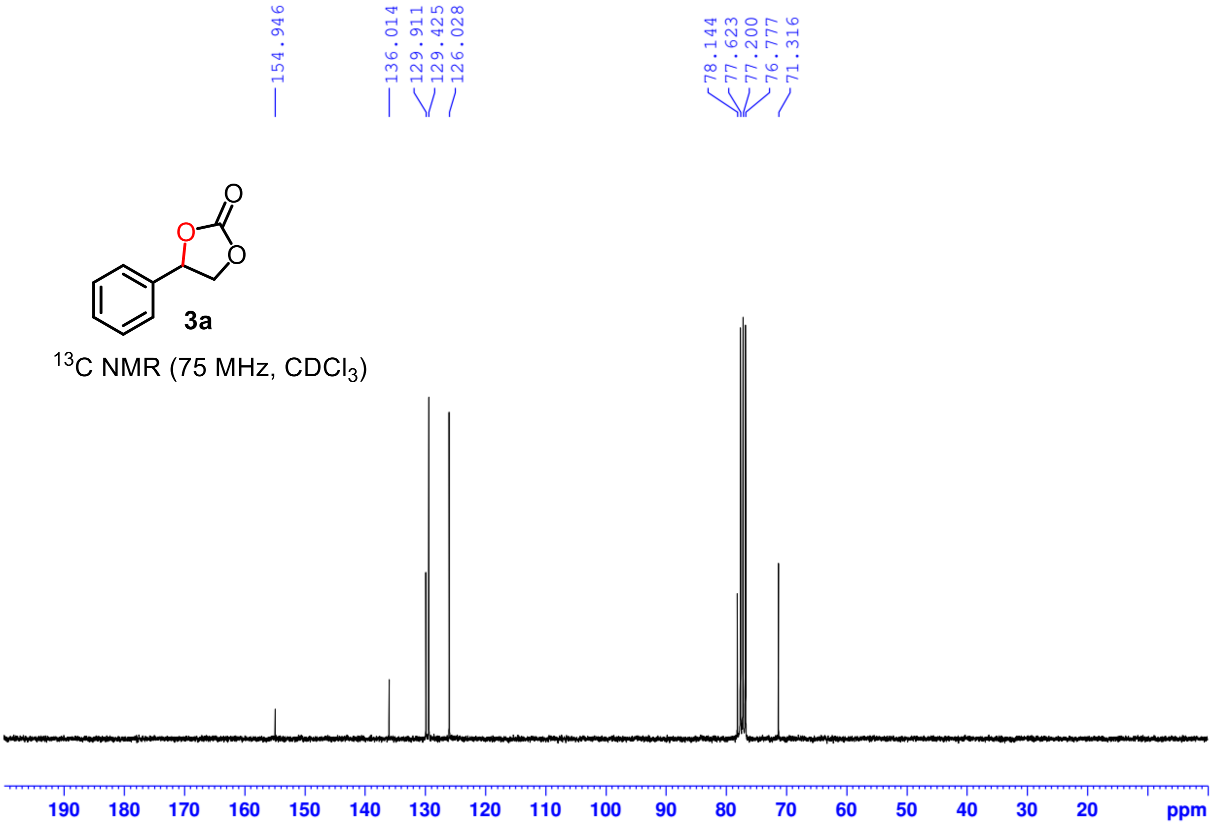

3a

^{13}C NMR (75 MHz, $CDCl_3$)

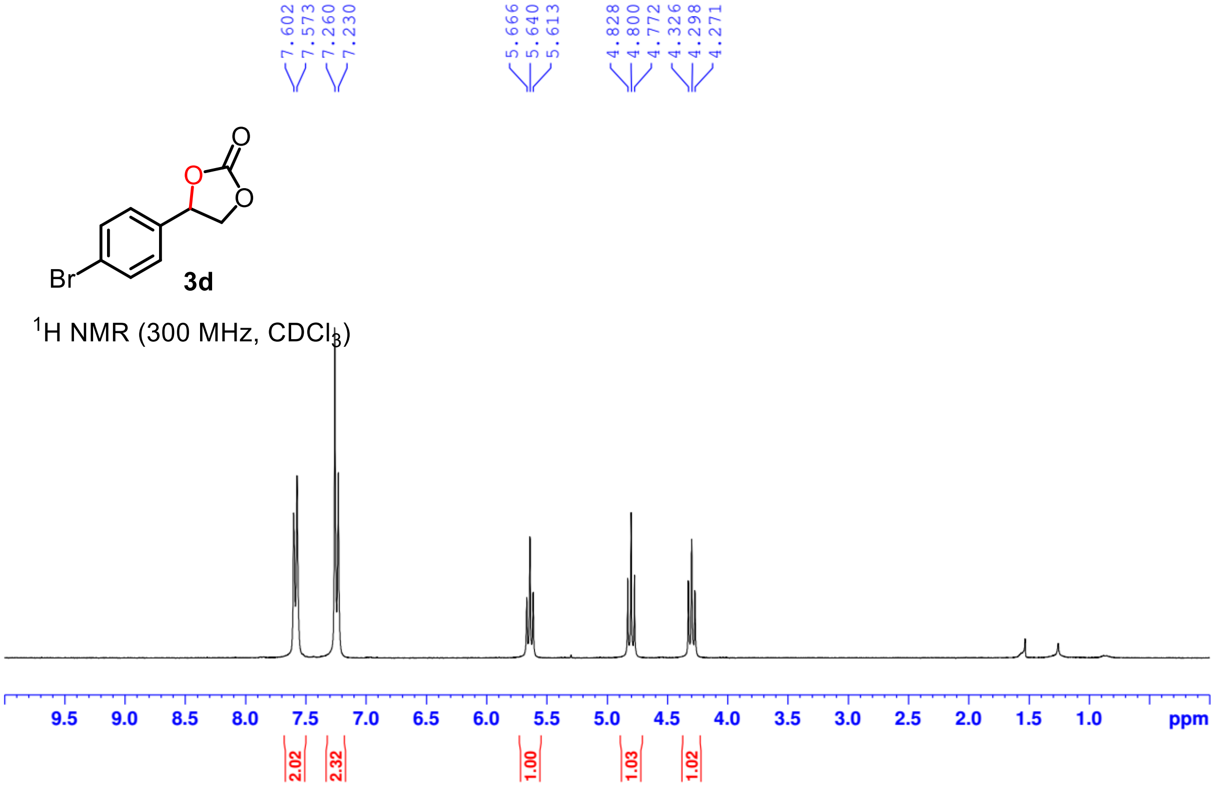
7.602
7.573
7.260
7.230
5.666
5.640
5.613
4.828
4.800
4.772
4.326
4.298
4.271
Br
3d
O
^{1}H NMR (300 MHz, CDCl$_3$)
9.5
9.0
8.5
8.0
7.5
7.0
6.5
6.0
5.5
5.0
4.5
4.0
3.5
3.0
2.5
2.0
1.5
1.0
ppm
2.02
2.32
1.00
1.03
1.02

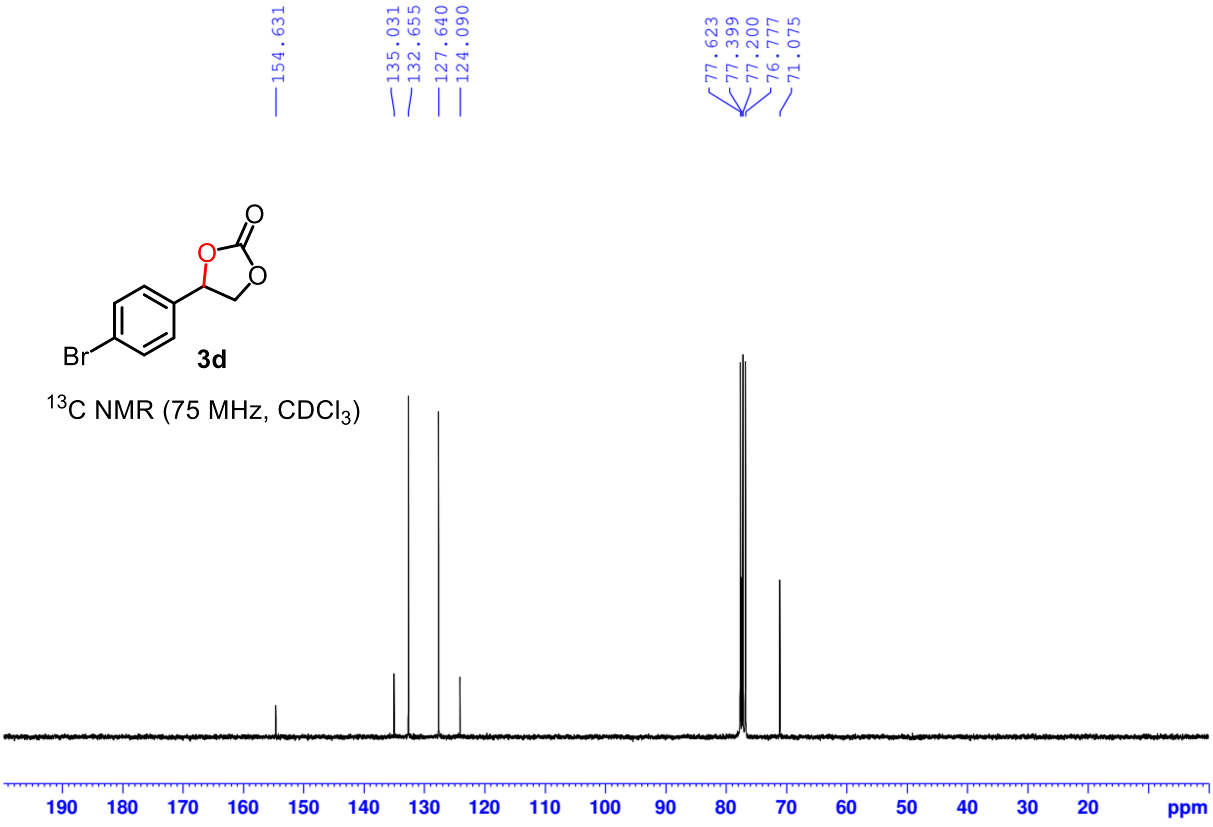
154.631
135.031
132.655
127.640
124.090
77.623
77.399
77.200
76.777
71.075
Br
3d
O
^{13}C NMR (75 MHz, CDCl$_3$)
190
180
170
160
150
140
130
120
110
100
90
80
70
60
50
40
30
20
ppm

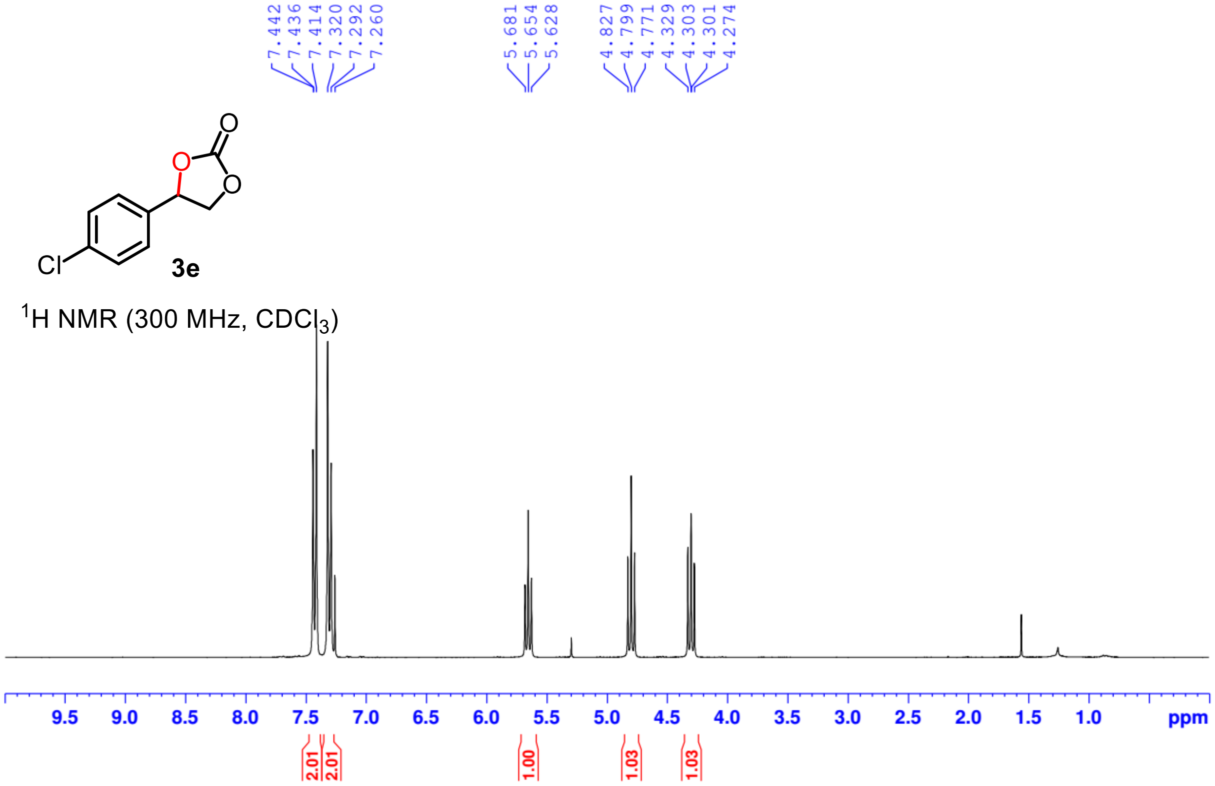
7.442
7.436
7.414
7.320
7.292
7.260
5.681
5.654
5.628
4.827
4.799
4.771
4.329
4.303
4.301
4.274
O
O
O
Cl
3e
1H NMR (300 MHz, CDCl3)
9.5 9.0 8.5 8.0 7.5 7.0 6.5 6.0 5.5 5.0 4.5 4.0 3.5 3.0 2.5 2.0 1.5 1.0 ppm
2.01
2.01
1.00
1.03
1.03

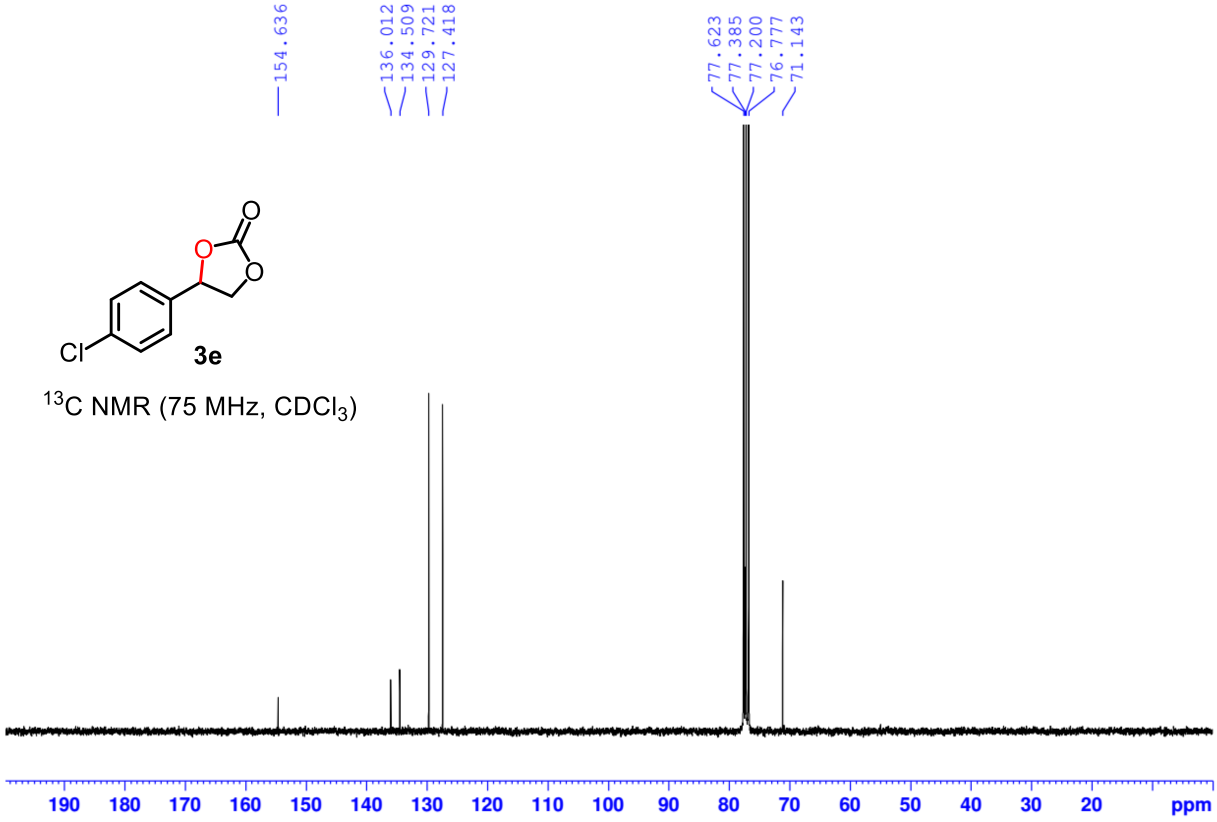
154.636
136.012
134.509
129.721
127.418
77.623
77.385
77.200
76.777
71.143
O
O
O
Cl
3e
13C NMR (75 MHz, CDCl3)
190 180 170 160 150 140 130 120 110 100 90 80 70 60 50 40 30 20 ppm

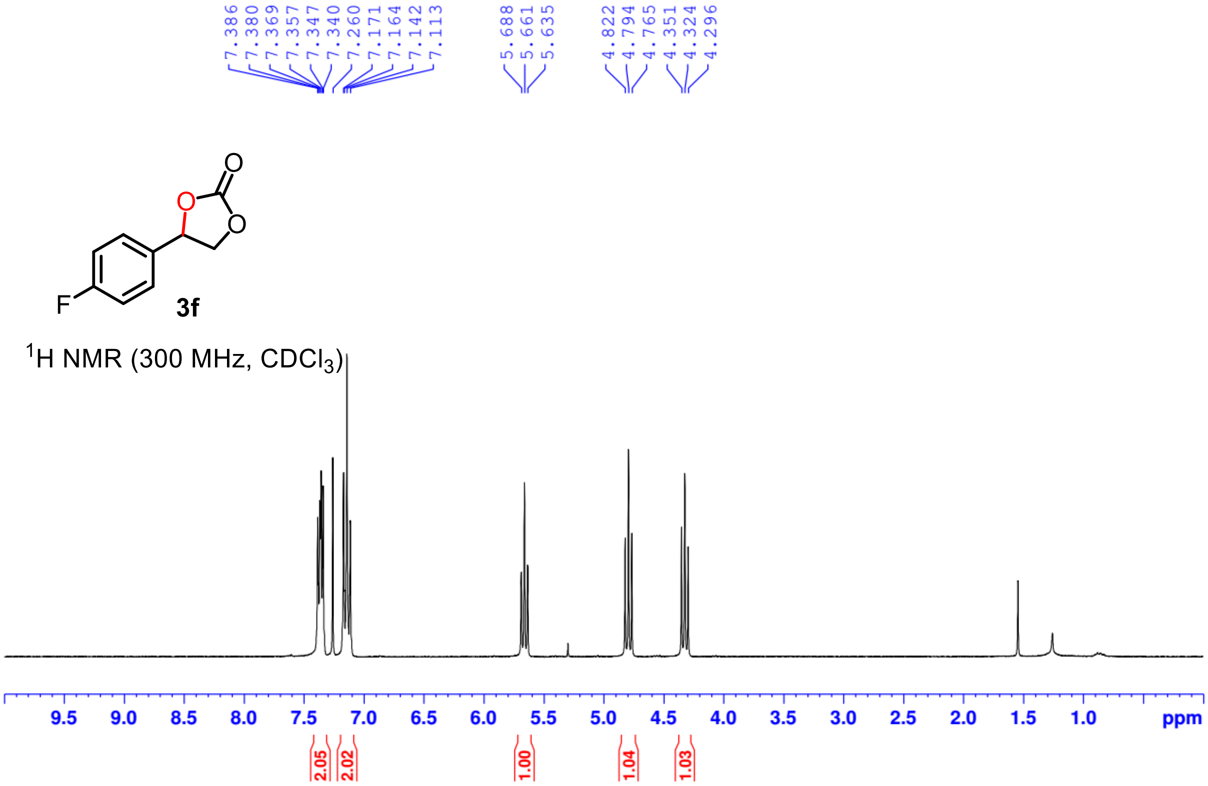

7.386 7.380 7.369 7.357 7.347 7.340 7.260 7.171 7.164 7.142 7.113
5.688 5.661 5.635
4.822 4.794 4.765 4.351 4.324 4.296
3f
1H NMR (300 MHz, CDCl3)
2.05 2.02 1.00 1.04 1.03
ppm

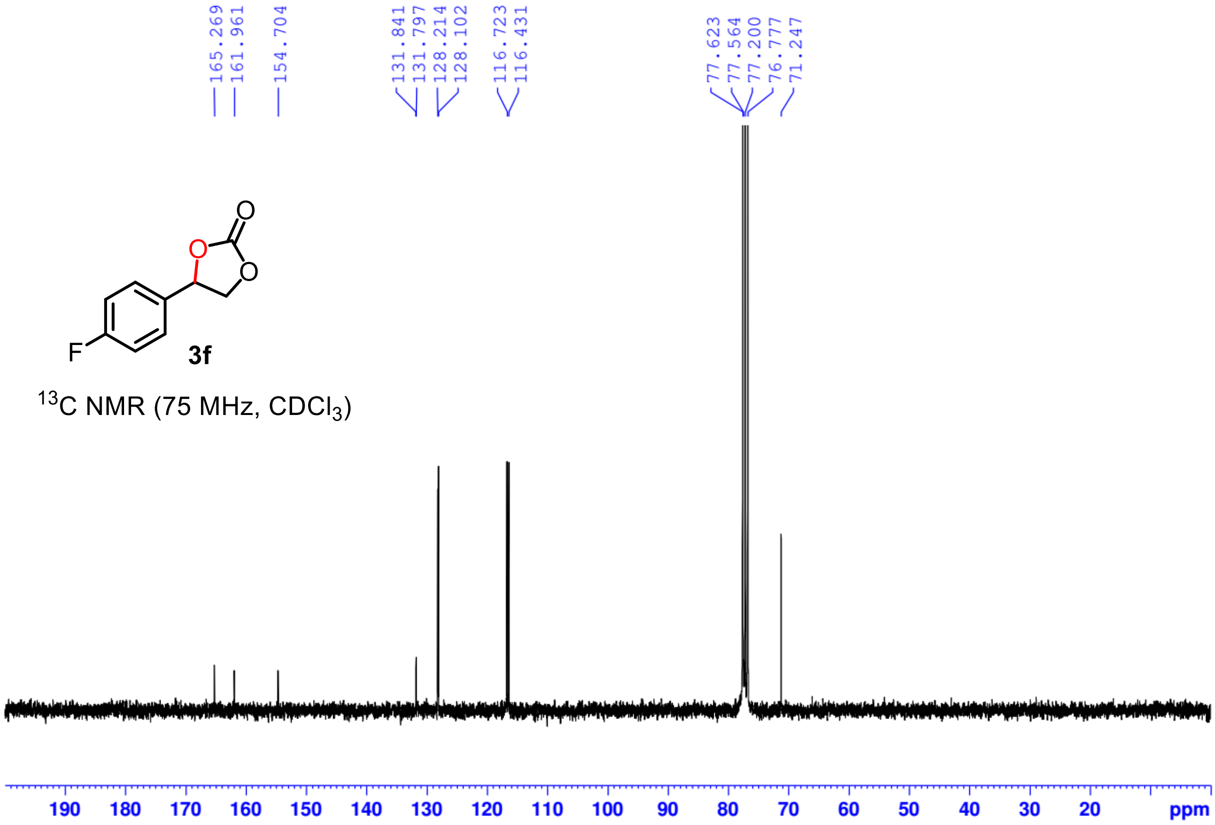

165.269 161.961 154.704
131.841 131.797 128.214 128.102 116.723 116.431
77.623 77.564 77.200 76.777 71.247
3f
13C NMR (75 MHz, CDCl3)
ppm

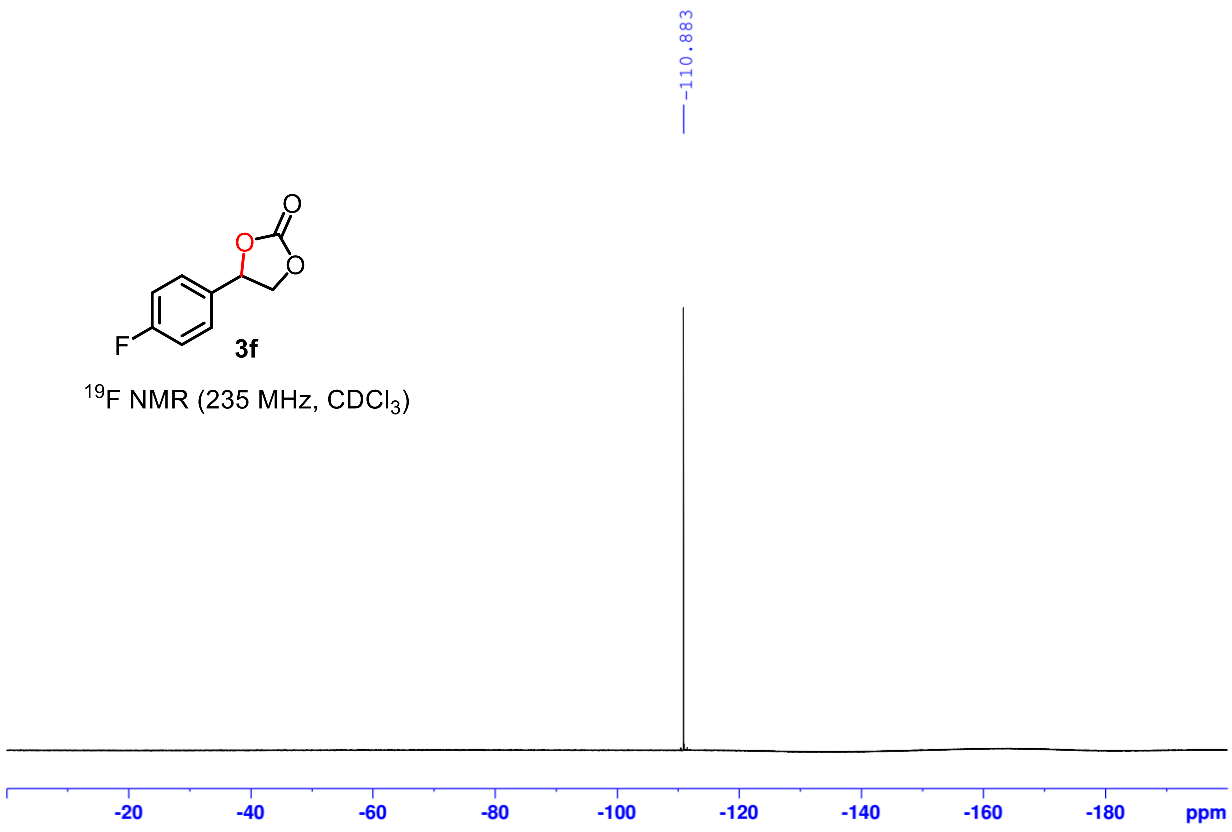

^{19}F NMR (235 MHz, $CDCl_3$)

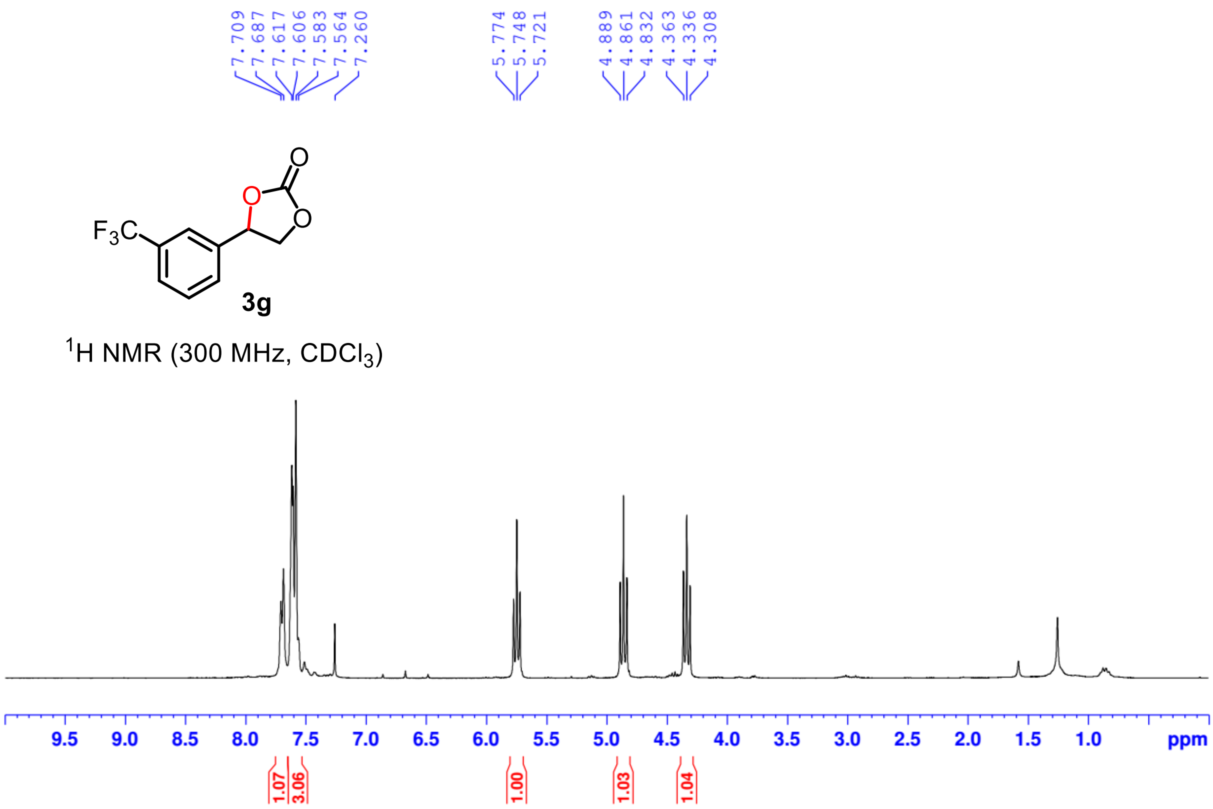

^{1}H NMR (300 MHz, $CDCl_3$)

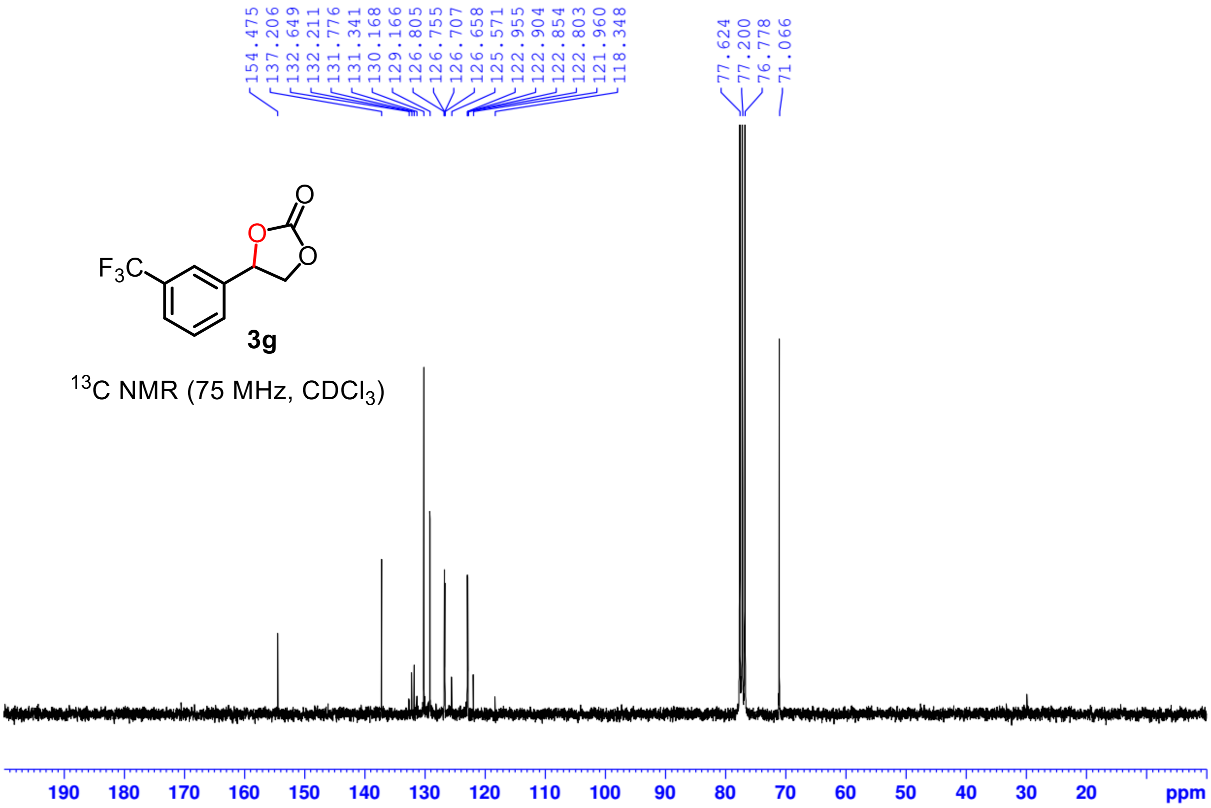
154.475
137.206
132.649
132.211
131.776
131.341
130.168
129.166
126.805
126.755
126.707
126.658
125.571
122.955
122.904
122.854
122.803
121.960
118.348
77.624
77.200
76.778
71.066
F3C
3g
13C NMR (75 MHz, CDCl3)
190
180
170
160
150
140
130
120
110
100
90
80
70
60
50
40
30
20
ppm

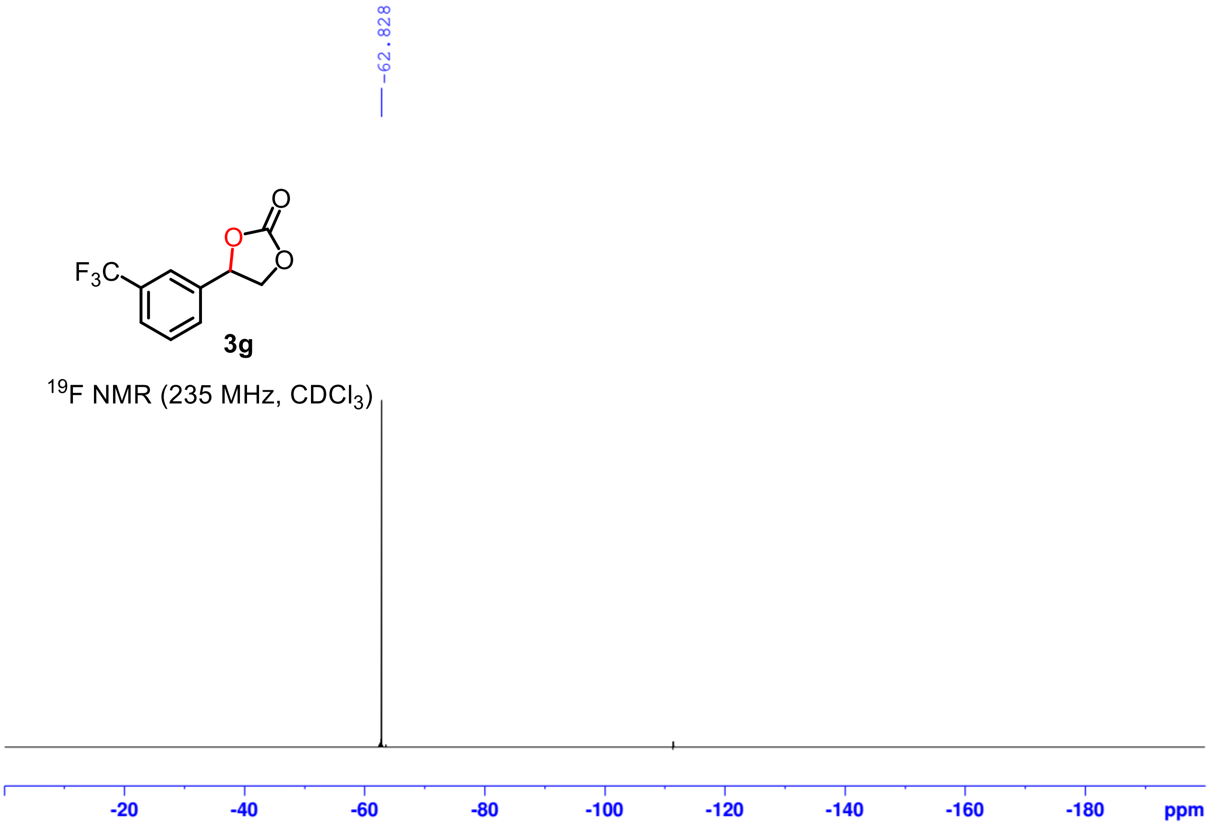
-62.828
F3C
3g
19F NMR (235 MHz, CDCl3)
-20
-40
-60
-80
-100
-120
-140
-160
-180
ppm

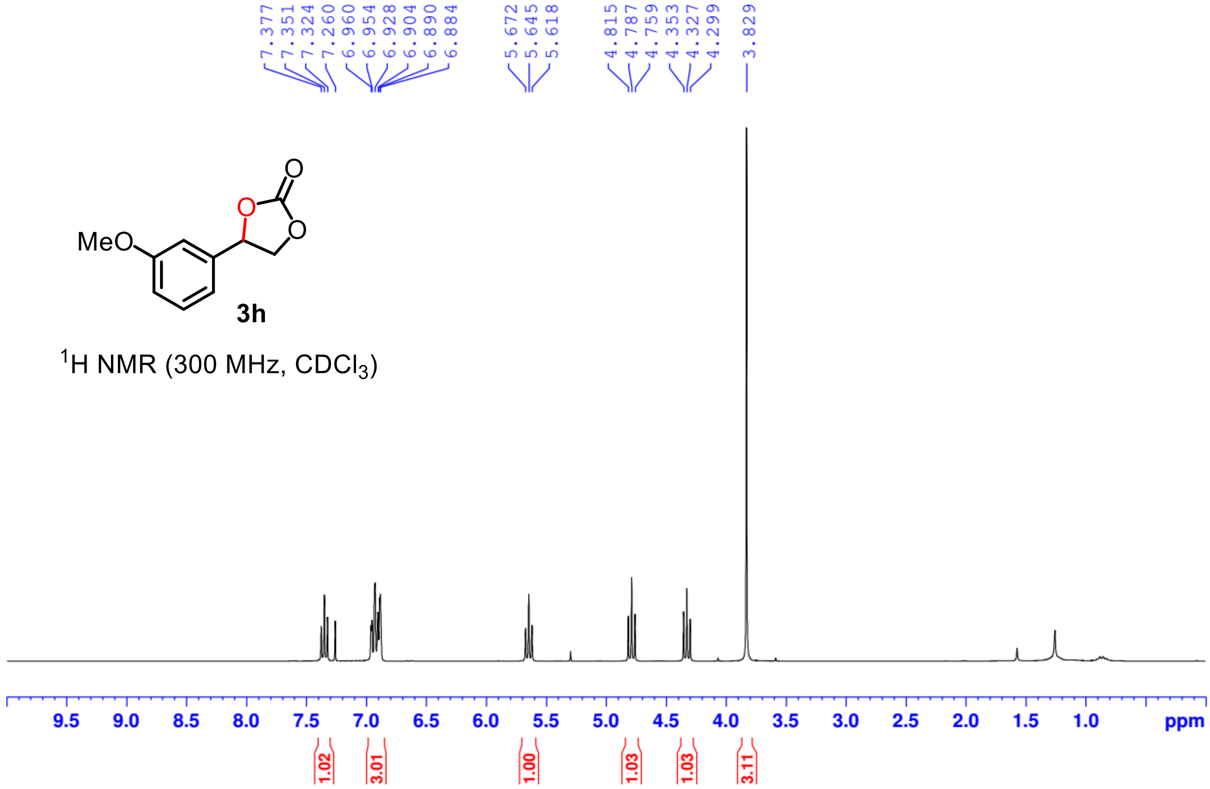
7.377
7.351
7.324
7.260
6.960
6.954
6.928
6.904
6.890
6.884
5.672
5.645
5.618
4.815
4.787
4.759
4.353
4.327
4.299
3.829
MeO
O
O
O
3h
1H NMR (300 MHz, CDCl3)
9.5 9.0 8.5 8.0 7.5 7.0 6.5 6.0 5.5 5.0 4.5 4.0 3.5 3.0 2.5 2.0 1.5 1.0 ppm
1.02
3.01
1.00
1.03
1.03
3.11

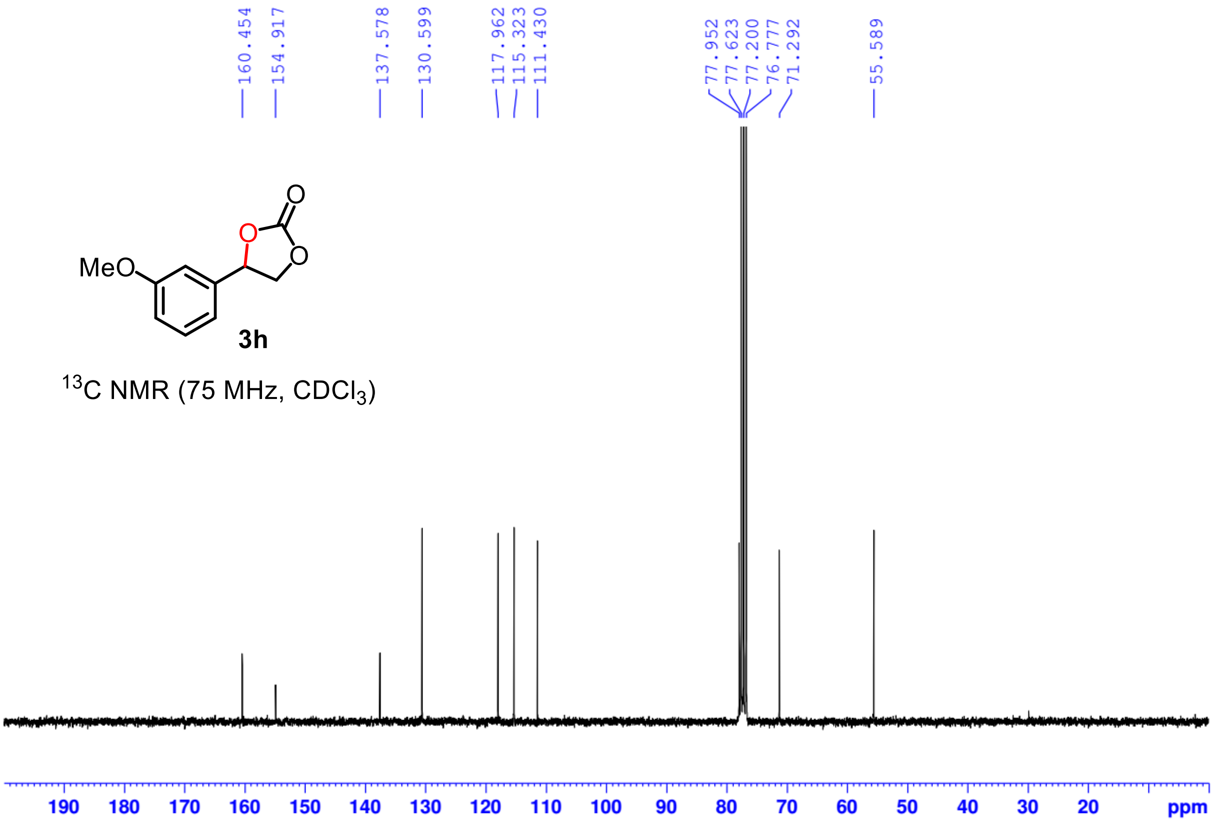
160.454
154.917
137.578
130.599
117.962
115.323
111.430
77.952
77.623
77.200
76.777
71.292
55.589
MeO
O
O
O
3h
13C NMR (75 MHz, CDCl3)
190 180 170 160 150 140 130 120 110 100 90 80 70 60 50 40 30 20 ppm

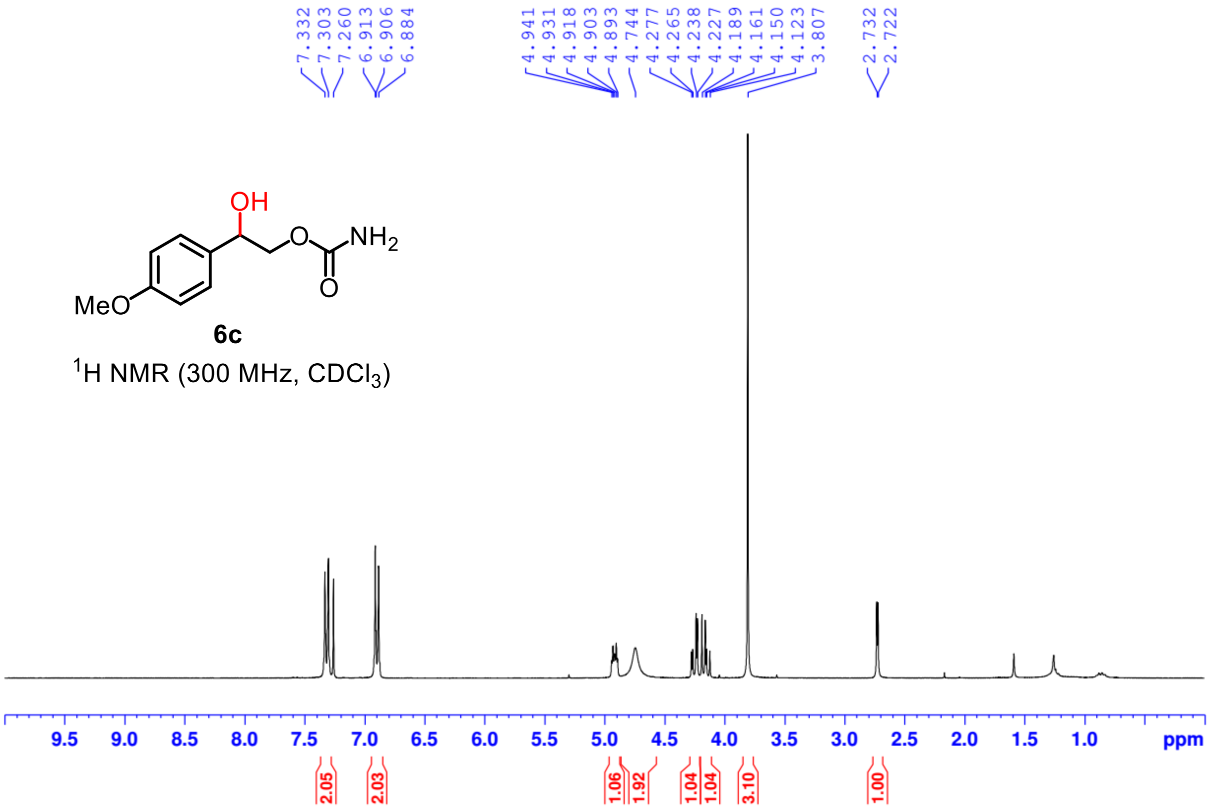
7.332
7.303
7.260
6.913
6.906
6.884
4.941
4.931
4.918
4.903
4.893
4.744
4.277
4.265
4.238
4.227
4.189
4.161
4.150
4.123
3.807
2.732
2.722
OH
NH2
O
O
MeO
6c
1H NMR (300 MHz, CDCl3)
9.5 9.0 8.5 8.0 7.5 7.0 6.5 6.0 5.5 5.0 4.5 4.0 3.5 3.0 2.5 2.0 1.5 1.0 ppm
2.05
2.03
1.06
1.92
1.04
1.04
3.10
1.00

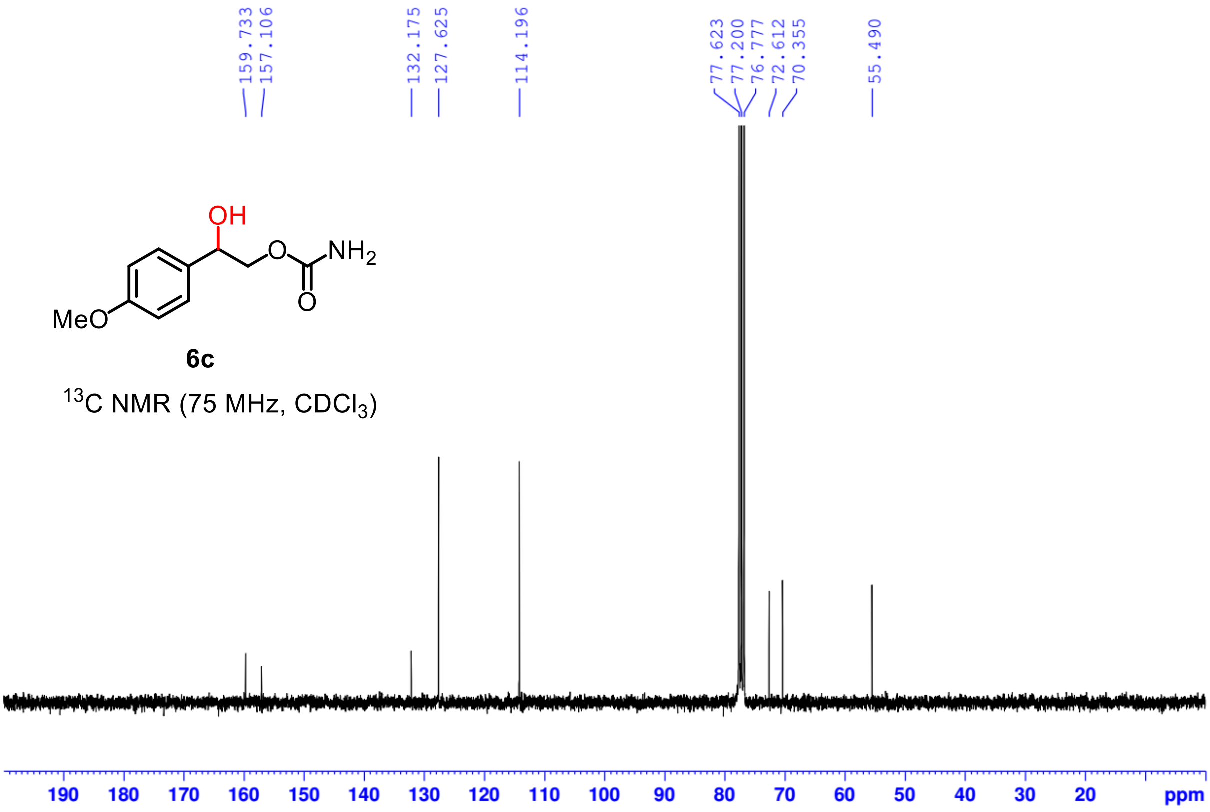
159.733
157.106
132.175
127.625
114.196
77.623
77.200
76.777
72.612
70.355
55.490
OH
NH2
O
O
MeO
6c
13C NMR (75 MHz, CDCl3)
190 180 170 160 150 140 130 120 110 100 90 80 70 60 50 40 30 20 ppm

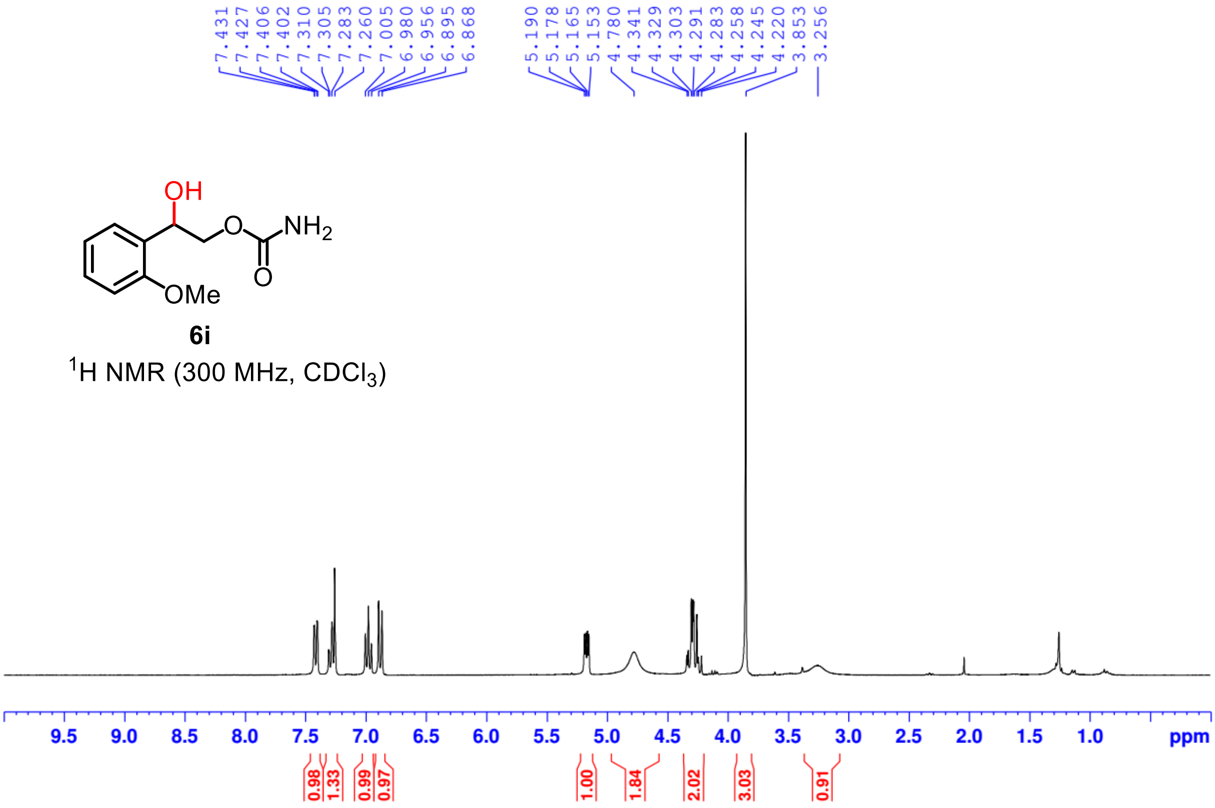

6i

^{1}H NMR (300 MHz, $CDCl_3$)

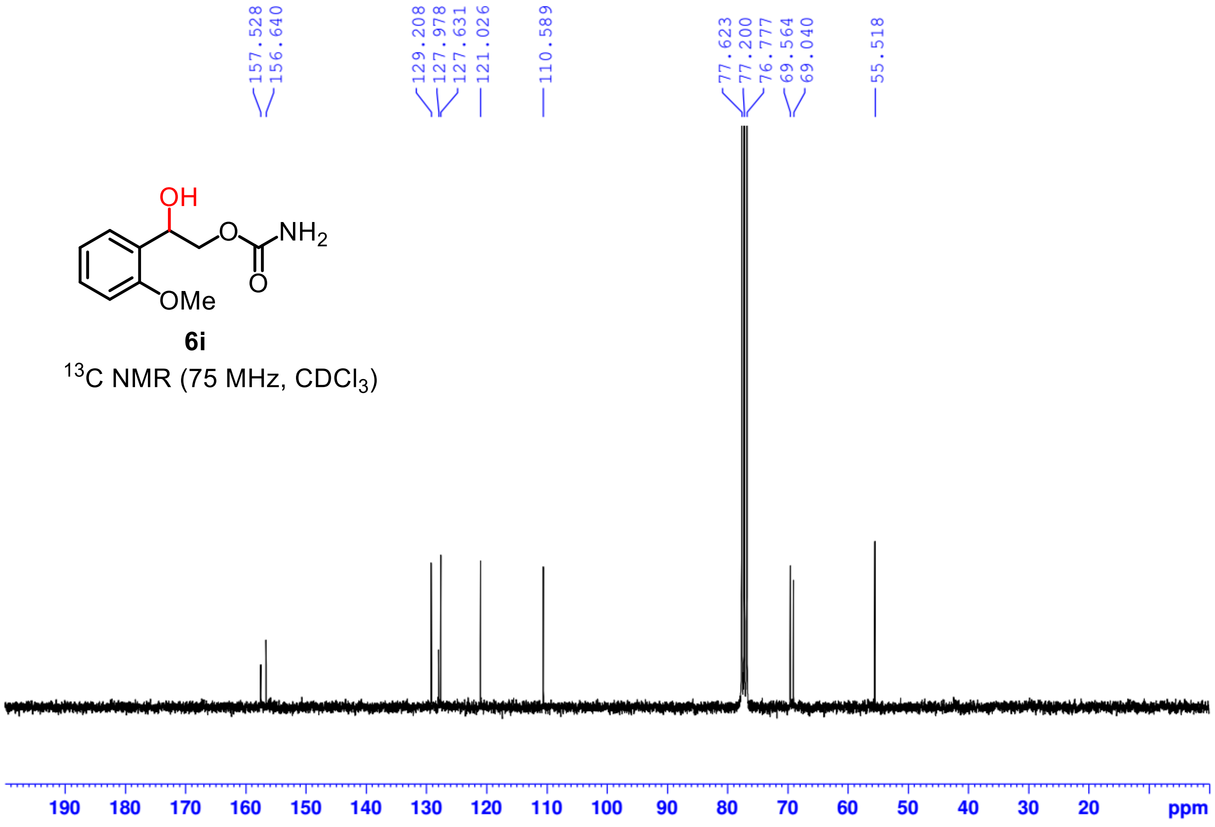

6i

^{13}C NMR (75 MHz, $CDCl_3$)

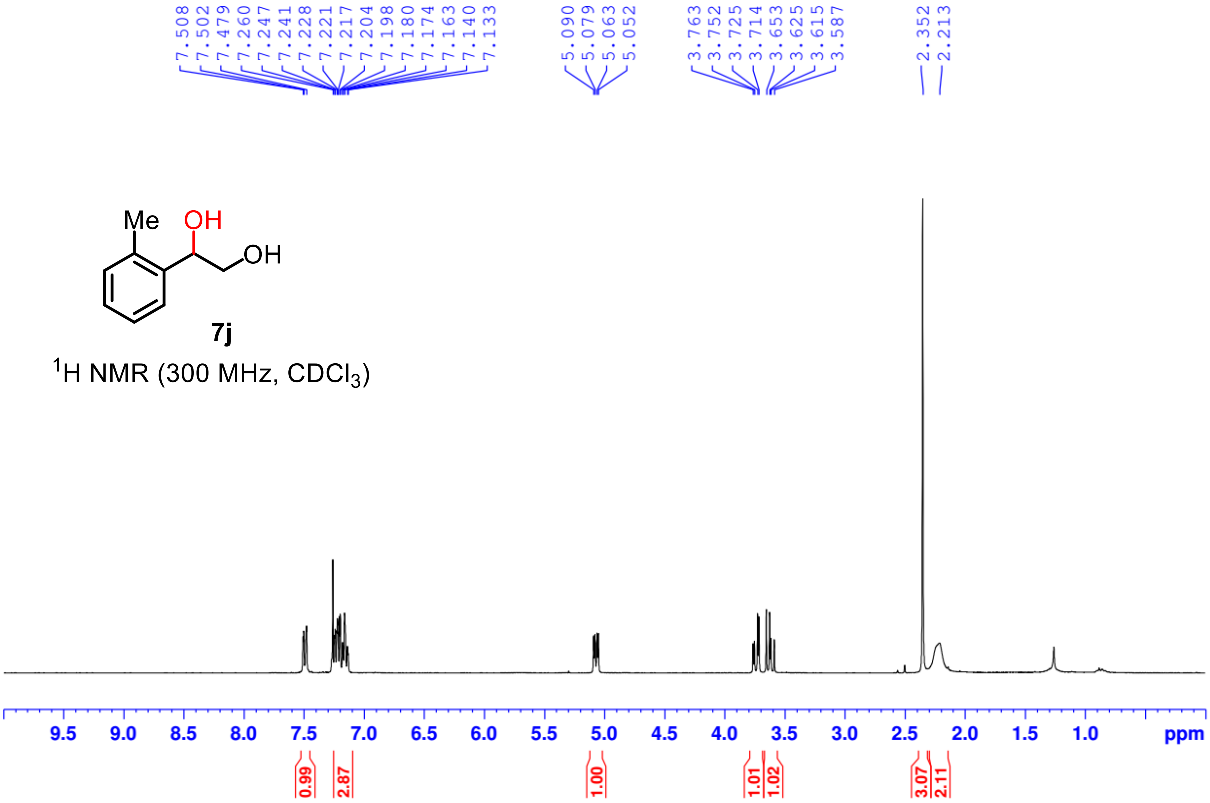

7j

^{1}H NMR (300 MHz, $CDCl_3$)

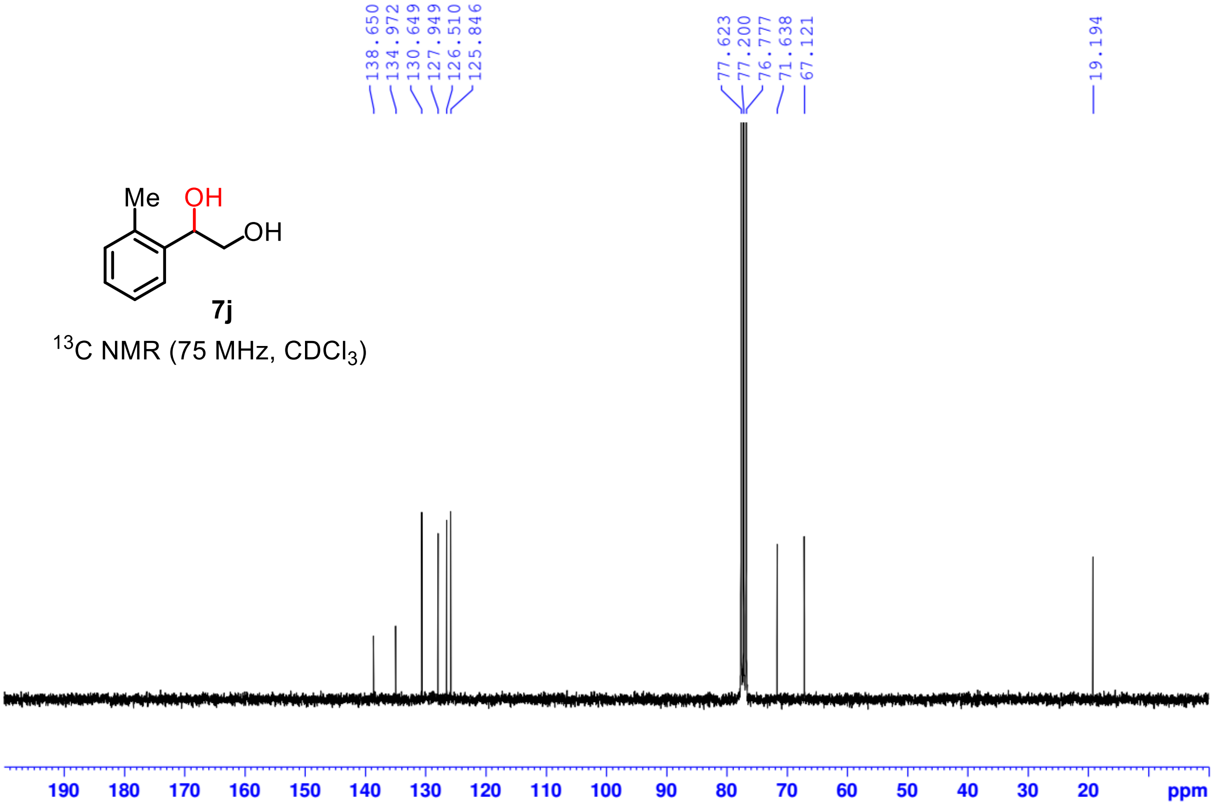

7j

^{13}C NMR (75 MHz, $CDCl_3$)

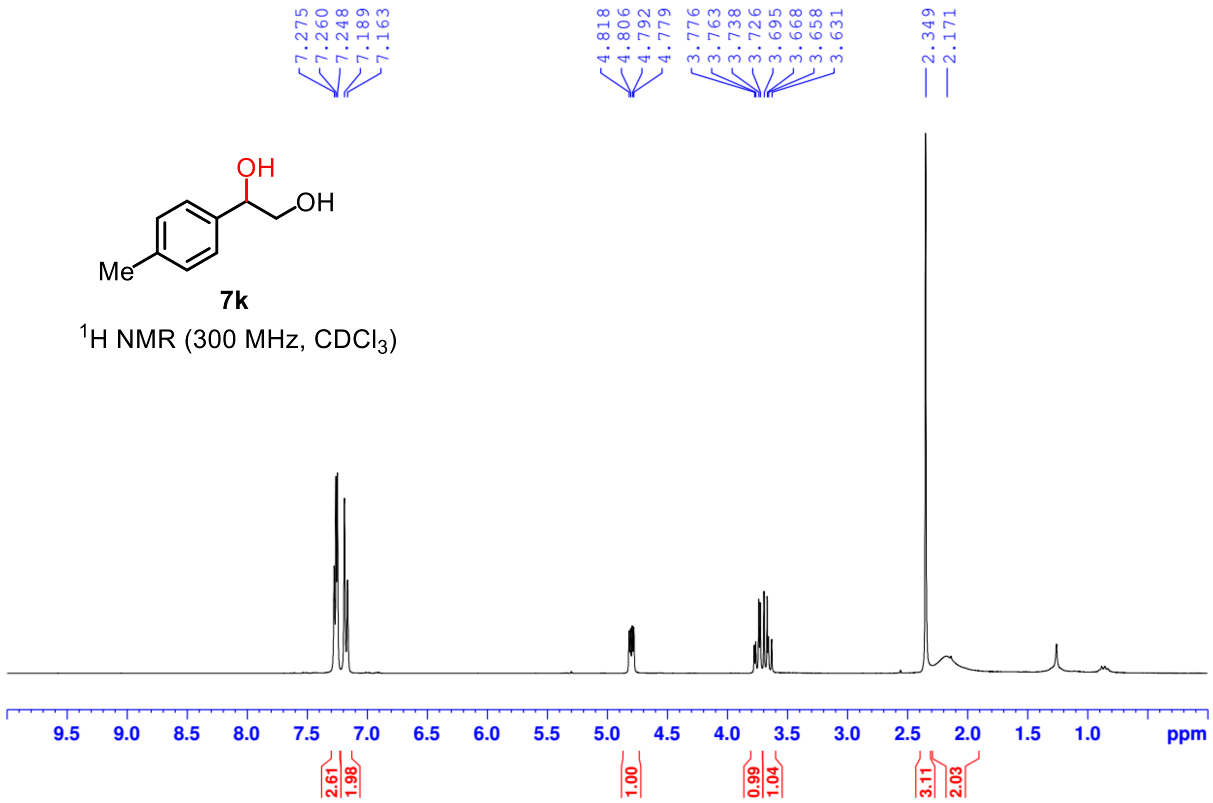
7.275
7.260
7.248
7.189
7.163
4.818
4.806
4.792
4.779
3.776
3.763
3.738
3.726
3.695
3.668
3.658
3.631
2.349
2.171
OH
OH
Me
7k
^{1}H NMR (300 MHz, $CDCl_3$)
9.5
9.0
8.5
8.0
7.5
7.0
6.5
6.0
5.5
5.0
4.5
4.0
3.5
3.0
2.5
2.0
1.5
1.0
ppm
2.61
1.98
1.00
0.99
1.04
3.11
2.03

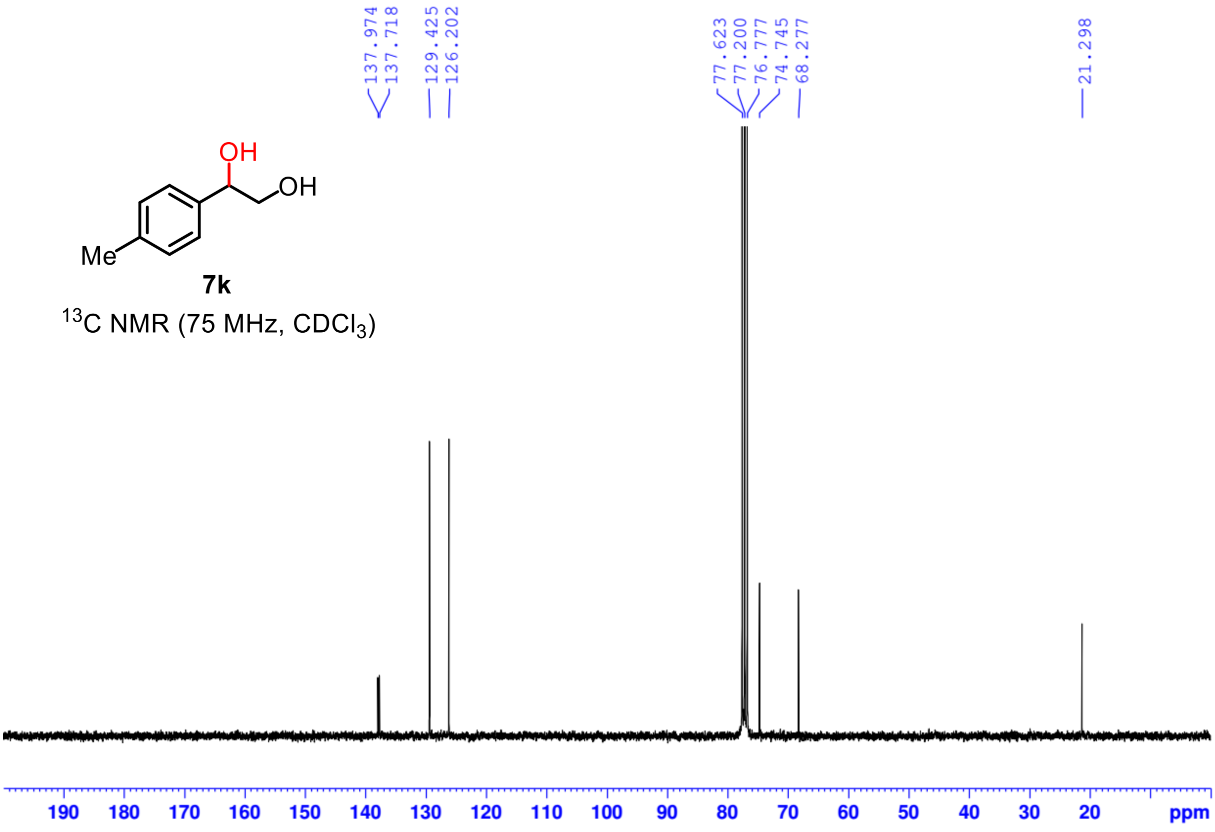
137.974
137.718
129.425
126.202
77.623
77.200
76.777
74.745
68.277
21.298
OH
OH
Me
7k
^{13}C NMR (75 MHz, $CDCl_3$)
190
180
170
160
150
140
130
120
110
100
90
80
70
60
50
40
30
20
ppm

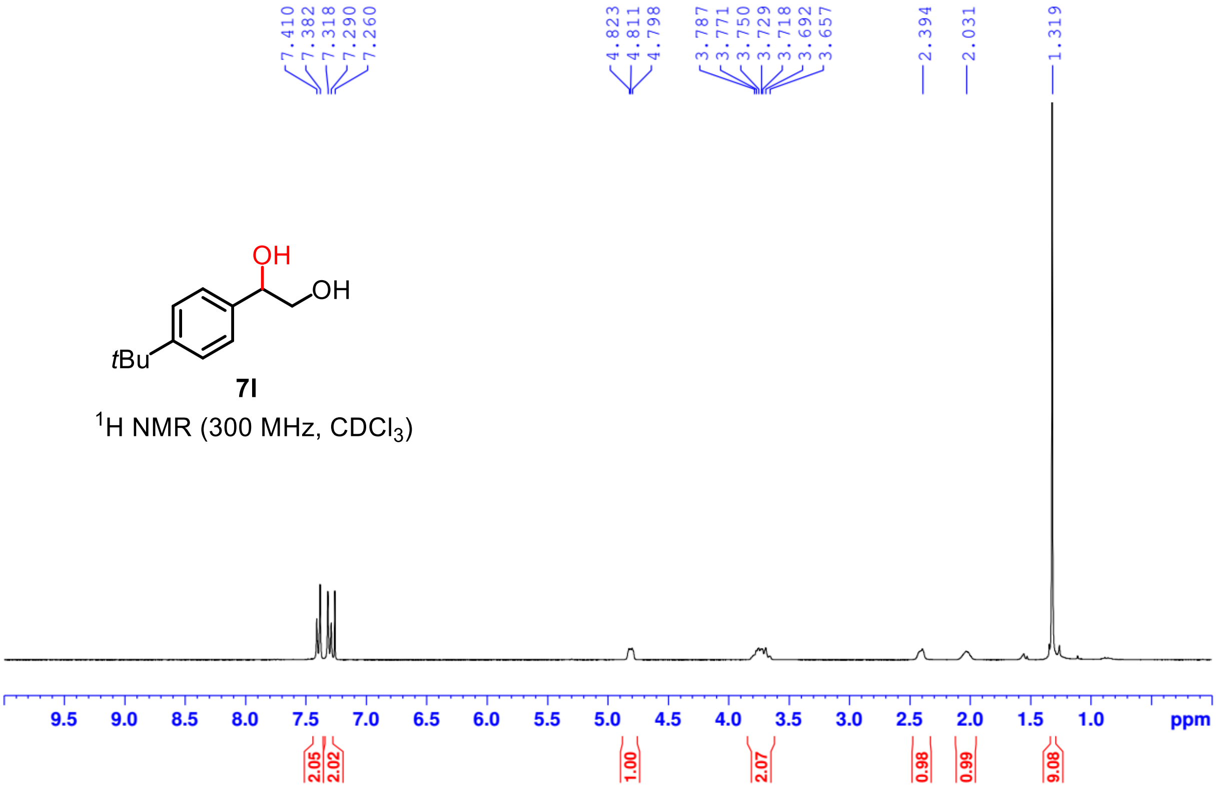
7.410
7.382
7.318
7.290
7.260
4.823
4.811
4.798
3.787
3.771
3.750
3.729
3.718
3.692
3.657
2.394
2.031
1.319
OH
OH
tBu
7l
1H NMR (300 MHz, CDCl3)
9.5
9.0
8.5
8.0
7.5
7.0
6.5
6.0
5.5
5.0
4.5
4.0
3.5
3.0
2.5
2.0
1.5
1.0
ppm
2.05
2.02
1.00
2.07
0.98
0.99
9.08

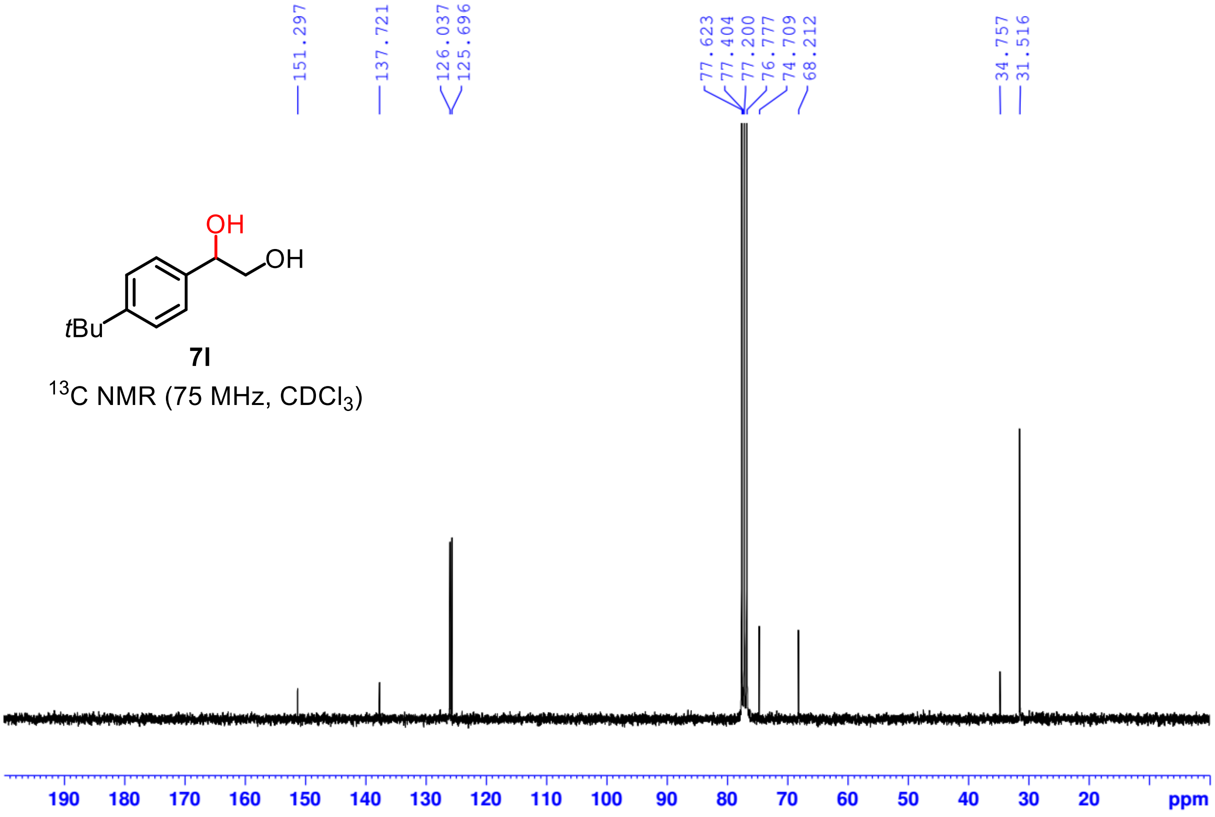
151.297
137.721
126.037
125.696
77.623
77.404
77.200
76.777
74.709
68.212
34.757
31.516
OH
OH
tBu
7l
13C NMR (75 MHz, CDCl3)
190
180
170
160
150
140
130
120
110
100
90
80
70
60
50
40
30
20
ppm

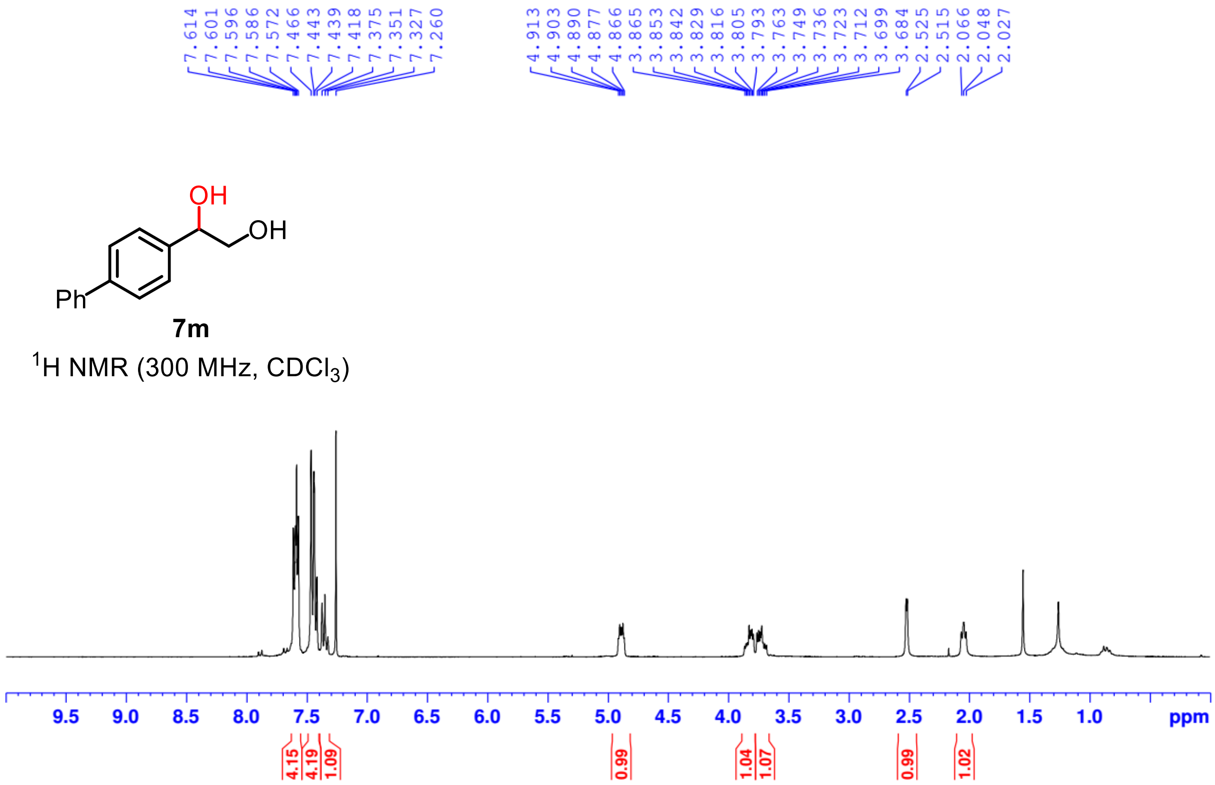

7.614
7.601
7.596
7.586
7.572
7.466
7.443
7.439
7.418
7.375
7.351
7.327
7.260
4.913
4.903
4.890
4.877
4.866
3.865
3.853
3.842
3.829
3.816
3.805
3.793
3.763
3.749
3.736
3.723
3.712
3.699
3.684
2.525
2.515
2.066
2.048
2.027
OH
OH
Ph
7m
^{1}H NMR (300 MHz, $CDCl_3$)
9.5
9.0
8.5
8.0
7.5
7.0
6.5
6.0
5.5
5.0
4.5
4.0
3.5
3.0
2.5
2.0
1.5
1.0
ppm
4.15
4.19
1.09
0.99
1.04
1.07
0.99
1.02

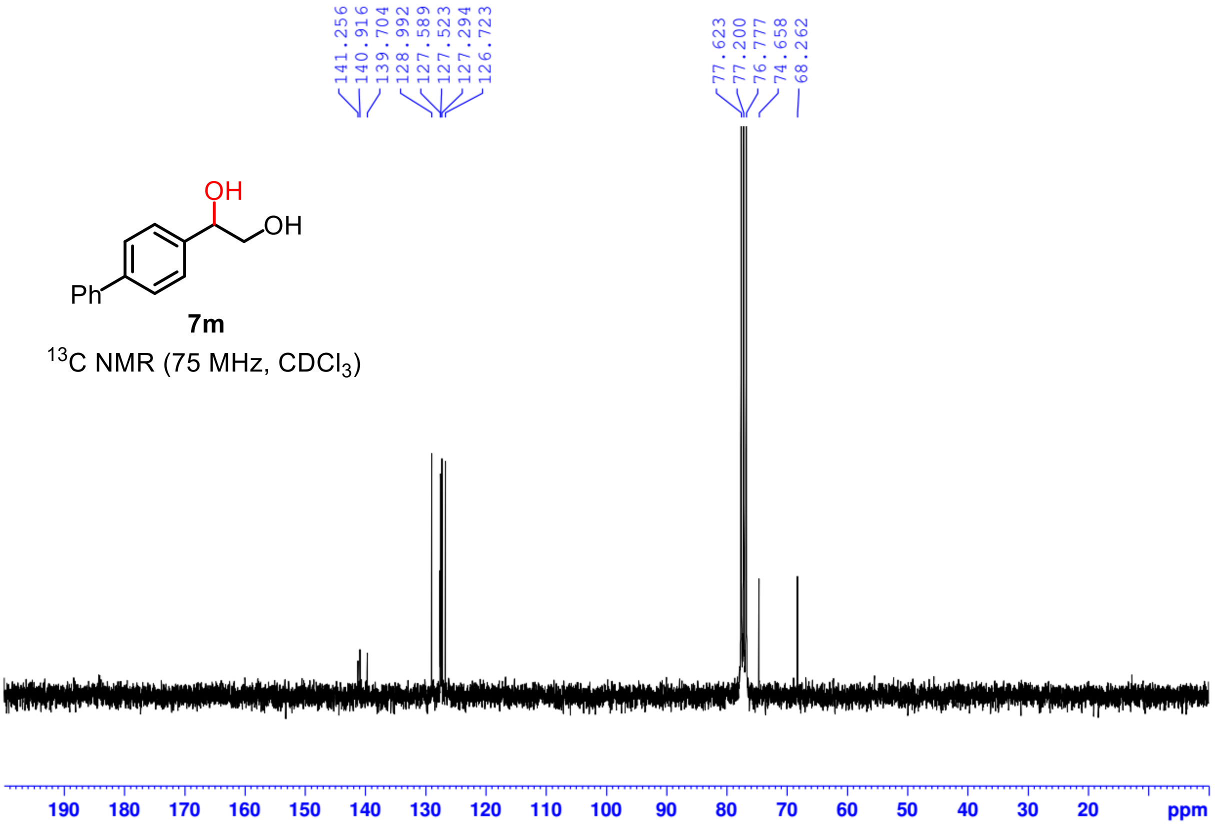

141.256
140.916
139.704
128.992
127.589
127.523
127.294
126.723
77.623
77.200
76.777
74.658
68.262
OH
OH
Ph
7m
^{13}C NMR (75 MHz, $CDCl_3$)
190
180
170
160
150
140
130
120
110
100
90
80
70
60
50
40
30
20
ppm

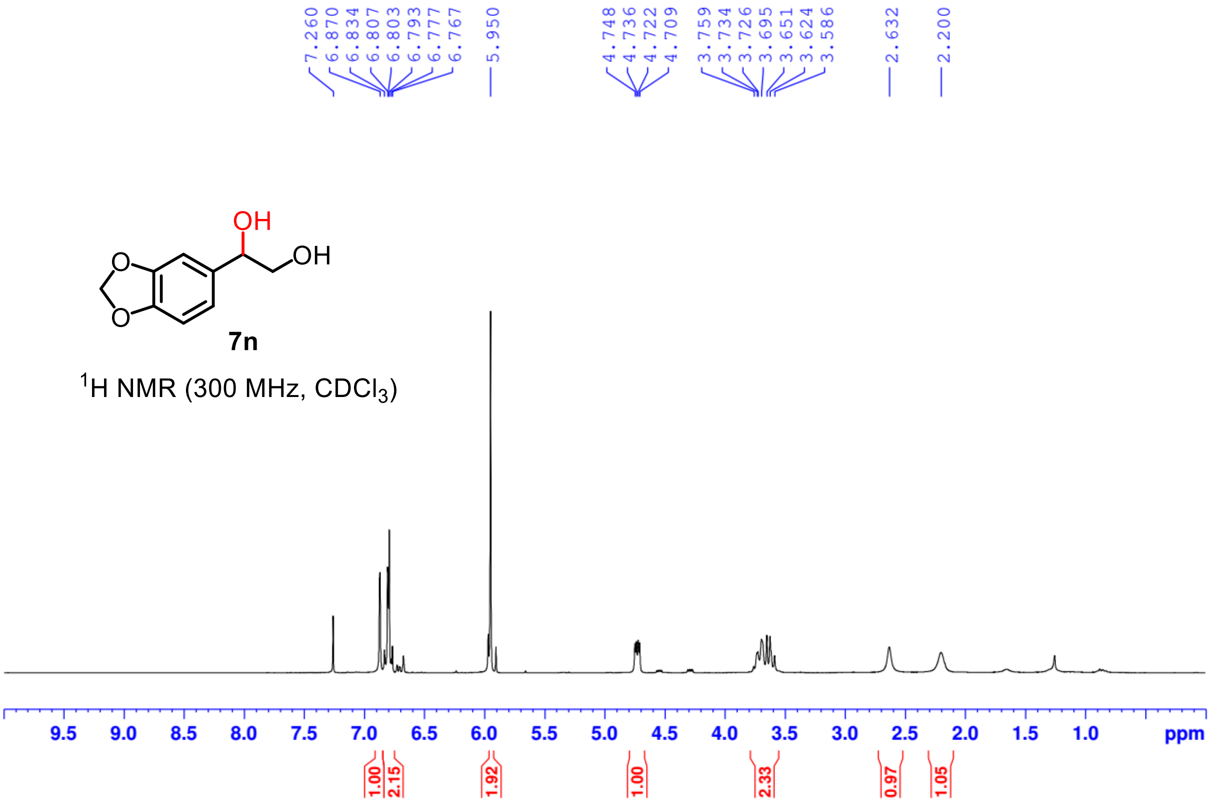
7.260
6.870
6.834
6.807
6.803
6.793
6.777
6.767
5.950
4.748
4.736
4.722
4.709
3.759
3.734
3.726
3.695
3.651
3.624
3.586
2.632
2.200
OH
OH
O
O
7n
^{1}H NMR (300 MHz, $CDCl_3$)
9.5 9.0 8.5 8.0 7.5 7.0 6.5 6.0 5.5 5.0 4.5 4.0 3.5 3.0 2.5 2.0 1.5 1.0 ppm
1.00
2.15
1.92
1.00
2.33
0.97
1.05

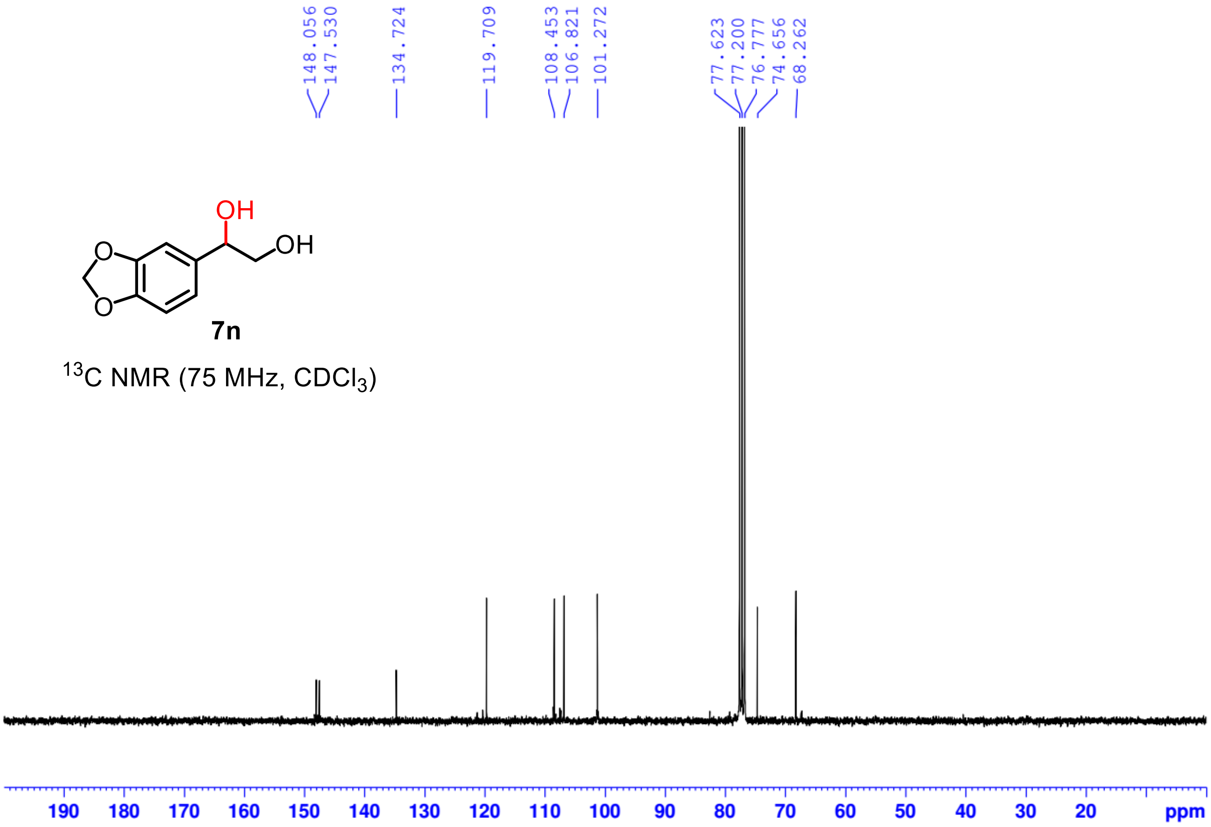
148.056
147.530
134.724
119.709
108.453
106.821
101.272
77.623
77.200
76.777
74.656
68.262
OH
OH
O
O
7n
^{13}C NMR (75 MHz, $CDCl_3$)
190 180 170 160 150 140 130 120 110 100 90 80 70 60 50 40 30 20 ppm

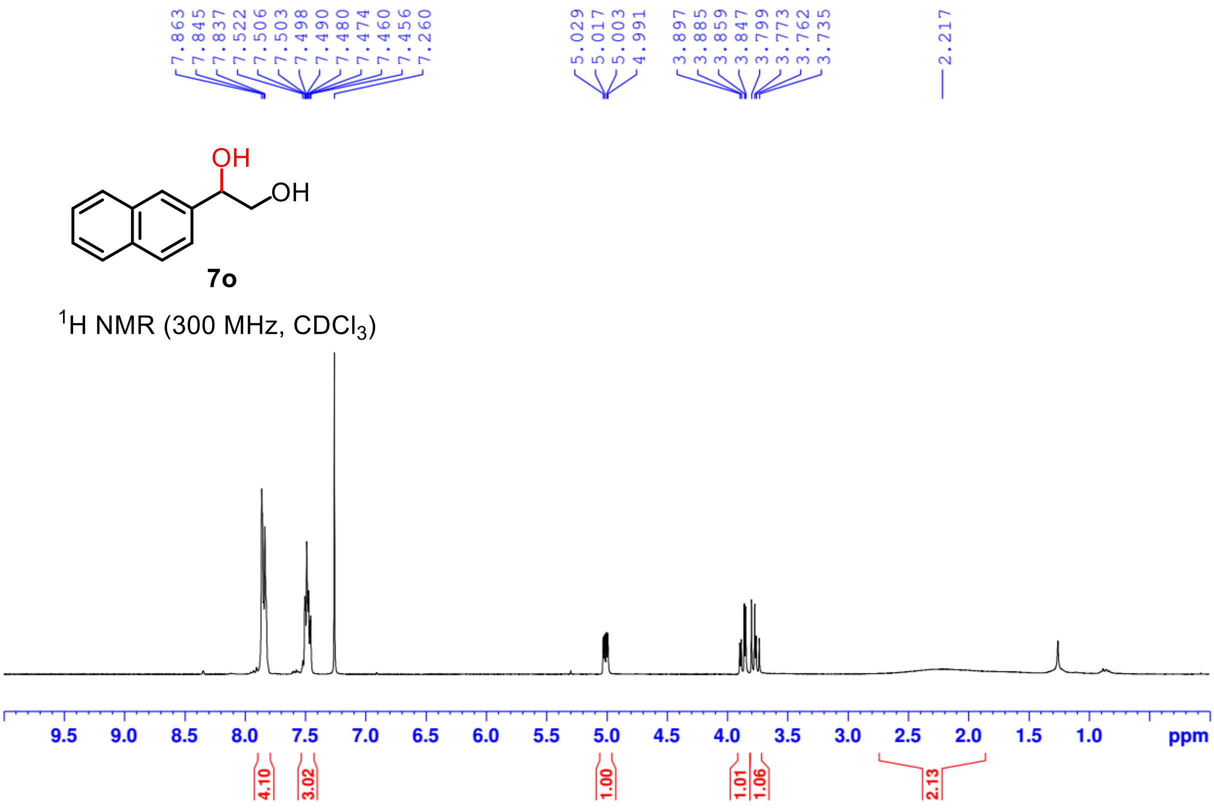
7.863
7.845
7.837
7.522
7.506
7.503
7.498
7.490
7.480
7.474
7.460
7.456
7.260
5.029
5.017
5.003
4.991
3.897
3.885
3.859
3.847
3.799
3.773
3.762
3.735
2.217
OH
OH
7o
1H NMR (300 MHz, CDCl3)
9.5 9.0 8.5 8.0 7.5 7.0 6.5 6.0 5.5 5.0 4.5 4.0 3.5 3.0 2.5 2.0 1.5 1.0 ppm
4.10
3.02
1.00
1.01
1.06
2.13

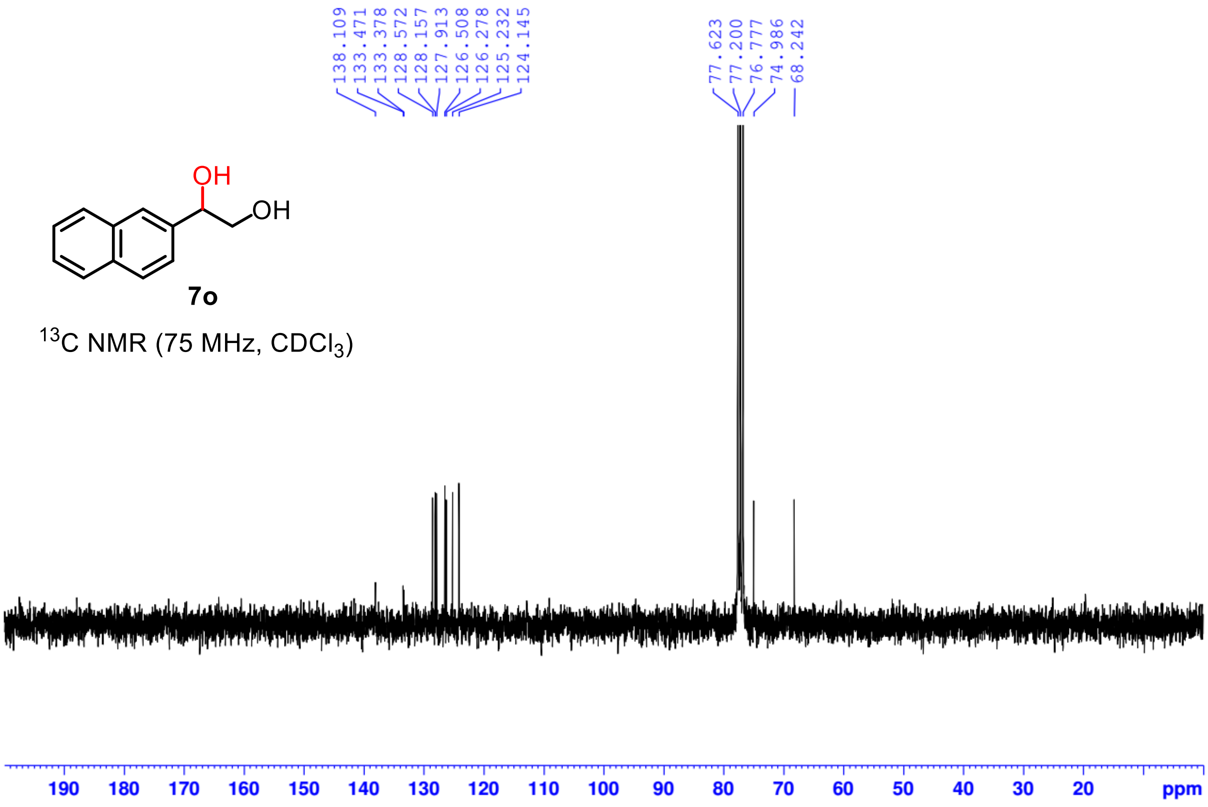
138.109
133.471
133.378
128.572
128.157
127.913
126.508
126.278
125.232
124.145
77.623
77.200
76.777
74.986
68.242
OH
OH
7o
13C NMR (75 MHz, CDCl3)
190 180 170 160 150 140 130 120 110 100 90 80 70 60 50 40 30 20 ppm

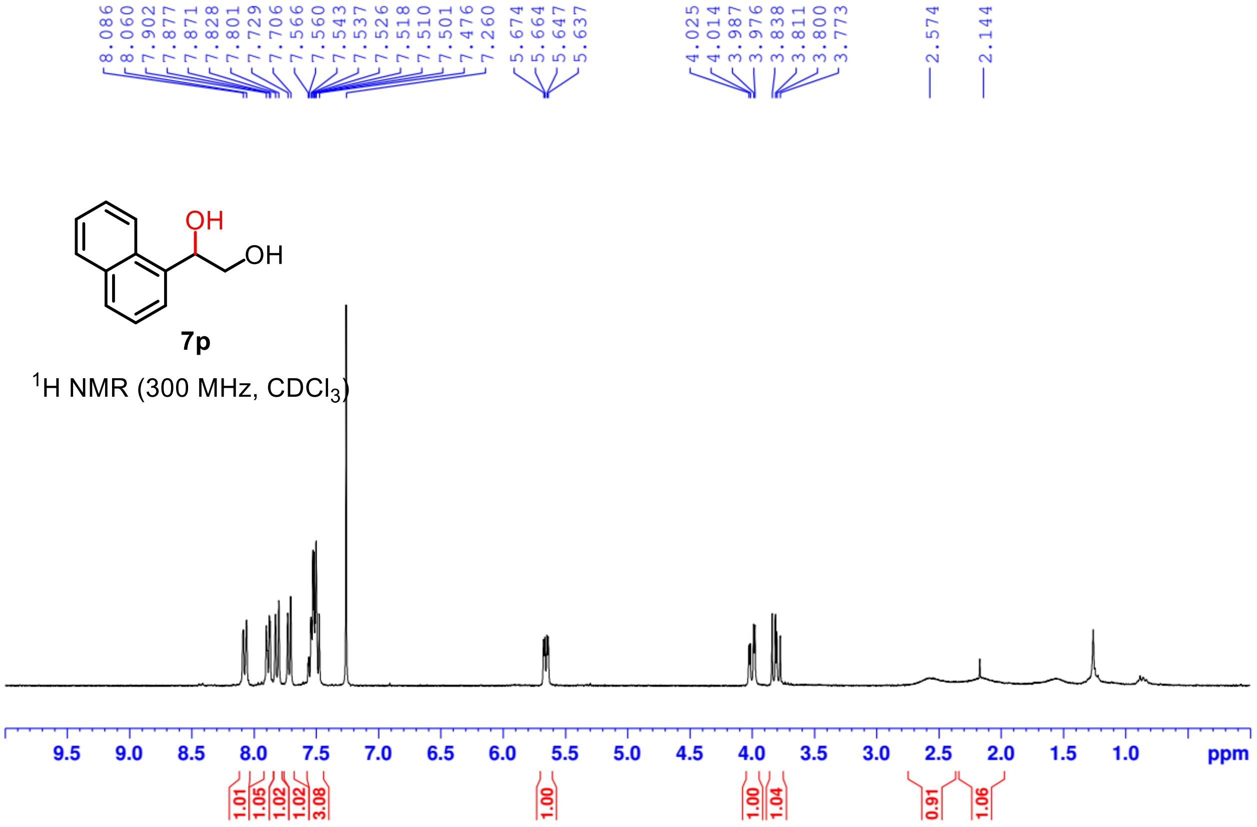
8.086 8.060 7.902 7.877 7.871 7.828 7.801 7.729 7.706 7.566 7.560 7.543 7.537 7.526 7.518 7.510 7.501 7.476 7.260 5.674 5.664 5.647 5.637
4.025 4.014 3.987 3.976 3.838 3.811 3.800 3.773
2.574
2.144
OH
OH
7p
^{1}H NMR (300 MHz, CDCl$_3$)
9.5 9.0 8.5 8.0 7.5 7.0 6.5 6.0 5.5 5.0 4.5 4.0 3.5 3.0 2.5 2.0 1.5 1.0 ppm
1.01 1.05 1.02 1.02 3.08 1.00 1.00 1.04 0.91 1.06

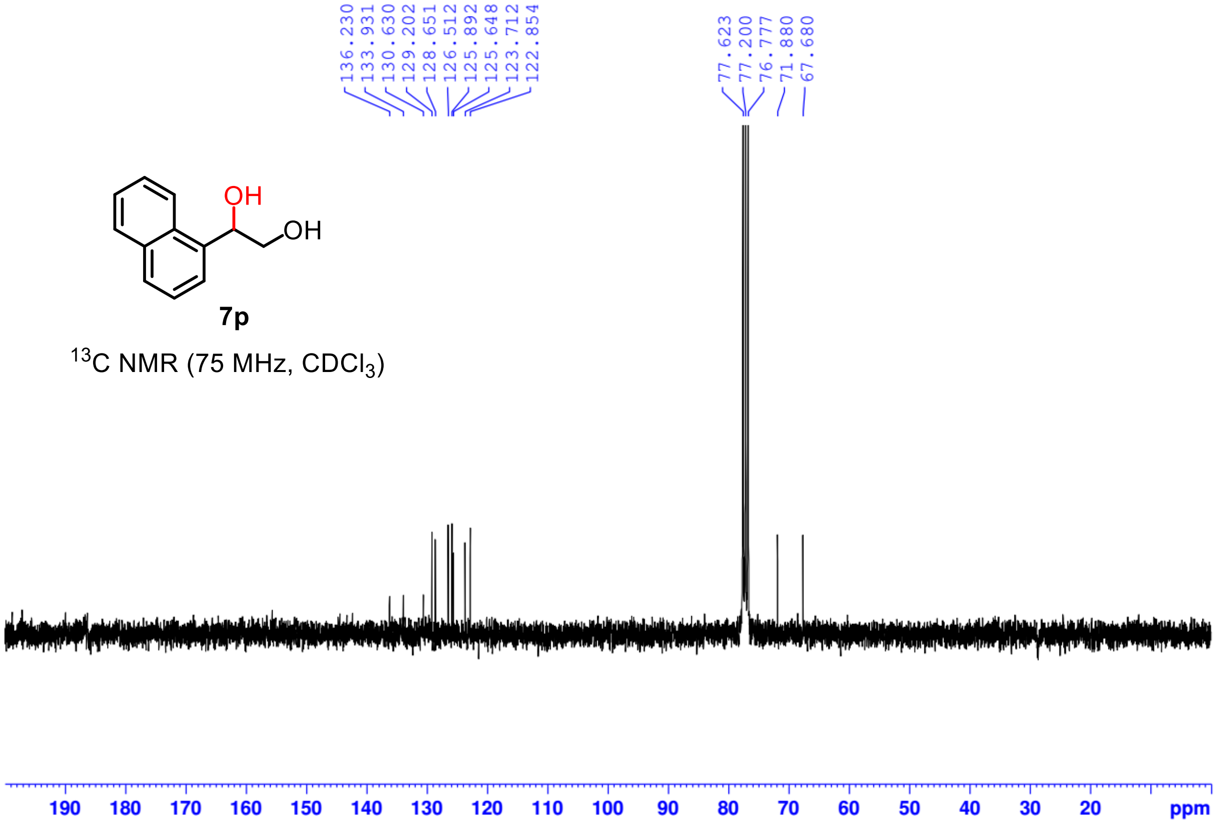
136.230 133.931 130.630 129.202 128.651 126.512 125.892 125.648 123.712 122.854
77.623 77.200 76.777 71.880 67.680
OH
OH
7p
^{13}C NMR (75 MHz, CDCl$_3$)
190 180 170 160 150 140 130 120 110 100 90 80 70 60 50 40 30 20 ppm

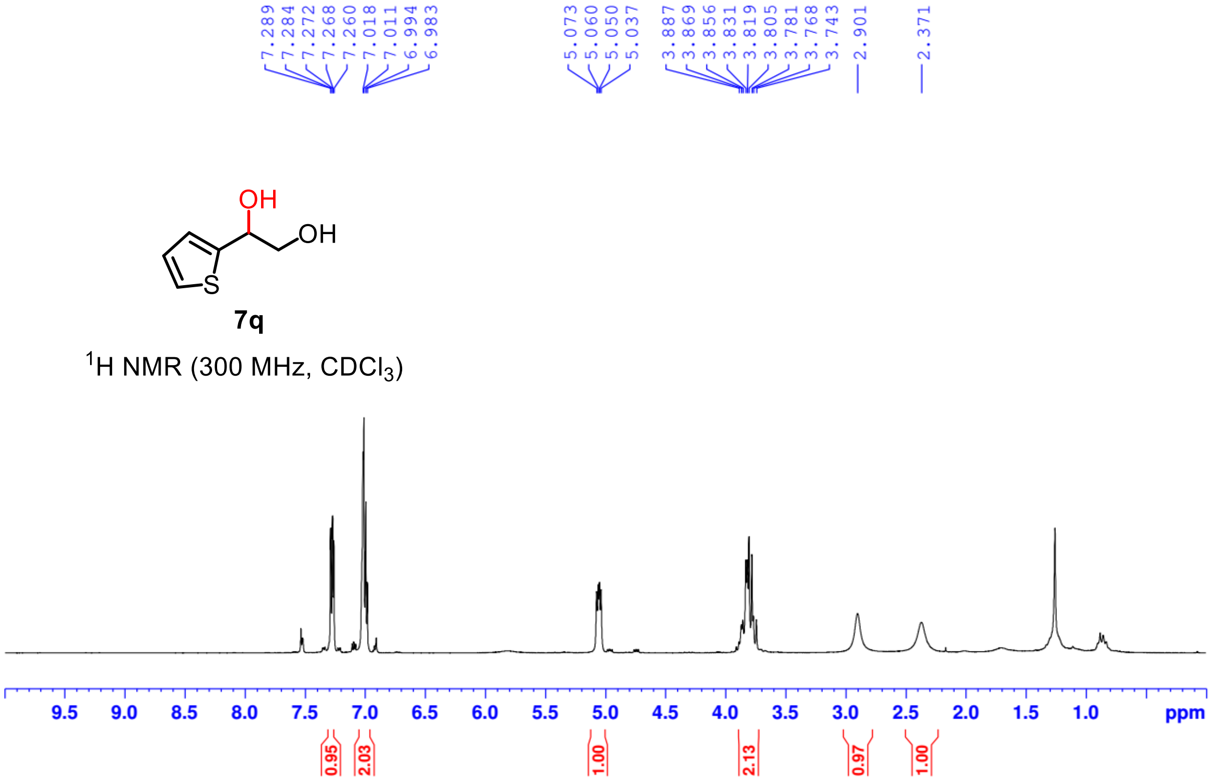
7.289
7.284
7.272
7.268
7.260
7.018
7.011
6.994
6.983
5.073
5.060
5.050
5.037
3.887
3.869
3.856
3.831
3.819
3.805
3.781
3.768
3.743
2.901
2.371
OH
OH
S
7q
^{1}H NMR (300 MHz, $CDCl_3$)
9.5 9.0 8.5 8.0 7.5 7.0 6.5 6.0 5.5 5.0 4.5 4.0 3.5 3.0 2.5 2.0 1.5 1.0 ppm
0.95
2.03
1.00
2.13
0.97
1.00

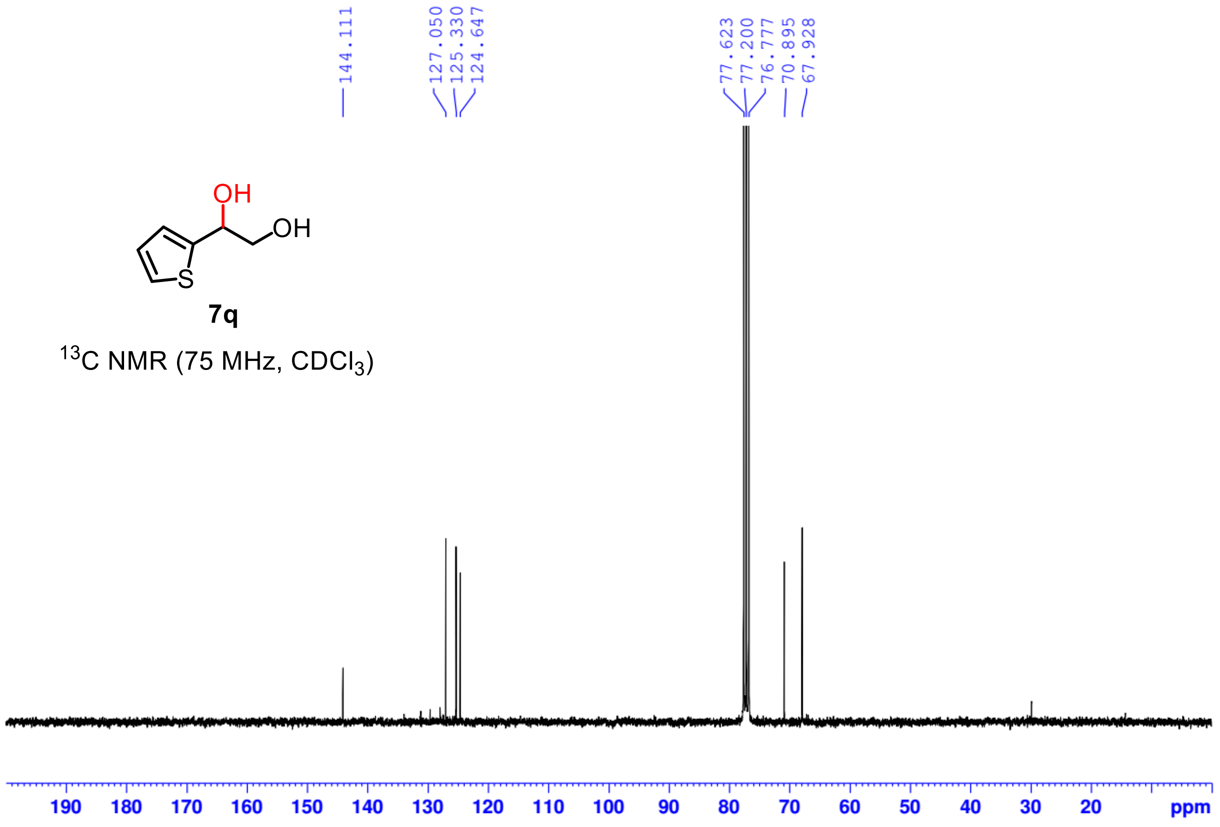
144.111
127.050
125.330
124.647
77.623
77.200
76.777
70.895
67.928
OH
OH
S
7q
^{13}C NMR (75 MHz, $CDCl_3$)
190 180 170 160 150 140 130 120 110 100 90 80 70 60 50 40 30 20 ppm

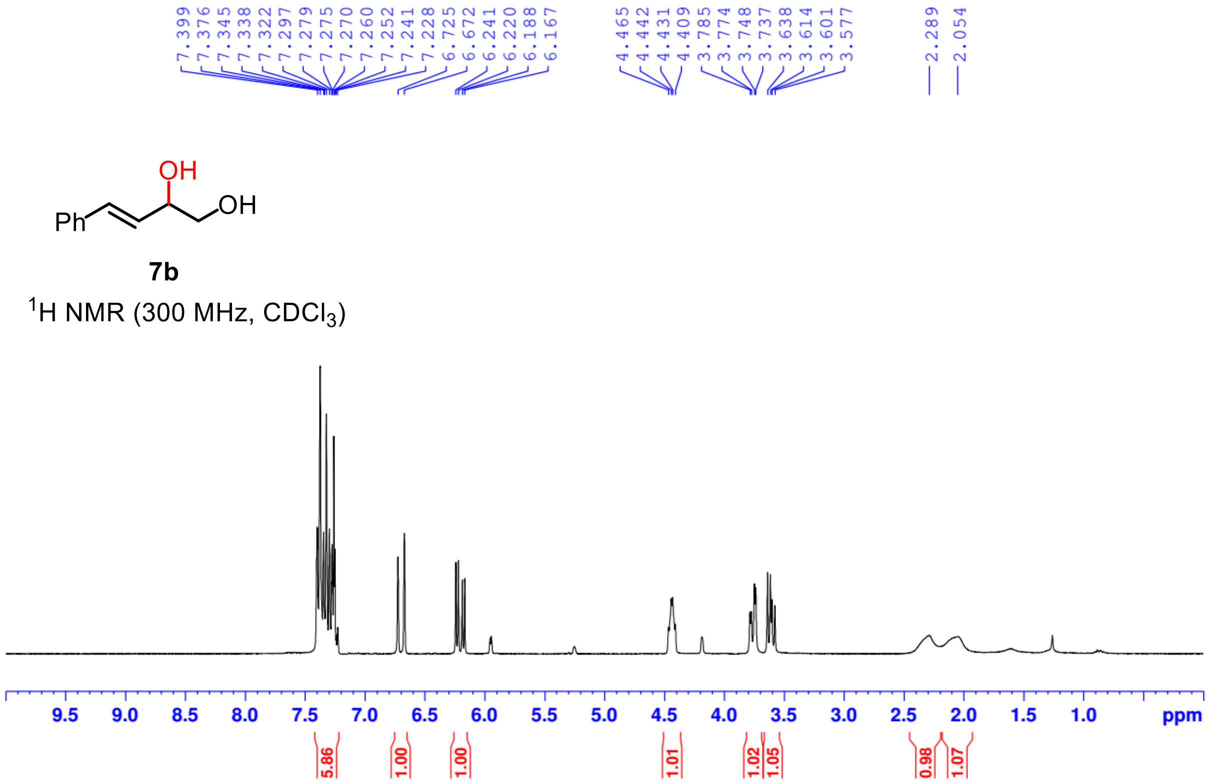
7.399
7.376
7.345
7.338
7.322
7.297
7.279
7.275
7.270
7.260
7.252
7.241
7.228
6.725
6.672
6.241
6.220
6.188
6.167
4.465
4.442
4.431
4.409
3.785
3.774
3.748
3.737
3.638
3.614
3.601
3.577
2.289
2.054
OH
Ph
OH
7b
¹H NMR (300 MHz, CDCl3)
9.5
9.0
8.5
8.0
7.5
7.0
6.5
6.0
5.5
5.0
4.5
4.0
3.5
3.0
2.5
2.0
1.5
1.0
ppm
5.86
1.00
1.00
1.01
1.02
1.05
0.98
1.07

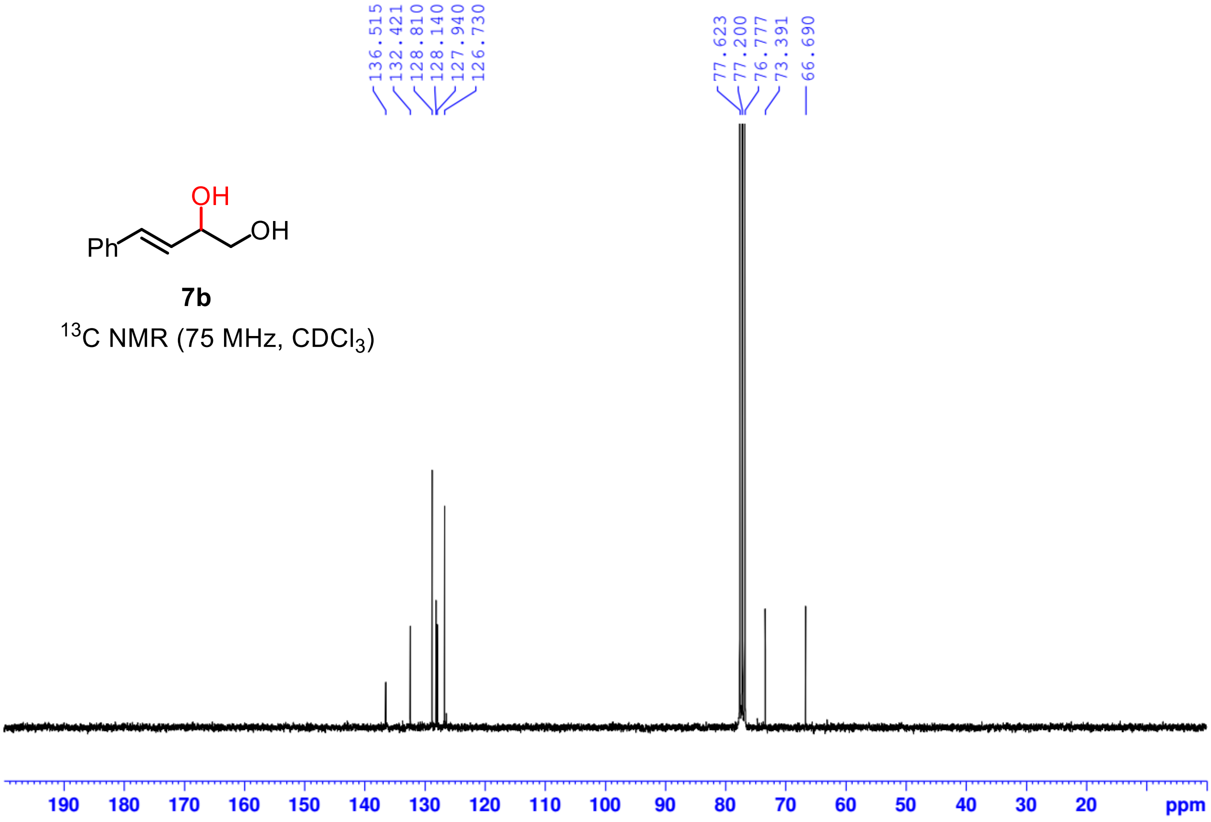
136.515
132.421
128.810
128.140
127.940
126.730
77.623
77.200
76.777
73.391
66.690
OH
Ph
OH
7b
¹³C NMR (75 MHz, CDCl3)
190
180
170
160
150
140
130
120
110
100
90
80
70
60
50
40
30
20
ppm

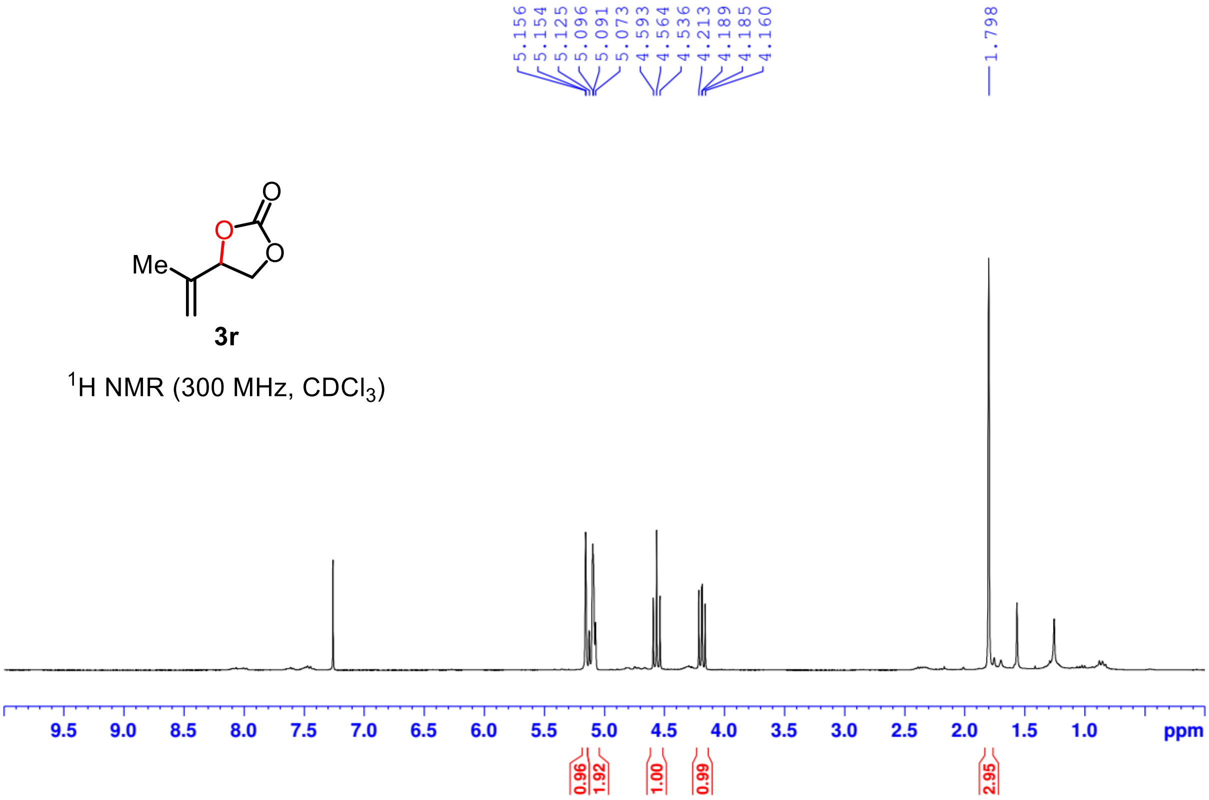
5.156
5.154
5.125
5.096
5.091
5.073
4.593
4.564
4.536
4.213
4.189
4.185
4.160
1.798
O
O
O
Me
3r
^{1}H NMR (300 MHz, $CDCl_3$)
9.5 9.0 8.5 8.0 7.5 7.0 6.5 6.0 5.5 5.0 4.5 4.0 3.5 3.0 2.5 2.0 1.5 1.0 ppm
0.96
1.92
1.00
0.99
2.95

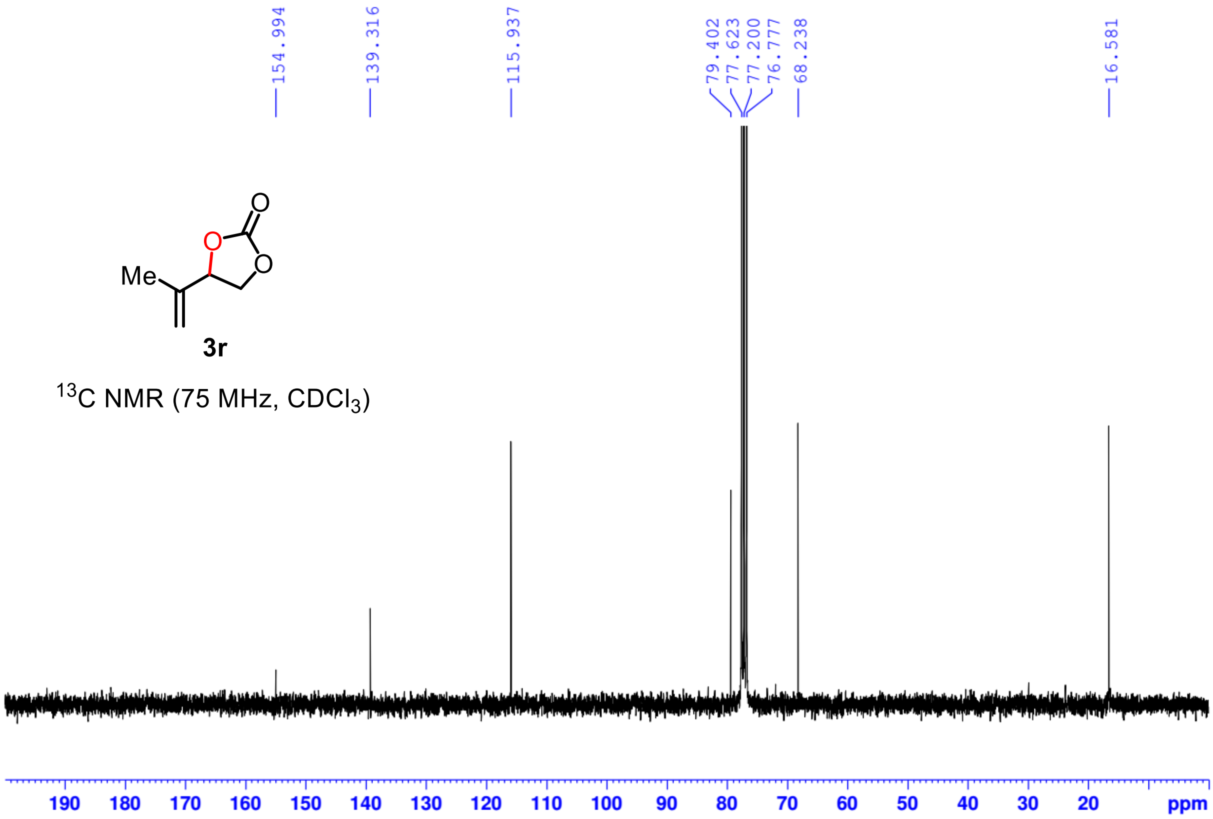
154.994
139.316
115.937
79.402
77.623
77.200
76.777
68.238
16.581
O
O
O
Me
3r
^{13}C NMR (75 MHz, $CDCl_3$)
190 180 170 160 150 140 130 120 110 100 90 80 70 60 50 40 30 20 ppm

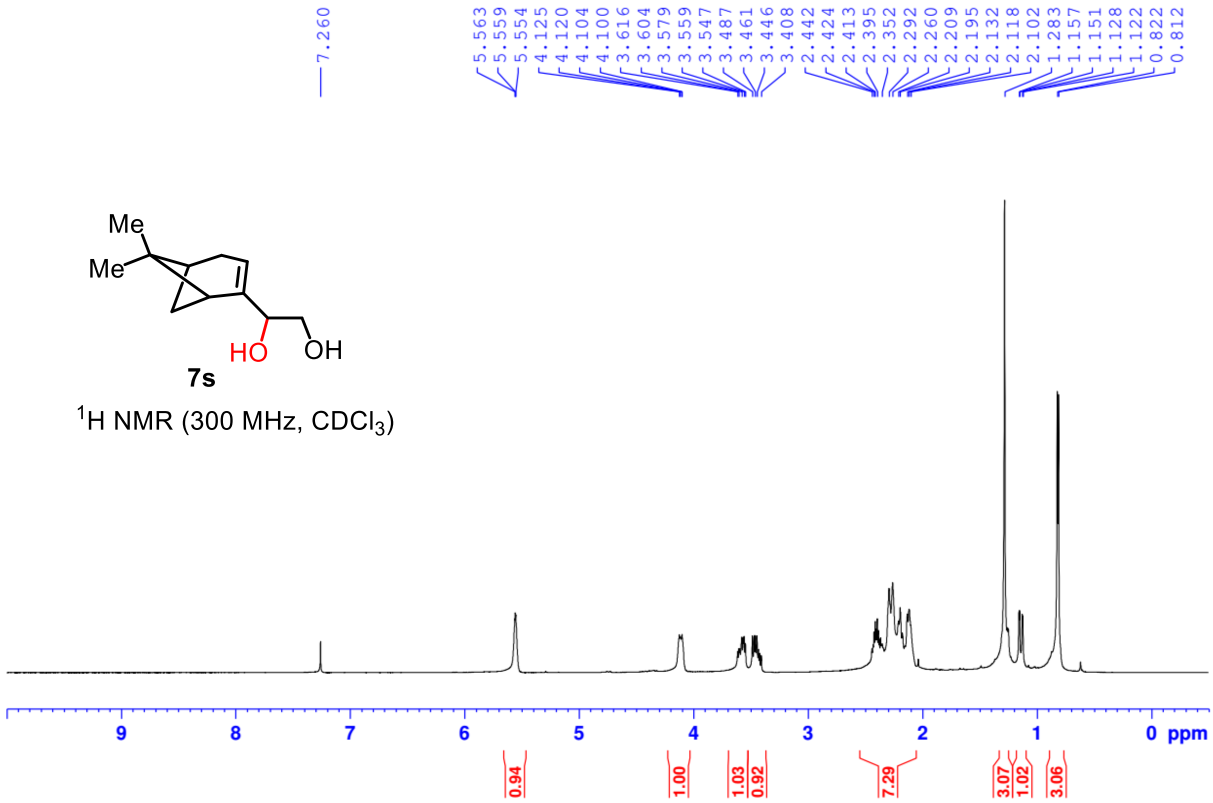

7.260
5.563
5.559
5.554
4.125
4.120
4.104
4.100
3.616
3.604
3.579
3.559
3.547
3.487
3.461
3.446
3.408
2.442
2.424
2.413
2.395
2.352
2.292
2.260
2.209
2.195
2.132
2.118
2.102
1.283
1.157
1.151
1.128
1.122
0.822
0.812
Me
Me
HO
OH
7s
1H NMR (300 MHz, CDCl3)
9
8
7
6
5
4
3
2
1
0 ppm
0.94
1.00
1.03
0.92
7.29
3.07
1.02
3.06

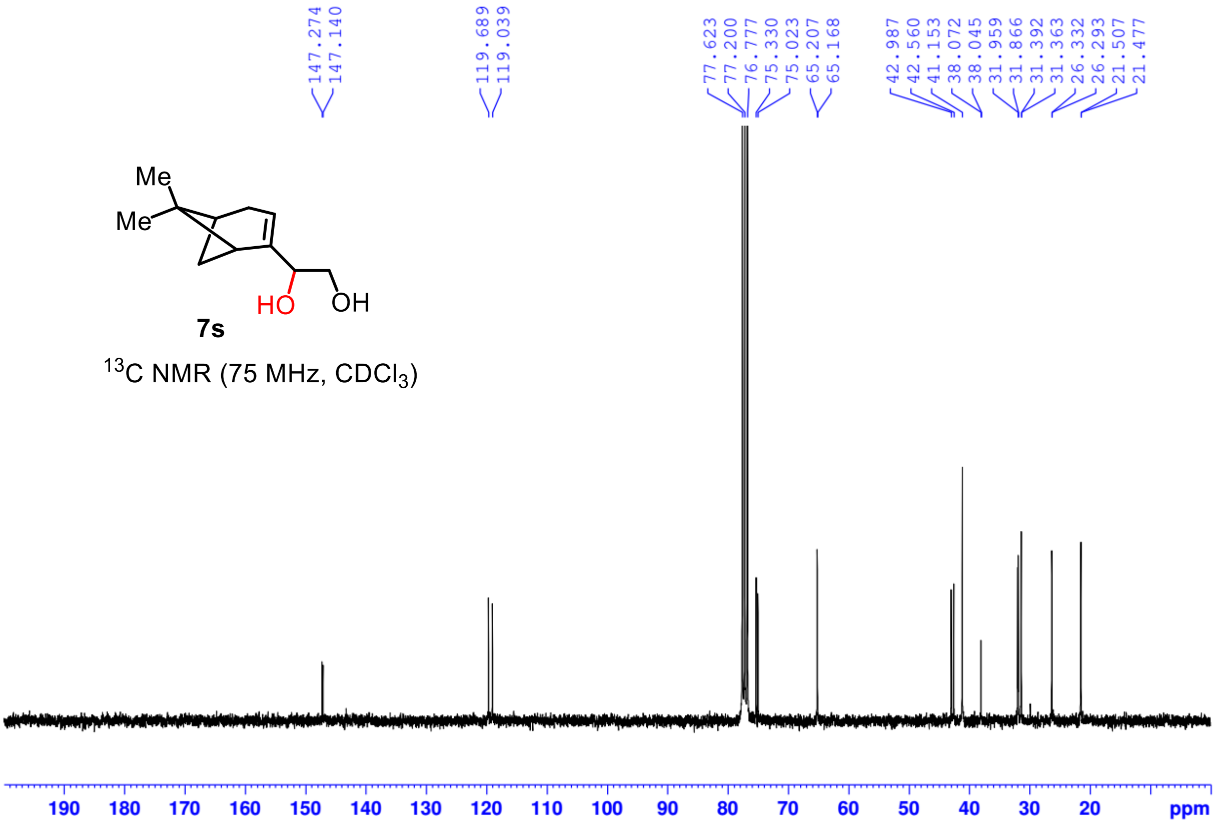

147.274
147.140
119.689
119.039
77.623
77.200
76.777
75.330
75.023
65.207
65.168
42.987
42.560
41.153
38.072
38.045
31.959
31.866
31.392
31.363
26.332
26.293
21.507
21.477
Me
Me
HO
OH
7s
13C NMR (75 MHz, CDCl3)
190
180
170
160
150
140
130
120
110
100
90
80
70
60
50
40
30
20
ppm

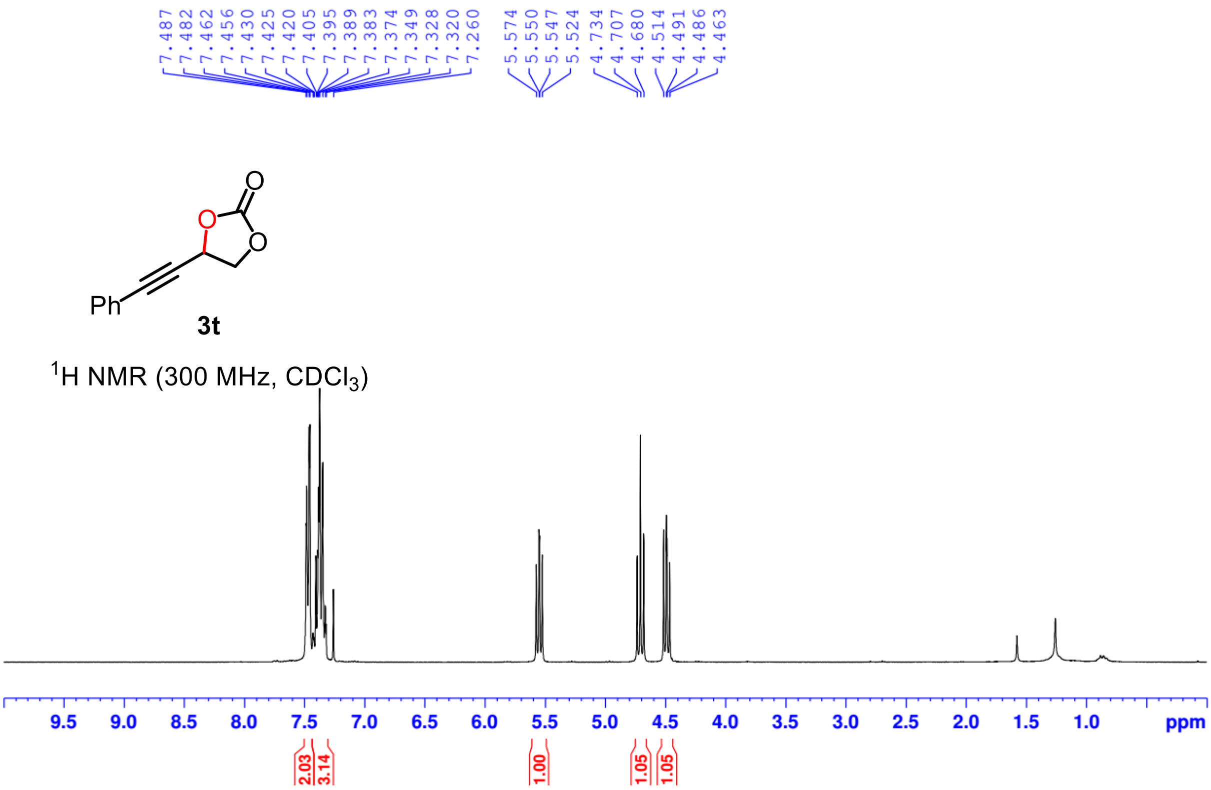
7.487
7.482
7.462
7.456
7.430
7.425
7.420
7.405
7.395
7.389
7.383
7.374
7.349
7.328
7.320
7.260
5.574
5.550
5.547
5.524
4.734
4.707
4.680
4.514
4.491
4.486
4.463
O
O
O
Ph
3t
1H NMR (300 MHz, CDCl3)
9.5 9.0 8.5 8.0 7.5 7.0 6.5 6.0 5.5 5.0 4.5 4.0 3.5 3.0 2.5 2.0 1.5 1.0 ppm
2.03
3.14
1.00
1.05
1.05

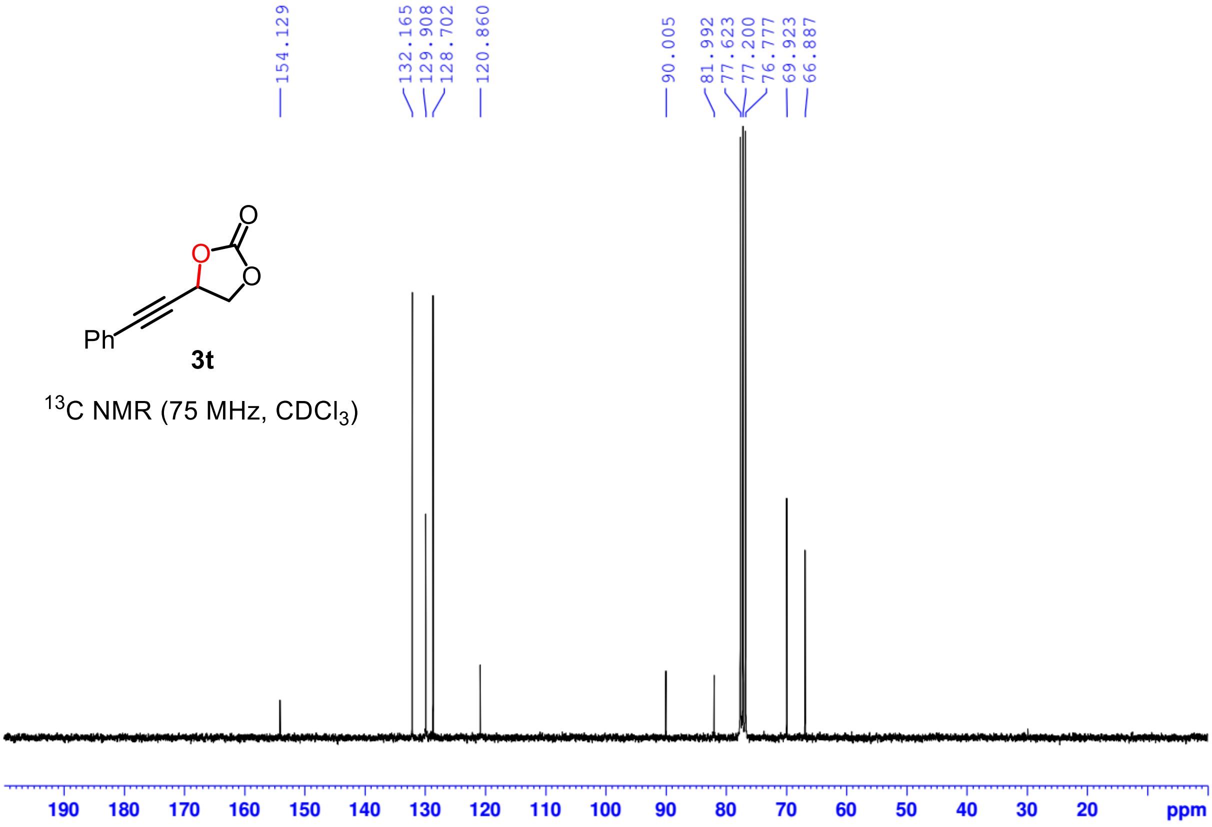
154.129
132.165
129.908
128.702
120.860
90.005
81.992
77.623
77.200
76.777
69.923
66.887
O
O
O
Ph
3t
13C NMR (75 MHz, CDCl3)
190 180 170 160 150 140 130 120 110 100 90 80 70 60 50 40 30 20 ppm

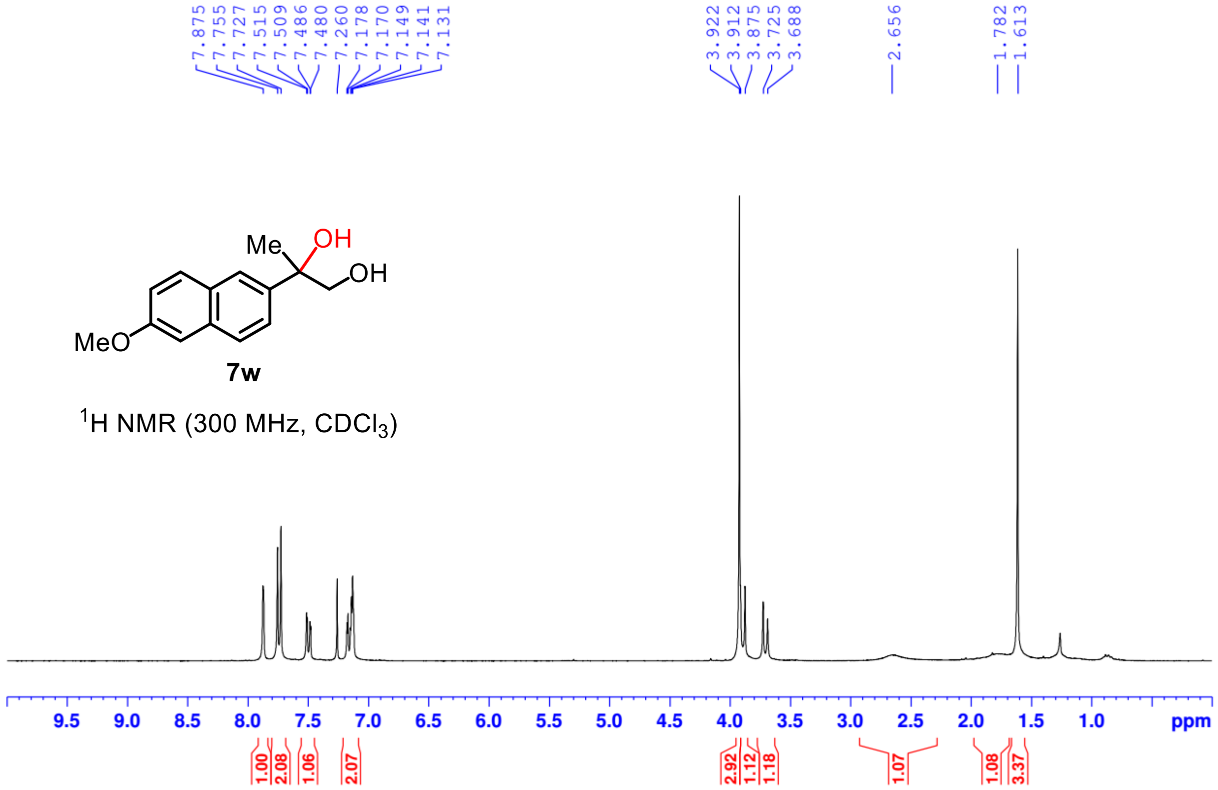
7.875
7.755
7.727
7.515
7.509
7.486
7.480
7.260
7.178
7.170
7.149
7.141
7.131
3.922
3.912
3.875
3.725
3.688
2.656
1.782
1.613
Me
OH
OH
MeO
7w
^{1}H NMR (300 MHz, CDCl$_3$)
9.5
9.0
8.5
8.0
7.5
7.0
6.5
6.0
5.5
5.0
4.5
4.0
3.5
3.0
2.5
2.0
1.5
1.0
ppm
1.00
2.08
1.06
2.07
2.92
1.12
1.18
1.07
1.08
3.37

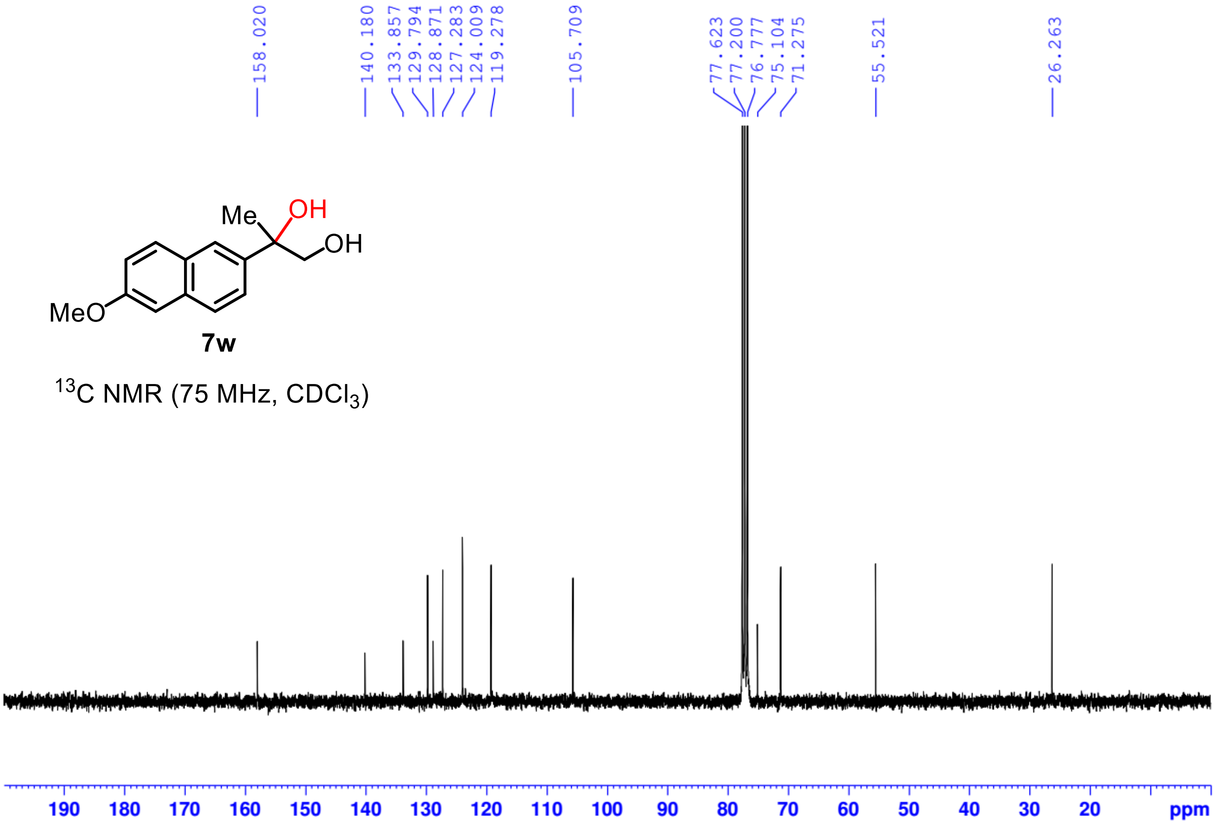
158.020
140.180
133.857
129.794
128.871
127.283
124.009
119.278
105.709
77.623
77.200
76.777
75.104
71.275
55.521
26.263
Me
OH
OH
MeO
7w
^{13}C NMR (75 MHz, CDCl$_3$)
190
180
170
160
150
140
130
120
110
100
90
80
70
60
50
40
30
20
ppm

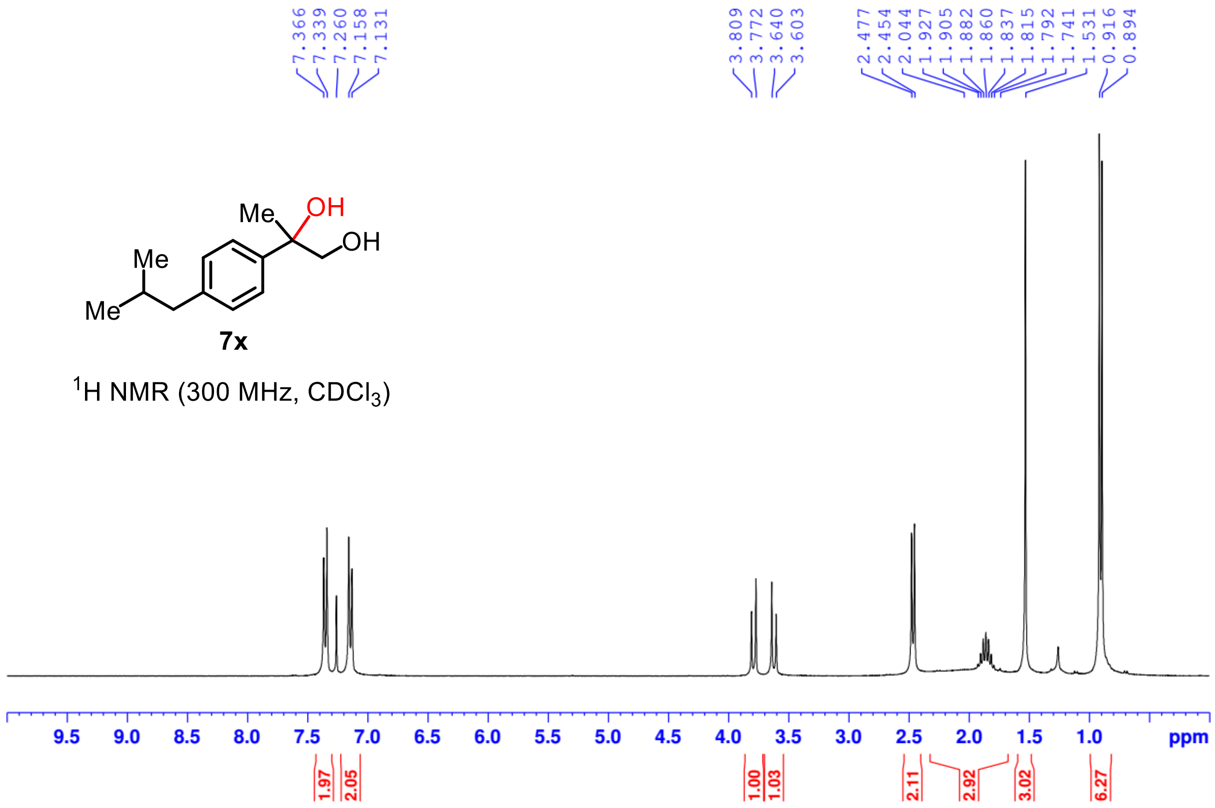
7.366
7.339
7.260
7.158
7.131
3.809
3.772
3.640
3.603
2.477
2.454
2.044
1.927
1.905
1.882
1.860
1.837
1.815
1.792
1.741
1.531
0.916
0.894
Me
OH
OH
Me
Me
7x
1H NMR (300 MHz, CDCl3)
9.5
9.0
8.5
8.0
7.5
7.0
6.5
6.0
5.5
5.0
4.5
4.0
3.5
3.0
2.5
2.0
1.5
1.0
ppm
1.97
2.05
1.00
1.03
2.11
2.92
3.02
6.27

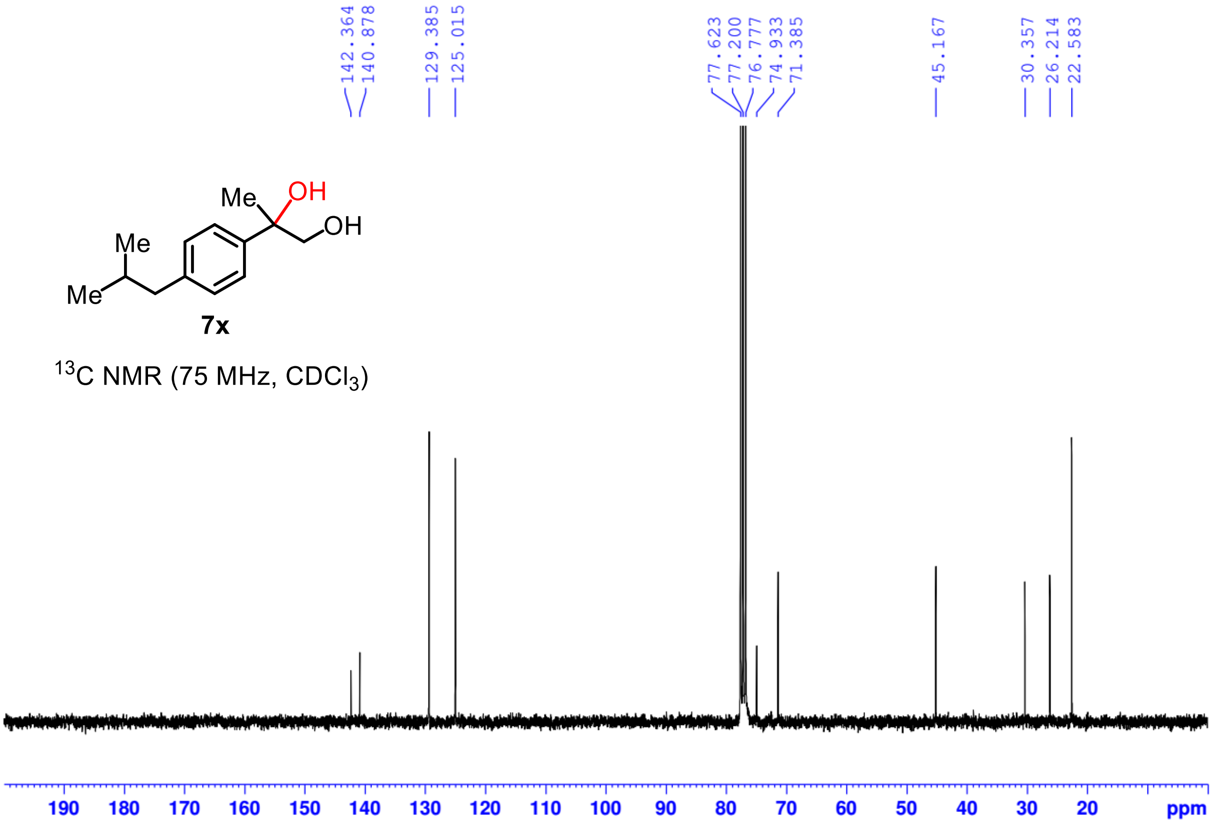
142.364
140.878
129.385
125.015
77.623
77.200
76.777
74.933
71.385
45.167
30.357
26.214
22.583
Me
OH
OH
Me
Me
7x
13C NMR (75 MHz, CDCl3)
190
180
170
160
150
140
130
120
110
100
90
80
70
60
50
40
30
20
ppm

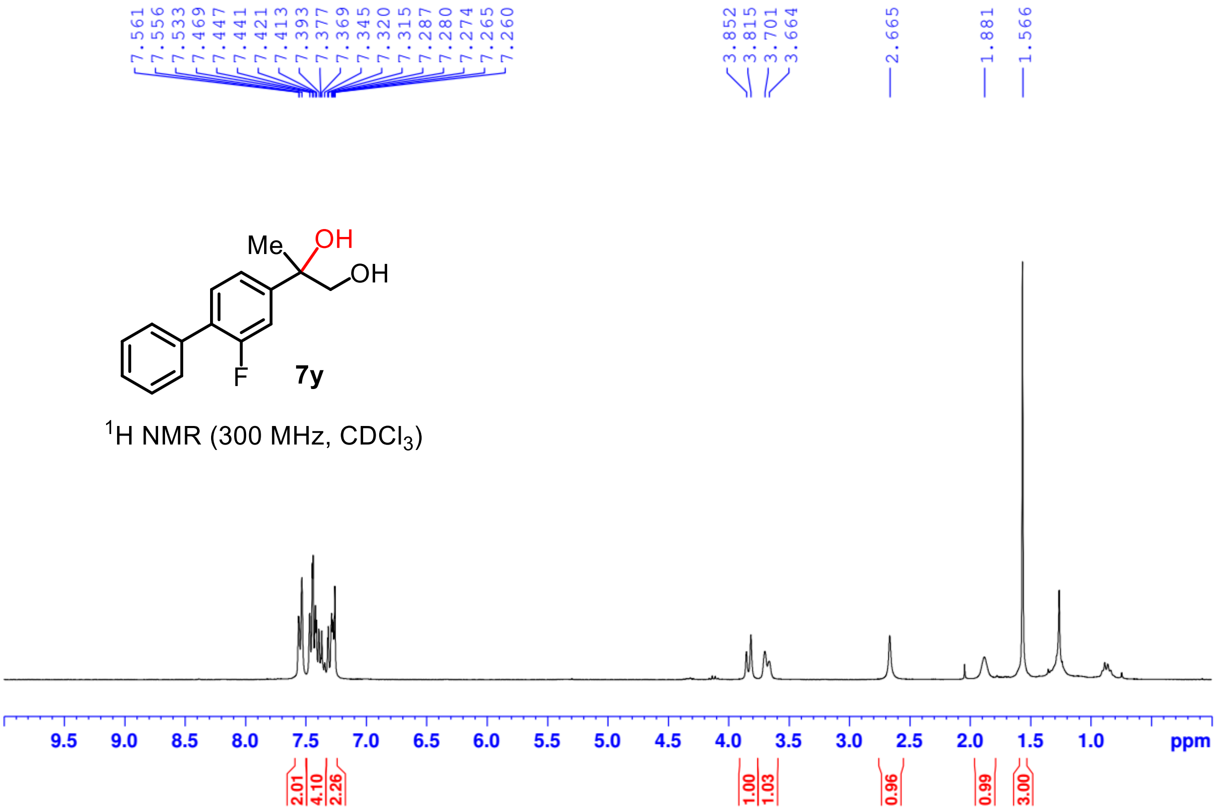
7.561
7.556
7.533
7.469
7.447
7.441
7.421
7.413
7.393
7.377
7.369
7.345
7.320
7.315
7.287
7.280
7.274
7.265
7.260
3.852
3.815
3.701
3.664
2.665
1.881
1.566
Me
OH
OH
F
7y
^{1}H NMR (300 MHz, CDCl$_3$)
9.5
9.0
8.5
8.0
7.5
7.0
6.5
6.0
5.5
5.0
4.5
4.0
3.5
3.0
2.5
2.0
1.5
1.0
ppm
2.01
4.10
2.26
1.00
1.03
0.96
0.99
3.00

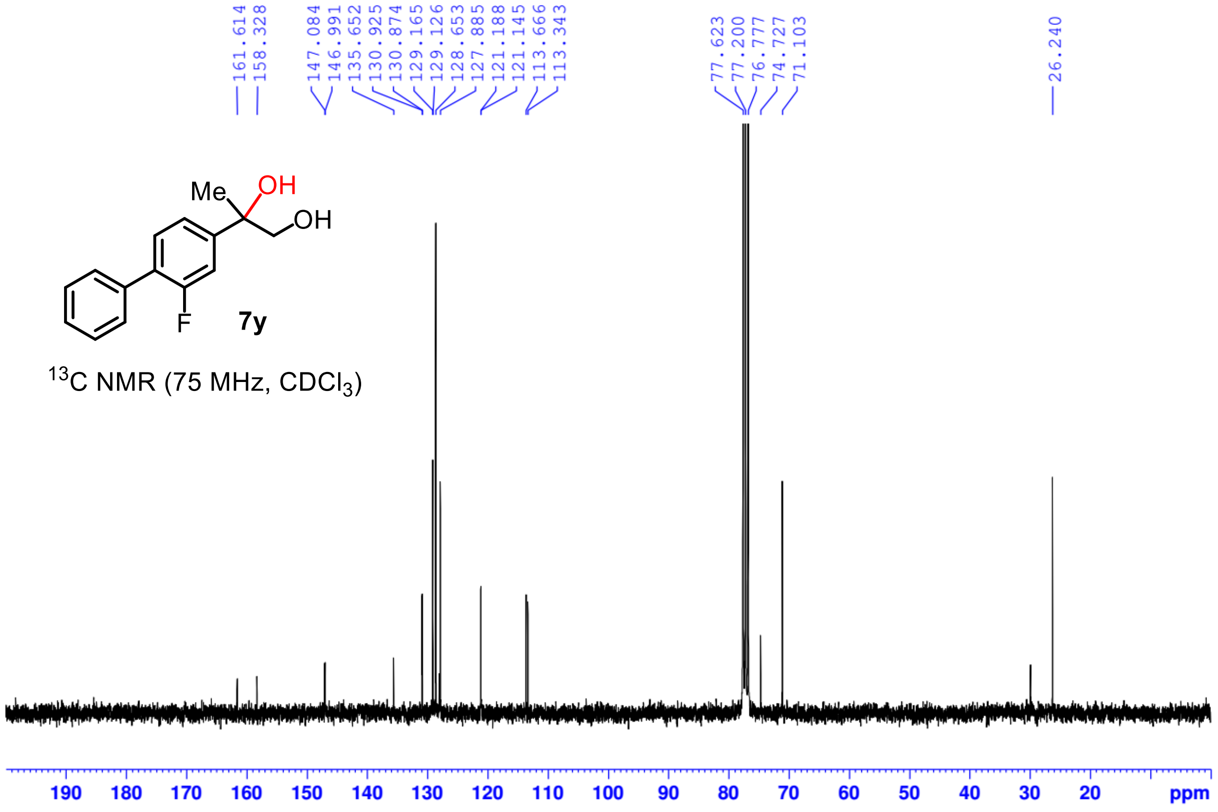
161.614
158.328
147.084
146.991
135.652
130.925
130.874
129.165
129.126
128.653
127.885
121.188
121.145
113.666
113.343
77.623
77.200
76.777
74.727
71.103
26.240
Me
OH
OH
F
7y
^{13}C NMR (75 MHz, CDCl$_3$)
190
180
170
160
150
140
130
120
110
100
90
80
70
60
50
40
30
20
ppm

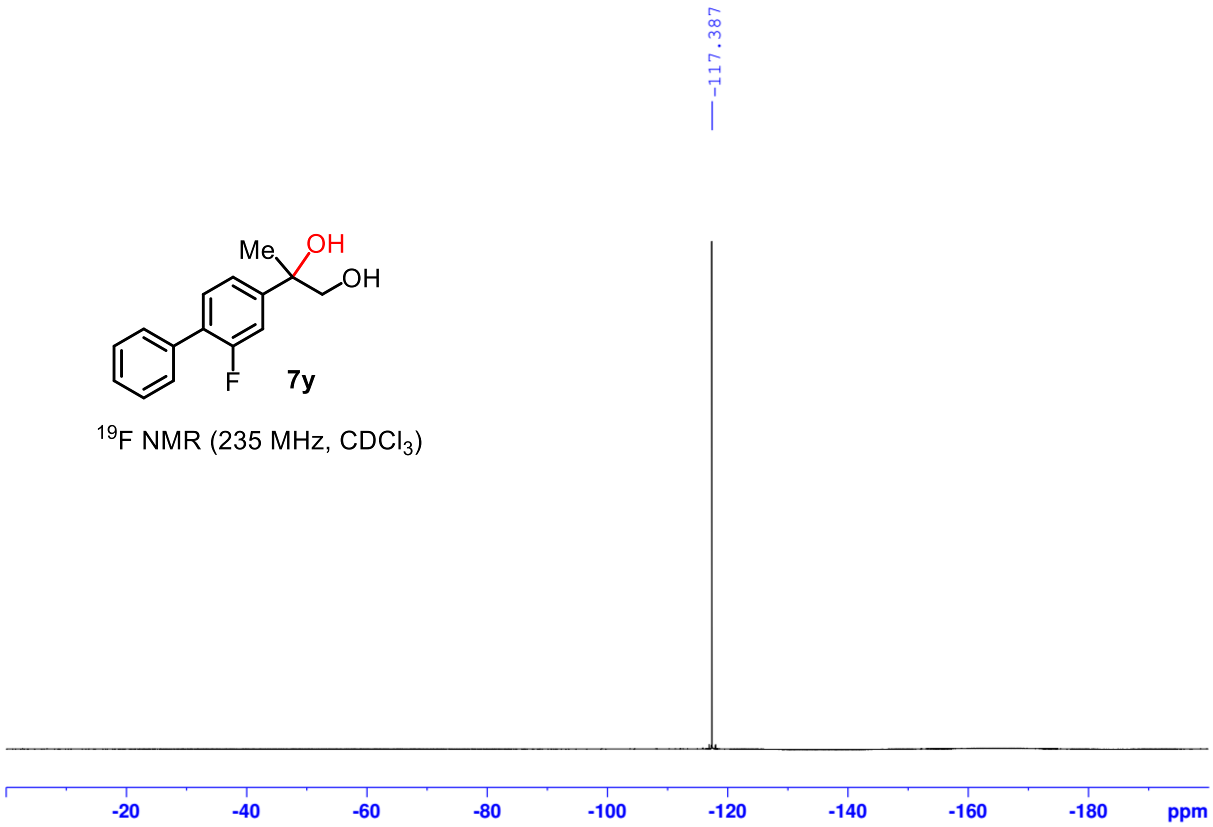
Me
OH
OH
F
7y
19F NMR (235 MHz, CDCl3)
-117.387
-20
-40
-60
-80
-100
-120
-140
-160
-180
ppm

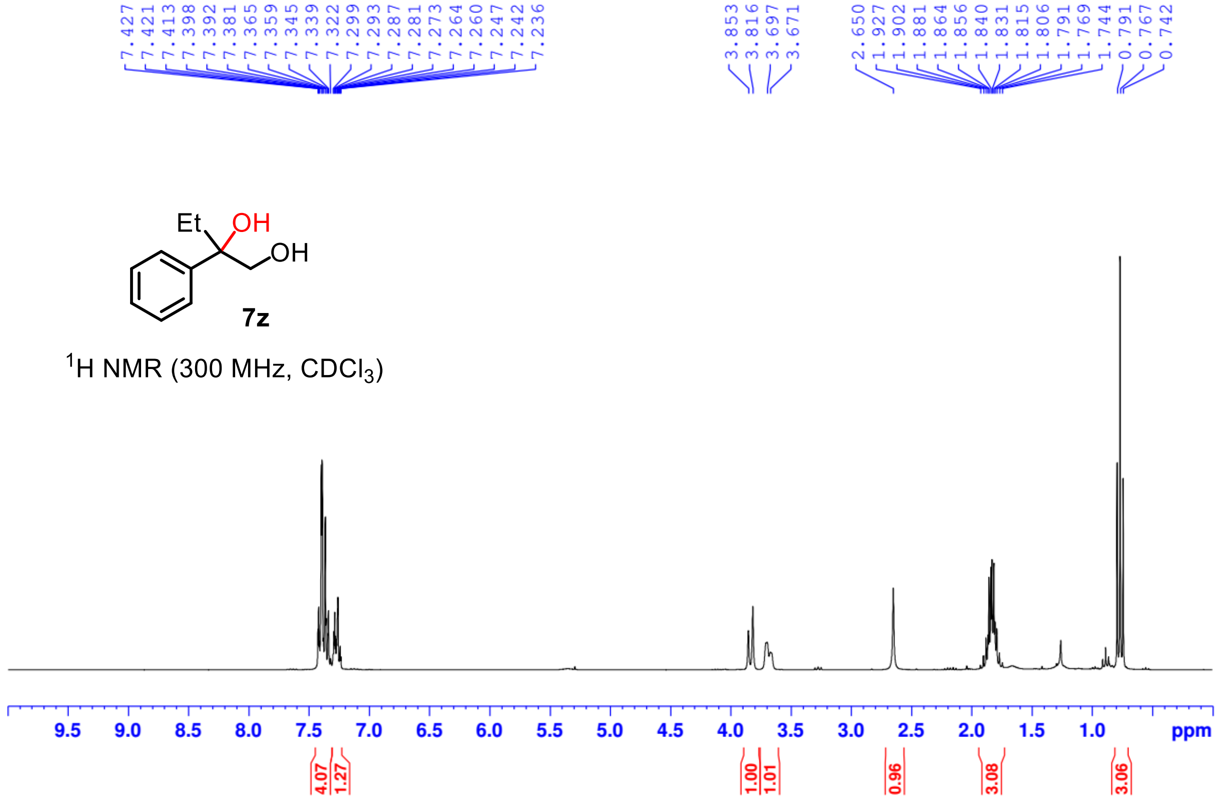
Et
OH
OH
7z
1H NMR (300 MHz, CDCl3)
7.427 7.421 7.413 7.398 7.392 7.381 7.365 7.359 7.345 7.339 7.322 7.299 7.293 7.287 7.281 7.273 7.264 7.260 7.247 7.242 7.236
3.853 3.816 3.697 3.671
2.650 1.927 1.902 1.881 1.864 1.856 1.840 1.831 1.815 1.806 1.791 1.769 1.744 0.791 0.767 0.742
9.5 9.0 8.5 8.0 7.5 7.0 6.5 6.0 5.5 5.0 4.5 4.0 3.5 3.0 2.5 2.0 1.5 1.0 ppm
4.07 1.27 1.00 1.01 0.96 3.08 3.06

7z

^{13}C NMR (75 MHz, $CDCl_3$)

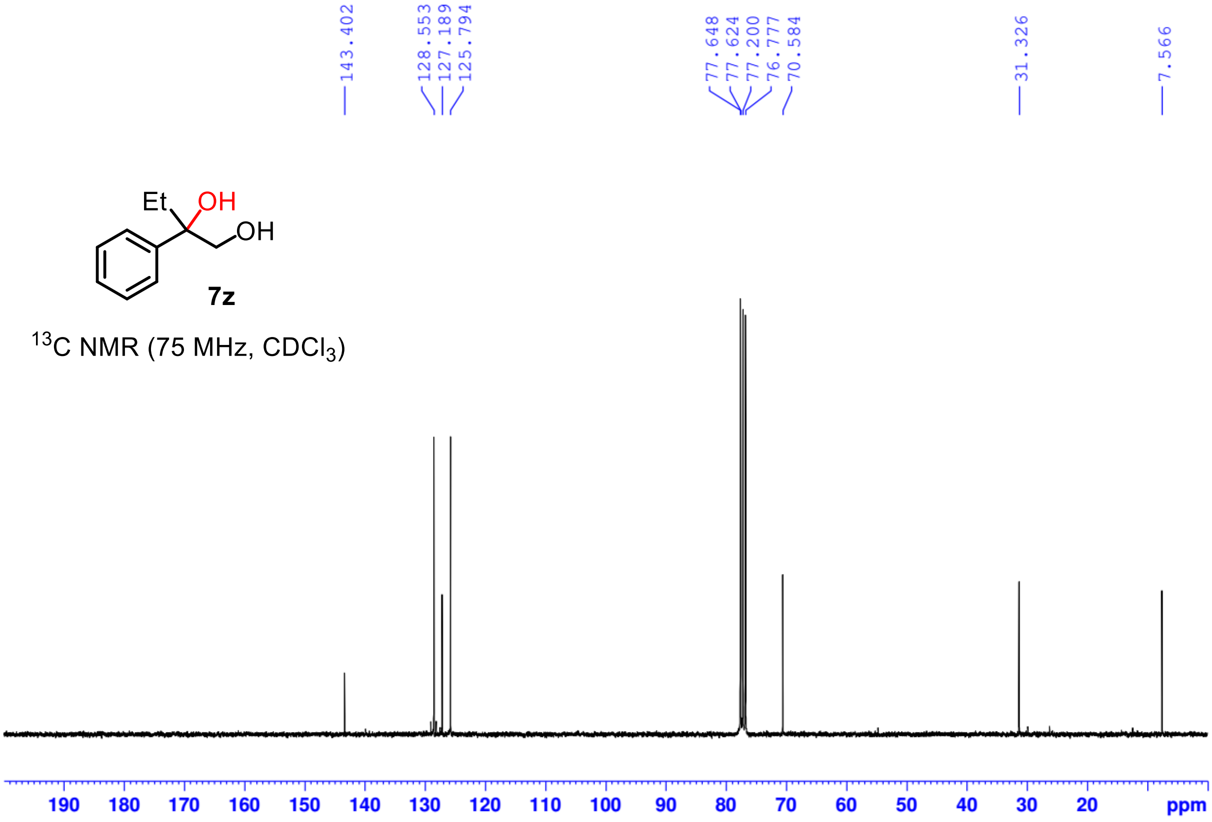

3za

^{1}H NMR (300 MHz, $CDCl_3$)

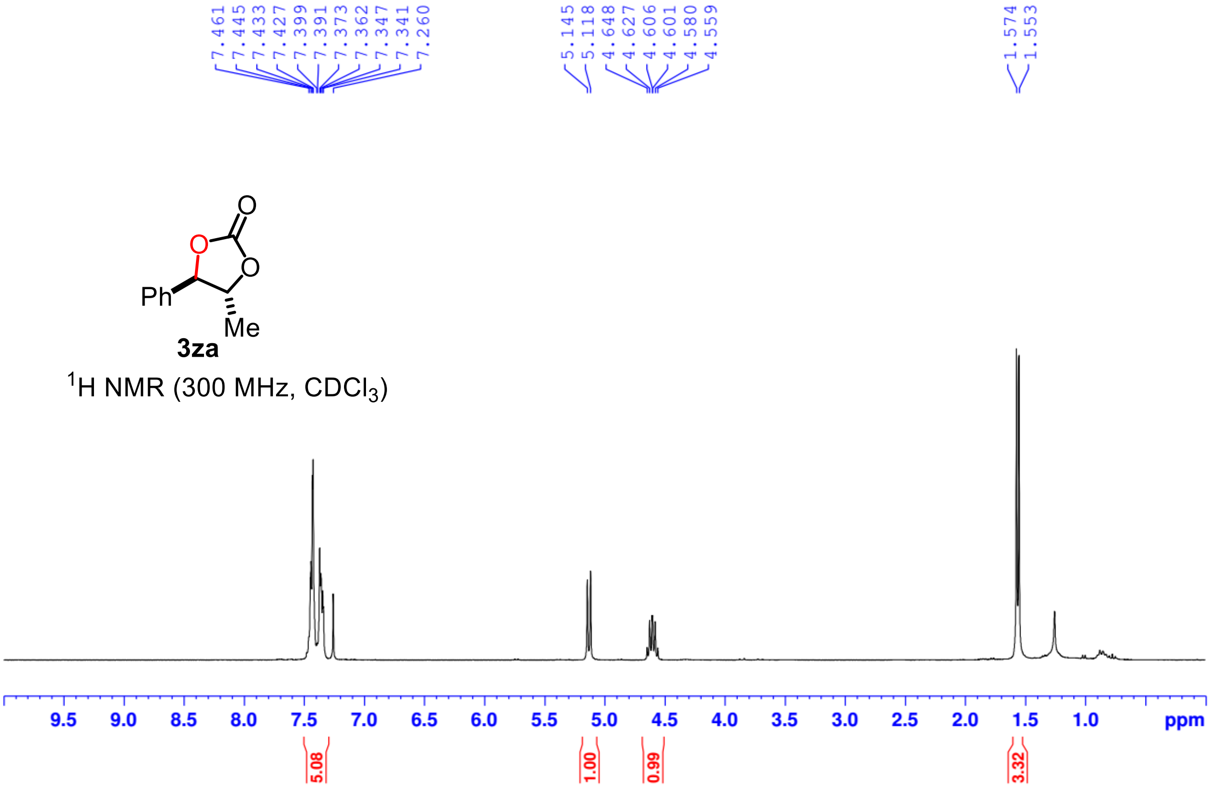

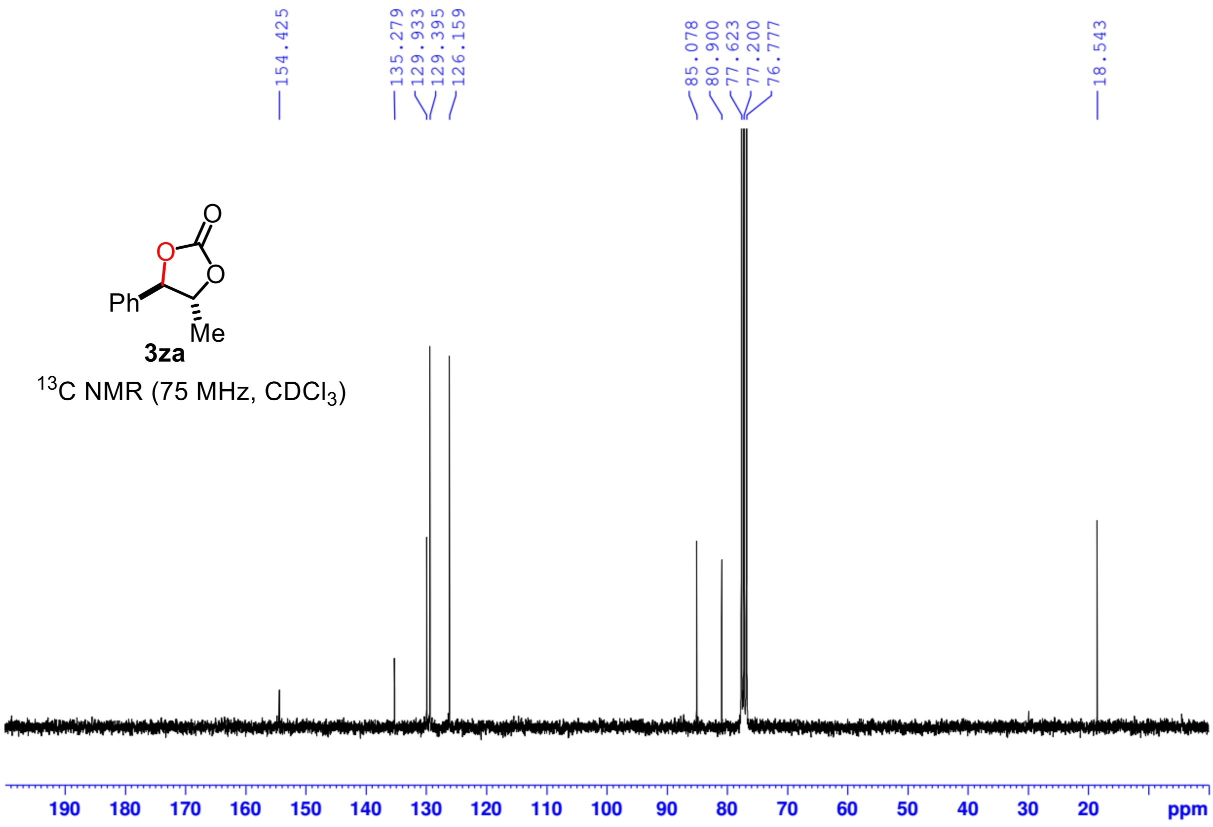

154.425
135.279
129.933
129.395
126.159
85.078
80.900
77.623
77.200
76.777
18.543
Ph
O
Me
3za
^{13}C NMR (75 MHz, CDCl$_3$)
190 180 170 160 150 140 130 120 110 100 90 80 70 60 50 40 30 20 ppm

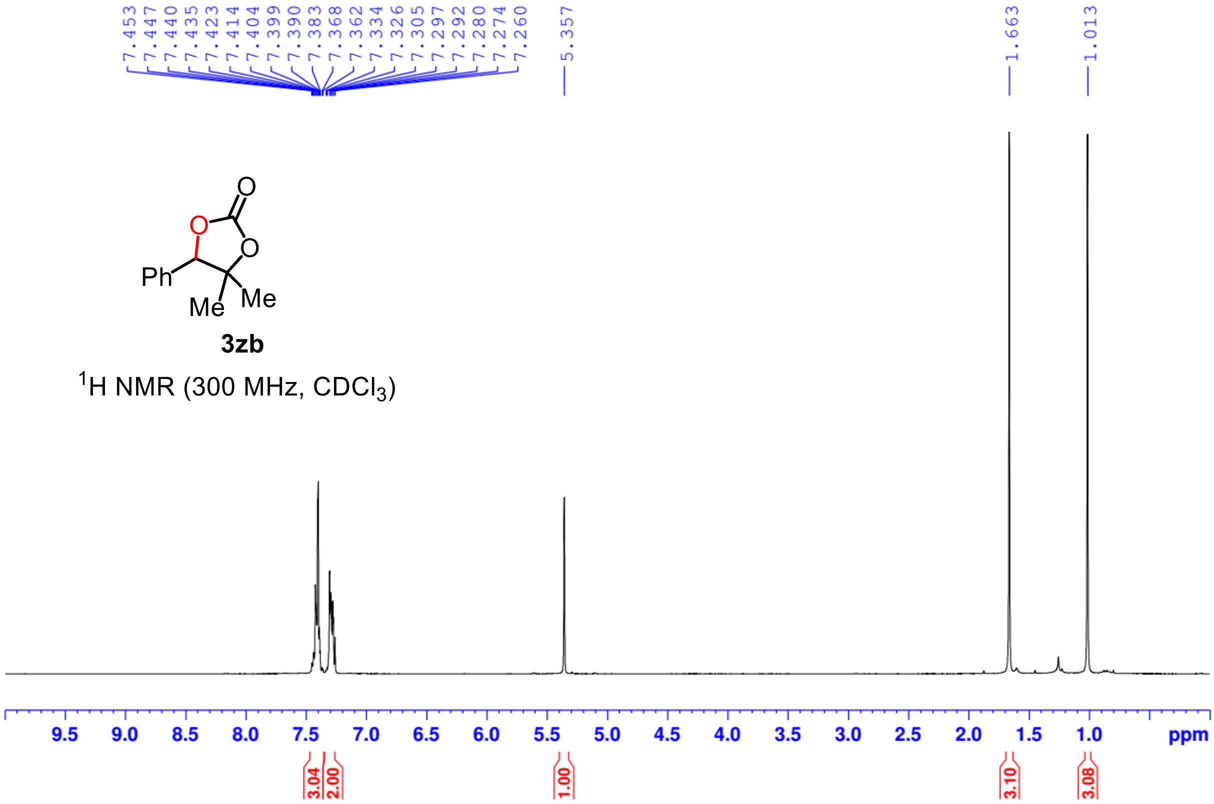

7.453
7.447
7.440
7.435
7.423
7.414
7.404
7.399
7.390
7.383
7.368
7.362
7.334
7.326
7.305
7.297
7.292
7.280
7.274
7.260
5.357
1.663
1.013
Ph
O
Me
Me
3zb
^{1}H NMR (300 MHz, CDCl$_3$)
9.5 9.0 8.5 8.0 7.5 7.0 6.5 6.0 5.5 5.0 4.5 4.0 3.5 3.0 2.5 2.0 1.5 1.0 ppm
3.04
2.00
1.00
3.10
3.08

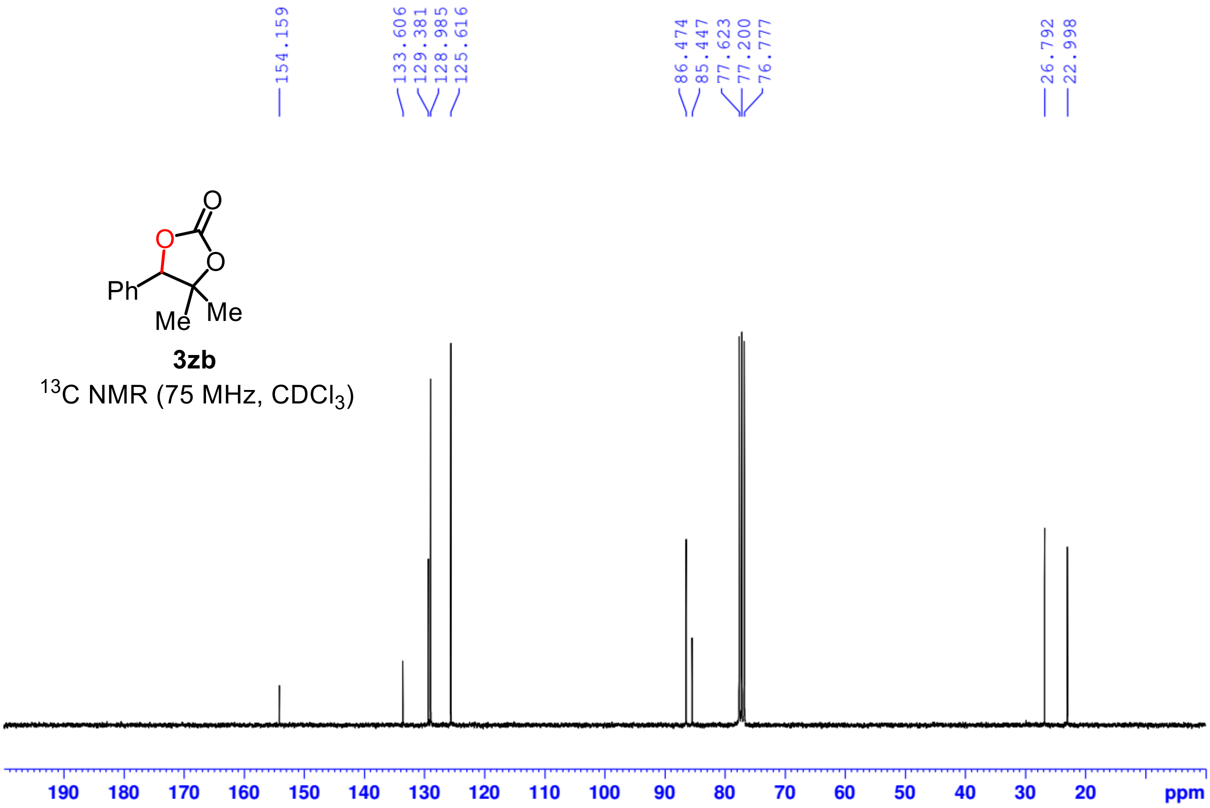

3zb

^{13}C NMR (75 MHz, $CDCl_3$)

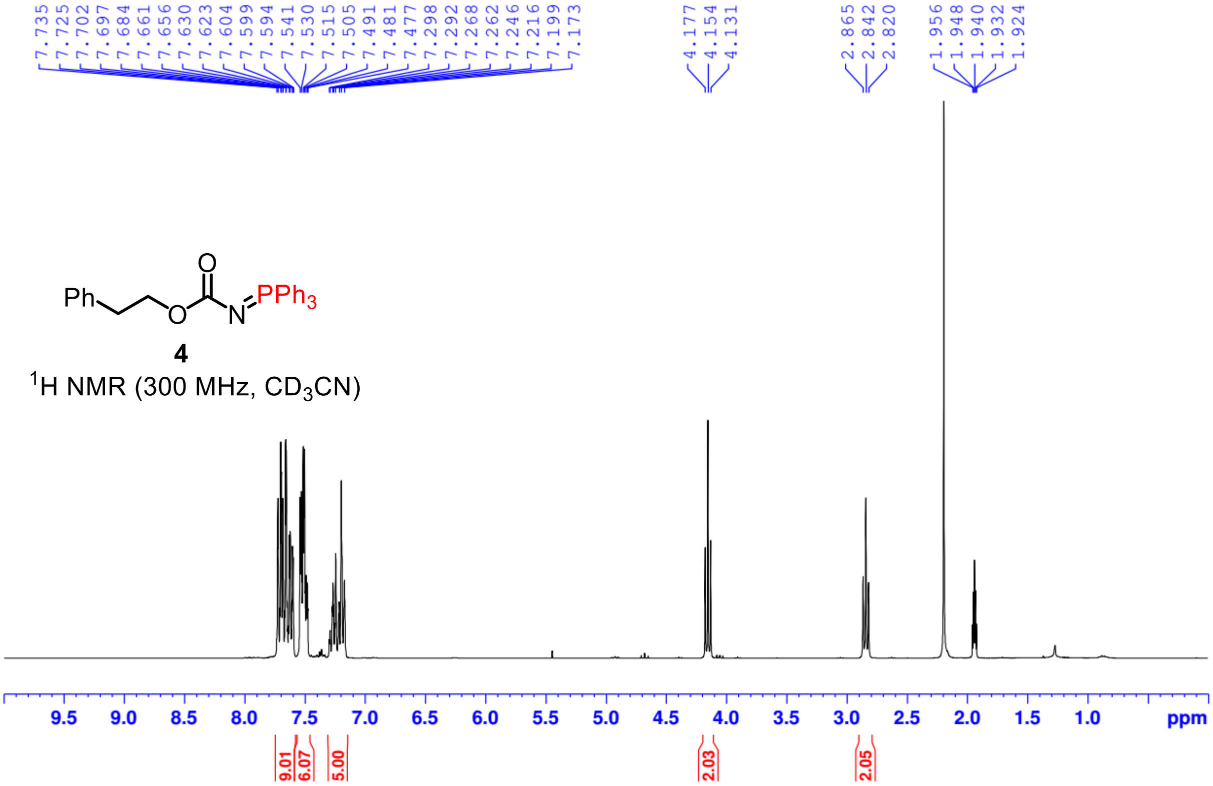

4

^{1}H NMR (300 MHz, CD_3CN)

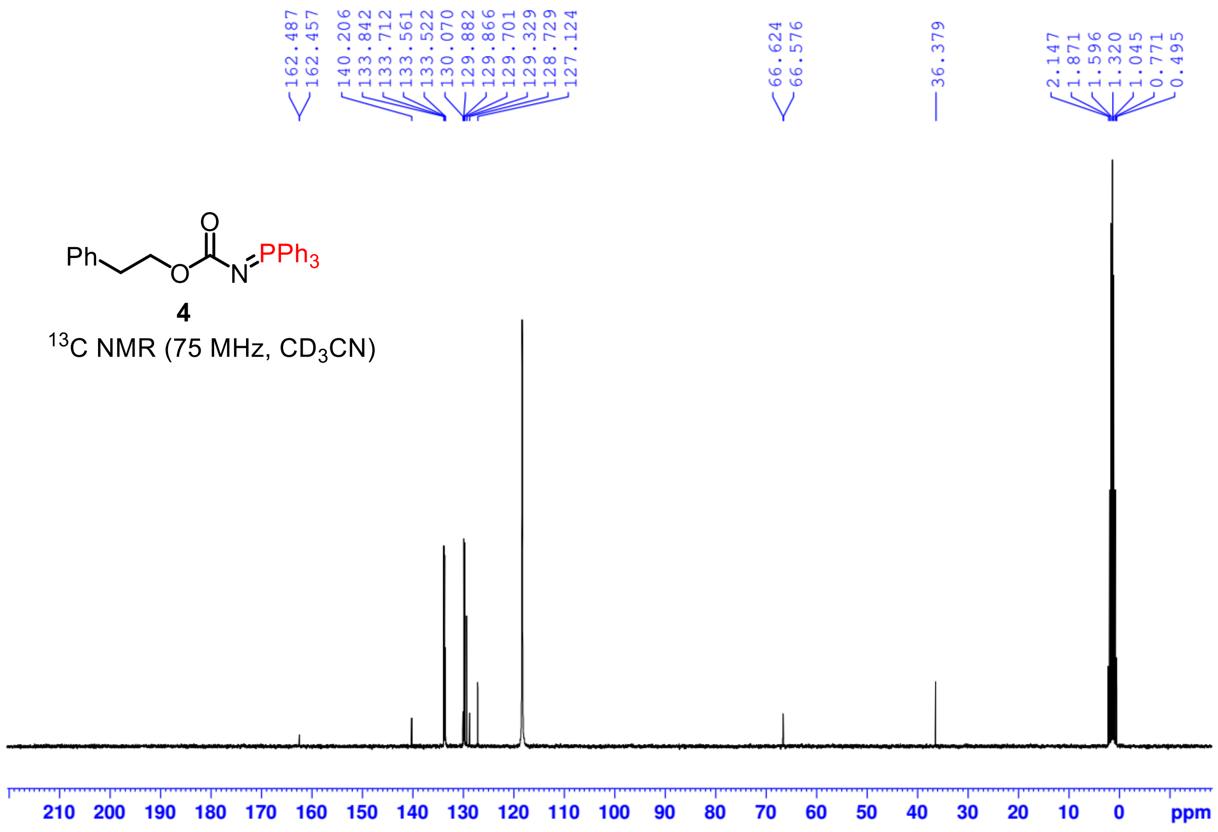

4

^{13}C NMR (75 MHz, CD_3CN)

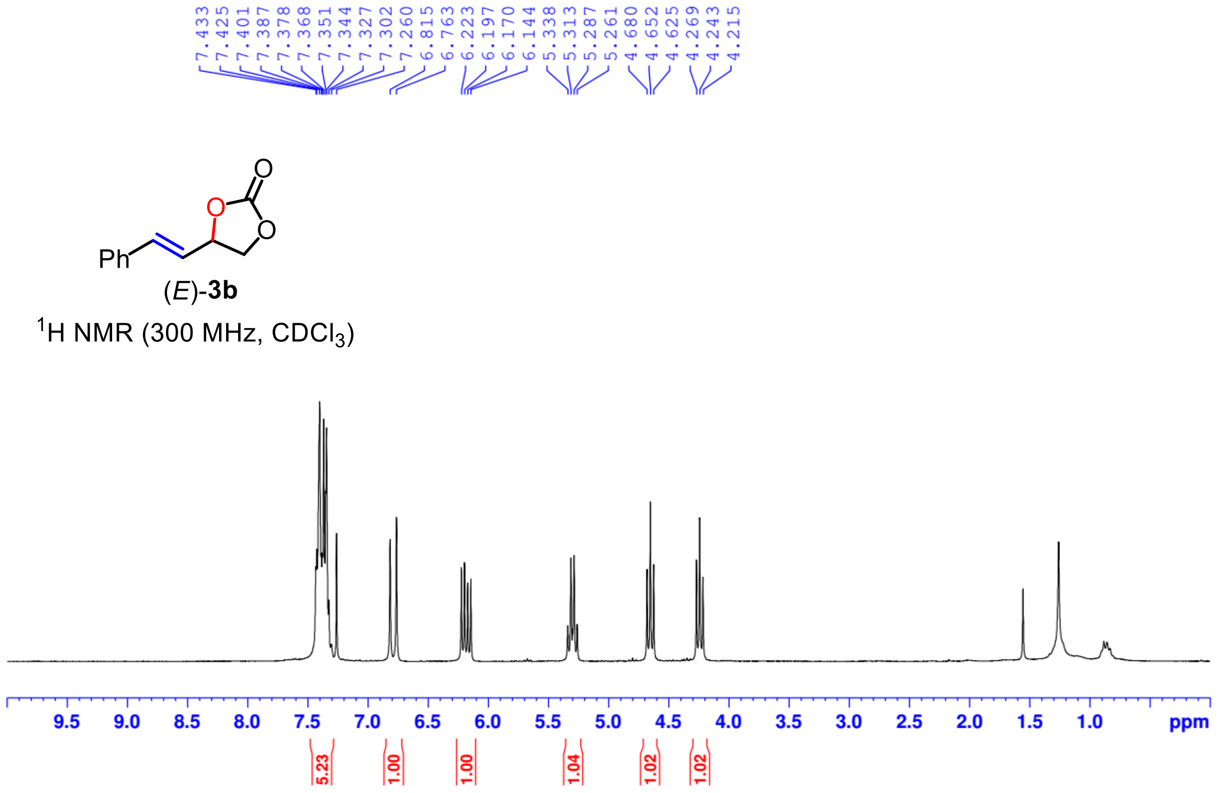

(*E*)-**3b**

^{1}H NMR (300 MHz, $CDCl_3$)

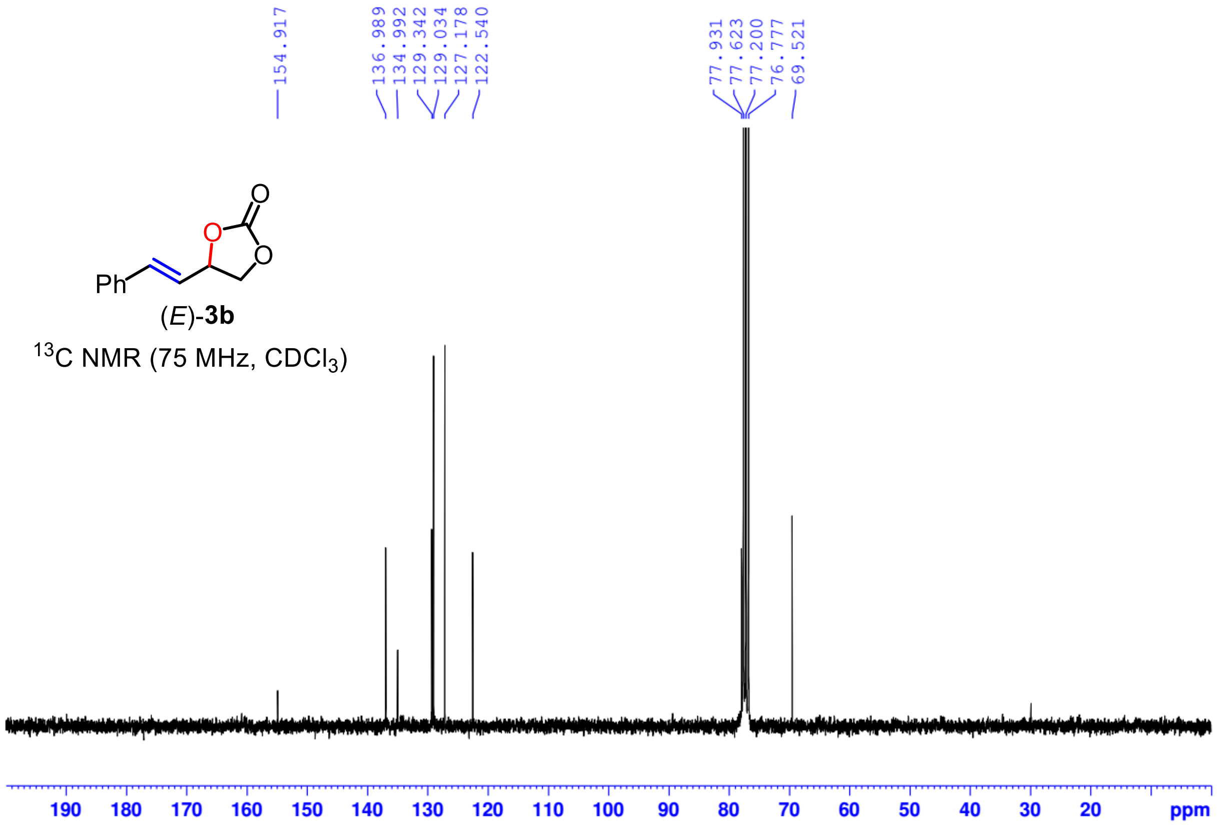
154.917
136.989
134.992
129.342
129.034
127.178
122.540
77.931
77.623
77.200
76.777
69.521
Ph
O
O
O
(E)-3b
^{13}C NMR (75 MHz, CDCl$_3$)
190 180 170 160 150 140 130 120 110 100 90 80 70 60 50 40 30 20 ppm

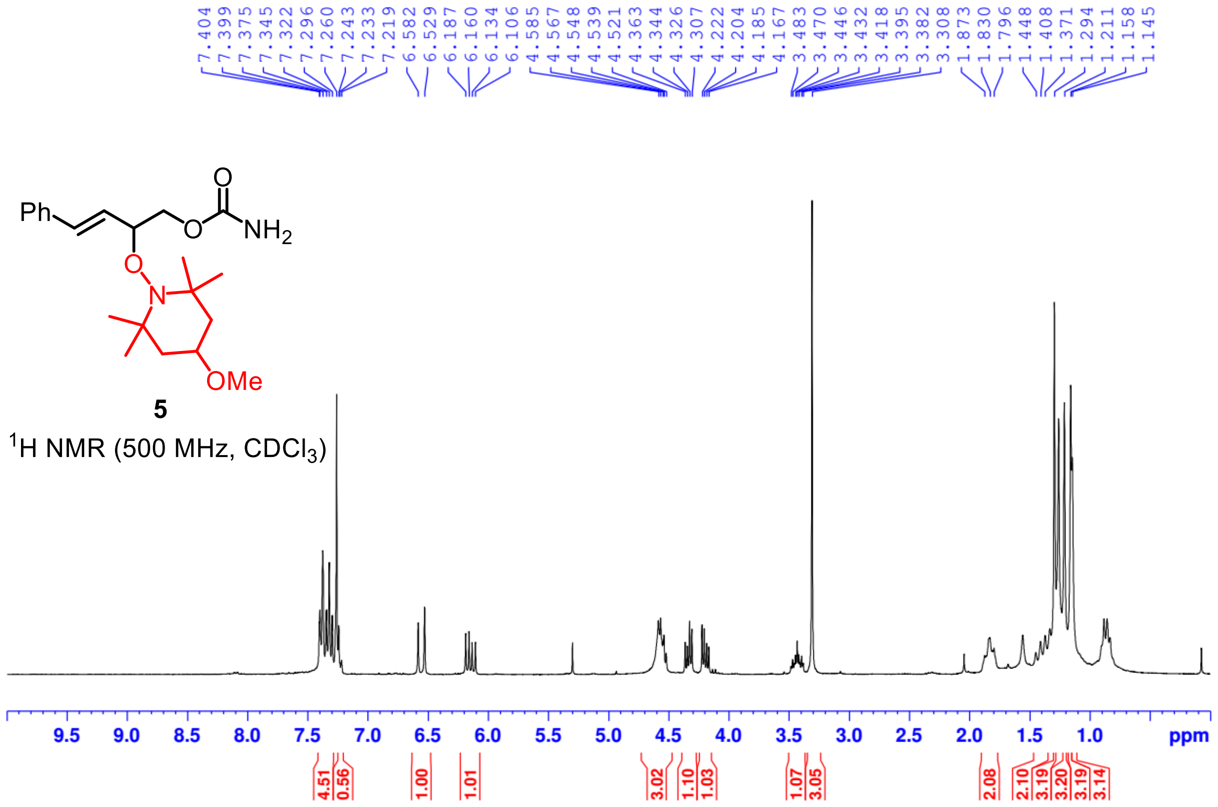
7.404
7.399
7.375
7.345
7.322
7.296
7.260
7.243
7.233
7.219
6.582
6.529
6.187
6.160
6.134
6.106
4.585
4.567
4.548
4.539
4.521
4.363
4.344
4.326
4.307
4.222
4.204
4.185
4.167
3.483
3.470
3.446
3.432
3.418
3.395
3.382
3.308
1.873
1.830
1.796
1.448
1.408
1.371
1.294
1.211
1.158
1.145
Ph
O
NH$_2$
N
OMe
5
^{1}H NMR (500 MHz, CDCl$_3$)
9.5 9.0 8.5 8.0 7.5 7.0 6.5 6.0 5.5 5.0 4.5 4.0 3.5 3.0 2.5 2.0 1.5 1.0 ppm
4.51
0.56
1.00
1.01
3.02
1.10
1.03
1.07
3.05
2.08
2.10
3.19
3.20
3.19
3.14

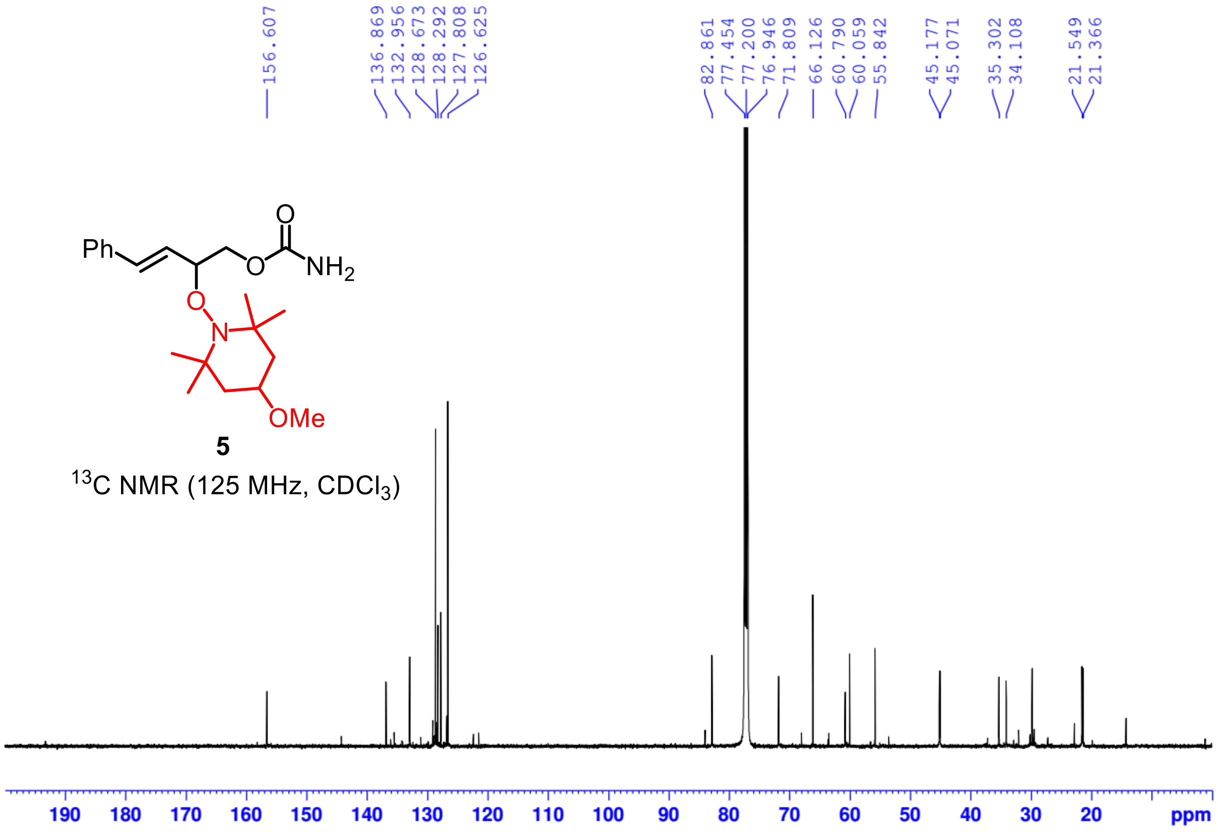
156.607
136.869 132.956 128.673 128.292 127.808 126.625
82.861 77.454 77.200 76.946 71.809 66.126 60.790 60.059 55.842
45.177 45.071 35.302 34.108 21.549 21.366
Ph
O
NH2
O
N
OMe
5
13C NMR (125 MHz, CDCl3)
190 180 170 160 150 140 130 120 110 100 90 80 70 60 50 40 30 20 ppm

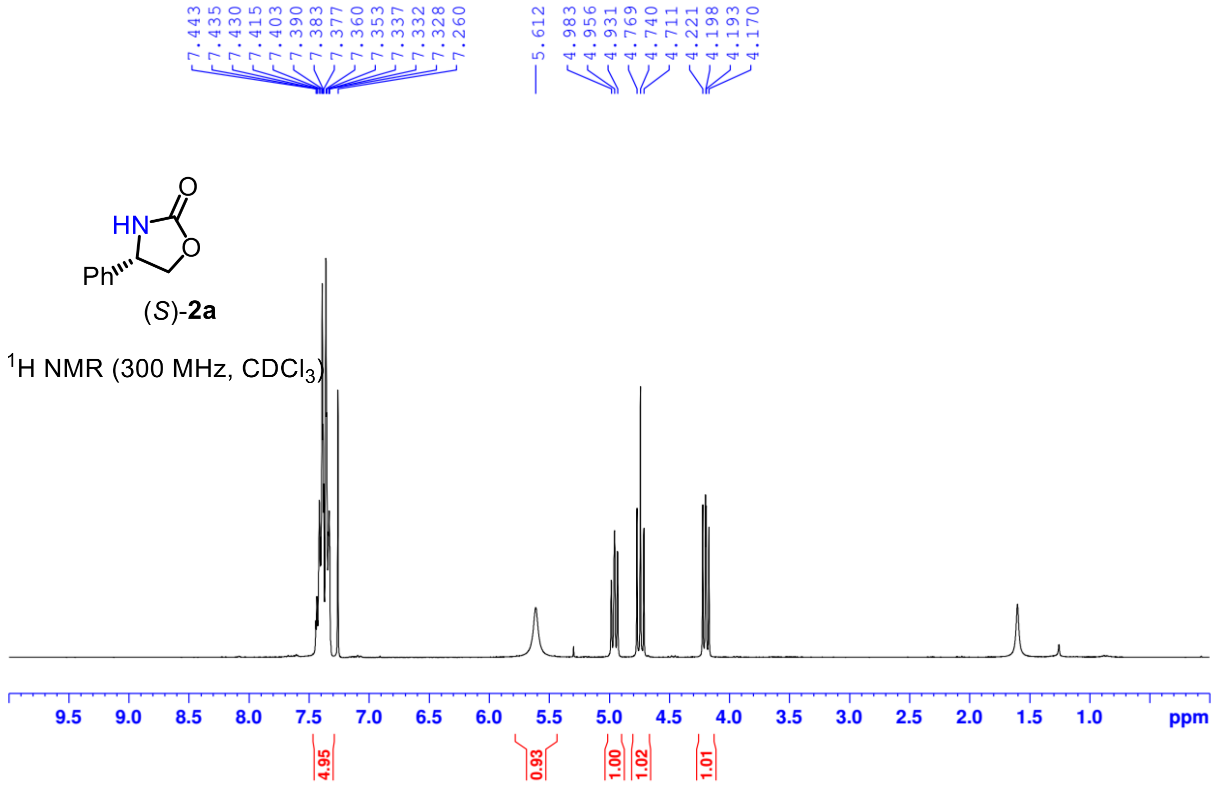
7.443 7.435 7.430 7.415 7.403 7.390 7.383 7.377 7.360 7.353 7.337 7.332 7.328 7.260
5.612
4.983 4.956 4.931 4.769 4.740 4.711 4.221 4.198 4.193 4.170
HN
O
Ph
(S)-2a
1H NMR (300 MHz, CDCl3)
9.5 9.0 8.5 8.0 7.5 7.0 6.5 6.0 5.5 5.0 4.5 4.0 3.5 3.0 2.5 2.0 1.5 1.0 ppm
4.95 0.93 1.00 1.02 1.01

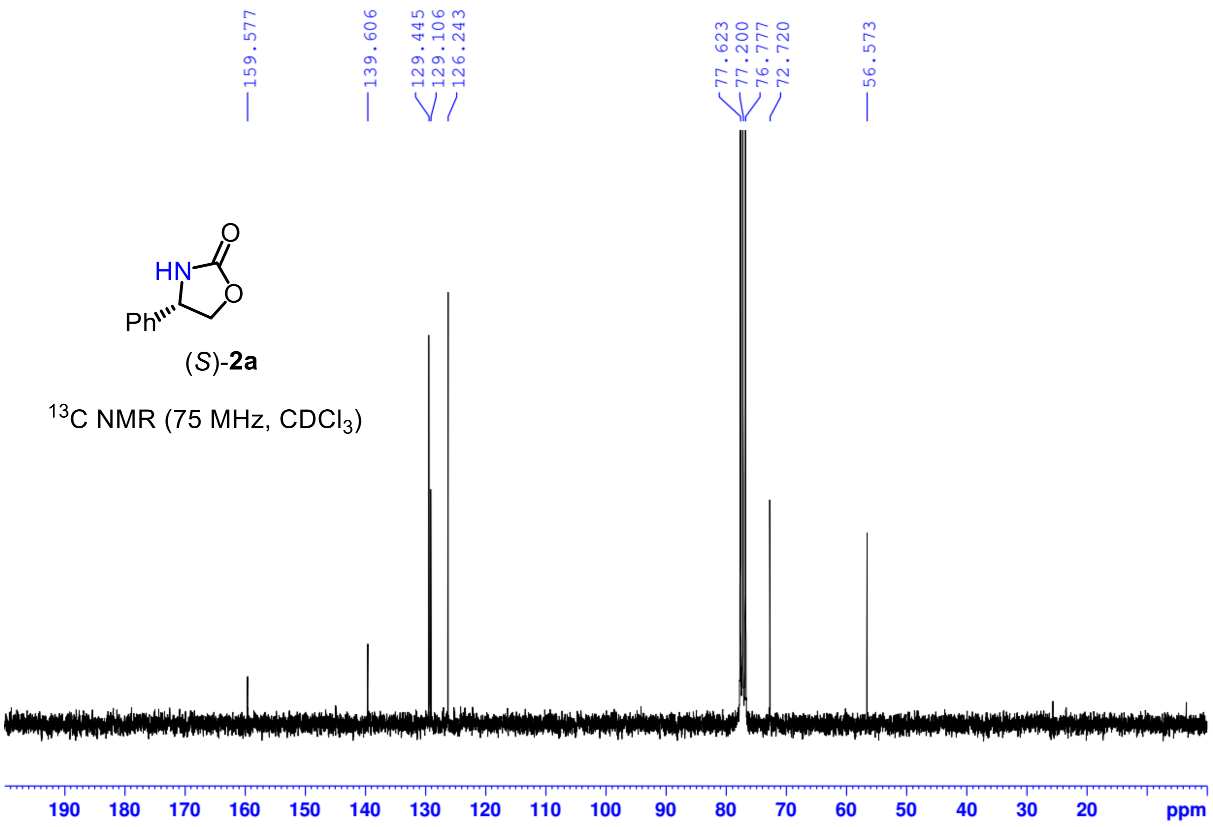
159.577
139.606
129.445
129.106
126.243
77.623
77.200
76.777
72.720
56.573
HN
O
O
Ph
(S)-2a
13C NMR (75 MHz, CDCl3)
190 180 170 160 150 140 130 120 110 100 90 80 70 60 50 40 30 20 ppm

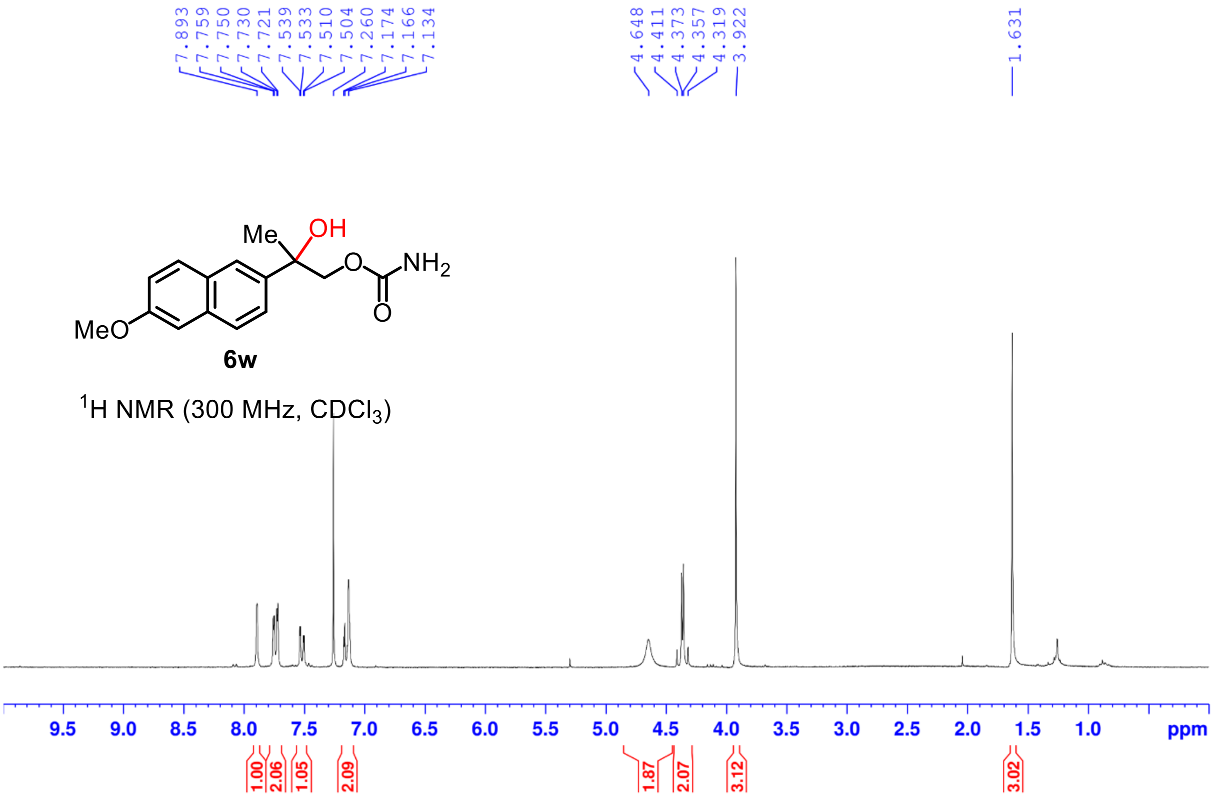
7.893
7.759
7.750
7.730
7.721
7.539
7.533
7.510
7.504
7.260
7.174
7.166
7.134
4.648
4.411
4.373
4.357
4.319
3.922
1.631
Me
OH
O
NH2
O
MeO
6w
1H NMR (300 MHz, CDCl3)
9.5 9.0 8.5 8.0 7.5 7.0 6.5 6.0 5.5 5.0 4.5 4.0 3.5 3.0 2.5 2.0 1.5 1.0 ppm
1.00
2.06
1.05
2.09
1.87
2.07
3.12
3.02

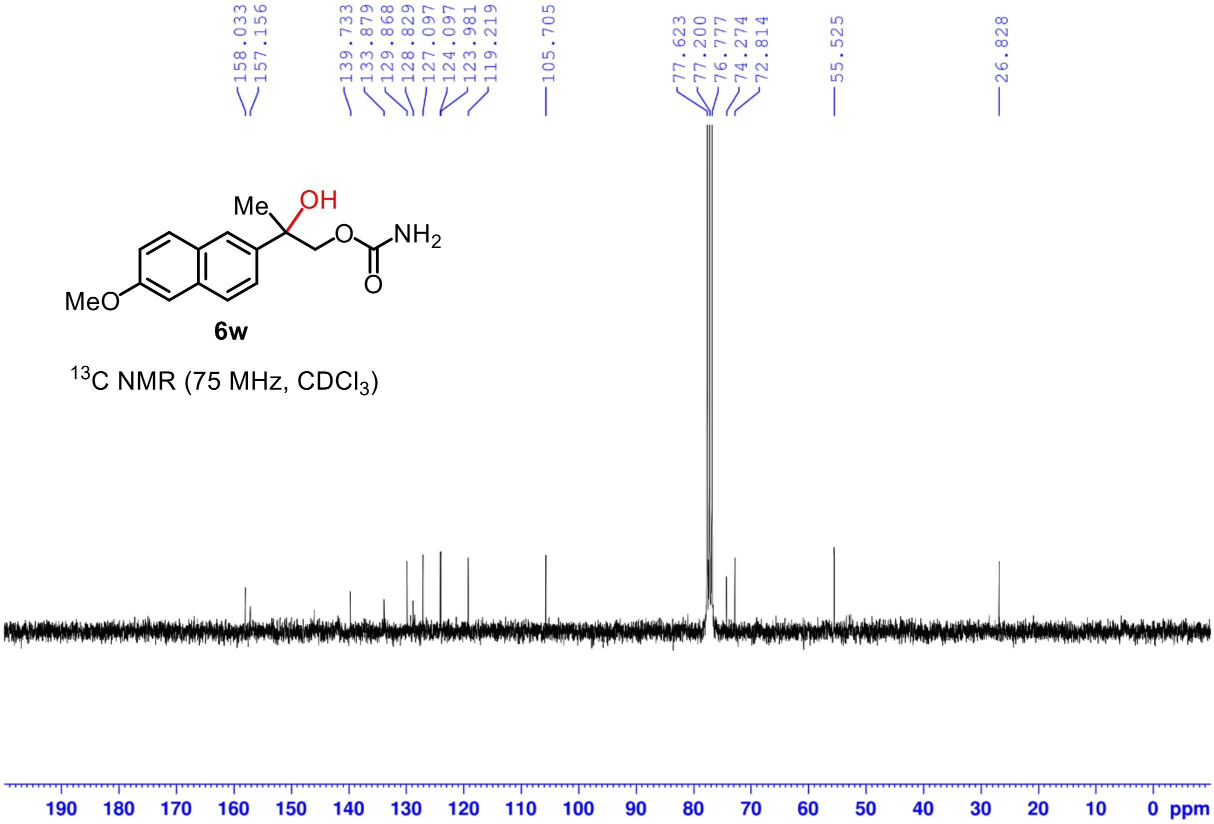

6w

^{13}C NMR (75 MHz, $CDCl_3$)

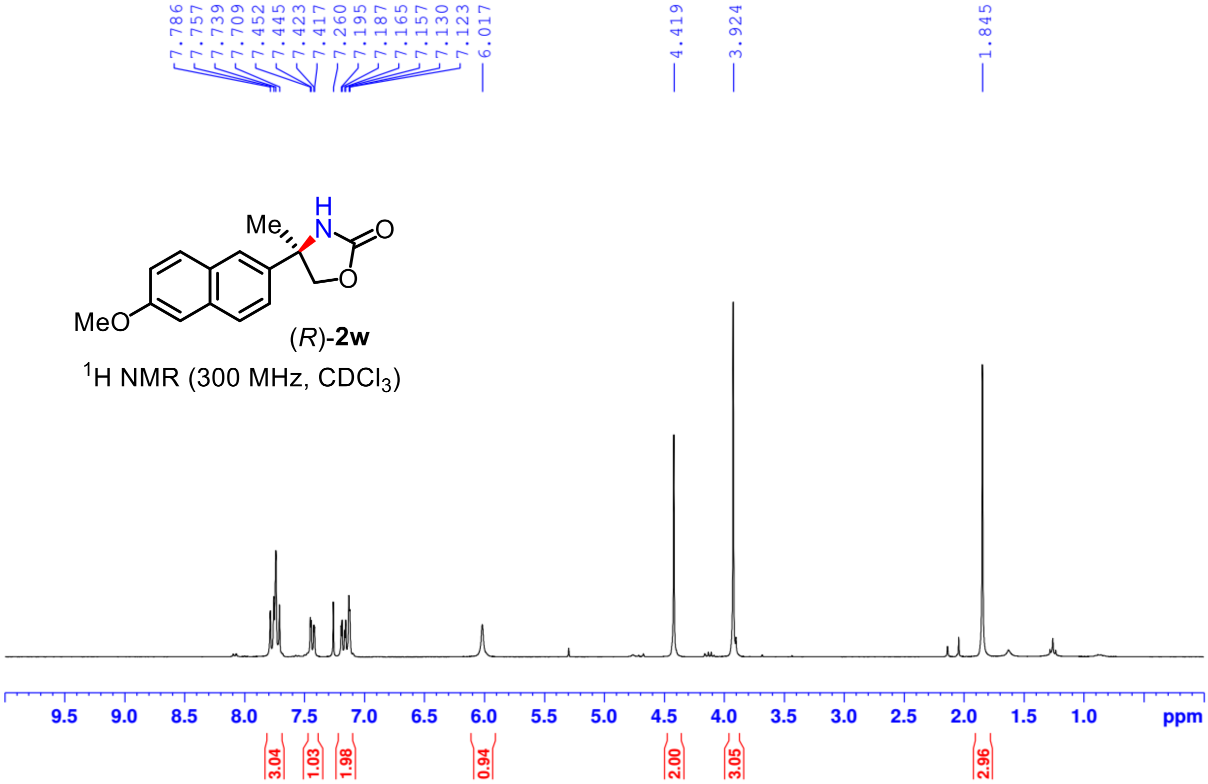

(*R*)-**2w**

^{1}H NMR (300 MHz, $CDCl_3$)

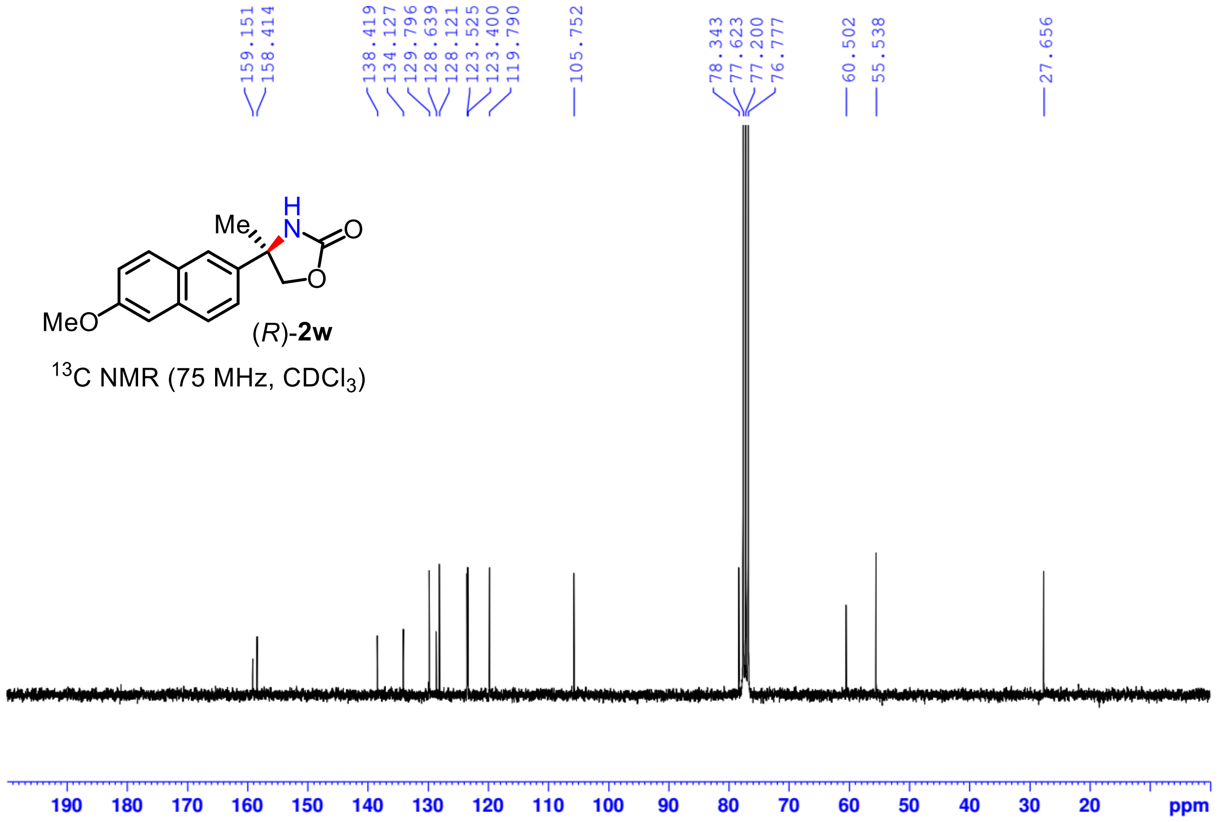
159.151
158.414
138.419
134.127
129.796
128.639
128.121
123.525
123.400
119.790
105.752
78.343
77.623
77.200
76.777
60.502
55.538
27.656
Me
H
N
O
O
MeO
(R)-2w
^{13}C NMR (75 MHz, $CDCl_3$)
190 180 170 160 150 140 130 120 110 100 90 80 70 60 50 40 30 20 ppm

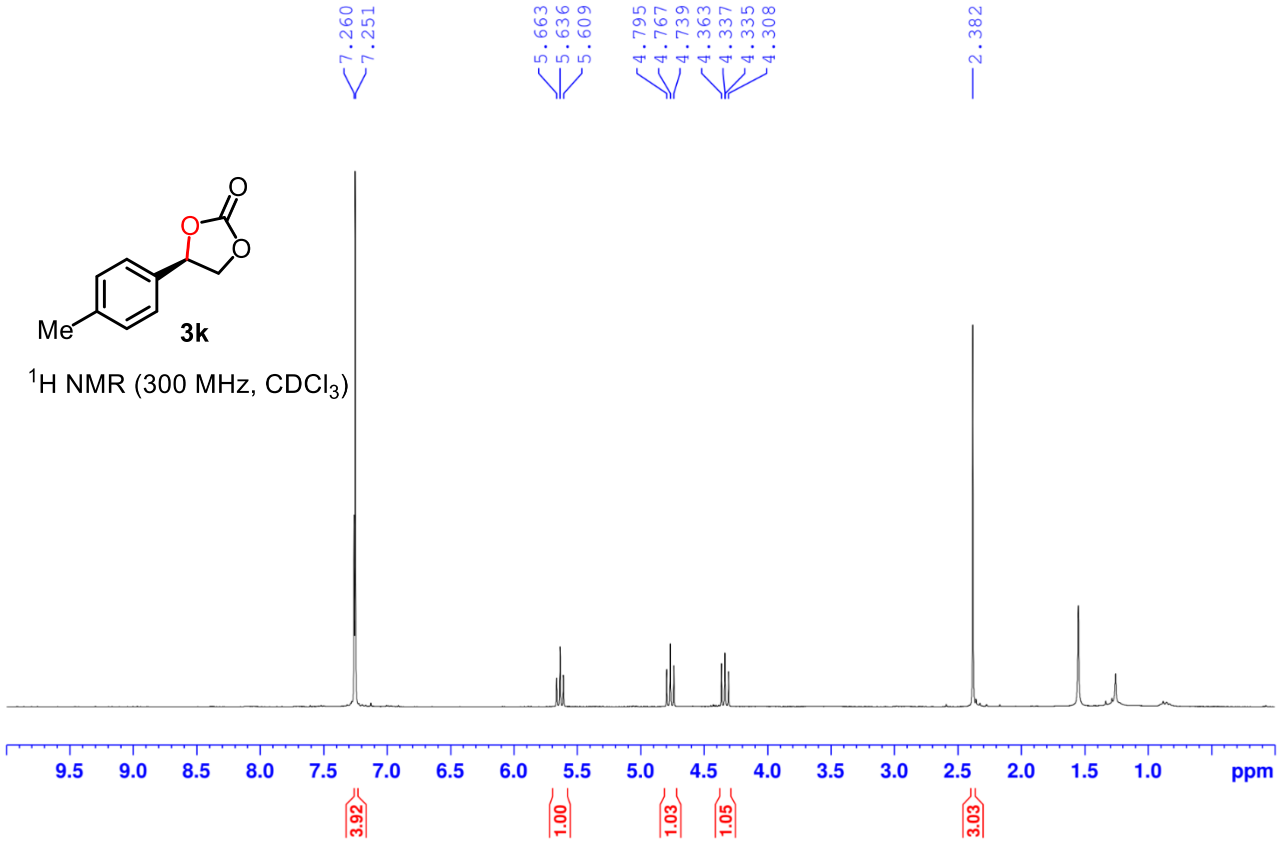
7.260
7.251
5.663
5.636
5.609
4.795
4.767
4.739
4.363
4.337
4.335
4.308
2.382
O
O
O
Me
3k
^{1}H NMR (300 MHz, $CDCl_3$)
9.5 9.0 8.5 8.0 7.5 7.0 6.5 6.0 5.5 5.0 4.5 4.0 3.5 3.0 2.5 2.0 1.5 1.0 ppm
3.92
1.00
1.03
1.05
3.03

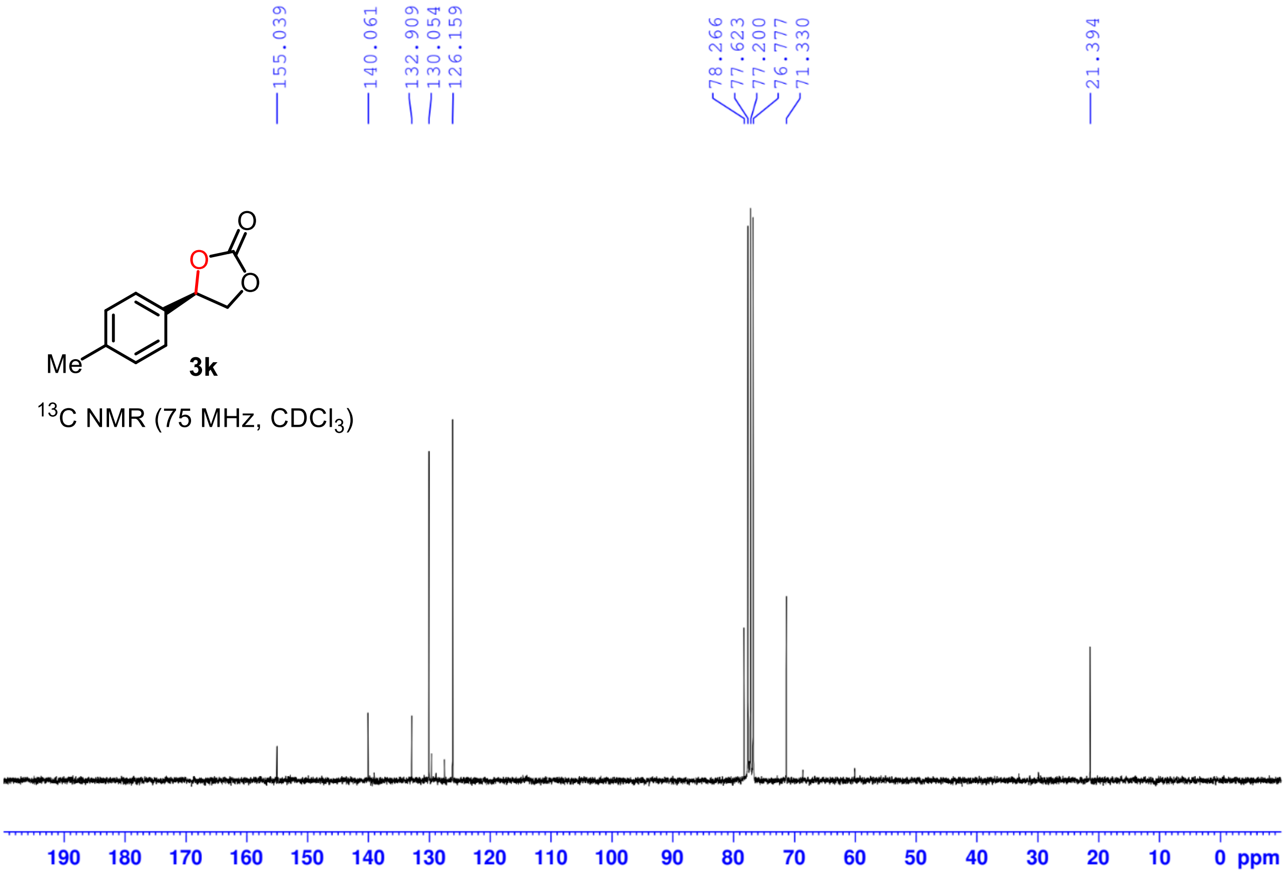
155.039
140.061
132.909
130.054
126.159
78.266
77.623
77.200
76.777
71.330
21.394
Me
3k
^{13}C NMR (75 MHz, $CDCl_3$)
190 180 170 160 150 140 130 120 110 100 90 80 70 60 50 40 30 20 10 0 ppm

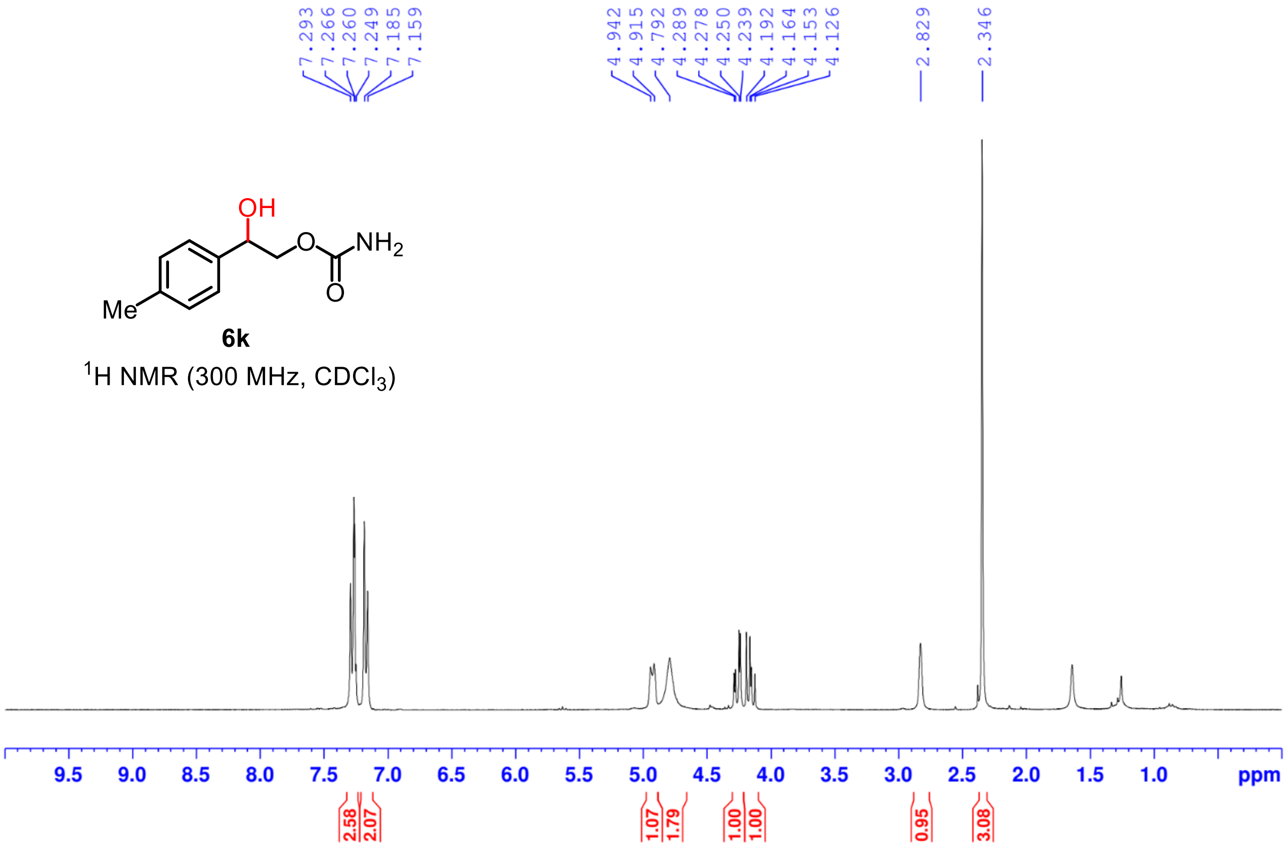
7.293
7.266
7.260
7.249
7.185
7.159
4.942
4.915
4.792
4.289
4.278
4.250
4.239
4.192
4.164
4.153
4.126
2.829
2.346
OH
NH_2
Me
6k
^{1}H NMR (300 MHz, $CDCl_3$)
9.5 9.0 8.5 8.0 7.5 7.0 6.5 6.0 5.5 5.0 4.5 4.0 3.5 3.0 2.5 2.0 1.5 1.0 ppm
2.58
2.07
1.07
1.79
1.00
1.00
0.95
3.08

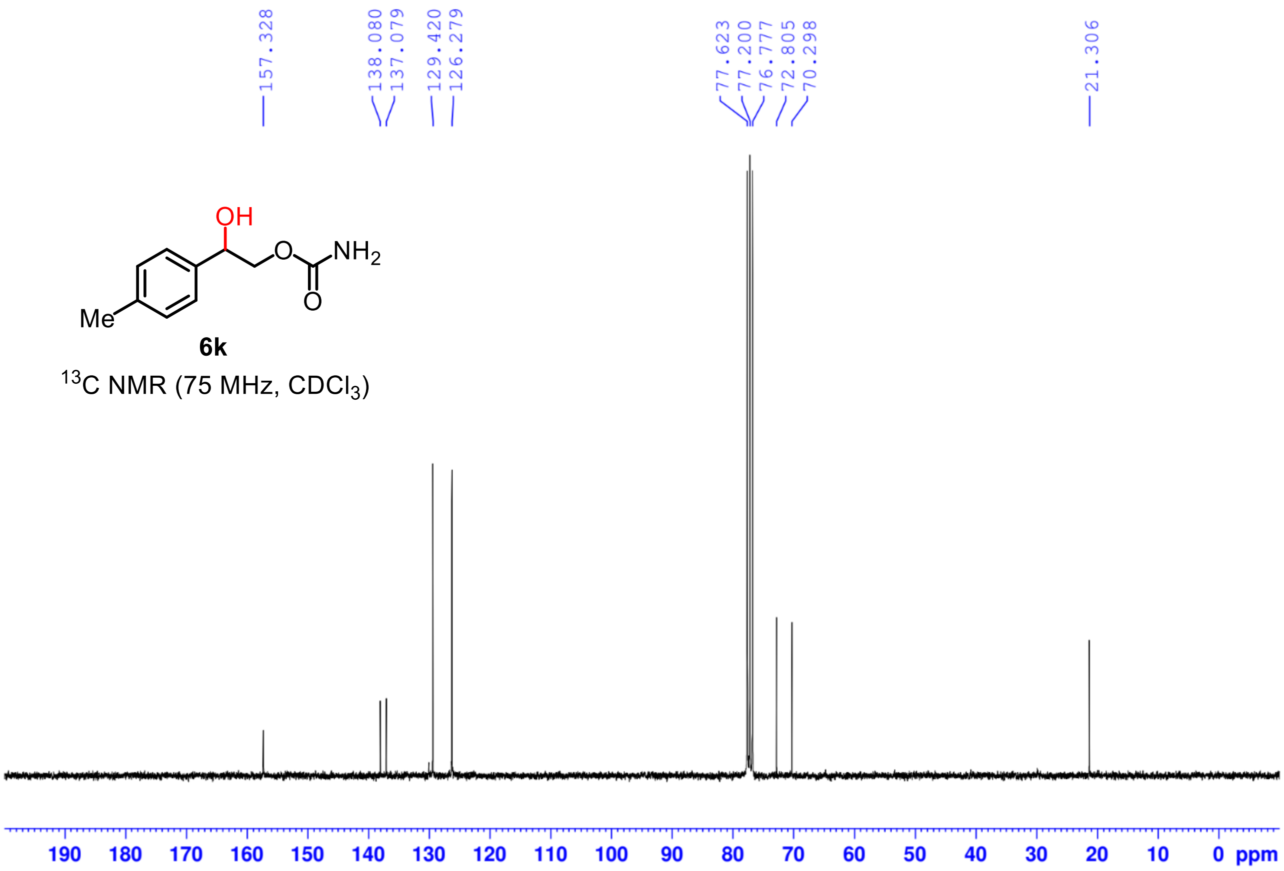
157.328
138.080
137.079
129.420
126.279
77.623
77.200
76.777
72.805
70.298
21.306
OH
O
NH2
O
Me
6k
13C NMR (75 MHz, CDCl3)
190 180 170 160 150 140 130 120 110 100 90 80 70 60 50 40 30 20 10 0 ppm

10. Computational Study

10.1 Computational Results

Density functional theory (DFT) calculations provide additional support for the proposed radical mechanism. For ruthenium intermediate **Ia** (intermediate **I** in which one MeCN ligand is replaced by a benzoate), the triplet state **^{3}Ia** is favored by 2.8 kcal/mol over the singlet state **^{1}Ia** (Figure S14a). With respect to the C-H amination step, the triplet 1,5-HAT transition state **^{3}TS-CHA** ($\Delta G^{\ddagger}$ = 12.7 kcal/mol) was also found to be 2.0 kcal/mol lower in energy than the singlet C–H amination transition state **^{1}TS-CHA** ($\Delta G^{\ddagger}$ = 14.7 kcal/mol). The carbamate moiety, with its two oxygen atoms, appeared necessary to ensure the dominance of the triplet radical pathway, as our previous DFT study on an analogous system with a simple amide substrate favored the concerted singlet C–H amination pathway.[30]

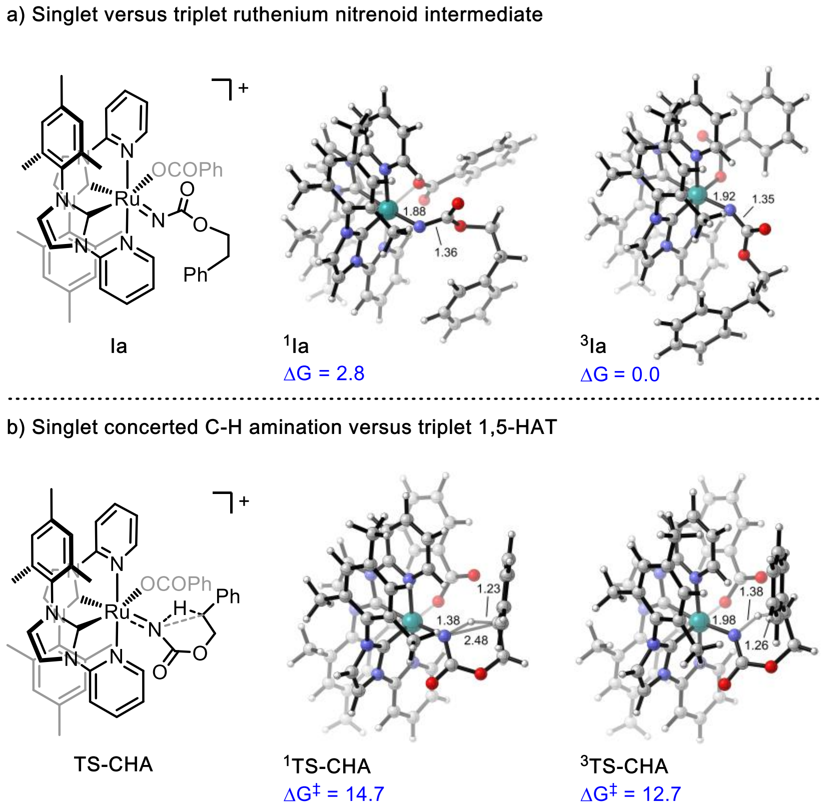

Figure S14. Computational results. Calculated structures of ruthenium nitrenoid intermediate **Ia** and N–H forming transition state **TS-CHA** at the M06-D3/6-311++G(d,p)–SDD (Ru), SMD ($CHCl_3$)//B3LYP-D3/6-31G(d)–LANL2DZ (Ru) level of theory. Interatomic distances are in ångströms. Energies are in kcal/mol.

10.2 Computational Methods

Density functional theory (DFT) computations were performed in Gaussian 16, Revision C.01.[31] Molecular geometries were optimized using the B3LYP[32] functional augmented with Grimme's D3[33] empirical dispersion term. The LANL2DZ basis set (including effective core potential)[34] was used for Ru, and the 6-31G(d) basis set was used for all other atoms. Frequency calculations were performed at the same level of theory as that used for geometry optimization to characterize the stationary points as either minima (no imaginary frequencies) or first-order saddle points (one imaginary frequency) on the potential energy surface. Intrinsic Reaction Coordinate (IRC) calculations were performed to ensure that the first-order saddle points found were real transition states connecting the reactants and the products. Thermal contributions to free energies were calculated from vibrational frequencies using the quasi-rigid rotor-harmonic oscillator (RRHO) approach of Grimme.[35] Single point energies were calculated with the M06[36] functional augmented with Grimme's D3 empirical dispersion term, with the SDD[37] basis set for Pd and the 6-311++G(d,p) basis set for all other atoms. Solvation effects were incorporated using the SMD[38] model with chloroform as the solvent. Molecular structure visualizations were obtained using CYLview.[39] Monte Carlo conformational searches were performed with the Merck molecular force field (MMFF) implemented in Spartan'18[40] to ensure that the lowest energy conformations are presented. Benchmarking calculations are performed with the ωB97X-D[41] functional, with the SDD basis set for Pd and the 6-311++G(d,p) basis set for all other atoms.

10.3 Calculated Energies with Different DFT Methods

We performed benchmarking calculations using the ωB97X-D functional, as computed values of singlet-triplet energy gaps for metal nitrenoids are known to be functional-dependent.[42] In agreement with experimental observations, both functionals indicated the triplet state to be favored for **Ia** and **TS-CHA**.

(a) Calculated relative energies of **Ia** and **TS-CHA** at the M06-D3/6-311++G(d,p)–SDD (Ru), SMD ($CHCl_3$)//B3LYP-D3/6-31G(d)–LANL2DZ (Ru) level of theory. Energy values are in Hartrees unless otherwise noted.

Structure	E($CHCl_3$)	ΔG	G($CHCl_3$) [= E($CHCl_3$) + ΔG]	G_{S-T} (Singlet-Triplet Energy Gap) (kcal/mol)
^{1}**Ia**	-2712.124821	0.796958	-2711.327863	2.8
^{3}**Ia**	-2712.130243	0.797983	-2711.332260	
^{1}**TS-CHA**	-2712.106742	0.797864	-2711.308878	2.0
^{3}**TS-CHA**	-2712.107385	0.795352	-2711.312033	

(b) Calculated relative energies of **Ia** and **TS-CHA** at the ωB97X-D/6-311++G(d,p)–SDD (Ru), SMD ($CHCl_3$)//B3LYP-D3/6-31G(d)–LANL2DZ (Ru) level of theory. Energy values are in Hartrees unless otherwise noted.

Structure	E($CHCl_3$)	ΔG	G($CHCl_3$) [= E($CHCl_3$) + ΔG]	G_{S-T} (Singlet-Triplet Energy Gap) (kcal/mol)
^{1}**Ia**	-2713.151840	0.796958	-2712.354882	4.8
^{3}**Ia**	-2713.160511	0.797983	-2712.362528	
^{1}**TS-CHA**	-2713.130222	0.797864	-2712.332358	5.6
^{3}**TS-CHA**	-2713.136590	0.795352	-2712.341238	

10.4 Cartesian Coordinates of Computed Structures

[1]Ia

Ru	0.24968200	0.10851200	0.64033600
C	0.43627500	-1.96790100	1.06868200
N	-0.25522600	-3.12431600	1.02393600
C	0.52366600	-4.19087000	1.48416000
H	0.13131000	-5.19474300	1.53323600
C	1.73981500	-3.69273600	1.80802000
H	2.62154300	-4.18307100	2.18639100
N	1.67160400	-2.32253600	1.54889000
C	2.69120300	-1.35561400	1.61701800
N	2.31544500	-0.13565800	1.17509800
C	3.22354700	0.86195800	1.17685000
H	2.88055200	1.82908500	0.83237000
C	4.52798900	0.66536700	1.61529200
C	4.90725800	-0.59414500	2.07592900
H	5.91726500	-0.77605800	2.42890700
C	3.97323100	-1.62865700	2.07933400
H	4.23800500	-2.61726200	2.43398300
C	-1.60182400	-3.28441100	0.52491900
C	-1.77626700	-3.74226800	-0.78975200
C	-3.08829100	-3.91898400	-1.24057900
H	-3.24998300	-4.27751000	-2.25415200
C	-4.19310500	-3.66230600	-0.42067900
C	-3.96547100	-3.20875300	0.88501300
H	-4.81481900	-3.00779900	1.53317000
C	-2.67573000	-3.01857900	1.38649700
C	-0.59797400	-4.03052100	-1.68920500
H	-0.93227200	-4.35019000	-2.67929600
H	0.03732600	-3.14811700	-1.81617800
H	0.03880100	-4.82538400	-1.28370100
C	-5.59976900	-3.88582000	-0.91900700
H	-5.64834200	-3.88611700	-2.01214900
H	-5.98239200	-4.85468400	-0.57492400
H	-6.28562600	-3.11908200	-0.54228800
C	-2.44170100	-2.54681600	2.80098800
H	-3.38953600	-2.38140900	3.31950600
H	-1.87047600	-3.28415500	3.37780300
H	-1.86847900	-1.61237300	2.82459500
C	0.33278600	-0.29681900	-1.31036200
N	1.24873000	-0.41751900	-2.28767400
C	0.62385100	-0.69581500	-3.51048700
H	1.19657300	-0.82368400	-4.41579500
C	-0.71128800	-0.74615700	-3.28760500

H	-1.53186700	-0.93219200	-3.96100600
N	-0.88191000	-0.49897800	-1.92272600
C	-2.04877600	-0.31354000	-1.16874500
N	-1.80031500	0.02697400	0.11439000
C	-2.83056800	0.36265000	0.91324000
H	-2.56675700	0.66981200	1.91662400
C	-4.14389700	0.32108000	0.47086300
C	-4.39866200	-0.09426500	-0.83772300
H	-5.41647400	-0.15342700	-1.20883500
C	-3.33757500	-0.41801300	-1.67752000
H	-3.50597200	-0.72268500	-2.70290300
C	2.66432100	-0.17155900	-2.11769600
C	3.51103000	-1.25366100	-1.83513900
C	4.86560900	-0.97629500	-1.64030900
H	5.54287500	-1.79702900	-1.41709900
C	5.37577100	0.32548100	-1.73819900
C	4.49305300	1.36842500	-2.03904500
H	4.87600600	2.38191600	-2.12617200
C	3.12542900	1.14798700	-2.23636200
C	2.98309100	-2.66554400	-1.75036700
H	3.78099700	-3.36606700	-1.49071300
H	2.19120100	-2.76002600	-1.00077400
H	2.55513900	-2.99289200	-2.70541800
C	6.84976000	0.58602400	-1.54756000
H	7.04618800	1.63019000	-1.28609200
H	7.27157700	-0.05240700	-0.76361400
H	7.40308200	0.37052600	-2.47005500
C	2.18748600	2.29031900	-2.54075900
H	2.74584100	3.20597300	-2.74978000
H	1.55472500	2.07596100	-3.40993000
H	1.51954200	2.48422800	-1.69299100
N	-0.04461400	0.60461400	2.77192200
C	-0.14744100	1.08019900	3.82148400
C	-0.28100900	1.71170900	5.12711900
H	0.67866200	1.69189600	5.65339300
H	-0.59176600	2.75276900	4.99017100
H	-1.02965700	1.18706300	5.72919400
H	5.22615000	1.49386900	1.58921600
H	-4.94752700	0.60795200	1.13881000
N	0.27758600	1.95714400	0.26671000
C	0.44085400	3.03385900	1.06250800
O	1.53186000	3.58640900	1.16669500
C	-0.97033200	4.86918300	1.64392400
H	-0.14995400	5.36887300	2.16504300
H	-1.88896500	4.95827700	2.22586100
C	-1.14803700	5.40800200	0.22082800

H	-1.45332400	6.45833500	0.30765100
H	-0.17372100	5.40562600	-0.28081300
C	-2.15669500	4.61518500	-0.58468700
C	-3.52929800	4.73282600	-0.33069800
C	-1.73242700	3.72849900	-1.58409400
C	-4.45778400	3.98740900	-1.05969600
H	-3.87782000	5.42889300	0.42926600
C	-2.65804500	2.98268300	-2.31744100
H	-0.67273200	3.63541300	-1.80216000
C	-4.02365800	3.11068000	-2.05785200
H	-5.51966400	4.10866900	-0.86465800
H	-2.30991900	2.32143400	-3.10772100
H	-4.74824800	2.55083000	-2.64288200
O	-0.71868300	3.42875400	1.62476500

^{3}Ia

Ru	-0.44670900	0.20529000	0.93277000
C	-0.98436000	2.13135700	0.31047300
N	-0.46167000	3.25716800	-0.21313900
C	-1.44919900	4.22918200	-0.38870400
H	-1.21802400	5.20404200	-0.78921100
C	-2.62338000	3.69269600	0.02606400
H	-3.61602200	4.11176100	0.04897100
N	-2.32429800	2.39807900	0.45214700
C	-3.19553400	1.37119500	0.87394400
N	-2.57619600	0.19126000	1.09290100
C	-3.31527100	-0.87010700	1.46655500
H	-2.76724500	-1.79093900	1.62339500
C	-4.69226100	-0.78537800	1.63600600
C	-5.32381100	0.43981000	1.42184900
H	-6.39649200	0.54008800	1.55234600
C	-4.56580400	1.54331800	1.03219300
H	-5.03176400	2.50550200	0.85695600
C	0.92479100	3.41411100	-0.59176000
C	1.27471800	3.23494100	-1.93783300
C	2.62669400	3.36156800	-2.27337400
H	2.92269800	3.23192300	-3.31130300
C	3.60142400	3.66196300	-1.31571000
C	3.19702900	3.85434100	0.01191300
H	3.94175600	4.10752800	0.76227800
C	1.86099000	3.74014800	0.40028500
C	0.23977300	2.91307300	-2.98853300
H	0.70513500	2.79192700	-3.96977400
H	-0.29701000	1.98886100	-2.75418500
H	-0.51225100	3.70578300	-3.07602400

C	5.05988200	3.75718300	-1.68889400
H	5.19978700	3.82632200	-2.77129500
H	5.53375100	4.63026800	-1.22836100
H	5.60689000	2.87195800	-1.33724500
C	1.43906500	3.95346400	1.83324800
H	2.28820900	4.26420100	2.44729800
H	0.66694100	4.72784200	1.91313800
H	1.01953900	3.03744700	2.26441600
C	-0.24819800	-0.31716500	-0.99649700
N	-1.00487000	-0.75244800	-2.02128400
C	-0.22174500	-0.90761500	-3.17113100
H	-0.65012100	-1.25400900	-4.09859500
C	1.05062600	-0.56892600	-2.84778300
H	1.95038400	-0.56183400	-3.43936900
N	1.02305200	-0.20827400	-1.50203400
C	2.07532500	0.09579300	-0.61921000
N	1.65252800	0.36842200	0.63310000
C	2.56983300	0.59200100	1.58965400
H	2.17956500	0.77888200	2.58118800
C	3.93243600	0.57195900	1.32487400
C	4.35970800	0.30228800	0.02417100
H	5.41716800	0.26128000	-0.21457500
C	3.41741200	0.06795100	-0.97346200
H	3.72072000	-0.17903000	-1.98119800
C	-2.38405300	-1.17361400	-1.90178800
C	-3.40460200	-0.26926300	-2.23506800
C	-4.72288600	-0.69875700	-2.07620300
H	-5.53125200	-0.01517400	-2.32414400
C	-5.03224300	-1.98939400	-1.62381300
C	-3.98070400	-2.86167400	-1.32836900
H	-4.20428200	-3.87053100	-0.99061500
C	-2.63993800	-2.47985100	-1.45915900
C	-3.09425100	1.11280400	-2.75708200
H	-4.01232900	1.68186100	-2.92489200
H	-2.46571600	1.67979800	-2.06402100
H	-2.55507300	1.07049800	-3.71092400
C	-6.47008300	-2.42633200	-1.48796500
H	-6.56605500	-3.31704000	-0.86011800
H	-7.08990200	-1.63175000	-1.05750900
H	-6.89595900	-2.66876000	-2.46937800
C	-1.53328500	-3.44577100	-1.10952200
H	-1.87704200	-4.47833400	-1.21379600
H	-0.65612000	-3.31756000	-1.75128700
H	-1.20158800	-3.31591600	-0.07250100
N	-0.45148300	0.92400700	3.02055700
C	-0.50487600	1.22964800	4.13526400

C	-0.56597700	1.60977600	5.54148300
H	-1.60992400	1.68321400	5.86302300
H	-0.05933700	0.85569600	6.15265800
H	-0.07820100	2.57862200	5.69011700
H	-5.25138400	-1.66658000	1.92838300
H	4.63976300	0.75403600	2.12593800
N	-0.16002700	-1.57518000	1.56567100
C	0.55912200	-2.64519200	2.02784000
O	0.04969200	-3.73467300	2.24479600
C	2.75809600	-3.37968800	2.62633200
H	2.36828400	-3.81803900	3.54976200
H	3.68370200	-2.84040600	2.83685500
C	2.96791800	-4.45960900	1.56051200
H	3.77447800	-5.10738500	1.92658100
H	2.06685900	-5.07498800	1.50131400
C	3.31449200	-3.91044500	0.19380900
C	4.61757500	-3.49486600	-0.11503200
C	2.33241500	-3.83213300	-0.80252900
C	4.93807800	-3.03728500	-1.39438800
H	5.39617600	-3.56586100	0.64131300
C	2.64901300	-3.38093500	-2.08603800
H	1.32420200	-4.17263300	-0.58035900
C	3.95476400	-2.98511600	-2.38765800
H	5.96145900	-2.75441600	-1.62621300
H	1.88426300	-3.37327000	-2.85767400
H	4.21510600	-2.67298000	-3.39621300
O	1.85529800	-2.32340300	2.19192200

[1]TS-CHA

Ru	0.25455400	-0.40648000	0.48671300
C	1.31300000	1.17005800	1.29635800
N	2.56380000	1.67423200	1.37193300
C	2.58663300	2.83348200	2.14954600
H	3.50372300	3.37024800	2.33705200
C	1.31575600	3.07331900	2.55906800
H	0.91387300	3.86953300	3.16417900
N	0.53995000	2.04565900	2.02542900
C	-0.86187500	1.88756600	2.02923200
N	-1.28905600	0.85691400	1.26751800
C	-2.61185600	0.65642300	1.13919100
H	-2.90162700	-0.15474400	0.48450700
C	-3.54797400	1.44967100	1.79287900
C	-3.09888700	2.49104300	2.60492500
H	-3.80229200	3.12560500	3.13451100
C	-1.72879000	2.72379400	2.72407500

H	-1.35233400	3.53422700	3.33675800
C	3.72428100	1.08762700	0.73876500
C	4.19035300	1.63292300	-0.46619600
C	5.30085000	1.02335600	-1.06150300
H	5.68338800	1.43242600	-1.99316500
C	5.93631200	-0.08239800	-0.48852600
C	5.44888200	-0.57398800	0.72968200
H	5.94800000	-1.41779800	1.19950200
C	4.34766500	-0.00183900	1.36757200
C	3.52531500	2.81894500	-1.12219600
H	4.14856900	3.21384200	-1.92850600
H	2.55843200	2.54444600	-1.55577100
H	3.34057900	3.63498200	-0.41603300
C	7.11102400	-0.74669800	-1.16252800
H	7.50476300	-0.14195100	-1.98415500
H	7.92605400	-0.92935900	-0.45420900
H	6.81992100	-1.72206200	-1.57480700
C	3.84707500	-0.53412400	2.68779400
H	4.51074300	-1.31450800	3.06909700
H	3.79064900	0.25756500	3.44435800
H	2.84097300	-0.95560500	2.58802000
C	0.51566400	0.44968300	-1.31579700
N	-0.09305200	1.34762600	-2.11432600
C	0.56931800	1.42648100	-3.34513000
H	0.22487500	2.08605100	-4.12594500
C	1.61113500	0.56160800	-3.30572100
H	2.35250500	0.32080400	-4.04944300
N	1.57717000	-0.02576200	-2.04107100
C	2.28586500	-1.12698900	-1.53983900
N	1.84960800	-1.53583400	-0.32750600
C	2.41024200	-2.63366600	0.21542800
H	2.02266900	-2.92778300	1.18297000
C	3.42163900	-3.34442900	-0.41508500
C	3.88128200	-2.89869600	-1.65660200
H	4.67161100	-3.42929600	-2.17741100
C	3.31017800	-1.76688500	-2.22952100
H	3.64011800	-1.40191800	-3.19413200
C	-1.32942900	2.03027000	-1.79686600
C	-1.26183000	3.26895000	-1.13239900
C	-2.46202600	3.88519600	-0.78355900
H	-2.43141700	4.84114400	-0.26613900
C	-3.70556800	3.31344200	-1.09364900
C	-3.72294500	2.10199600	-1.78418100
H	-4.67659200	1.64955000	-2.04602500
C	-2.54615500	1.43626500	-2.15805700
C	0.06080700	3.93033400	-0.83139600

H	-0.08107500	4.83228700	-0.23010000
H	0.74071600	3.26550000	-0.29428300
H	0.57417700	4.22770900	-1.75390400
C	-4.98202600	4.00614000	-0.68484000
H	-5.01206900	4.16633200	0.40038200
H	-5.06285400	4.99318700	-1.15463400
H	-5.86649800	3.42823000	-0.96753000
C	-2.64409900	0.15014100	-2.94073200
H	-3.38721400	-0.51450000	-2.48771500
H	-2.98034500	0.35925500	-3.96389500
H	-1.70294900	-0.39474600	-2.99959500
N	0.22307600	-1.39897900	2.42634300
C	0.19999800	-1.87720400	3.48124900
C	0.18908900	-2.47791900	4.80986800
H	0.01446200	-3.55629500	4.73530000
H	1.15321800	-2.30858900	5.30101300
H	-0.60271000	-2.03008900	5.41905400
H	-4.60382900	1.24703800	1.65539700
H	3.83793100	-4.22678200	0.05756900
N	-1.10483800	-1.60064200	-0.36113500
C	-0.90489100	-2.51138500	-1.36234700
O	-0.36610400	-2.16144800	-2.40681600
C	-1.87370100	-4.25991300	0.00690100
H	-1.01114500	-4.60052500	0.59163600
H	-2.49588000	-5.12573500	-0.23488400
C	-2.64604100	-3.20700500	0.78956400
H	-2.58827000	-3.37664200	1.86874100
H	-1.93691800	-2.24476400	0.58835900
C	-3.97604100	-2.74936200	0.34323100
C	-4.36300600	-2.78551800	-1.01309400
C	-4.85071900	-2.16465700	1.28393600
C	-5.59479000	-2.26804300	-1.40593600
H	-3.70409600	-3.22590100	-1.75386900
C	-6.07900400	-1.64461200	0.88664100
H	-4.56470500	-2.13562800	2.33293000
C	-6.45300400	-1.69236100	-0.46165400
H	-5.89050600	-2.31759300	-2.44953200
H	-6.75330400	-1.21973000	1.62448300
H	-7.41424300	-1.29450100	-0.77303600
O	-1.39793400	-3.77060100	-1.25619300

^{3}TS-CHA

Ru	0.32484100	-0.41535200	0.52479400
C	1.39009100	1.24598700	1.22581800
N	2.63214900	1.77037200	1.25014300

C	2.65256300	2.97077600	1.96388700
H	3.56455100	3.53101200	2.10062200
C	1.38624300	3.21443100	2.38245800
H	0.98076900	4.03969000	2.94459200
N	0.61899500	2.14401100	1.92397900
C	-0.77916100	1.96639400	1.96502100
N	-1.20862600	0.88879900	1.27233100
C	-2.53217400	0.66917000	1.18348300
H	-2.83445200	-0.17245400	0.57852300
C	-3.46431400	1.48927600	1.80834500
C	-3.01186100	2.57872900	2.55103300
H	-3.71185200	3.23669200	3.05595700
C	-1.64246200	2.83173500	2.62755100
H	-1.26281700	3.67969800	3.18483900
C	3.78765900	1.18805900	0.60495300
C	4.17305500	1.67462900	-0.65232800
C	5.28655200	1.08098600	-1.25734800
H	5.60729300	1.44305800	-2.23086800
C	6.00200400	0.04894700	-0.64165500
C	5.58642900	-0.39016500	0.62258100
H	6.14091600	-1.18332000	1.11786800
C	4.48507800	0.16933900	1.27187900
C	3.41658400	2.78261500	-1.34500700
H	3.94630800	3.11146000	-2.24250900
H	2.41756800	2.45515400	-1.65007700
H	3.28392700	3.65741600	-0.69923500
C	7.20519800	-0.57353700	-1.30580200
H	7.28820200	-0.27947200	-2.35603000
H	8.13000500	-0.26707000	-0.80227300
H	7.16311500	-1.66792100	-1.25740800
C	4.05869800	-0.30147200	2.64094600
H	4.73420800	-1.07528700	3.01454300
H	4.06076100	0.52219600	3.36504700
H	3.04226000	-0.71076200	2.62413000
C	0.50613700	0.27794400	-1.34275100
N	-0.13793500	1.10183200	-2.19481700
C	0.52405100	1.13926700	-3.42766500
H	0.15356900	1.73733900	-4.24521500
C	1.60388700	0.32647800	-3.33803000
H	2.36020800	0.07917300	-4.06445900
N	1.59074100	-0.18968800	-2.04292100
C	2.35488400	-1.20973600	-1.46749400
N	1.95812700	-1.52698200	-0.21532300
C	2.56339300	-2.55398500	0.41180000
H	2.19497000	-2.78021400	1.40433500
C	3.59050900	-3.27788400	-0.17461500

C	4.01453800	-2.92337300	-1.45853300
H	4.81708700	-3.46771500	-1.94540700
C	3.39261000	-1.86920500	-2.11821600
H	3.69089400	-1.58059000	-3.11829600
C	-1.41947600	1.71501300	-1.92307600
C	-1.45887500	2.96826000	-1.29182600
C	-2.71314300	3.48921600	-0.96688700
H	-2.76757100	4.45420800	-0.46908900
C	-3.89829600	2.80292700	-1.26307600
C	-3.81005900	1.57824400	-1.93090800
H	-4.72027400	1.03793300	-2.17698700
C	-2.57988100	1.01607300	-2.29073600
C	-0.19896100	3.74624800	-0.99928400
H	-0.41227800	4.61399900	-0.36915000
H	0.55597400	3.13635900	-0.49843300
H	0.25811400	4.11789200	-1.92467400
C	-5.24157700	3.38128900	-0.89109200
H	-5.17559500	4.01444200	-0.00008100
H	-5.63606600	4.00481800	-1.70296000
H	-5.97672300	2.59196600	-0.70162900
C	-2.53340900	-0.26045500	-3.09330500
H	-3.42321700	-0.86475000	-2.90418000
H	-2.52150000	-0.02984500	-4.16650900
H	-1.65308200	-0.86974000	-2.88474700
N	0.35451100	-1.24153100	2.58108100
C	0.35834600	-1.63997400	3.66793900
C	0.37197500	-2.14543600	5.03594200
H	0.24035100	-3.23237200	5.03560600
H	1.32679900	-1.90298000	5.51390900
H	-0.43942400	-1.68945500	5.61234800
H	-4.51862900	1.26666100	1.69694800
H	4.04539900	-4.10239300	0.36238200
N	-0.89203900	-1.88392700	-0.01266200
C	-0.89502300	-2.72192500	-1.10670700
O	-0.18625300	-2.52652800	-2.08645800
C	-2.35499200	-4.21269500	0.13467400
H	-1.59001200	-4.72327900	0.73016200
H	-3.10757900	-4.94310600	-0.17190900
C	-2.95434400	-3.06171400	0.93281800
H	-2.92892900	-3.24477000	2.01059100
H	-1.98126900	-2.25936900	0.73509100
C	-4.19261300	-2.40931400	0.47406600
C	-4.57399500	-2.40103500	-0.88473900
C	-5.00347400	-1.72044300	1.40245400
C	-5.72663900	-1.73302700	-1.29256700
H	-3.96929000	-2.92707400	-1.61609200

C	-6.15265900	-1.05048900	0.99086300
H	-4.73177500	-1.73013300	2.45563500
C	-6.51684500	-1.05012300	-0.36110300
H	-6.01772700	-1.75428800	-2.33890400
H	-6.77952600	-0.54968200	1.72349200
H	-7.42053700	-0.54053700	-0.68209800
O	-1.74688700	-3.77641800	-1.10274100

11. References

1. Y. Zheng, Y. Tan, K. Harms, M. Marsch, R. Riedel, L. Zhang, E. Meggers, *J. Am. Chem. Soc.* **2017**, *139*, 4322-4325.
2. Z. Zhou, S. Chen, J. Qin, X. Nie, X. Zheng, K. Harms, R. Riedel, K. N. Houk, E. Meggers, *Angew. Chem. Int. Ed.* **2019**, *58*, 1088-1093.
3. A. Takahashi, Y. Hirose, H. Kusama, N. Iwasawa, *Chem. Commun.* **2008**, 609-611.
4. H. Lebel, K. Huard, S. Lectard, *J. Am. Chem. Soc.* **2005**, *127*, 14198-14199.
5. G.-S. Liu, Y.-Q. Zhang, Y.-A. Yuan, H. Xu, *J. Am. Chem. Soc.* **2013**, *135*, 3343-3346.
6. D. Li, T. Wu, K. Liang, C. Xia, *Org. Lett.* **2016**, *18*, 2228-2231.
7. D. Zhao, X.-H. Liu, Z.-Z., Shi, C.-D., Zhu, P. Wang W.-Y. Sun, *Dalton Trans.* **2016**, *45*, 14184-14190.
8. A. Pilevar, A. Hosseini, J. Becker, P. R. Schreiner, *J. Org. Chem.* **2019**, *84*, 12377-12386.
9. W.-X. Hu, P.-R., Li, G. Jiang, C.-M. Che, J. Chen, *Adv. Synth. Catal.* **2010**, *352*, 3190-3194.
10. R. Bag, D. Sar, T. Punniyamurthy, *Org. Biomol. Chem.* **2016**, *14*, 3246-3255.
11. J. Park, S. F. Pedersen, *Tetrahedron.* **1992**, *48*, 2069-2080.
12. R. Bag, T. Punniyamurthy, *Chemistryselect* **2018**, *3*, 6152-6155.
13. B. Liu, J. Yan, R. Huang, W. Wang, Z. Jin, G. Zanoni, P. Zheng, S. Yang, Y. R. Chi, *Org. Lett.* **2018**, *20*, 3447-3450.
14. M. Quan, N. Butt, J. Shen, K. Shen, D. Liu, W. Zhang, *Org. Biomol. Chem.* **2013**, *11*, 7412-7419.
15. X. Tang, S. Woodward, N. Krause, *Eur. J. Org. Chem.* **2009**, 2836-2844.
16. M. Cleij, A. Auchelas, R. Furstoss, *J. Org. Chem.* **1999**, *64*, 5029-5035.
17. B. Yang, Z. Lu, *Chem. Commun.* **2017**, *53*, 12634-12637.
18. S. Sopeña, E. Martin, E. C. Escudero-Adán, A. W. Kleij, *ACS Catal.* **2017**, *7*, 3532-3539.
19. J. Qin, V. A. Larionov, K. Harms, E. Meggers, *ChemSusChem.* **2019**, *12*, 320-325.
20. R. P. Reddy, H. M. L. Davies, *Org. Lett.* **2006**, *8*, 5013-5016.
21. H. Lebel, L. M. Laparra, M. Khalifa, C. Trudel, C. Audubert, M. Szponarski, C. D. Leduc, E. Azek, M. Ernzerhof, *Org. Biomol. Chem.* **2017**, *15*, 4144-4158.
22. *X-Area*, STOE & Cie GmbH, Darmstadt, Germany, **2018**.
23. *X-RED32*, STOE & Cie GmbH, Darmstadt, Germany, **2018**.
24. *LANA-Laue Analyzer*, STOE & Cie GmbH, Darmstadt, Germany, **2019**.

25. G. M. Sheldrick, *Acta Crystallogr., Sect. A: Found. Adv.* **2015**, *71*, 3-8.

26. G. M. Sheldrick, *Acta Crystallogr., Sect. C: Sturct. Chem.* **2015**, *71*, 3-8.

27. C. B. Hübschle, G. M. Sheldrick, B. Dittrich, *J. Appl. Crystallogr*. **2011**, *44*, 1281-1284.

28. S. I. Ivlev, M. Conrad, F. Kraus, *Z. Kristallogr. - Cryst. Mater*. **2019**, *234*, 415-418.

29. D. Kratzert, I. Krossing, *J. Appl. Crystallogr*. **2018**, *51*, 928-934.

30. Z. Zhou, S. Chen, Y. Hong, E. Winterling, Y. Tan, M. Hemming, K. Harms, K. N. Houk, E. Meggers, *J. Am. Chem. Soc.* **2019**, *141*, 19048-19057.

31. Gaussian 16, Revision C.01, M. J. Frisch, G. W. Trucks, H. B. Schlegel, G. E. Scuseria, M. A. Robb, J. R. Cheeseman, G. Scalmani, V. Barone, G. A. Petersson, H. Nakatsuji, X. Li, M. Caricato, A. V. Marenich, J. Bloino, B. G. Janesko, R. Gomperts, B. Mennucci, H. P. Hratchian, J. V. Ortiz, A. F. Izmaylov, J. L. Sonnenberg, D. Williams-Young, F. Ding, F. Lipparini, F. Egidi, J. Goings, B. Peng, A. Petrone, T. Henderson, D. Ranasinghe, V. G. Zakrzewski, J. Gao, N. Rega, G. Zheng, W. Liang, M. Hada, M. Ehara, K. Toyota, R. Fukuda, J. Hasegawa, M. Ishida, T. Nakajima, Y. Honda, O. Kitao, H. Nakai, T. Vreven, K. Throssell, J. A. Montgomery, Jr., J. E. Peralta, F. Ogliaro, M. J. Bearpark, J. J. Heyd, E. N. Brothers, K. N. Kudin, V. N. Staroverov, T. A. Keith, R. Kobayashi, J. Normand, K. Raghavachari, A. P. Rendell, J. C. Burant, S. S. Iyengar, J. Tomasi, M. Cossi, J. M. Millam, M. Klene, C. Adamo, R. Cammi, J. W. Ochterski, R. L. Martin, K. Morokuma, O. Farkas, J. B. Foresman, and D. J. Fox, Gaussian, Inc., Wallingford CT, **2019**.

32. (a) M. Head-Gordon, J. A. Pople, M. J. Frisch, MP2 Energy Evaluation by Direct Methods. *Chem. Phys. Lett.* **1988**, *153*, 503. (b) A. D. Becke, Density-Functional Thermochemistry. III. The Role of Exact Exchange. *J. Chem. Phys.* **1993**, *98*, 5648. (c) C. Lee, W. Yang, R. G. Parr, Development of the Colle-Salvetti Correlation-Energy Formula into a Functional of the Electron Density. *Phys. Rev. B: Condens. Matter Mater. Phys.* **1988**, *37*, 785. (d) S. H. Vosko, L. Wilk, M. Nusair, Accurate Spin-Dependent Electron Liquid Correlation Energies for Local Spin Density Calculations: a Critical Analysis. *Can. J. Phys.* **1980**, *58*, 1200. (e) P. J. Stephens, F. J. Devlin, C. F. Chabalowski, M. J. Frisch, Ab Initio Calculation of Vibrational Absorption and Circular Dichroism Spectra Using Density Functional Force Fields. *J. Phys. Chem.* **1994**, *98*, 11623.

33. S. Grimme, J. Antony, S. Ehrlich, H. A. Krieg, Consistent and Accurate ab initio Parametrization of Density Functional Dispersion Correction (DFT-D) for the 94 Elements H-Pu. *J. Chem. Phys.* **2010**, *132*, 154104.

34. P. J. Hay, W. R. Wadt, Ab initio Effective Core Potentials for Molecular Calculations. Potentials for K to Au Including the Outermost Core Orbitals. *J. Chem. Phys.* **1985**, *82*, 299.
35. S. Grimme, Supramolecular Binding Thermodynamics by Dispersion-Corrected Density Functional Theory. *Chem. Eur. J.* **2012**, *18*, 9955-9964.
36. Y. Zhao, D. G. Truhlar, The M06 Suite of Density Functionals for Main Group Thermochemistry, Thermochemical Kinetics, Noncovalent Interactions, Excited States, and Transition Elements: Two New Functionals and Systematic Testing of Four M06-Class.
37. (a) U. Haussermann, M. Dolg, H. Stoll, H. Preuss, P. Schwerdtfeger, R. M. Pitzer, Accuracy of Energy-Adjusted Quasirelativistic Ab initio Pseudopotentials. *Mol. Phys.* **1993**, *78*, 1211. (b) W. K chle, M. Dolg, H. Stoll, H. Preuss, Energy-Adjusted Pseudopotentials for the Actinides. Parameter Sets and Test Calculations for Thorium and Thorium Monoxide. *J. Chem. Phys.* **1994**, *100*, 7535.
38. A. V. Marenich, C. J. Cramer, D. G. Truhlar, Universal Solvation Model Based on Solute Electron Density and on a Continuum Model of the Solvent Defined by the Bulk Dielectric Constant and Atomic Surface Tensions. *J. Phys. Chem. B* **2009**, *113*, 6378.
39. Legault, C. Y. CYLview, 1.0b; Université de Sherbrooke, **2009**; http://www.cylview.org.
40. Spartan ’18, Wavefunction, Inc. Irvine, CA.
41. J. D. Chai, M. Head-Gordon, M. Long-Range Corrected Hybrid Density Functionals with Damped Atom-Atom Dispersion Corrections. *Phys. Chem. Chem. Phys.* **2008**, *10*, 6615.
41. X. Lin, C. Zhao, C.-M. Che, Z. Ke, D. L. Phillips, A DFT Study on the Mechanism of $Rh_2^{II,II}$-Catalyzed Intramolecular Amidation of Carbamates. *Chem. Asian J.* **2007**, *2*, 1101-1108.
